# Iron-Catalyzed Stereospecific Heterocycle N‑Glycosylation with Glycal Epoxides

**DOI:** 10.1021/acs.orglett.5c05451

**Published:** 2026-02-20

**Authors:** Xiao-Wen Zhang, Dakang Zhang, Zixiang Jiang, Hao Xu

**Affiliations:** Department of Chemistry, 8244 Brandeis University, 415 South Street, Waltham, Massachusetts 02453, United States

## Abstract

Stereospecific N-glycosylation of heterocycles with glycal epoxides could readily provide valuable building blocks for drug discovery, but heterocycle N-glycosylation with a pyranose-based glycal epoxide is still difficult using existing methods. We report herein an iron-catalyzed stereospecific heterocycle N-glycosylation method for these glycal epoxides in high yields with low catalyst loadings. This method is functional-group-tolerant and effective for a wide variety of functionalized, complex glycal epoxides and heterocycles.

## Graphical abstract

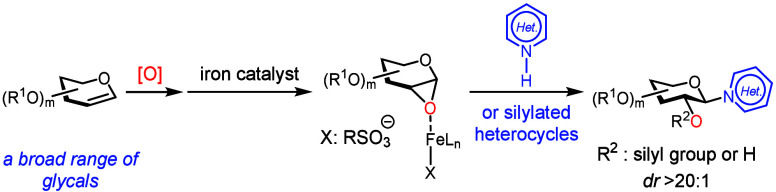

Heterocycle N-glycosylation plays an important role in the discovery of nucleoside-based therapeutics for infectious diseases (Figure 1A). The pioneering Vorbrüggen reaction^1^ (reaction of silylated pyrimidine with glycosyl acetate in the presence of a Lewis acid) is still practiced today and can be scaled up to the multi-kilogram scale for the synthesis of a complex nucleoside analogue.^2^ However, development of effective and functional-group-tolerant catalytic methods for heterocycle N-glycosylation remains a challenge.^3^ Lewis basic heterocyclic nitrogen atoms often deactivate Lewis acid catalysts and promoters, so that the glycosylation reaction requires forcing conditions. Additionally, the basic reaction medium that facilitates most C–N bond formation reactions is less compatible with functional groups presented in commonly used glycosyl donors.

**Figure 1:**
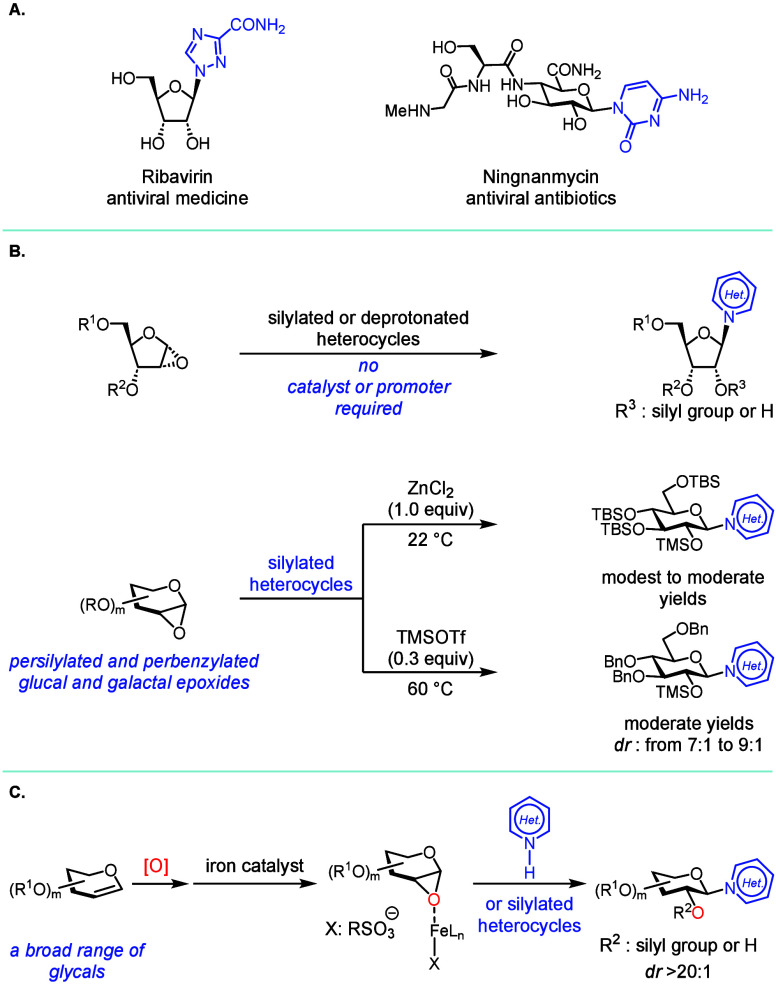
(A) Biologically active N-glycosylated heterocycles: ribavirin and ningnanmycin. (B) Existing heterocycle N-glycosylation methods with glycal epoxides. (C) This research: iron-catalyzed stereospecific heterocycle N-glycosylation with glycal epoxides.

These challenges inspired the development of an array of valuable heterocycle N-glycosylation methods,^4–26^ but a mechanistically distinct approach could capitalize on the stereospecific N-glycosylation with glycal epoxides (Figure 1B). It is known that a furanose-based glycal epoxide can readily glycosylate a silylated or deprotonated heterocycle in the absence of a catalyst or a promoter,^27–32^ but stereospecific N-glycosylation with a pyranose-based glycal epoxide is still difficult. The existing methods require electron-rich, persilylated and perbenzylated glycosyl donors, and even these have limitations (Figure 1B).^32,33^

A stoichiometric ZnCl_2_-mediated method afforded an N-glycosylated thymine in a modest yield.^32^ An N-glycosylation promoted by a sub-stoichiometric amount of TMSOTf had to be operated at elevated temperatures leading to decreased dr (Figure 1B).^33^ Thus, a generally applicable and functional-group-tolerant heterocycle N-glycosylation method with glycal epoxides has yet to be developed. We have recently discovered the iron-catalyzed highly stereospecific glycosylation of hindered secondary sugar acceptors with glycal epoxides.^34^ Building upon this discovery, we report here an iron-catalyzed stereospecific heterocycle N-glycosylation method that is functional-group-tolerant and effective for a wide variety of complex glycal epoxides (Figure 1C).^35^

Electron-deficient glucuronic-acid-based glycosyl donors are less reactive and therefore difficult to activate.^36–40^ Jacobsen^36^ and we^34^ have recently developed catalytic methods that are effective in promoting stereospecific glycosylation with glucuronic ester epoxides.^34,36,37^ Therefore, we selected glucuronal **2** as a model substrate for reaction discovery (Figure 2). Epoxidation of glucuronal **2** in a biphasic reaction medium with oxone^41^ quantitatively afforded the corresponding glucuronic ester α-epoxide (Figure S1; dr > 20:1 and ^3^ *J*_H1–H2_ = 2.6 Hz),^42^ which was azeotropically dried and used directly. Bis-silylation of uracil (**3**) with *N*,*O*-bis­(trimethylsilyl)­trifluoroacetamide (BSTFA)^43^ followed by solvent exchange (MeCN → CH_2_Cl_2_) quantitatively generated the activated glycosyl acceptor. Extensive exploration of catalysts and other reaction parameters revealed that readily available, hemin-derived iron catalyst **1a** (5 mol %) used for stereospecific glycosylation with hindered sugar acceptors^34^ is optimal in promoting this N-glycosylation at 0 °C in 2 h to afford **4** (91% yield and dr > 20:1). Notably, glucuronic ester epoxide **S1** is stable to catalyst **1a** in the absence of a nucleophile. Further experiments suggested that both iron catalyst **1a** and bis-silylation of glycosyl acceptor **3** are crucial for the effective glycosylation (Figure 2. entries 1 and 2). Replacement of catalyst **1a** with either AgOTf, TMSOTf, Fe­(OTf)_2_, or iron catalyst **1b** ^44,45^ resulted in significantly lower reactivity (<5% conversion in 2 h in Table S1). The glycosylation in a prolonged time (24 h) afforded **4** in 13–21% yields with a variety of byproducts from non-productive glycal epoxide decomposition (entries 3–6 of Figure 2). We observed that this glycosylation can be carried out in acetonitrile used for pyrimidine bis-silylation without solvent exchange, albeit with a slower rate (entry 7) and that N-glycosylation product **4** can still be obtained in 90% yield with a low catalyst loading (2 mol %) by increasing the reaction time to 6 h (entry 8).

**Figure 2:**
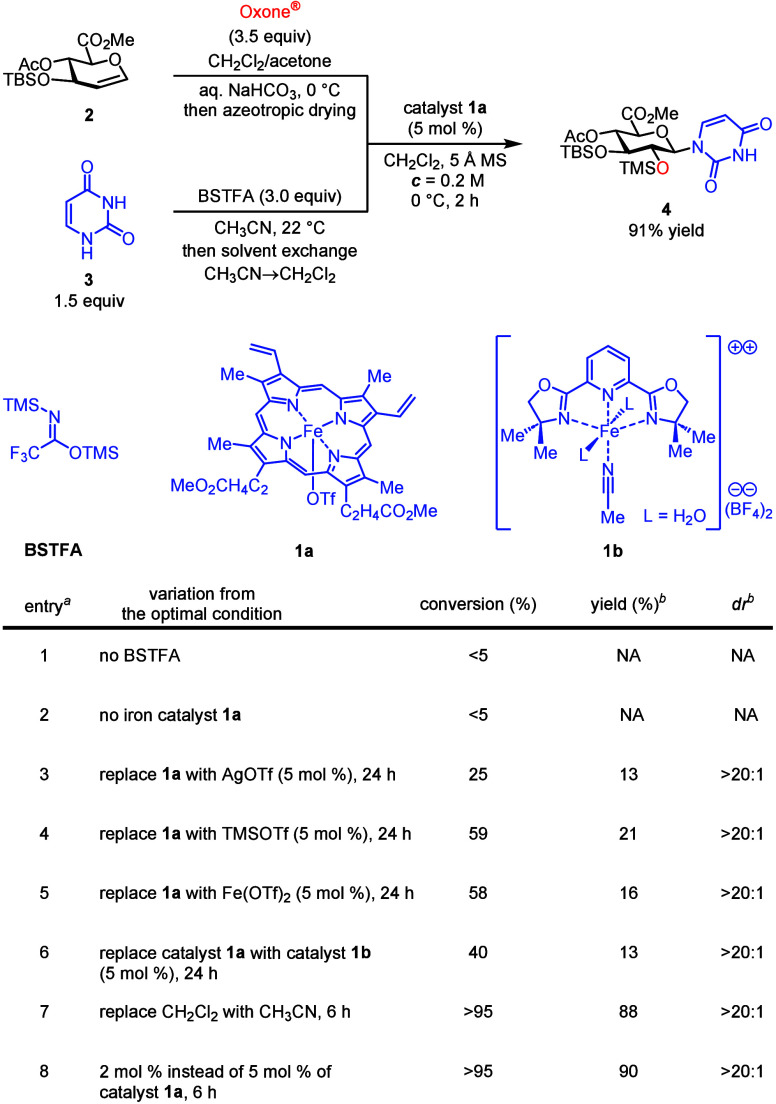
Catalyst discovery for the iron-catalyzed stereospecific heterocycle N-glycosylation with glycal epoxides. ^*a*^Epoxidation was carried out in a biphasic reaction medium with oxone and acetone. The glycal epoxide was dried azeotropically with toluene, assayed by ^1^H NMR, and then directly used. The glycosylation was carried out at 0 °C in CH_2_Cl_2_. The reaction was quenched by methanol and imidazole for conversion measurement. ^*b*^Isolated yield; dr was determined by ^1^H NMR analysis. Iron­(III) porphyrin catalyst **1a** was formed *in situ* from the corresponding iron porphyrin chloride (6 mol %) and AgOTf (5 mol %) to avoid AgOTf in excess.

With optimal catalyst **1a** confirmed, we explored a variety of glycals and heterocycles to determine the generality of this method (Figures 3 and 4). An N-glycosylated uronic ester is a valuable building block for the synthesis of Ningnanmycin-type antibiotics and gougerotin.^46^ Therefore, we are particularly interested in heterocycle N-glycosylation with glycal epoxides derived from highly electron-deficient glucuronal **S2** and galactoronal **S4** (Figure 2). The epoxides obtained from these two glycals can be smoothly converted to the N-glycosylated uracils in good yields (products **4**–**6**; dr > 20:1). Next, we examined an array of electronically differentiated glucals and galactals as well as 6-deoxy glucal and xylal: all of them are excellent substrates, and the corresponding glycal epoxides can readily N-glycosylate bis-silylated uracil in an excellent yield (products **7**–**13**; dr > 20:1). To probe for the functional group compatibility and synthetic utility of this method, we subsequently evaluated a range of readily available, disaccharide-based glycosyl donors,^34^ including the epoxides derived from glucosamine (GlcN)-α-1,6-glucose (Glu), GlcN-α-1,4-glucuronic acid (GlcA), GlcN-α-1,3-Glu, and GlcN-β-1,3-Glu as well as those from maltose and lactose. Using iron catalyst **1a**, the N-glycosylation with all of these donors afforded single diastereomeric products in a high yield (products **14**–**19**; dr > 20:1). It is also worth mentioning that bis-silylated thymine and *N*-benzoyl cytosine are both compatible with this method, affording N-glycosylated thymines and cytosines in good yields (products **20**–**26**; dr > 20:1).

**Figure 3:**
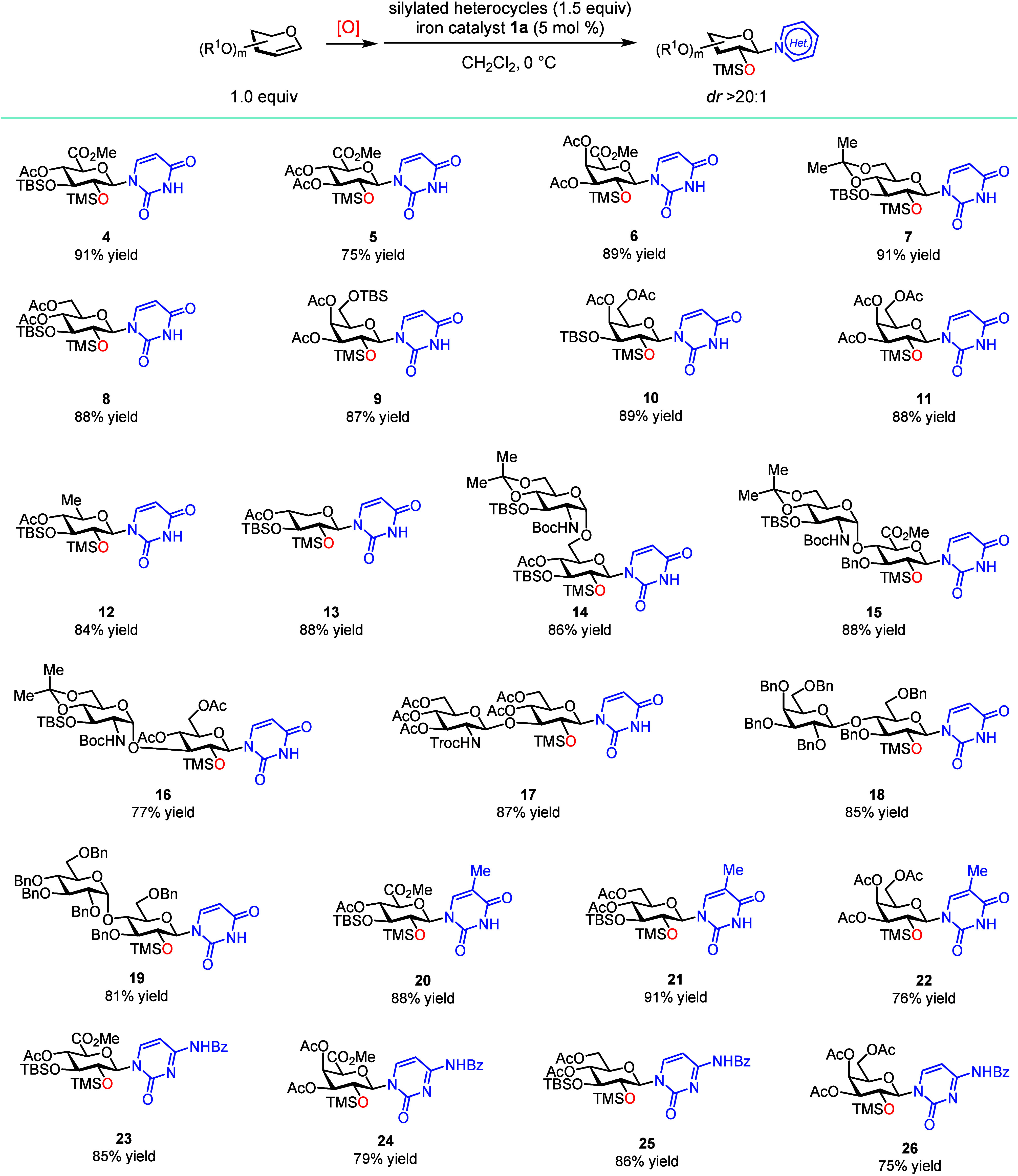
Substrate scope for iron-catalyzed stereospecific pyrimidine N-glycosylation with glycal epoxides. All yields are isolated yields of iron catalyst **1a** (5 mol %). See the Supporting Information for experimental details.

**Figure 4:**
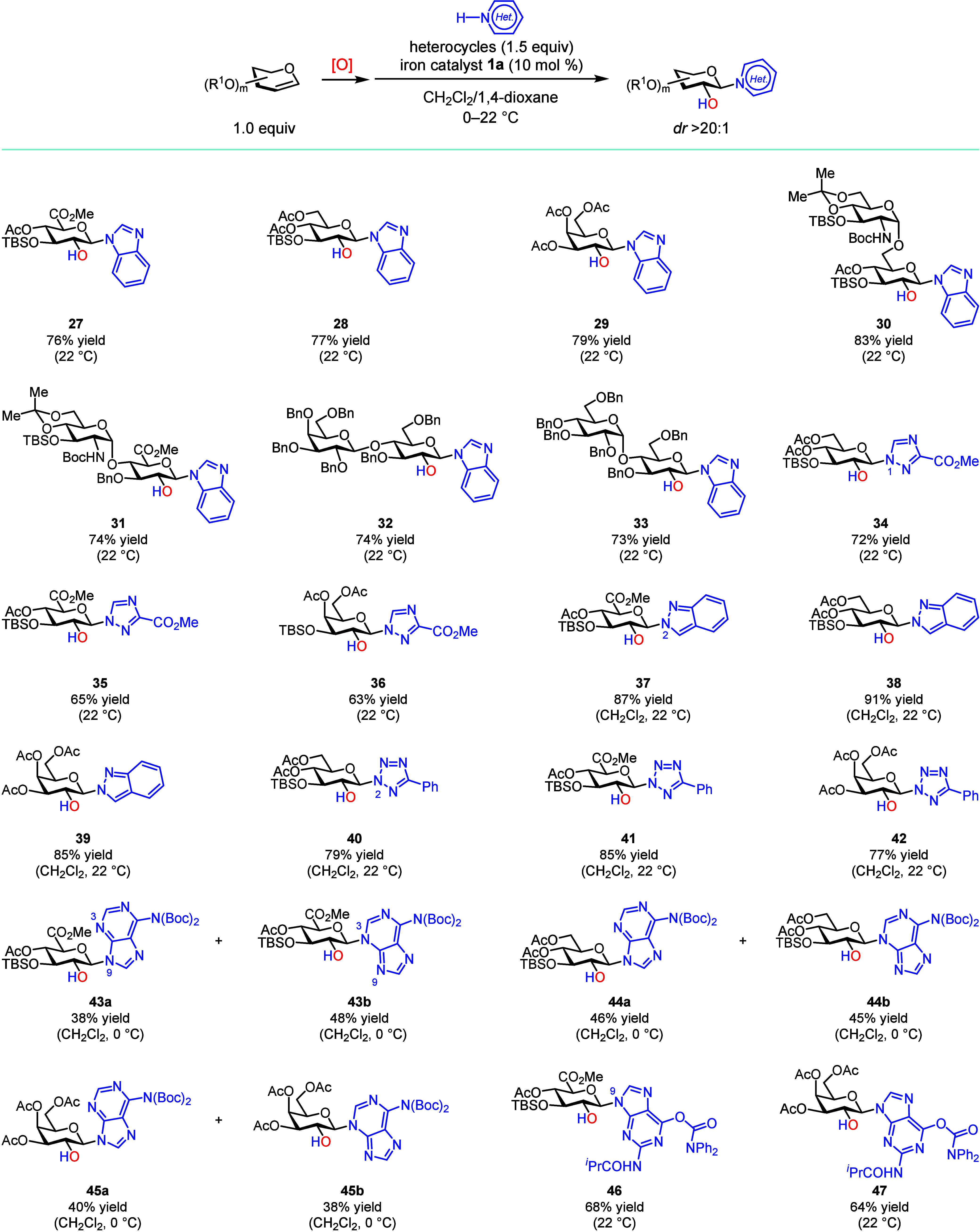
Substrate scope for the iron-catalyzed stereospecific heterocycle N-glycosylation with glycal epoxides. All yields are isolated yields, with iron catalyst **1a** (10 mol %). See the Supporting Information for experimental details.

Interestingly, heterocycles with multiple Lewis basic nitrogen atoms can directly participate in this iron-catalyzed stereospecific N-glycosylation without BSTFA activation (Figure 4). 1,4-Dioxane is a necessary co-solvent for heterocycles that have low solubility in CH_2_Cl_2_, and a longer reaction time is often needed for full conversion. Ribosylated benzimidazoles are promising inhibitors of human cytomegalovirus (HCMV);^47^ therefore, we first evaluated benzimidazole N-glycosylation with an array of functionalized, pyranose-based glycal epoxides. All of these glycosylations afford the desired products in good yield (products **27**–**33**; dr > 20:1). 1,2,4-Triazoles are synthetically valuable because of their relevance to antiviral medicine ribavirin.^48^ We observed that the catalytic N-glycosylation occurs regioselectively at the triazole N1 position in decent yields (corresponding products **34**–**36**; dr > 20:1; see the Supporting Information for details). Furthermore, we explored the N-glycosylation of indazole and tetrazole: both of the regioselectively N-glycosylated heterocycles were isolated in excellent yields (corresponding products **37**–**42**; dr > 20:1; see the Supporting Information for details).

Most known purine N-glycosylation methods predominantly afford N9-glycosylated adenines. Initially formed N3-glycosylated adenine undergoes an irreversible N3 to N9 transglycosylation, presumably by formation of 3,9-N,N′-diglycosylated adenine and cleavage of the N3 glycoside.^49,50^ However, the iron-catalyzed glycosylation of bis­(N6-Boc)-protected adenine **S24** affords both N9-glycosylated (**43a**–**45a**) and N3-glycosylated (**43b**–**45b**) adenines in an excellent combined yield. Notably, **43a** and **43b** do not interconvert under the reaction conditions (Figure S7), and they are readily separable by column chromatography, providing an expedient way for the synthesis of 3-isoadenosine analogues. Interestingly, the iron-catalyzed N-glycosylation of protected guanine **S27** exclusively generated the N9-glycosylated guanines (**46** and **47**).

In conclusion, we have developed an iron-catalyzed highly stereospecific heterocycle N-glycosylation method with pyranose-based glycal epoxides. This method is effective for a wide variety of glycals and heterocycles, and it is compatible with an array of functional groups often used in complex glycan synthesis. Our current effort focuses on applications of this method in rapid synthesis of small-molecule therapeutics.

## Supplementary Material



## Data Availability

The data underlying this study are available in the published article and its Supporting Information.

## Notes

Experimental procedure, characterization data for all new compounds, and selected NMR spectra (PDF)
