# Supplementary material for: Iron-Catalyzed Stereospecific Heterocycle N‑Glycosylation with Glycal Epoxides
Source: Org Lett. 2026 Feb 20;28(9):2859–64. doi: 10.1021/acs.orglett.5c05451 (PMC12949690; doi:10.1021/acs.orglett.5c05451)
Supplement: Supplementary file 1 [file ol5c05451_si_001.pdf]

Supporting Information

Iron-Catalyzed Stereospecific Heterocycle *N*-Glycosylation with Glycal Epoxides

Xiao-Wen Zhang, Dakang Zhang, Zixiang Jiang, and Hao Xu*

haohxu@brandeis.edu

Department of Chemistry, Brandeis University, 415 South Street, Waltham, Massachusetts 02453, United States

A. General Information

B. Catalyst Discovery for the Iron-Catalyzed Stereospecific Heterocycle *N*-Glycosylation with Glycal Epoxides

C. General Procedures for the Iron-Catalyzed Stereospecific Pyrimidine *N*-Glycosylation with Glycal Epoxides

D. General Procedures for the Iron-Catalyzed Stereospecific Heterocycle *N*-Glycosylation with Glycal Epoxides

E. References

F. NMR Spectra

A. General Information

General Procedures. All reactions were performed in oven-dried or flame-dried round-bottom flasks and vials. Stainless steel syringes and cannula were used to transfer air- and moisture-sensitive liquids. Flash chromatography was performed using silica gel 60 (230−400 mesh) from Sigma–Aldrich.

Materials. Commercial reagents were purchased from Sigma–Aldrich, TCI, Oakwood Chemicals, Combi-Blocks, Chem-Impex, Thermo Fischer Scientific and used as received. All solvents were used after being freshly distilled unless otherwise noted.

Instrumentation. Proton nuclear magnetic resonance (^{1}H NMR) spectra and carbon nuclear magnetic resonance (^{13}C NMR) spectra were recorded on Advance NEO 400 (400 MHz) and Varian 400-MR (400 MHz). Chemical shifts for protons are reported in parts per million downfield from tetramethylsilane and are referenced to the NMR solvent residual peak ($CHCl_3$ δ 7.26, acetone-d_6 δ 2.05). Chemical shifts for carbons are reported in parts per million downfield from tetramethylsilane and are referenced to the carbon resonances of the NMR solvent ($CDCl_3$ δ 77.0, acetone-d_6 δ 29.8). Data are represented as follows: chemical shift, multiplicity (br = broad, s = singlet, d = doublet, t = triplet, q = quartet, quint = quintet, m = multiplet), coupling constants in Hertz (Hz), and integration. The mass spectroscopic data were obtained using a Bruker timsTOF Pro instrument by electrospray ionization (ESI). Infrared (IR) spectra were obtained using a Nicolet IR200 spectrometer with a diamond ATR. Data are represented as follows: frequency of absorption (cm^{-1}) and absorption strength (s = strong, m = medium, w = weak).

Abbreviations Used: EtOAc–ethyl acetate, CH_2Cl_2–dichloromethane, Et_2O–diethyl ether, MeCN–acetonitrile, PhMe–toluene, TMSOTf–trimethylsilyl trifluoromethanesulfonate, TBSCl−*tert*-butyldimethylsilyl chloride, Ac_2O−acetic anhydride, AcCl−acetyl chloride, AcOH−acetic acid, DMAP–4-dimethylaminopyridine, TLC–thin layer chromatography, AgOTf−silver trifluoromethanesulfonate. BSTFA−*N*,*O*-bis(trimethylsilyl)trifluoroacetamide,

DMDO−dimethyldioxirane, Troc−2,2,2-trichloroethoxycarbonyl, Boc−*tert*-Butyloxycarbonyl, Bz−benzoyl, Bn−benzyl.

.

B. Catalyst Discovery for the Iron-Catalyzed Stereospecific Heterocycle *N*-Glycosylation with Glycal Epoxides

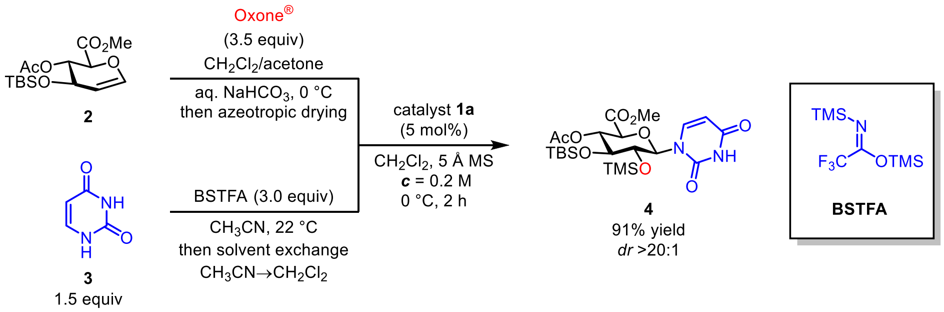

a. The Procedure for Glycal Epoxidation

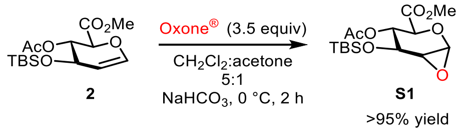

To a 25 mL flask equipped with a stir bar at 0 °C were added glycal **2**[1] (33.0 mg, 0.1 mmol, 1.0 equiv) in CH_2Cl_2/acetone mixture (v/v: 5:1, 1.7 mL) and saturated aqueous $NaHCO_3$ solution (2.8 mL), followed by addition of Oxone® ($KHSO_5 \cdot 0.5KHSO_4 \cdot 0.5K_2SO_4$) (215.5 mg, 0.35 mmol, 3.5 equiv) in H_2O (1.8 mL) dropwise. After stirring vigorously at 0 °C for 2 h, the reaction mixture was extracted with CH_2Cl_2 (3 mL × 3). The combined organic phase was dried over anhydrous Na_2SO_4 and concentrated *in vacuo*. The residue was further azeotropically dried with anhydrous toluene (1.0 mL × 3). The obtained glycal epoxide **S1** was assayed by ^{1}H NMR to get the yield (>95% yield) and diastereomeric ratio (*dr* > 20:1) and directly used in the next step.

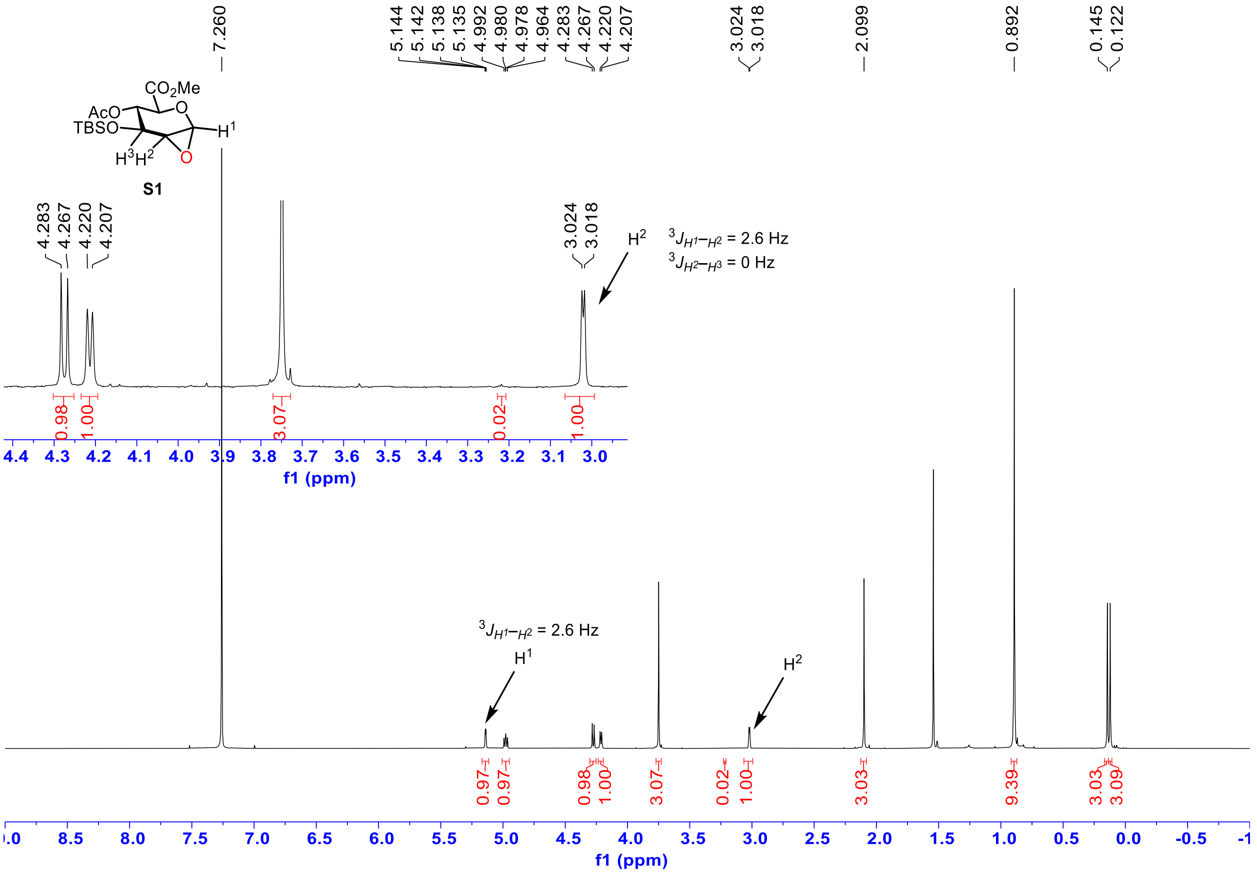

Figure S1a. Stereochemistry Determination of Glycal Epoxide **S1**.

The stereochemistry of the glycal epoxide **S1** was determined by ^{1}H NMR analysis. It is known that the C(2)–H chemical shift of a glucal α-epoxide is around 3.0 ppm, whereas that of a glucal β-epoxide is around 3.2 ppm.[2]

Additionally, the $^{3}J_{H1–H2}$ value for a typical glucal α-epoxide is around 2.6 Hz, reflecting the dihedral angle ($\Phi_{1,2}$) that approaches 0°, whereas the $^{3}J_{H2–H3}$ value is < 0.5 Hz because the dihedral angle ($\Phi_{2,3}$) approaches 90°, characteristic of a *trans*-relationship between H^2 and H^3 that is pseudo-axial.[2] As a result, the H^2 signal of a glucal α-epoxide appears as a doublet.

In contrast, while a glucal β-epoxide retains a similar $\Phi_{1,2}$ and thus a comparable ${}^3J_{H1-H2}$, its $\Phi_{2,3}$ is around 45°, leading to a ${}^3J_{H2-H3}$ around 1.7 Hz.[2] This causes the H^2 signal to appear as a doublet of doublets (though often observed as a triplet due to the broadening line width).

Based on these data, we have assigned glycal epoxide **S1** as an α-epoxide (*dr* > 20:1) with ${}^3J_{H1-H2}$ = 2.6 Hz and ${}^3J_{H2-H3}$ = 0 Hz. This assignment was further corroborated by the stereochemistry analysis of the iron-catalyzed heterocycle *N*-glycosylation product **4**.

b. The Procedure of Catalyst Discovery for the Iron-Catalyzed Stereospecific Heterocycle *N*-Glycosylation with Glycal Epoxides

To a flame-dried sealable 2-dram vial equipped with a stir bar was added a solution of uracil **3** (16.8 mg, 0.15 mmol, 1.5 equiv) in anhydrous CH_3CN (0.5 mL), followed by the addition of *N*,*O*-bis(trimethylsilyl)trifluoroacetamide (BSTFA) (80 μL, 0.30 mmol, 3.0 equiv). After stirring at room temperature for 30 min, the reaction mixture was concentrated *in vacuo,* and the obtained bis-silylated uracil was used directly in the next step.

To a flame-dried sealable 2-dram vial equipped with a stir bar were added the bis-silylated uracil (0.15 mmol, 1.5 equiv) obtained from the previous step and freshly activated 5 Å molecular sieves, powder (*ca.* 100 mg). After the vial was evacuated and backfilled with N_2 three times, a catalyst (0.005 mmol, 5 mol %) in anhydrous CH_2Cl_2 (0.1 mL) was added at −40 °C dropwise into the mixture. After the mixture was stirred at −40 °C for 5 min, the aforementioned glycal epoxide **S1** (0.1 mmol, 1.0 equiv) in anhydrous CH_2Cl_2 (0.4 mL) was added dropwise. The reaction mixture was kept at −40 °C for an additional 3 min before being warmed up to 0 °C. The reaction mixture was stirred at 0 °C for another 2 h and then quenched with MeOH (50 μL) and imidazole (8.5 mg in 1 mL CH_2Cl_2) at the same temperature. The mixture was further stirred at room temperature for 5 min. The mixture was then filtered through a piece of cotton and eluted with EtOAc (2 mL × 2). The organic layer was concentrated *in vacuo*. The *dr* was determined based on the ^1H NMR analysis of the crude reaction mixture. The residue was

purified through a silica gel flash column (hexanes/EtOAc: from 20:1 to 1:1) to afford the desired *N*-glycosylation product **4**.

Table S1. Catalyst Discovery for the Iron-Catalyzed Stereospecific Heterocycle *N*-Glycosylation with Glycal Epoxides

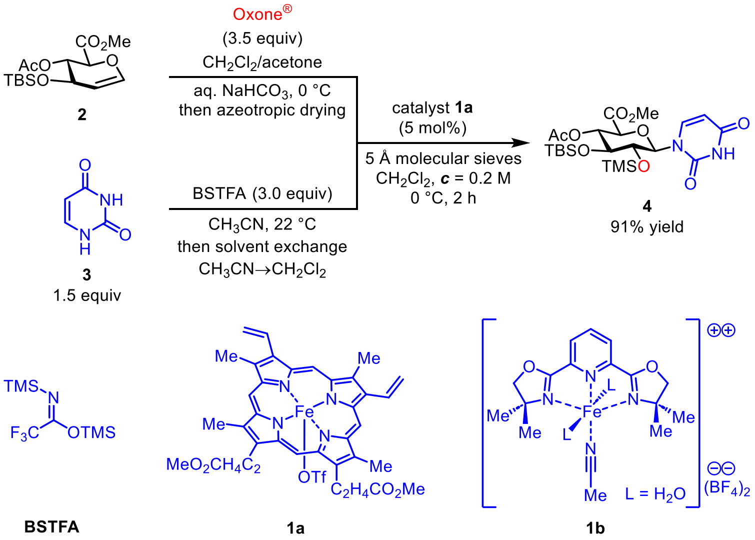

entry[a]	variation from the optimal conditions	conversion (%)	yield (%)[b]	*dr*[b]
1	no BSTFA	<5	NA	NA
2	no catalyst **1a**	<5	NA	NA
3	replace catalyst **1a** with AgOTf (5 mol%), 2 h	<5	NA	NA
4	replace catalyst **1a** with AgOTf (5 mol%), 24 h	25	13	>20:1
5	replace catalyst **1a** with TMSOTf (5 mol%), 2 h	<5	NA	NA
6	replace catalyst **1a** with TMSOTf (5 mol%), 24 h	59	21	>20:1
7	replace catalyst **1a** with $Fe(OTf)_2$ (5 mol%), reaction time: 2 h	<5	NA	NA
8	replace catalyst **1a** with $Fe(OTf)_2$ (5 mol%), reaction time: 24 h	58	16	>20:1
9	replace catalyst **1a** with catalyst **1b** (5 mol%), reaction time: 2 h	<5	NA	NA
10	replace catalyst **1a** with catalyst **1b** (5 mol%), reaction time: 24 h	40	13	>20:1
11	replace CH_2Cl_2 with CH_3CN	>95	88	>20:1
12	2 mol% instead of 5 mol% of catalyst **1a**, 6 h	>95	90	>20:1

[a]Epoxidation was carried out in a biphasic reaction medium with Oxone® and acetone. The glycal epoxide was dried azeotropically with toluene, assayed by ^{1}H NMR, and then directly used. The glycosylation was carried out at 0 °C in CH_2Cl_2. The reaction was quenched by methanol and imidazole for conversion measurement. [b]Isolated yield; *dr* was determined by ^{1}H NMR analysis.

Iron(III) porphyrin triflate catalyst **1a** was freshly synthesized according to the following procedure.

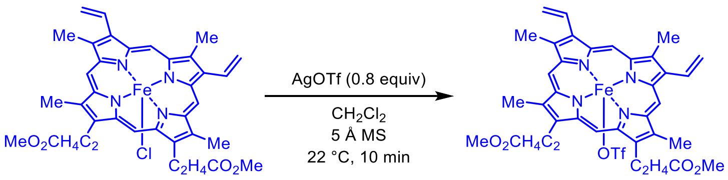

To a flame-dried sealable 2-dram vial equipped with a stir bar were added hemin dimethyl ester (**1c**)[1] (4.1 mg, 0.006 mmol, 6 mol %), AgOTf (1.3 mg, 0.005 mmol, 5 mol %), and freshly activated 5 Å molecular sieves, powder (*ca.* 20 mg). After the vial was evacuated and backfilled with N_2 three times, anhydrous CH_2Cl_2 (0.1 mL) was added and the solution was stirred at room temperature for 10 min. The resulting iron(III) porphyrin triflate catalyst **1a** solution was directly used in the iron-catalyzed stereospecific heterocycle *N*-glycosylation reactions with glycal epoxides.

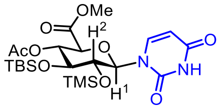

4

1-(Methyl 4-*O*-acetyl-3-*O*-*tert*-butyldimethylsilyl-2-*O*-trimethylsilyl-β-D-glucopyranosyluronate)uracil (**4**): IR ν_{max} (neat)/cm^{-1}: 2953 (w), 2360 (s), 1727 (m), 1697 (m), 1375 (s), 1250 (m), 1233 (m), 1115 (s), 831 (s); ^{1}H NMR (400 MHz, $CDCl_3$) δ 8.33 (brs, 1H), 7.50 (d, J = 8.2 Hz, 1H), 5.87 (dd, J = 8.2, 2.4 Hz, 1H), 5.73 (d, J = 8.8 Hz, 1H), 5.01 (dd, J =

9.1, 8.2 Hz, 1H), 4.09 (d, J = 9.1 Hz, 1H), 3.90 (t, J = 7.9 Hz, 1H), 3.75 – 3.67 (m, 1H), 3.71 (s, 3H), 2.10 (s, 3H), 0.87 (s, 9H), 0.14 (s, 3H), 0.11 (s, 3H), 0.06 (s, 9H); ^{13}C NMR (100 MHz, $CDCl_3$) δ 169.8, 167.6, 162.5, 150.7, 139.6, 103.9, 82.1, 75.6, 74.5, 73.9, 71.7, 52.8, 25.8 (3C), 21.0, 17.9, 0.5 (3C), -3.1, -4.2; HRMS: m/z (ESI) calcd for $C_{22}H_{39}N_2O_9Si_2^+$, [M + H]$^+$, 531.2189, found 531.2199. $^1J^{13}{}_{C1–H1}$ = 161.7 Hz.

The C1 stereochemistry was determined by measuring $^1J^{13}{}_{C1–H1}$ (161.7 Hz) through un-decoupled HSQC experiments. The C2 stereochemistry was determined by measuring $^3J_{H1–H2}$ (8.8 Hz). A $^1J^{13}{}_{C1–H1}$ value of 161.7 Hz suggested that the newly formed glycosidic bond is in equatorial position. The $^3J_{H1–H2}$ value of 8.8 Hz suggested that the H2 is in axial position and corroborated the exclusive formation of the α-epoxide in glycal epoxidation.

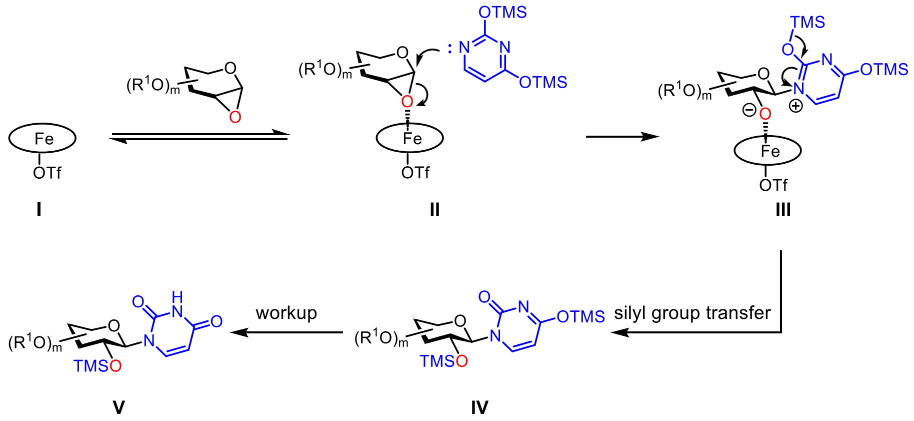

Figure S1b. Mechanistic Working Hypothesis for the Iron-Catalyzed Stereospecific Heterocycle *N*-Glycosylation.

First, the iron(III) porphyrin triflate catalyst **I** could coordinate to the glycal α-epoxide to form the iron–epoxide complex **II**. In the presence of a bis-silylated glycosyl acceptor, the presumable rate-determining glycosylation could proceed from **II** via an S_N2-type pathway to deliver intermediate **III**. Subsequent silyl group transfer could convert **III** into intermediate **IV**. Finally, exposure of **IV** to H_2O during workup affords the desired *N*-glycosylation product **V**.

C. General Procedures for the Iron-Catalyzed Stereospecific Pyrimidine *N*-Glycosylation with Glycal Epoxides

a. General Procedures for Glycal Epoxidation

Procedure A

To a 25 mL flask equipped with a stir bar at 0 °C were added a glycal (0.1 mmol, 1.0 equiv) in CH_2Cl_2/acetone mixture (v/v: 5:1, 1.7 mL) and saturated aqueous $NaHCO_3$ solution (2.8 mL), followed by addition of Oxone® ($KHSO_5 \cdot 0.5KHSO_4 \cdot 0.5K_2SO_4$) (215.5 mg, 0.35 mmol, 3.5 equiv) in H_2O (1.8 mL) dropwise. After stirring vigorously at 0 °C for 2 h, the reaction mixture was extracted with CH_2Cl_2 (3 mL × 3). The combined organic phase was dried over anhydrous Na_2SO_4 and concentrated *in vacuo*. The residue was further azeotropically dried with anhydrous toluene (1.0 mL × 3). The obtained glycal epoxide was assayed by ^{1}H NMR to get the diastereomeric ratio and directly used in the next step.

Procedure B

For glycals that are hydrophilic, an alternative epoxidation procedure has been applied.

To a 25 mL flask equipped with a stir bar at 0 °C were added a glycal (0.1 mmol, 1.0 equiv) in CH_2Cl_2, followed by the addition of freshly prepared dimethyldioxirane (DMDO in acetone, 0.06 M, 2.5 mL, 0.15 mmol, 1.5 equiv)[3] dropwise. After stirring at 0 °C for 2 h, the reaction mixture was concentrated *in vacuo*, then the residue was re-dissolved in anhydrous CH_2Cl_2 (2 mL) and dried over anhydrous Na_2SO_4. The organic phase concentrated *in vacuo*. The residue was further dried azeotropically with anhydrous toluene (1.0 mL × 3). The obtained glycal epoxide was assayed by ^{1}H NMR to get the diastereomeric ratio and directly used in the next step.

b. **General Procedures for the Iron-Catalyzed Stereospecific Pyrimidine *N*-Glycosylation**

Procedure C

To a flame-dried sealable 2-dram vial equipped with a stir bar were added a pyrimidine (0.15 mmol, 1.5 equiv) in anhydrous CH_3CN (0.5 mL), followed by the addition of BSTFA (0.30 mmol, 3.0 equiv). After stirring at room temperature for 30 min, the reaction mixture was concentrated *in vacuo*, and the obtained bis-silylated pyrimidine was used directly in the next step.

To a flame-dried sealable 2-dram vial equipped with a stir bar were added the bis-silylated pyrimidine (0.15 mmol, 1.5 equiv) and freshly activated 5 Å molecular sieves, powder (*ca.* 100 mg). After the vial was evacuated and backfilled with N_2 three times, iron (III) porphyrin triflate catalyst **1a** (0.005 mmol, 5 mol %) in anhydrous CH_2Cl_2 (0.1 mL) was added at −40 °C dropwise. After the mixture was stirred at −40 °C for 10 min, the corresponding glycal epoxide (0.1 mmol, 1.0 equiv) dissolved in anhydrous CH_2Cl_2 (0.4 mL) was added to the vial dropwise. The reaction mixture was raised 0 °C and kept at the same temperature until its completion (monitored by TLC). The reaction was quenched with imidazole (8.5 mg in 1 mL CH_2Cl_2) at the same temperature, then stirred at room temperature for 5 min. The mixture was filtered through a piece of cotton and eluted with EtOAc (2 mL × 2). The organic layer was then concentrated *in vacuo* and the *dr* was determined based on the ^{1}H NMR analysis of the crude reaction mixture. The residue was purified through a silica gel flash column to afford the desired *N*-glycosylation product.

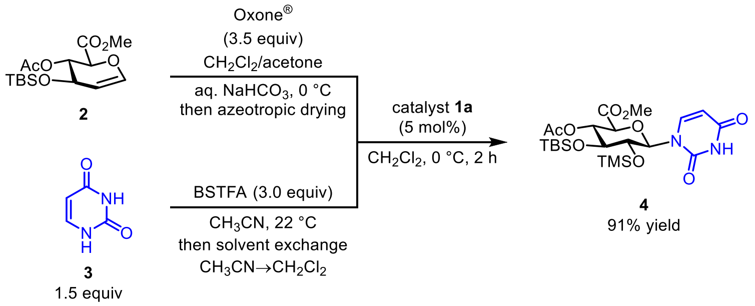

The reaction was carried out on a 1 mmol scale by following **Procedure A** for epoxidation and **Procedure C** for *N*-glycosylation. The *dr* of the corresponding glycal α-epoxide is >20:1. The *dr* of the glycosylation product determined based on crude reaction mixture is >20:1. The desired product **4** was purified through a silica gel flash column (hexanes/EtOAc: from 20:1 to 1:1) as colorless oil (483.1 mg, 91% yield).

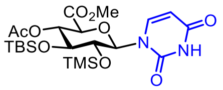

4

1-(Methyl 4-*O*-acetyl-3-*O*-*tert*-butyldimethylsilyl-2-*O*-trimethylsilyl-β-D-glucopyranosyluronate)uracil (**4**): IR ν_{max} (neat)/cm^{-1}: 2953 (w), 2360 (s), 1727 (m), 1697 (m), 1375 (s), 1250 (m), 1233 (m), 1115 (s), 831 (s); ^{1}H NMR (400 MHz, $CDCl_3$) δ 8.33 (brs, 1H), 7.50 (d, *J* = 8.2 Hz, 1H), 5.87 (dd, *J* = 8.2, 2.4 Hz, 1H), 5.73 (d, *J* = 8.8 Hz, 1H), 5.01 (dd, *J* = 9.1, 8.2 Hz, 1H), 4.09 (d, *J* = 9.1 Hz, 1H), 3.90 (t, *J* = 7.9 Hz, 1H), 3.75 – 3.67 (m, 1H), 3.71 (s, 3H), 2.10 (s, 3H), 0.87 (s, 9H), 0.14 (s, 3H), 0.11 (s, 3H), 0.06 (s, 9H); ^{13}C NMR (100 MHz, $CDCl_3$) δ 169.8, 167.6, 162.5, 150.7, 139.6, 103.9, 82.1, 75.6, 74.5, 73.9, 71.7, 52.8, 25.8 (3C), 21.0, 17.9, 0.5 (3C), -3.1, -4.2; HRMS: m/z (ESI) calcd for $C_{22}H_{39}N_2O_9Si_2^+$, $[M + H]^+$, 531.2189, found 531.2199. $^1J^{13}_{C1-H1}$ = 161.7 Hz.

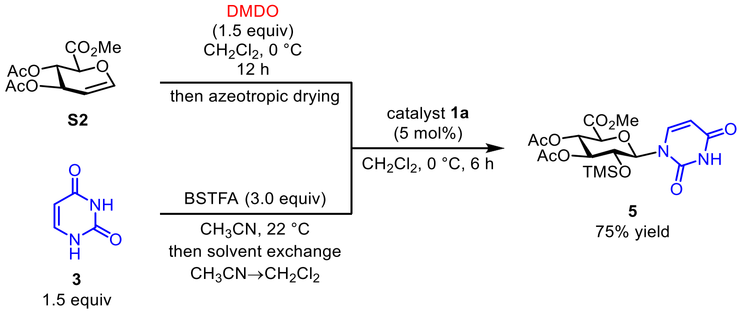

The reaction was carried out on a 0.2 mmol scale by following **Procedure B** for epoxidation and **Procedure C** for *N*-glycosylation with the modifications: the *N*-glycosylation was carried out at 0 °C for 6 h. The *dr* of the corresponding glycal α-epoxide is 4.8:1. The *dr* of the glycosylation product determined based on crude reaction mixture is >20:1. The desired product **5** was

purified through a silica gel flash column (hexanes/acetone: from 20:1 to 3:2) as colorless oil (68.7 mg, 75% yield).

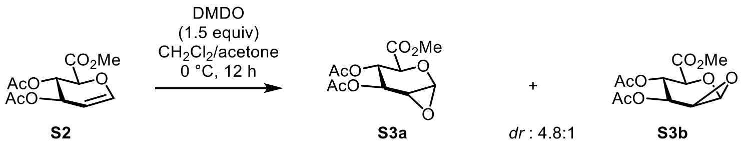

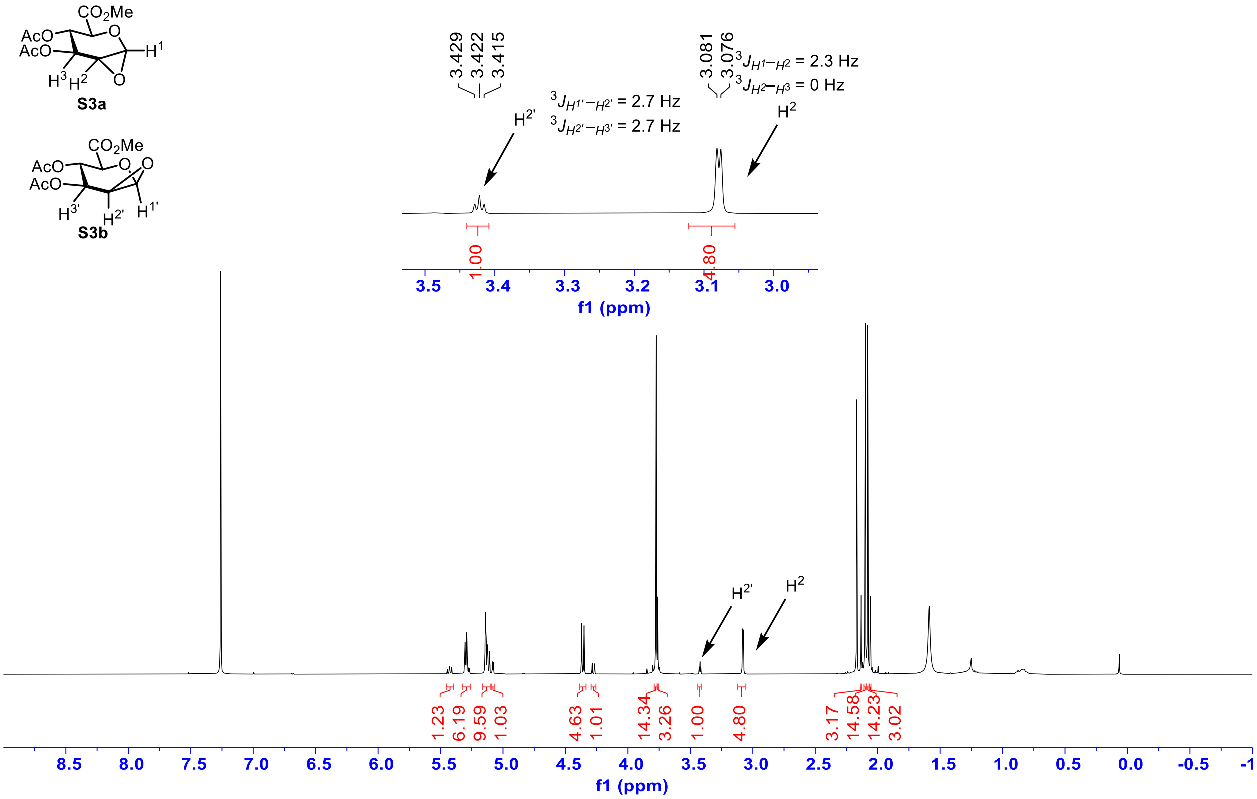

The stereochemistry of **S3a** and **S3b** was determined through ^{1}H NMR analysis. The C(2)–H chemical shift of **S3a** appears around 3.1 ppm, consistent with a glucal α-epoxide, while the corresponding chemical shift for **S3b** is around 3.4 ppm, which matches a glucal β-epoxide.[2]

The assignment is further corroborated by the $^{3}J_{\mathrm{H1–H2}}$ and $^{3}J_{\mathrm{H2–H3}}$ coupling constants: for **S3a** they are 2.3 Hz and 0 Hz, respectively, whereas **S3b** shows a comparable $^{3}J_{\mathrm{H1'–H2'}}$ and $^{3}J_{\mathrm{H2'–H3'}}$ value of 2.7 Hz.

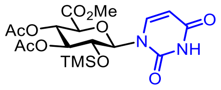

5

1-(Methyl 3,4-di-*O*-acetyl-2-*O*-trimethylsilyl-β-D-glucopyranosyluronate)uracil (**5**): IR ν_{max} (neat)/cm^{-1}: 2970 (w), 2359 (w), 1739 (s), 1694 (s), 1372 (m), 1217 (s), 1045 (m), 841 (s); ^{1}H NMR (400 MHz, $CDCl_3$) δ 8.61 (brs, 1H), 7.36 (d, *J* = 8.1 Hz, 1H), 5.86 (d, *J* = 8.1 Hz, 1H), 5.77 (d, *J* = 9.0 Hz, 1H), 5.30 (t, *J* = 9.2 Hz, 1H), 5.16 (t, *J* = 9.8 Hz, 1H), 4.23 (d, *J* = 9.9 Hz, 1H), 3.82 (t, *J* = 9.0 Hz, 1H), 3.74 (s, 3H), 2.07 (s, 3H), 2.04 (s, 3H), 0.01 (s, 9H); ^{13}C NMR (100 MHz, $CDCl_3$) δ 169.6, 169.4, 166.7, 162.0, 150.0, 139.0, 103.8, 82.6, 74.69, 74.66, 71.4, 69.2, 53.0, 20.8, 20.5, 0.1 (3C); HRMS: m/z (ESI) calcd for $C_{18}H_{27}N_2O_{10}Si^+$, $[M + H]^+$, 459.1429, found 459.1437. $^{1}J^{13}{}_{\mathrm{C1–H1}}$ = 161.0 Hz.

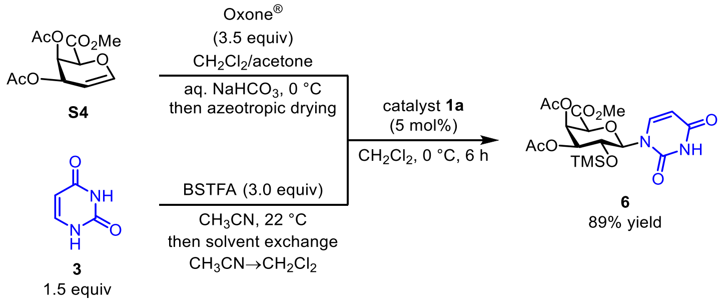

S4 was synthesized according to a modified literature procedure on a 1 mmol scale.[4] **S4** was obtained as colorless oil (194 mg, 75% yield).

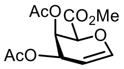

S4

Methyl 3,4-di-*O*-acetyl-2,6-anhydro-5-deoxy-D-arabino-hex-5-enonate (S4): IR ν_{max} (neat)/cm^{-1}: 2966 (w), 2360 (w), 1754 (s), 1731 (s), 1646 (m), 1218 (s), 841 (s); ^{1}H NMR (400 MHz, $CDCl_3$) δ 6.56 (dd, *J* = 6.3, 1.9 Hz, 1H), 5.73 – 5.67 (m, 1H), 5.65 – 5.89 (m, 1H), 4.77 (ddd, *J* = 6.3, 2.5, 1.7 Hz, 1H), 4.71 (s, 1H), 3.80 (s, 3H), 2.11 (s, 3H), 2.03 (s, 3H); ^{13}C NMR (100 MHz, $CDCl_3$) δ 170.2, 170.0, 167.2, 145.1, 99.2, 73.4, 64.4, 63.9, 52.8, 20.8, 20.6; HRMS: m/z (ESI) calcd for $C_{11}H_{15}O_7^+$, $[M + H]^+$, 259.0812, found 259.0818.

The reaction was carried out on a 0.1 mmol scale by following **Procedure A** for epoxidation and **Procedure C** for *N*-glycosylation with the modifications: the *N*-glycosylation was carried out at 0 °C for 6 h. The *dr* of the corresponding glycal α-epoxide is >20:1. The *dr* of the glycosylation product determined based on crude reaction mixture is >20:1. The desired product **6** was purified through a silica gel flash column (hexanes/acetone: from 20:1 to 1:1) as white foam (40.8 mg, 89% yield).

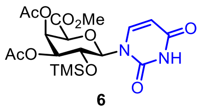

6

1-(Methyl 3,4-di-*O*-acetyl-2-*O*-trimethylsilyl-β-D-galactopyranosyluronate)uracil (6): IR ν_{max} (neat)/cm^{-1}: 2956 (w), 2359 (w), 1751 (m), 1692 (s), 1374 (m), 1230 (s), 1137 (s), 1083 (s), 841 (s); ^{1}H NMR (400 MHz, $CDCl_3$) δ 9.41 (brs, 1H), 7.40 (d, *J* = 8.1 Hz, 1H), 5.86 (d, *J* = 8.1 Hz, 1H), 5.84 – 5.75 (m, 2H), 5.08 (dd, *J* = 9.5, 3.3 Hz, 1H), 4.60 (d, *J* = 1.5 Hz, 1H), 4.01 – 3.84 (m, 1H), 3.73 (s, 3H), 2.11 (s, 3H), 2.02 (s, 3H), -0.01 (s, 9H); ^{13}C NMR (100 MHz, $CDCl_3$) δ 169.5, 169.4, 166.2, 162.5, 150.2, 139.1, 103.7, 82.4, 74.4, 73.3, 68.5, 68.4, 52.8, 20.6, 20.5, 0.0 (3C); HRMS: m/z (ESI) calcd for $C_{18}H_{27}N_2O_{10}Si^+$, $[M + H]^+$, 459.1429, found 459.1433. $^{1}J^{13}{}_{C1-H1}$ = 162.7 Hz.

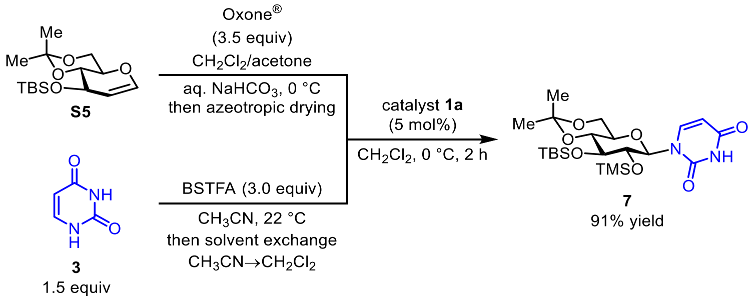

S5 was synthesized according to a literature procedure.[5]

The reaction was carried out on a 0.2 mmol scale by following **Procedure A** for epoxidation and **Procedure C** for *N*-glycosylation. The *dr* of the corresponding glycal α-epoxide is 16:1. The *dr* of the glycosylation product determined based on crude reaction mixture is >20:1. The desired product **7** was purified through a silica gel flash column (hexanes/acetone: from 20:1 to 3:1) as white foam (91.0 mg, 91% yield).

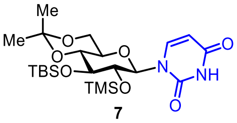

7

1-(3-*O*-*tert*-Butyldimethylsilyl-4,6-*O*-isopropylidene-2-*O*-trimethylsilyl-β-D-glucopyranosyl)uracil (**7**): IR ν_{max} (neat)/cm^{-1}: 2951 (w), 2359 (w), 1695 (s), 1458 (m), 1255 (m), 1102 (s), 1074 (m), 832 (s); ^{1}H NMR (400 MHz, $CDCl_3$) δ 8.64 (brs, 1H), 7.27 (d, *J* = 7.7 Hz, 1H), 5.82 (d, *J* = 8.1 Hz, 1H), 5.67 (brs, 1H), 3.89 (dd, *J* = 10.7, 5.0 Hz, 1H), 3.73 (t, *J* = 8.2 Hz, 1H), 3.66 (t, *J* = 10.3 Hz, 1H), 3.58 (brs, 1H), 3.50 – 3.36 (m, 2H), 1.47 (s, 3H), 1.41 (s, 3H), 0.90 (s, 9H), 0.12 (s, 3H), 0.09 (s, 3H), 0.04 (s, 9H); ^{13}C NMR (100 MHz, $CDCl_3$) δ 162.5, 150.5, 139.4, 103.4, 99.5, 83.1, 76.3, 75.0, 73.3, 69.9, 61.8, 28.8, 26.1 (3C), 18.7, 18.3, 0.5 (3C), -3.0, -3.9; HRMS: m/z (ESI) calcd for $C_{22}H_{41}N_2O_7Si_2^+$, [M + H]$^+$, 501.2447, found 501.2461. $^1J^{13}{}_{C1-H1}$ = 160.8 Hz.

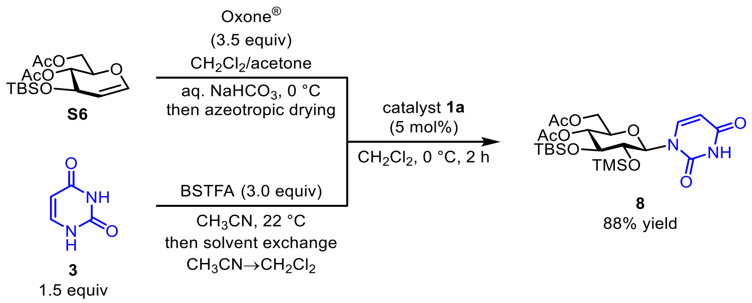

S6 was synthesized according to a literature procedure.[5]

The reaction was carried out on a 0.2 mmol scale by following **Procedure A** for epoxidation and **Procedure C** for *N*-glycosylation. The *dr* of the corresponding glycal α-epoxide is >20:1. The *dr* of the glycosylation product determined based on crude reaction mixture is >20:1. The desired product **8** was purified through a silica gel flash column (hexanes/EtOAc: from 20:1 to 3:2) as white foam (98.8 mg, 88% yield).

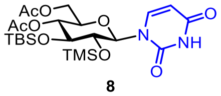

8

1-(4,6-di-*O*-acetyl-3-*O*-*tert*-butyldimethylsilyl-2-*O*-trimethylsilyl-β-D-glucopyranosyl)uracil (**8**): IR ν_{max} (neat)/cm^{-1}: 3226 (w), 2979 (w), 2359 (m), 1734 (s), 1375 (m), 1234 (s), 1115 (s), 813 (m); ^{1}H NMR (400 MHz, $CDCl_3$) δ 8.53 (brs, 1H), 7.31 (d, *J* = 8.2 Hz, 1H), 5.85 (dd, *J* = 8.2, 2.3 Hz, 1H), 5.66 (d, *J* = 8.6 Hz, 1H), 4.93 (dd, *J* = 9.9, 8.9 Hz, 1H), 4.13 (dd, *J* = 12.5, 5.5 Hz, 1H), 4.02 (dd, *J* = 12.5, 2.4 Hz, 1H), 3.83 (dd, *J* = 8.9, 7.9 Hz, 1H), 3.72 (ddd, *J* = 10.0, 5.4, 2.5 Hz, 1H), 3.65 (t, *J* = 8.3 Hz, 1H), 2.11 (s, 3H), 2.07 (s, 3H), 0.87 (s, 9H), 0.11 (s, 3H), 0.11 (s, 3H), 0.06 (s, 9H); ^{13}C NMR (100 MHz, $CDCl_3$) δ 170.6, 169.7, 162.5, 150.7, 139.7, 103.7, 82.9, 75.7, 74.7, 74.2, 70.1, 62.3, 25.9 (3C), 21.3, 20.8, 17.9, 0.6 (3C), -2.8, -4.1; HRMS: m/z (ESI) calcd for $C_{23}H_{41}N_2O_9Si_2^+$, $[M + H]^+$, 545.2345, found 545.2355. $^1J^{13}{}_{C1-H1}$ = 161.6 Hz.

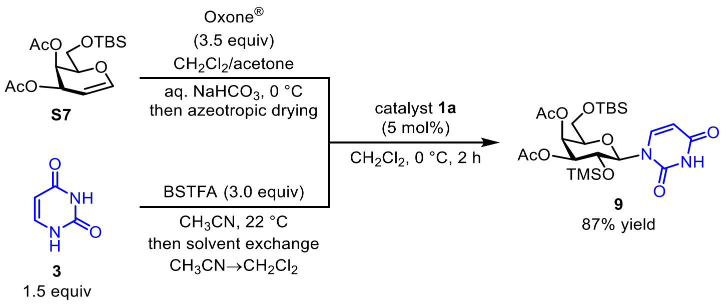

S7 was synthesized according to a literature procedure.[6]

The reaction was carried out on a 0.1 mmol scale by following **Procedure A** for epoxidation and **Procedure C** for *N*-glycosylation. The *dr* of the corresponding glycal α-epoxide is >20:1. The *dr* of the glycosylation product determined based on crude reaction mixture is >20:1. The desired product **9** was purified through a silica gel flash column (hexanes/EtOAc: from 20:1 to 3:1) as white foam (47.4 mg, 87% yield).

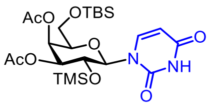

1-(3,4-Di-*O*-acetyl-6-*O*-*tert*-butyldimethylsilyl-2-*O*-trimethylsilyl-β-D-galactopyranosyl)uracil (**9**): IR ν_{max} (neat)/cm^{-1}: 2970 (w), 2360 (m), 1739 (s), 1366 (m), 1229 (s), 1217 (s), 1105 (m), 837 (m); ^{1}H NMR (400 MHz, $CDCl_3$) δ 9.13 (brs, 1H), 7.32 (d, *J* = 8.1 Hz, 1H), 5.84 (d, *J* = 8.1 Hz, 1H), 5.70 (d, *J* = 9.2 Hz, 1H), 5.53 (dd, *J* = 3.3, 1.2 Hz, 1H), 5.00 (dd, *J* = 9.6, 3.2 Hz, 1H), 3.96 – 3.77 (m, 2H), 3.67 (dd, *J* = 9.9, 5.9 Hz, 1H), 3.55 (dd, *J* = 9.9, 7.9 Hz, 1H), 2.14 (s, 3H), 2.02 (s, 3H), 0.83 (s, 9H), -0.00 (s, 12H), -0.01 (s, 3H); ^{13}C NMR (100 MHz, $CDCl_3$) δ 169.6, 169.5, 162.6, 150.2, 139.2, 103.3, 82.8, 76.0, 74.1, 69.1, 67.2, 60.3, 25.7 (3C), 20.7, 20.6, 18.1, 0.0 (3C), -5.6, -5.7; HRMS: m/z (ESI) calcd for $C_{23}H_{41}N_2O_9Si_2^+$, [M + H]$^+$, 545.2345, found 545.2358. $^{1}J^{13}_{C1-H1}$ = 162.9 Hz.

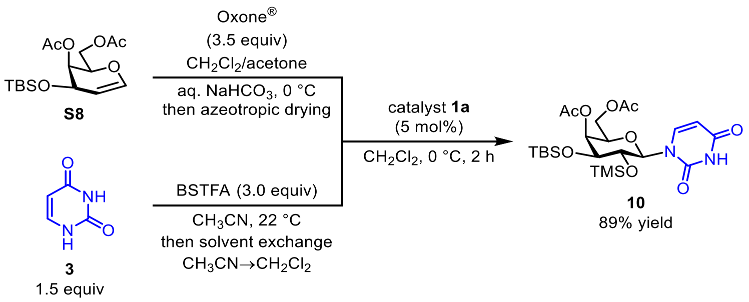

S8 was synthesized according to a literature procedure.[5]

The reaction was carried out on a 0.1 mmol scale by following **Procedure A** for epoxidation and **Procedure C** for *N*-glycosylation. The *dr* of the corresponding glycal α-epoxide is >20:1. The *dr* of the glycosylation product determined based on crude reaction mixture is >20:1. The desired product **10** was purified through a silica gel flash column (hexanes/EtOAc: from 20:1 to 3:2) as white foam (48.4 mg, 89% yield).

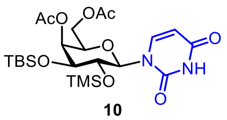

10

1-(4,6-Di-*O*-acetyl-3-*O*-*tert*-butyldimethylsilyl-2-*O*-trimethylsilyl-β-D-galactopyranosyl)uracil (**10**): IR ν_{max} (neat)/cm^{-1}: 2954 (w), 2360 (w), 1718 (s), 1678 (s), 1380 (m), 1228 (s), 1079 (s), 831 (s); ^{1}H NMR (400 MHz, $CDCl_3$) δ 8.44 (brs, 1H), 7.33 (d, *J* = 8.1 Hz, 1H), 5.87 (dd, *J* = 8.1, 2.3 Hz, 1H), 5.63 (brs, 1H), 5.28 (d, *J* = 1.7 Hz, 1H), 4.13 (q, *J* = 8.3 Hz, 1H), 4.05 – 3.93 (m, 2H), 3.82 – 3.75 (m, 2H), 2.14 (s, 3H), 2.05 (s, 3H), 0.87 (s, 9H), 0.15 (s, 3H), 0.10 (s, 3H), 0.03 (s, 9H); ^{13}C NMR (100 MHz, $CDCl_3$) δ 170.5, 169.8, 162.6, 150.7, 139.6, 103.6, 82.7, 73.9, 73.8, 71.5, 70.2, 62.3, 25.9 (3C), 20.73, 20.71, 17.7, 0.4 (3C), -4.36, -4.42; HRMS: m/z (ESI) calcd for $C_{23}H_{41}N_2O_9Si_2^+$, [M + H]$^+$, 545.2345, found 545.2358. $^{1}J^{13}_{C1-H1}$ = 162.0 Hz.

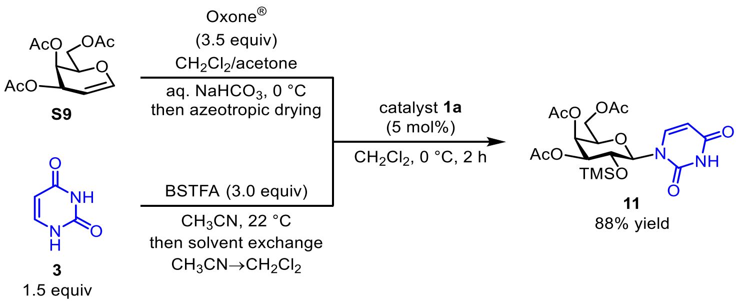

S9 was synthesized according to a literature procedure.[6]

The reaction was carried out on a 0.1 mmol scale by following **Procedure A** for epoxidation and **Procedure C** for *N*-glycosylation. The *dr* of the corresponding glycal α-epoxide is >20:1. The *dr* of the glycosylation product determined based on crude reaction mixture is >20:1. The desired product **11** was purified through a silica gel flash column (hexanes/acetone: from 20:1 to 3:2) as white foam (41.5 mg, 88% yield).

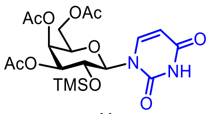

11

1-(3,4,6-Tri-*O*-acetyl-2-*O*-trimethylsilyl-β-D-galactopyranosyl)uracil (**11**): IR ν_{max} (neat)/cm^{-1}: 2959 (w), 1745 (s), 1691 (s), 1368 (m), 1250 (s), 1137 (m), 1080 (s), 840 (m); ^{1}H NMR (400 MHz, $CDCl_3$) δ 9.18 (brs, 1H), 7.32 (d, *J* = 8.1 Hz, 1H), 5.87 (dd, *J* = 8.1, 1.9 Hz, 1H), 5.79 – 5.70 (m, 1H), 5.46 (d, *J* = 3.2 Hz, 1H), 4.99 (dd, *J* = 9.6, 3.3 Hz, 1H), 4.18 – 4.01 (m, 3H), 3.88 (brs, 1H), 2.16 (s, 3H), 2.04 (s, 3H), 2.02 (s, 3H), 0.00 (s, 9H); ^{13}C NMR (100 MHz, $CDCl_3$) δ 170.4, 169.8, 169.6, 162.5, 150.3, 139.1, 103.5, 82.7, 73.8, 73.6, 68.9, 67.4, 61.4, 20.7, 20.62, 20.56, 0.0 (3C); HRMS: m/z (ESI) calcd for $C_{19}H_{29}N_2O_{10}Si^+$, $[M + H]^+$, 473.1586, found 473.1600. $^1J^{13}_{C1–H1}$ = 161.2 Hz.

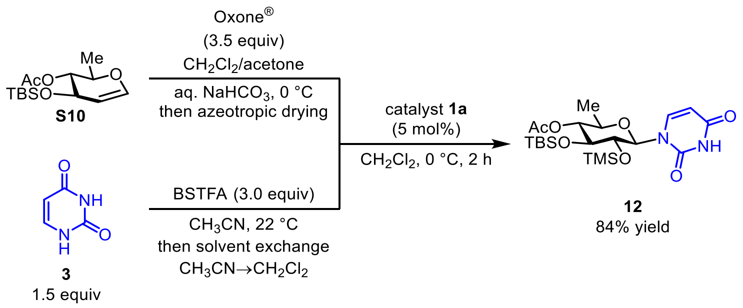

S10 was synthesized according to a literature procedure.[5]

The reaction was carried out on a 0.1 mmol scale by following **Procedure A** for epoxidation and **Procedure C** for *N*-glycosylation. The *dr* of the corresponding glycal α-epoxide is 15:1. The *dr* of the glycosylation product determined based on crude reaction mixture is >20:1. The desired product **12** was purified through a silica gel flash column (hexanes/EtOAc: from 20:1 to 3:2) as white foam (40.8 mg, 84% yield).

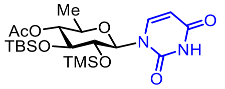

12

1-(4-*O*-Acetyl-3-*O*-*tert*-butyldimethylsilyl-6-deoxy-2-*O*-trimethylsilyl-β-D-glucopyranosyl)uracil (**12**): IR ν_{max} (neat)/cm^{-1}: 2929 (w), 2359 (w), 1730 (s), 1684 (s), 1459 (m), 1248 (s), 1062 (s), 832 (m); ^{1}H NMR (400 MHz, $CDCl_3$) δ 8.81 (brs, 1H), 7.30 (d, *J* = 8.1 Hz, 1H), 5.84 (dd, *J* = 8.2, 1.9 Hz, 1H), 5.62 (d, *J* = 8.8 Hz, 1H), 4.74 (t, *J* = 9.5 Hz, 1H), 3.79 (dd, *J* = 9.2, 8.1 Hz, 1H), 3.65 – 3.53 (m, 2H), 2.12 (s, 3H), 1.14 (d, *J* = 6.2 Hz, 3H), 0.86 (s, 9H), 0.10 (s, 6H), 0.04 (s, 9H); ^{13}C NMR (100 MHz, $CDCl_3$) δ 170.0, 162.7, 150.8, 139.8, 103.5, 82.7, 75.7, 74.9, 74.5, 73.0, 25.9 (3C), 21.5, 17.9, 17.6, 0.6 (3C), -2.7, -4.1; HRMS: m/z (ESI) calcd for $C_{21}H_{39}N_2O_7Si_2^+$, $[M + H]^+$, 487.2290, found 487.2292. $^{1}J^{13}{}_{C1–H1}$ = 159.5 Hz.

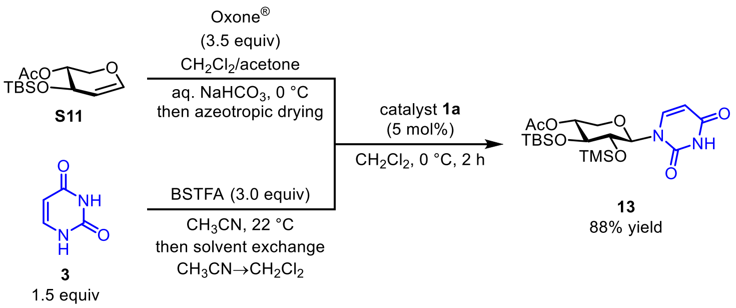

S11 was synthesized according to a literature procedure.[5]

The reaction was carried out on a 0.1 mmol scale by following **Procedure A** for epoxidation and **Procedure C** for *N*-glycosylation. The *dr* of the corresponding glycal α-epoxide is 11:1. The *dr* of the glycosylation product determined based on crude reaction mixture is >20:1. The desired product **13** was purified through a silica gel flash column (hexanes/EtOAc: from 20:1 to 3:2) as white foam (41.5 mg, 88% yield).

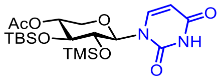

13

1-(4-*O*-Acetyl-3-*O*-*tert*-butyldimethylsilyl-2-*O*-trimethylsilyl-β-D-xylopyranosyl)uracil (**13**): IR ν_{max} (neat)/cm^{-1}: 2954 (w), 2358 (w), 1726 (m), 1699 (s), 1455 (m), 1244 (s), 1087 (s), 832 (m); ^{1}H NMR (400 MHz, $CDCl_3$) δ 9.12 (brs, 1H), 7.29 (d, *J* = 8.2 Hz, 1H), 5.83 (d, *J* = 8.1 Hz, 1H), 5.56 (d, *J* = 8.8 Hz, 1H), 4.74 (ddd, *J* = 10.2, 9.2, 5.4 Hz, 1H), 4.08 (dd, *J* = 11.3, 5.5 Hz, 1H), 3.84 (dd, *J* = 9.2, 8.0 Hz, 1H), 3.59 (t, *J* = 8.5 Hz, 1H), 3.29 (t, *J* = 10.8 Hz, 1H), 2.09 (s, 3H), 0.87 (s, 9H), 0.13 (s, 3H), 0.11 (s, 3H), 0.03 (s, 9H); ^{13}C NMR (100 MHz, $CDCl_3$) δ 170.1, 162.6, 150.7, 139.7, 103.5, 83.5, 75.7, 74.3, 71.6, 64.9, 25.9 (3C), 21.1, 18.1, 0.5 (3C), -2.8, -4.2; HRMS: m/z (ESI) calcd for $C_{20}H_{37}N_2O_7Si_2^+$, $[M + H]^+$, 473.2134, found 473.2143. $^{1}J^{13}_{C1–H1}$ = 161.8 Hz.

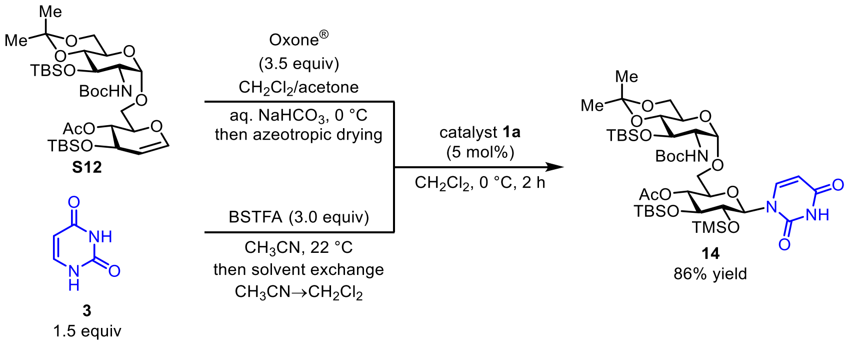

S12 was synthesized according to a literature procedure.[5]

The reaction was carried out on a 0.1 mmol scale by following **Procedure A** for epoxidation and **Procedure C** for *N*-glycosylation. The *dr* of the corresponding glycal α-epoxide is >20:1. The *dr* of the glycosylation product determined based on crude reaction mixture is >20:1. The desired product **14** was purified through a silica gel flash column (hexanes/EtOAc: from 20:1 to 2:1) as white foam (78.8 mg, 86% yield).

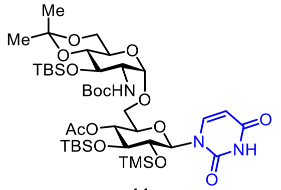

14

1-[(2-*tert*-Butoxycarbonylamino-3-*O*-*tert*-butyldimethylsilyl-2-deoxy-4,6-*O*-isopropylidene-α-D-glucopyranosyl)-(1 → 6)-(4-*O*-acetyl-3-*O*-*tert*-butyldimethylsilyl-2-*O*-trimethylsilyl-β-D-glucopyranosyl)]uracil (**14**): IR ν_{max} (neat)/cm^{-1}: 2930 (w), 2357 (w), 1695 (s), 1370 (m), 1250 (s), 1130 (m), 1079 (s), 833 (m); ^{1}H NMR (400 MHz, $CDCl_3$) δ 8.95 (brs, 1H), 7.22 (d, *J* = 8.2 Hz, 1H), 5.85 (d, *J* = 8.1 Hz, 1H), 5.65 (d, *J* = 8.6 Hz, 1H), 5.08 (d, *J* = 10.0 Hz, 1H), 4.95 (t, *J* = 9.5 Hz, 1H), 4.77 (d, *J* = 3.7 Hz, 1H), 3.81 (dd, *J* = 9.1, 7.8 Hz, 1H), 3.77 – 3.58 (m, 6H), 3.57 – 3.51 (m, 2H), 3.49 – 3.41 (m, 2H), 2.13 (s, 3H), 1.44 (s, 9H), 1.43 (s, 3H), 1.34 (s, 3H), 0.87 (s, 9H), 0.85 (s, 9H), 0.11 (s, 6H), 0.06 (s, 3H), 0.03 (s, 12H); ^{13}C NMR (100 MHz, $CDCl_3$) δ 169.2, 162.3, 155.5, 150.7, 139.3, 104.0, 99.3, 98.8, 83.1, 79.3, 76.5, 75.9, 74.7, 74.5, 70.8, 70.1, 64.7, 63.8, 62.3, 55.6, 29.0, 28.5 (3C), 25.9 (3C), 25.8 (3C), 21.5, 18.9, 18.2, 17.9, 0.7 (3C), -2.8, -4.1,

-4.2, -5.0; HRMS: m/z (ESI) calcd for $C_{41}H_{76}N_3O_{14}Si_3^+$, $[M + H]^+$, 918.4630, found 918.4655. $^1J^{13}{}_{C1–H1}$ = 170.8 Hz, 161.4 Hz.

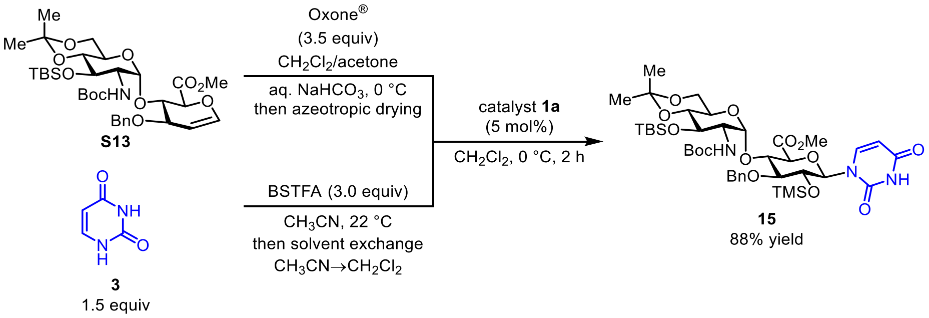

S13 was synthesized according to a literature procedure.[1]

The reaction was carried out on a 0.1 mmol scale by following **Procedure A** for epoxidation and **Procedure C** for *N*-glycosylation. The *dr* of the corresponding glycal α-epoxide is >20:1. The *dr* of the glycosylation product determined based on crude reaction mixture is >20:1. The desired product **15** was purified through a silica gel flash column (hexanes/EtOAc: from 20:1 to 2:1) as white foam (77.4 mg, 88% yield).

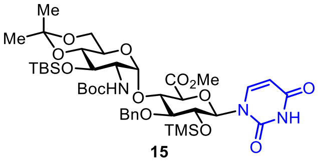

15

1-[(2-*tert*-Butoxycarbonylamino-3-*O*-*tert*-butyldimethylsilyl-2-deoxy-4,6-*O*-isopropylidene-α-D-glucopyranosyl)-(1→4)-(methyl 3-*O*-benzyl-2-*O*-trimethylsilyl-β-D-glucopyranosyluronate)]uracil (**15**): IR ν_{max} (neat)/cm^{-1}: 2928 (w), 1748 (w), 1696 (s), 1367 (m), 1253 (m), 1199 (s), 1075 (m), 836 (s), 594 (m); ^{1}H NMR (400 MHz, $CDCl_3$) δ 8.56 (brs, 1H), 7.40 (d, *J* = 8.2 Hz, 1H), 7.34 – 7.27 (m, 2H), 7.27 – 7.20 (m, 3H), 5.83 (d, *J* = 8.2 Hz, 1H), 5.75 (d, *J* = 8.3 Hz, 1H), 5.31 (d, *J* = 3.9 Hz, 1H), 4.83 (d, *J* = 10.9 Hz, 1H), 4.79 – 4.68 (m, 2H), 4.25 – 4.07 (m, 2H), 3.94 – 3.85 (m, 1H), 3.81 (s, 3H), 3.83 – 3.70 (m, 3H), 3.66 (t, *J* = 10.5 Hz, 1H), 3.56 – 3.43 (m, 2H), 3.28 – 3.16 (m, 1H), 1.44 (s, 3H), 1.36 (s, 3H), 1.30 (s, 9H), 0.85 (s,

9H), 0.04 (s, 3H), 0.03 (s, 3H), -0.00 (s, 9H); ^{13}C NMR (100 MHz, $CDCl_3$) δ 168.5, 162.1, 154.9, 150.3, 139.3, 136.9, 128.5 (2C), 127.9, 127.2 (2C), 103.6, 99.3, 99.0, 85.1, 82.4, 79.8, 76.6, 75.3, 74.4, 73.7, 73.5, 71.2, 64.5, 61.7, 55.2, 53.0, 29.1, 28.3 (3C), 25.7 (3C), 18.9, 18.2, 0.2 (3C), -4.1, -5.1; HRMS: m/z (ESI) calcd for $C_{41}H_{66}N_3O_{14}Si_2^+$, [M + H]$^+$, 880.4078, found 880.4095. $^1J^{13}{}_{C1–H1}$ = 161.7 Hz.

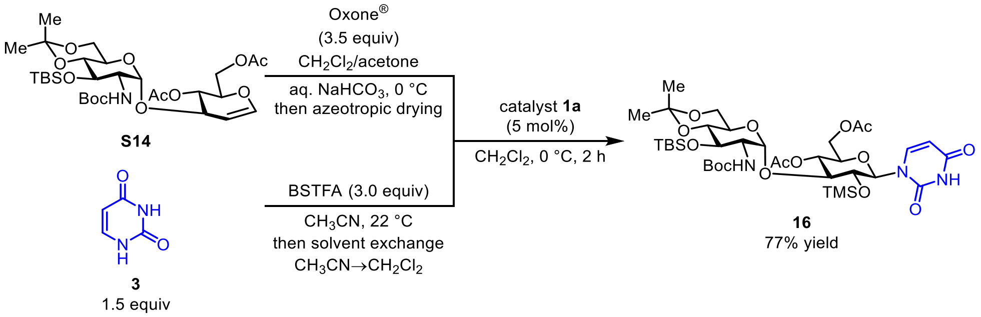

S14 was synthesized according to a literature procedure.[1]

The reaction was carried out on a 0.1 mmol scale by following **Procedure A** for epoxidation and **Procedure C** for *N*-glycosylation. The *dr* of the corresponding glycal α-epoxide is 10:1. The *dr* of the glycosylation product determined based on crude reaction mixture is >20:1. The desired product **16** was purified through a silica gel flash column (hexanes/EtOAc: from 20:1 to 3:2) as white foam (65.3 mg, 77% yield).

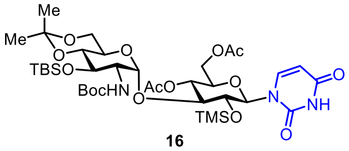

16

1-[(2-*tert*-Butoxycarbonylamino-3-*O*-*tert*-butyldimethylsilyl-2-deoxy-4,6-*O*-isopropylidene-α-D-glucopyranosyl)-(1→3)-(4,6-di-*O*-acetyl-2-*O*-trimethylsilyl-β-D-glucopyranosyl)]uracil (**16**): IR ν_{max} (neat)/cm^{-1}: 2928 (w), 1694 (s), 1635 (w), 1503 (m), 1368 (m), 1231 (s), 1077 (s), 836 (s), 777 (m); ^{1}H NMR (400 MHz, $CDCl_3$) δ 8.90 (brs, 1H), 7.24 (d, *J* = 8.2 Hz, 1H), 5.87 (d, *J* = 8.1 Hz, 1H), 5.66 (d, *J* = 8.7 Hz, 1H), 5.17 (d, *J* = 3.9 Hz, 1H), 5.11 (t, *J* = 9.8 Hz, 1H), 4.54

(d, J = 9.9 Hz, 1H), 4.13 (dd, J = 12.6, 5.3 Hz, 1H), 4.07 – 3.96 (m, 2H), 3.92 – 3.82 (m, 2H), 3.79 (dd, J = 10.0, 3.9 Hz, 1H), 3.76 – 3.70 (m, 2H), 3.69 – 3.58 (m, 2H), 3.50 (t, J = 9.0 Hz, 1H), 2.07 (s, 3H), 2.06 (s, 3H), 1.46 (s, 9H), 1.45 (s, 3H), 1.34 (s, 3H), 0.85 (s, 9H), 0.11 (s, 9H), 0.02 (s, 3H), 0.01 (s, 3H); ^{13}C NMR (100 MHz, $CDCl_3$) δ 170.5, 169.3, 162.2, 155.2, 150.5, 139.4, 103.9, 99.4, 98.4, 83.2, 79.8, 76.3, 74.8, 74.5, 72.3, 70.5, 70.3, 63.6, 62.2, 61.9, 55.3, 28.8, 28.4 (3C), 25.7 (3C), 20.8, 20.7, 18.9, 18.2, 0.6 (3C), -4.2, -5.0; HRMS: m/z (ESI) calcd for $C_{37}H_{64}N_3O_{15}Si_2^+$, $[M + H]^+$, 846.3870, found 846.3898. $^{1}J^{13}{}_{C1–H1}$ = 175.7 Hz, 162.2 Hz.

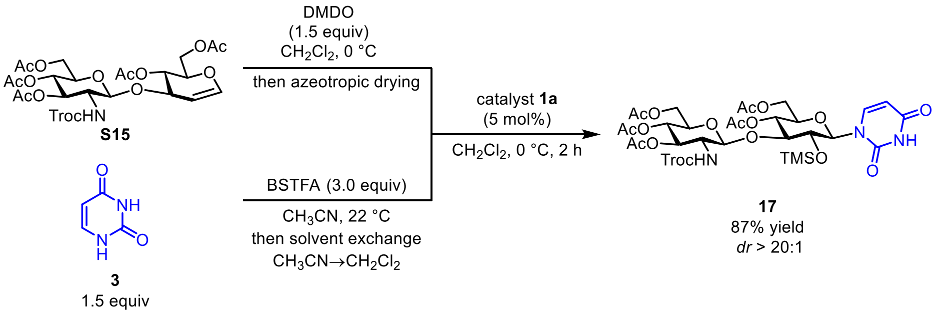

S15 was synthesized according to a literature procedure on a 3.0 mmol scale.[7] **S15** was obtained as white foam (1.74 g, 84% yield).

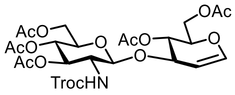

S15

4,6-Di-*O*-acetyl-3-*O*-(3,4,6-tri-*O*-acetyl-2-deoxy-2-(2,2,2-trichloroethoxycarbonylamino)-β-D-glucopyranosyl)-1,5-anhydro-2-deoxy-D-*arabino*-hex-1-enitol (**S15**): IR ν_{max} (neat)/cm^{-1}: 3277 (w), 2931 (w), 1735 (s), 1365 (m), 1226 (s), 1046 (s), 910 (w), 845 (m); ^{1}H NMR (400 MHz, $CDCl_3$) δ 6.41 (d, J = 6.3 Hz, 1H), 5.51 – 5.37 (m, 2H), 5.25 (s, 1H), 5.03 (t, J = 9.6 Hz, 1H), 4.96 (d, J = 8.3 Hz, 1H), 4.85 (dd, J = 6.3, 3.2 Hz, 1H), 4.80 (d, J = 12.0 Hz, 1H), 4.58 (d, J = 12.1 Hz, 1H), 4.41 – 4.39 (m, 2H), 4.25 (dd, J = 12.3, 4.7 Hz, 1H), 4.20 – 4.09 (m, 3H), 3.74 (d, J = 11.8 Hz, 1H), 3.43 (q, J = 8.9 Hz, 1H), 2.08 (s, 3H), 2.07 (s, 3H), 2.07 (s, 3H), 2.01 (s, 6H); ^{13}C NMR (100 MHz, $CDCl_3$) δ 170.83, 170.77, 170.5, 169.8, 169.6, 154.0, 145.3, 98.1, 97.6, 95.6, 74.6, 73.8, 72.0, 71.6, 69.8, 68.9, 67.7, 62.1, 61.8, 56.7, 21.0, 20.9, 20.81, 20.75, 20.7.

HRMS: m/z (ESI) calcd for $C_{25}H_{33}Cl_3NO_{15}^+$, $[M + H]^+$, 692.0910, found 692.0931. $^1J^{13}_{C1–H1}$ = 162.0 Hz.

The reaction was carried out on a 0.2 mmol scale by following **Procedure B** for epoxidation and **Procedure C** for *N*-glycosylation. The *dr* of the corresponding glycal α-epoxide is >20:1. The *dr* of the glycosylation product determined based on crude reaction mixture is >20:1. The desired product **17** was purified through a silica gel flash column (hexanes/acetone: from 20:1 to 1:1) as white foam (155.3 mg, 87% yield).

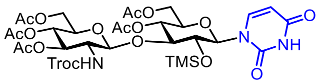

17

1-[(3,4,6-Tri-*O*-acetyl-2-deoxy-2-(2,2,2-trichloroethoxycarbonylamino)-β-D-glucopyranosyl)-(1→3)-(4,6-di-*O*-acetyl-2-*O*-trimethylsilyl-β-D-glucopyranosyl)]uracil (**17**): IR ν_{max} (neat)/cm^{-1}: 2957 (w), 1740 (s), 1692 (s), 1536 (w), 1368 (m), 1222 (s), 1030 (s), 842 (m), 732 (m); ^{1}H NMR (400 MHz, $CDCl_3$) δ 9.24 (brs, 1H), 7.29 (d, *J* = 8.2 Hz, 1H), 5.85 (d, *J* = 8.2 Hz, 1H), 5.63 (d, *J* = 8.9 Hz, 1H), 5.24 – 5.14 (m, 1H), 5.12 – 4.89 (m, 3H), 4.86 – 4.72 (m, 2H), 4.55 (d, *J* = 12.2 Hz, 1H), 4.38 (dd, *J* = 12.5, 4.5 Hz, 1H), 4.22 – 3.98 (m, 4H), 3.90 – 3.75 (m, 2H), 3.73 – 3.54 (m, 2H), 2.10 (s, 3H), 2.07 (s, 3H), 2.05 (s, 3H), 2.01 (s, 3H), 1.99 (s, 3H), 0.06 (s, 9H); ^{13}C NMR (100 MHz, $CDCl_3$) δ 170.7, 170.5, 170.4, 169.44, 169.35, 162.4, 153.7, 150.7, 139.3, 103.7, 99.6, 95.2, 82.5, 79.7, 74.8, 74.5, 73.4, 72.02, 71.96, 68.1, 67.2, 62.2, 61.6, 56.8, 20.72, 20.69, 20.67, 20.5 (two peaks overlapped, 2C), 0.5 (3C); HRMS: m/z (ESI) calcd for $C_{32}H_{45}Cl_3N_3O_{18}Si^+$, $[M + H]^+$, 892.1527, found 892.1550. $^1J^{13}_{C1–H1}$ = 160.7 Hz, 162.1 Hz.

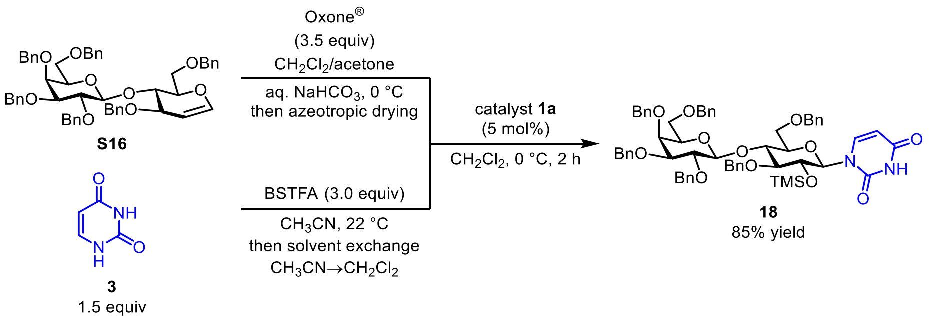

S16 was synthesized according to a literature procedure.[8]

The reaction was carried out on a 0.1 mmol scale by following **Procedure A** for epoxidation and **Procedure C** for *N*-glycosylation. The *dr* of the corresponding glycal α-epoxide is >20:1. The *dr* of the glycosylation product determined based on crude reaction mixture is >20:1. The desired product **18** was purified through a silica gel flash column (hexanes/EtOAc: from 20:1 to 3:2) as white foam (89.1 mg, 85% yield).

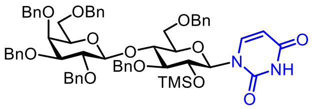

1-[(2,3,4,6-Tetra-*O*-benzyl-β-D-galactopyranosyl)-(1→4)-(3,6-di-*O*-benzyl-2-*O*-trimethylsilyl-β-D-glucopyranosyl)]uracil (**18**): IR ν_{max} (neat)/cm^{-1}: 3062 (w), 2867 (w), 1692 (s), 1452 (m), 1249 (m), 1069 (s), 839 (m), 731 (s), 694 (s); ^{1}H NMR (400 MHz, $CDCl_3$) δ 8.24 (brs, 1H), 7.36 – 7.27 (m, 15H), 7.27 – 7.19 (m, 13H), 7.19 – 7.11 (m, 3H), 5.77 (dd, *J* = 8.1, 2.0 Hz, 1H), 5.58 (d, *J* = 8.0 Hz, 1H), 5.19 (d, *J* = 10.8 Hz, 1H), 4.91 (d, *J* = 11.5 Hz, 1H), 4.82 (d, *J* = 11.2 Hz, 1H), 4.78 – 4.68 (m, 3H), 4.59 (d, *J* = 10.9 Hz, 1H), 4.50 (d, *J* = 8.1 Hz, 1H), 4.47 (d, *J* = 8.6 Hz, 1H), 4.40 (d, *J* = 7.7 Hz, 1H), 4.35 (d, *J* = 5.7 Hz, 1H), 4.32 (d, *J* = 5.5 Hz, 1H), 4.21 (d, *J* = 11.8 Hz, 1H), 4.02 (t, *J* = 9.3 Hz, 1H), 3.90 – 3.83 (m, 2H), 3.73 (dd, *J* = 9.7, 7.7 Hz, 1H), 3.59 – 3.45 (m, 4H), 3.41 – 3.26 (m, 4H), -0.05 (s, 9H); ^{13}C NMR (100 MHz, $CDCl_3$) δ 162.6, 150.5, 139.6, 139.0, 138.9, 138.6, 138.4, 138.2, 137.9, 128.3 (three peaks overlapped, 6C), 128.2 (2C), 128.1 (2C), 127.87, 127.85, 127.72 (two peaks overlapped, 4C), 127.68 (2C), 127.64, 127.58, 127.5 (three peaks overlapped, 6C), 127.4 (2C), 127.2, 126.8, 103.0, 102.7, 83.4, 82.9, 82.4, 79.8, 77.9, 75.7, 75.4, 75.0, 74.5, 73.53, 73.50, 73.29, 73.26, 73.1, 72.5, 68.1, 67.6, 0.2 (3C); HRMS: m/z (ESI) calcd for $C_{61}H_{69}N_2O_{12}Si^+$, $[M + H]^+$, 1049.4614, found 1049.4633. $^1J^{13}{}_{C1-H1}$ = 162.4 Hz, 160.7 Hz.

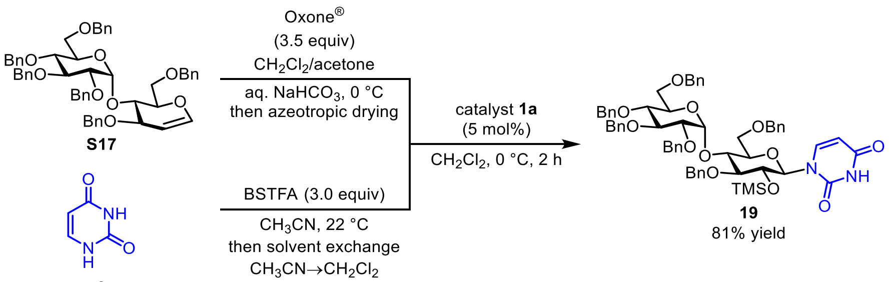

S17 was synthesized according to a modified literature procedure.[8]

The reaction was carried out on a 0.1 mmol scale by following **Procedure A** for epoxidation and **Procedure C** for *N*-glycosylation. The *dr* of the corresponding glycal α-epoxide is >20:1. The *dr* of the glycosylation product determined based on crude reaction mixture is >20:1. The desired product **19** was purified through a silica gel flash column (hexanes/EtOAc: from 20:1 to 3:2) as white foam (84.9 mg, 81% yield).

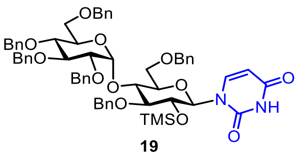

19

1-[(2,3,4,6-Tetra-*O*-benzyl-α-D-glucopyranosyl)-(1→4)-(3,6-di-*O*-benzyl-2-*O*-trimethylsilyl-β-D-glucopyranosyl)]uracil (**19**): IR ν_{max} (neat)/cm^{-1}: 3062 (w), 2867 (w), 1692 (s), 1452 (m), 1251 (m), 1069 (s), 840 (m), 732 (s), 694 (s); ^{1}H NMR (400 MHz, $CDCl_3$) δ 8.88 (brs, 1H), 7.46 – 7.15 (m, 31H), 5.89 (d, *J* = 8.2 Hz, 1H), 5.73 (d, *J* = 8.6 Hz, 1H), 5.63 (d, *J* = 3.6 Hz, 1H), 5.09 (d, *J* = 12.1 Hz, 1H), 4.93 – 4.85 (m, 3H), 4.82 (d, *J* = 10.8 Hz, 1H), 4.64 – 4.56 (m, 3H), 4.53 (d, *J* = 11.6 Hz, 2H), 4.48 – 4.40 (m, 2H), 4.18 (t, *J* = 9.1 Hz, 1H), 4.03 – 3.94 (m, 2H), 3.89 – 3.64 (m, 7H), 3.56 (dd, *J* = 10.7, 2.0 Hz, 1H), 3.52 (dd, *J* = 9.9, 3.6 Hz, 1H), 0.00 (s, 9H); ^{13}C NMR (100 MHz, $CDCl_3$) δ 162.4, 150.3, 139.5, 138.5, 138.33, 138.27, 138.0, 137.9, 137.8, 128.32 (two peaks overlapped, 4C), 128.29 (two peaks overlapped, 4C), 128.2 (2C), 128.1 (2C), 127.97 (2C), 127.82 (2C), 127.73 (2C), 127.69, 127.64, 127.60, 127.49, 127.46, 127.43 (2C), 127.39 (2C), 126.8, 125.7 (2C), 103.1, 97.4, 85.8, 82.8, 81.9, 79.1, 77.6, 77.5, 75.5, 75.0, 74.5, 74.1,

73.5, 73.4, 73.3, 72.9, 71.2, 68.6, 68.3, 0.1 (3C); HRMS: m/z (ESI) calcd for $C_{61}H_{69}N_2O_{12}Si^+$, $[M + H]^+$, 1049.4614, found 1049.4635. $^1J^{13}_{C1-H1}$ = 171.1 Hz, 161.0 Hz.

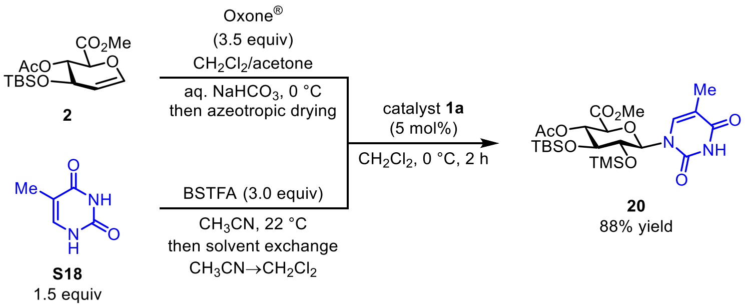

The reaction was carried out on a 0.2 mmol scale by following **Procedure A** for epoxidation and **Procedure C** for *N*-glycosylation. The *dr* of the corresponding glycal α-epoxide is >20:1. The *dr* of the glycosylation product determined based on crude reaction mixture is >20:1. The desired product **20** was purified through a silica gel flash column (hexanes/EtOAc: from 20:1 to 3:2) as white foam (95.8 mg, 88% yield).

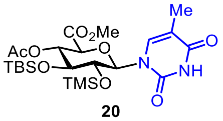

20

1-(Methyl 4-*O*-acetyl-3-*O*-*tert*-butyldimethylsilyl-2-*O*-trimethylsilyl-β-D-glucopyranosyluronate)thymine (**20**): IR ν_{max} (neat)/cm^{-1}: 2928 (w), 1710 (m), 1683 (s), 1450(w), 1239 (m), 1037 (m), 836 (s), 782 (m), 734 (m); ^{1}H NMR (400 MHz, $CDCl_3$) δ 9.31 (brs, 1H), 7.26 (s, 1H), 5.72 (d, *J* = 8.9 Hz, 1H), 5.00 (t, *J* = 8.9 Hz, 1H), 4.06 (d, *J* = 9.4 Hz, 1H), 3.88 (t, *J* = 8.1 Hz, 1H), 3.72 (t, *J* = 8.4 Hz, 1H), 3.69 (s, 3H), 2.08 (s, 3H), 1.97 (s, 3H), 0.85 (s, 9H), 0.11 (s, 3H), 0.09 (s, 3H), 0.01 (s, 9H); ^{13}C NMR (100 MHz, $CDCl_3$) δ 169.8, 167.6, 163.3, 150.9, 135.0, 112.4, 82.0, 75.6, 74.5, 73.7, 71.6, 52.8, 25.8 (3C), 21.0, 17.9, 12.5, 0.5 (3C), -3.0, -4.2; HRMS: m/z (ESI) calcd for $C_{23}H_{41}N_2O_9Si_2^+$, $[M + H]^+$, 545.2345, found 545.2351. $^1J^{13}_{C1-H1}$ = 162.2 Hz.

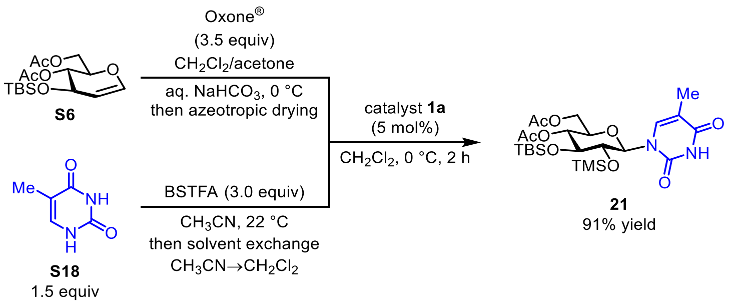

The reaction was carried out on a 0.2 mmol scale by following **Procedure A** for epoxidation and **Procedure C** for *N*-glycosylation. The *dr* of the corresponding glycal α-epoxide is >20:1. The *dr* of the glycosylation product determined based on crude reaction mixture is >20:1. The desired product **21** was purified through a silica gel flash column (hexanes/EtOAc: from 20:1 to 2:1) as white foam (102.1 mg, 91% yield).

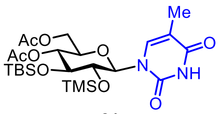

21

1-(4,6-Di-*O*-acetyl-3-*O*-*tert*-butyldimethylsilyl-2-*O*-trimethylsilyl-β-D-glucopyranosyl)thymine (**21**): IR ν_{max} (neat)/cm^{-1}: 3488 (w), 2929 (w), 2857 (w), 1742 (m), 1369 (w), 1233 (m), 1139 (m), 1034 (s), 916 (m), 853 (m), 837 (s), 779 (m); ^{1}H NMR (400 MHz, $CDCl_3$) δ 8.96 (brs, 1H), 7.11 (s, 1H), 5.65 (d, *J* = 8.7 Hz, 1H), 4.94 (t, *J* = 9.5 Hz, 1H), 4.12 (dd, *J* = 12.5, 5.5 Hz, 1H), 4.01 (dd, *J* = 12.5, 2.4 Hz, 1H), 3.82 (t, *J* = 8.6 Hz, 1H), 3.75 – 3.62 (m, 2H), 2.11 (s, 3H), 2.06 (s, 3H), 1.98 (d, *J* = 1.3 Hz, 3H), 0.86 (s, 9H), 0.101 (s, 3H), 0.095 (s, 3H), 0.02 (s, 9H); ^{13}C NMR (100 MHz, $CDCl_3$) δ 170.6, 169.8, 163.2, 150.9, 135.2, 112.1, 82.7, 75.8, 74.7, 74.1, 70.2, 62.4, 25.9 (3C), 21.4, 20.8, 17.9, 12.5, 0.6 (3C), -2.8, -4.1; HRMS: m/z (ESI) calcd for $C_{24}H_{43}N_2O_9Si_2^+$, [M + H]$^+$, 559.2502, found 559.2520. $^1J^{13}{}_{C1–H1}$ = 161.2 Hz.

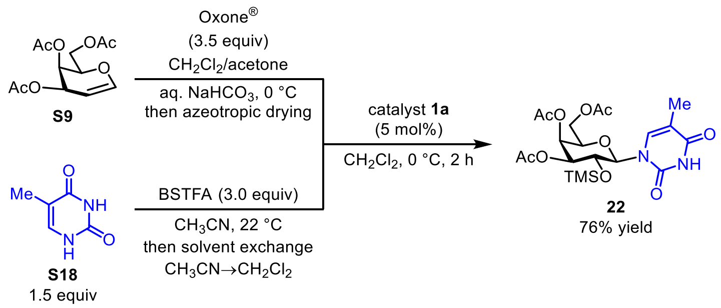

The reaction was carried out on a 0.2 mmol scale by following **Procedure A** for epoxidation and **Procedure C** for *N*-glycosylation. The *dr* of the corresponding glycal α-epoxide is >20:1. The *dr* of the glycosylation product determined based on crude reaction mixture is >20:1. The desired product **22** was purified through a silica gel flash column (hexanes/EtOAc: from 20:1 to 1:1) as white foam (73.9 mg, 76% yield).

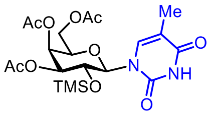

22

1-(3,4,6-Tri-*O*-acetyl-2-*O*-trimethylsilyl-β-D-galactopyranosyl)thymine (**22**): IR ν_{max} (neat)/cm^{-1}: 3490 (w), 2927 (w), 2861 (w), 1749 (m), 1373 (w), 1135 (m), 1055 (s), 837 (s); ^{1}H NMR (400 MHz, $CDCl_3$) δ 9.15 (d, *J* = 8.9 Hz, 1H), 7.07 (d, *J* = 1.5 Hz, 1H), 5.73 (brs, 1H), 5.45 (d, *J* = 3.3 Hz, 1H), 4.99 (dd, *J* = 9.5, 3.3 Hz, 1H), 4.18 – 4.01 (m, 3H), 3.87 (brs, 1H), 2.17 (s, 3H), 2.03 (s, 3H), 2.01 (s, 3H), 1.99 (s, 3H), -0.02 (s, 9H); ^{13}C NMR (100 MHz, $CDCl_3$) δ 170.4, 169.8, 169.5, 163.2, 150.5, 134.5, 111.9, 82.6, 73.8, 73.5, 68.6, 67.5, 61.4, 20.63, 20.60, 20.58, 12.5, 0.0 (3C); HRMS: m/z (ESI) calcd for $C_{20}H_{31}N_2O_{10}Si^+$, $[M + H]^+$, 487.1742, found 487.1747. $^{1}J^{13}{}_{C1–H1}$ = 159.2 Hz.

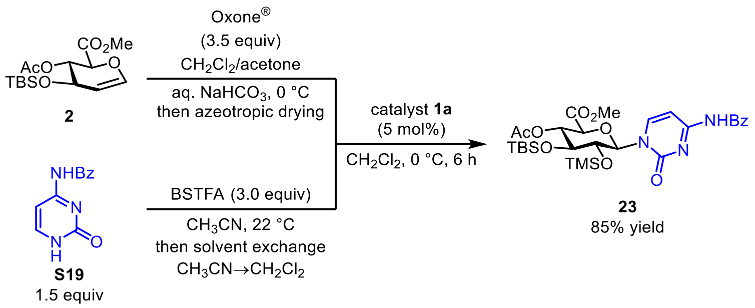

S19 was synthesized according to a literature procedure.[9]

The reaction was carried out on a 0.2 mmol scale by following **Procedure A** for epoxidation and **Procedure C** for *N*-glycosylation with the modifications: the *N*-glycosylation was carried out at 0 °C for 6 h. The *dr* of the corresponding glycal α-epoxide is >20:1. The *dr* of the glycosylation product determined based on crude reaction mixture is >20:1. The desired product **23** was purified through a silica gel flash column (hexanes/acetone: from 20:1 to 3:2) as white foam (107.6 mg, 85% yield).

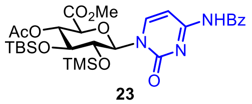

23

1-(Methyl 4-*O*-acetyl-3-*O*-*tert*-butyldimethylsilyl-2-*O*-trimethylsilyl-β-D-glucopyranosyluronate)4-*N*-benzoylcytosine (**23**): IR ν_{max} (neat)/cm^{-1}: 2950 (w), 1747 (w), 1668 (m), 1484 (m), 1237 (m), 1028 (m), 832 (s), 783 (m), 684 (m); ^{1}H NMR (400 MHz, $CDCl_3$) δ 8.74 (brs, 1H), 8.01 – 7.80 (m, 3H), 7.70 – 7.56 (m, 2H), 7.54 – 7.45 (m, 2H), 5.98 (d, *J* = 8.2 Hz, 1H), 5.05 (dd, *J* = 9.2, 7.9 Hz, 1H), 4.15 (d, *J* = 9.3 Hz, 1H), 3.94 (t, *J* = 7.6 Hz, 1H), 3.78 (t, *J* = 7.9 Hz, 1H), 3.70 (s, 3H), 2.10 (s, 3H), 0.85 (s, 9H), 0.12 (s, 3H), 0.09 (s, 3H), -0.00 (s, 9H); ^{13}C NMR (100 MHz, $CDCl_3$) δ 169.8, 167.7, 166.4, 162.2, 155.1, 144.7, 133.3, 132.9, 129.0 (2C), 127.7 (2C), 97.9, 83.3, 75.5, 74.6, 74.5, 71.8, 52.8, 25.8 (3C), 21.0, 17.9, 0.3 (3C), -3.1, -4.3; HRMS: m/z (ESI) calcd for $C_{29}H_{44}N_3O_9Si_2^+$, [M + H]$^+$, 634.2611, found 634.2628. $^1J^{13}{}_{C1-H1}$ = 162.8 Hz .

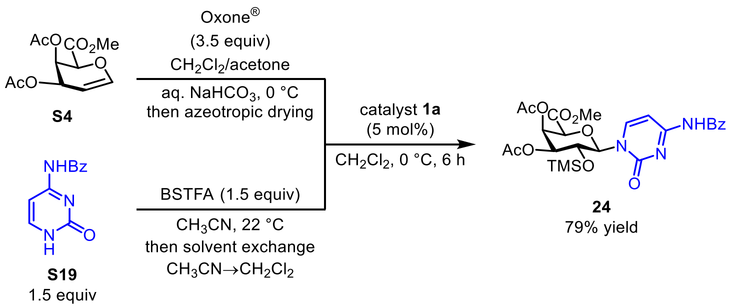

The reaction was carried out on a 0.1 mmol scale by following **Procedure A** for epoxidation and **Procedure C** for *N*-glycosylation with the modifications: the *N*-glycosylation was carried out at 0 °C for 6 h. The *dr* of the corresponding glycal α-epoxide is >20:1. The *dr* of the glycosylation product determined based on crude reaction mixture is >20:1. The desired product **24** was purified through a silica gel flash column (hexanes/acetone: from 20:1 to 1:1) as white foam (44.3 mg, 79% yield).

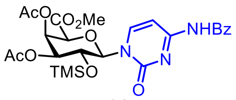

24

1-(Methyl 3,4-di-*O*-acetyl-2-*O*-trimethylsilyl-β-D-galactopyranosyluronate)4-*N*-benzoylcytosine (**24**): IR ν_{max} (neat)/cm^{-1}: 2935 (m), 2359 (w), 1670 (s), 1464 (s) , 1330 (m), 1235 (s), 1041 (s), 837 (s), 703 (m); ^{1}H NMR (400 MHz, $CDCl_3$) δ 8.71 (brs, 1H), 7.91 (d, *J* = 7.7 Hz, 2H), 7.84 (d, *J* = 7.5 Hz, 1H), 7.72 – 7.57 (m, 2H), 7.51 (t, *J* = 7.6 Hz, 2H), 6.03 (d, *J* = 8.9 Hz, 1H), 5.79 (dd, *J* = 3.3, 1.5 Hz, 1H), 5.08 (dd, *J* = 9.6, 3.4 Hz, 1H), 4.55 (d, *J* = 1.5 Hz, 1H), 3.97 (t, *J* = 8.5 Hz, 1H), 3.73 (s, 3H), 2.14 (s, 3H), 2.04 (s, 3H), -0.04 (s, 9H); ^{13}C NMR (100 MHz, $CDCl_3$) δ 169.44, 169.42, 165.9, 162.0, 154.1 (two peaks overlapped, 2C), 144.1, 133.3, 132.8, 129.0 (2C), 127.7 (2C), 97.9, 83.4, 74.7, 73.5, 69.4, 68.6, 52.7, 20.6, 20.5, -0.0 (3C); HRMS: m/z (ESI) calcd for $C_{25}H_{32}N_3O_{10}Si^+$, $[M + H]^+$, 562.1851, found 562.1864. $^1J^{13}{}_{C1\text{–}H1}$ = 162.1 Hz.

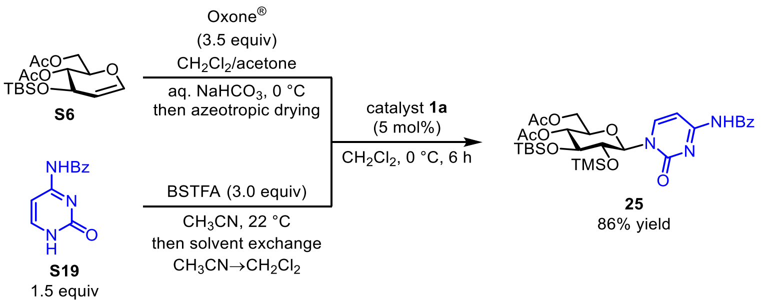

The reaction was carried out on a 0.2 mmol scale by following **Procedure A** for epoxidation and **Procedure C** for glycosylation with the modifications: heterocycle *N*-glycosylation was carried out at 0 °C for 6 h. The *dr* of the corresponding glycal α-epoxide is >20:1. The *dr* of the glycosylation product determined based on crude reaction mixture is >20:1. The desired product **25** was purified through a silica gel flash column (hexanes/acetone: from 20:1 to 2:1) as white foam (111.2 mg, 86% yield).

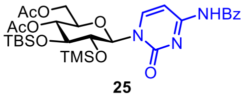

25

1-(4,6-Di-*O*-acetyl-3-*O*-*tert*-butyldimethylsilyl-2-*O*-trimethylsilyl-β-D-glucopyranosyl)4-*N*-benzoylcytosine (**25**): IR ν_{max} (neat)/cm^{-1}: 2928 (m), 1660 (s), 1482 (s) , 1311 (m), 1240 (s), 1041 (s), 834 (s), 780 (s), 703 (m); ^{1}H NMR (400 MHz, $CDCl_3$) δ 8.69 (brs, 1H), 7.89 (d, *J* = 7.7 Hz, 2H), 7.76 (d, *J* = 7.5 Hz, 1H), 7.68 – 7.53 (m, 2H), 7.53 – 7.46 (m, 2H), 5.91 (d, *J* = 8.0 Hz, 1H), 4.96 (dd, *J* = 9.9, 8.4 Hz, 1H), 4.13 (dd, *J* = 12.5, 5.6 Hz, 1H), 4.03 (dd, *J* = 12.4, 2.5 Hz, 1H), 3.86 (t, *J* = 8.1 Hz, 1H), 3.77 (ddd, *J* = 9.9, 5.6, 2.4 Hz, 1H), 3.71 (t, *J* = 7.9 Hz, 1H), 2.10 (s, 3H), 2.05 (s, 3H), 0.85 (s, 9H), 0.09 (s, 3H), 0.08 (s, 3H), -0.01 (s, 9H); ^{13}C NMR (100 MHz, $CDCl_3$) δ 170.6, 169.7, 166.4, 162.1, 155.0, 144.6, 133.2, 132.9, 129.0 (2C), 127.6 (2C), 97.8, 83.9, 75.6, 74.8, 74.7, 70.3, 62.5, 25.8 (3C), 21.3, 20.8, 17.9, 0.4 (3C), -2.9, -4.2; HRMS: m/z (ESI) calcd for $C_{30}H_{46}N_3O_9Si_2^+$, [M + H]$^+$, 648.2767, found 648.2782. $^1J^{13}_{C1-H1}$ = 162.1 Hz.

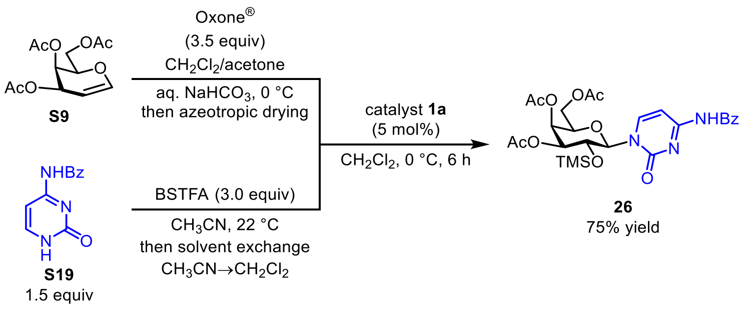

The reaction was carried out on a 0.2 mmol scale by following **Procedure A** for epoxidation and **Procedure C** for *N*-glycosylation with the modifications: the *N*-glycosylation was carried out at 0 °C for 6 h. The *dr* of the corresponding glycal α-epoxide is >20:1. The *dr* of the glycosylation product determined based on crude reaction mixture is >20:1. The desired product **26** was purified through a silica gel flash column (hexanes/acetone: from 20:1 to 3:2) as white foam (85.8 mg, 75% yield).

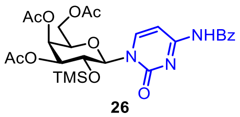

26

1-(3,4,6-Tri-*O*-acetyl-2-*O*-trimethylsilyl-β-D-galactopyranosyl)4-*N*-benzoylcytosine (**26**): IR ν_{max} (neat)/cm^{-1}: 2958 (w), 1667 (s), 1552 (m), 1481 (s), 1222 (s), 1079 (m), 841 (m), 782 (m), 690 (m); ^{1}H NMR (400 MHz, $CDCl_3$) δ 8.81 (brs, 1H), 7.91 (d, *J* = 7.7 Hz, 2H), 7.74 (d, *J* = 7.5 Hz, 1H), 7.65 – 7.53 (m, 2H), 7.53 – 7.44 (m, 2H), 5.99 (brs, 1H), 5.47 (d, *J* = 2.4 Hz, 1H), 5.02 (dd, *J* = 9.6, 3.3 Hz, 1H), 4.20 – 4.03 (m, 3H), 3.92 (brs, 1H), 2.17 (s, 3H), 2.021 (s, 3H), 2.018 (s, 3H), -0.05 (s, 9H); ^{13}C NMR (100 MHz, $CDCl_3$) δ 170.3, 169.8, 169.5, 166.5, 162.0, 154.4, 143.8, 133.3, 132.9, 128.9 (2C), 127.6 (2C), 97.8, 83.5, 73.79, 73.76, 69.9, 67.5, 61.4, 20.58, 20.56, 20.5, -0.1 (3C); HRMS: m/z (ESI) calcd for $C_{26}H_{34}N_3O_{10}Si^+$, $[M + H]^+$, 576.2008, found 576.2021. $^{1}J^{13}_{C1-H1}$ = 161.8 Hz.

D. General Procedures for the Iron-Catalyzed Stereospecific Heterocycle *N*-Glycosylation with Glycal Epoxides

General procedure D

To a flame-dried sealable 2-dram vial equipped with a stir bar were added an *N*-heterocycle (0.3 mmol, 1.5 equiv) and freshly activated 5 Å molecular sieves, powder (*ca.* 100 mg). After the vial was evacuated and backfilled with N_2 three times, iron (III) porphyrin triflate catalyst **1a** (0.010 mmol, 10 mol %) in anhydrous CH_2Cl_2 (0.2 mL) or in anhydrous CH_2Cl_2/1,4-dioxane (1:1, 0.2 mL) was added at −20 °C dropwise. After the mixture was stirred at −20 °C for 10 min, a glycal epoxide (0.2 mmol, 1.0 equiv) in anhydrous CH_2Cl_2 (0.3 mL) or anhydrous CH_2Cl_2/1,4-dioxane (1:1, 0.3 mL) was added to the vial dropwise. The reaction mixture was raised to 0 °C or 22 °C and kept at the same temperature until its completion (monitored by TLC). The reaction was quenched with MeOH (100 μL) and imidazole (17.0 mg in 2 mL CH_2Cl_2) at the same temperature. The mixture was filtered through a piece of cotton and eluted with EtOAc (4 mL × 2). The organic layer was then concentrated *in vacuo*. The *dr* was determined based on the ^{1}H NMR analysis of the crude reaction mixture. The residue was purified through a silica gel flash column to afford the desired glycosylation product.

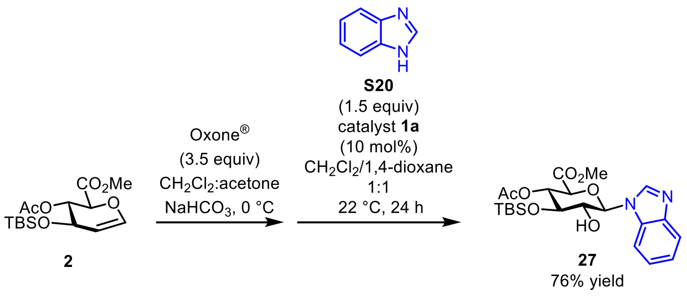

The reaction was carried out on a 0.2 mmol scale by following **Procedure A** for epoxidation and **Procedure D** for *N*-glycosylation. CH_2Cl_2/1,4-dioxane was used as the mixed solvent and the *N*-glycosylation was carried out at 22 °C for 24 h. The *dr* of the corresponding glycal α-epoxide is >20:1. The *dr* of the glycosylation product determined based on crude reaction mixture is >20:1.

The desired product **27** was purified through a silica gel flash column (hexanes/acetone: from 20:1 to 3:2) as white foam (70.5 mg, 76% yield).

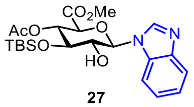

27

1-(Methyl 4-*O*-acetyl-3-*O*-*tert*-butyldimethylsilyl-β-D-glucopyranosyluronate)benzimidazole (**27**): IR ν_{max} (neat)/cm^{-1}: 3125 (w), 2928 (w), 1751 (s), 1738 (s), 1372 (m), 1224 (s), 1033 (m), 838 (s), 779 (s); ^{1}H NMR (400 MHz, $CDCl_3$) δ 7.61 (d, *J* = 8.0 Hz, 1H), 7.51 (s, 1H), 7.24 – 7.17 (m, 2H), 7.03 (t, *J* = 7.6 Hz, 1H), 7.00 (brs, 1H), 5.32 – 5.23 (m, 2H), 4.15 (d, *J* = 10.0 Hz, 1H), 4.10 (t, *J* = 8.6 Hz, 1H), 3.99 (t, *J* = 8.8 Hz, 1H), 3.72 (s, 3H), 2.14 (s, 3H), 0.91 (s, 9H), 0.21 (s, 3H), 0.19 (s, 3H); ^{13}C NMR (100 MHz, $CDCl_3$) δ 169.8, 167.4, 142.4, 140.8, 131.6, 123.7, 123.3, 119.1, 112.0, 87.0, 75.4, 75.2, 72.5, 71.7, 52.9, 25.7 (3C), 20.9, 18.2, -3.9, -4.9; HRMS: m/z (ESI) calcd for $C_{22}H_{33}N_2O_7Si^+$, [M + H]$^+$, 465.2052, found 465.2057. $^{1}J^{13}{}_{C1-H1}$ = 157.3 Hz.

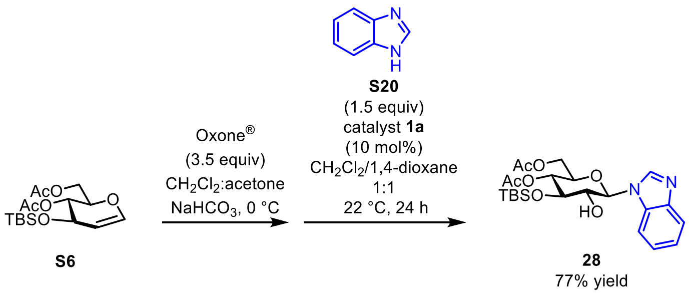

The reaction was carried out on a 0.2 mmol scale by following **Procedure A** for epoxidation and **Procedure D** for *N*-glycosylation. CH_2Cl_2/1,4-dioxane was used as the mixed solvent and the *N*-glycosylation was carried out at 22 °C for 24 h. The *dr* of the corresponding glycal α-epoxide is >20:1. The *dr* of the glycosylation product determined based on crude reaction mixture is >20:1. The desired product **28** was purified through a silica gel flash column (hexanes/acetone: from 20:1 to 3:2) as white foam (73.6 mg, 77% yield).

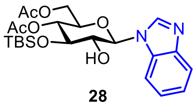

28

1-(4,6-Di-*O*-acetyl-3-*O*-*tert*-butyldimethylsilyl-β-D-glucopyranosyl)benzimidazole (**28**): IR ν_{max} (neat)/cm^{-1}: 3101 (w), 2928 (w), 1741 (s), 1458 (m), 1370 (m), 1224 (s), 1037 (s), 837 (s), 738 (s); ^{1}H NMR (400 MHz, $CDCl_3$) δ 7.54 (s, 1H), 7.52 (d, J = 8.3 Hz, 1H), 7.20 – 7.12 (m, 2H), 7.10 (d, J = 8.1 Hz, 1H), 6.97 (t, J = 7.1 Hz, 1H), 5.27 (d, J = 8.4 Hz, 1H), 5.29 – 5.22 (m, 1H), 4.22 (dd, J = 12.5, 5.0 Hz, 1H), 4.14 (dd, J = 12.4, 2.4 Hz, 1H), 4.07 (t, J = 8.6 Hz, 1H), 3.94 (t, J = 8.9 Hz, 1H), 3.82 (ddd, J = 10.2, 5.0, 2.4 Hz, 1H), 2.15 (s, 3H), 2.06 (s, 3H), 0.90 (s, 9H), 0.21 (s, 3H), 0.18 (s, 3H); ^{13}C NMR (100 MHz, $CDCl_3$) δ 170.7, 169.7, 142.4, 141.0, 131.5, 123.4, 123.1, 119.1, 112.0, 87.6, 75.9, 74.7, 72.8, 70.4, 62.4, 25.7 (3C), 21.2, 20.7, 18.2, -3.8, -5.0; HRMS: m/z (ESI) calcd for $C_{23}H_{35}N_2O_7Si^+$, [M + H]$^+$, 479.2208, found 479.2230. $^1J^{13}_{C1–H1}$ = 154.7 Hz.

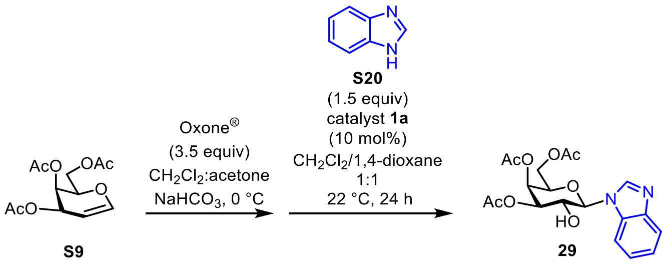

The reaction was carried out on a 0.2 mmol scale by following **Procedure A** for epoxidation and **Procedure D** for *N*-glycosylation. CH_2Cl_2/1,4-dioxane was used as the mixed solvent and the *N*-glycosylation was carried out at 22 °C for 24 h. The *dr* of the corresponding glycal α-epoxide is >20:1. The *dr* of the glycosylation product determined based on crude reaction mixture is >20:1. The desired product **29** was purified through a silica gel flash column (hexanes/acetone: from 20:1 to 1:1) as white foam (64.2 mg, 79% yield).

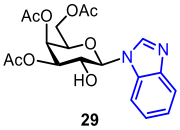

29

1-(3,4,6-Tri-*O*-acetyl-β-D-galactopyranosyl)benzimidazole (**29**): IR ν_{max} (neat)/cm^{-1}: 3456 (w), 2970 (w), 1739 (s), 1435 (w), 1365 (m), 1228 (s), 1091 (w), 924 (w), 744 (w); ^{1}H NMR (400 MHz, acetone-d_6) δ 8.21 (s, 1H), 7.77 (dd, *J* = 7.6, 1.4 Hz, 1H), 7.65 (d, *J* = 7.9 Hz, 1H), 7.34 – 7.28 (m, 1H), 7.25 (td, *J* = 7.6, 1.3 Hz, 1H), 5.81 (d, *J* = 9.0 Hz, 1H), 5.54 (dd, *J* = 3.4, 1.1 Hz, 1H), 5.24 (brs, 1H), 5.22 (dd, *J* = 9.9, 3.3 Hz, 1H), 4.63 – 4.48 (m, 2H), 4.23 (dd, *J* = 11.4, 5.7 Hz, 1H), 4.12 (dd, *J* = 11.4, 6.9 Hz, 1H), 2.25 (s, 3H), 1.99 (s, 3H), 1.96 (s, 3H); ^{13}C NMR (100 MHz, acetone-d_6) δ 170.8, 170.6, 170.5, 145.4, 143.3, 134.0, 123.7, 123.1, 120.9, 112.8, 86.9, 74.5, 73.9, 68.7, 67.5, 62.6, 20.71, 20.68, 20.6; HRMS: m/z (ESI) calcd for $C_{19}H_{23}N_2O_8^+$, [M + H]$^+$, 407.1449, found 407.1455. $^{1}J^{13}{}_{C1-H1}$ = 155.4 Hz.

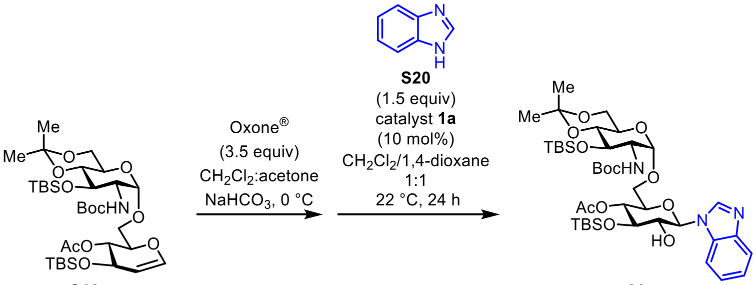

S12 **30** 83% yield *dr* > 20:1

The reaction was carried out on a 0.1 mmol scale by following **Procedure A** for epoxidation and **Procedure D** for *N*-glycosylation. CH_2Cl_2/1,4-dioxane was used as the mixed solvent and the *N*-glycosylation was carried out at 22 °C for 24 h. The *dr* of the corresponding glycal α-epoxide is >20:1. The *dr* of the glycosylation product determined based on crude reaction mixture is >20:1. The desired product **30** was purified through a silica gel flash column (hexanes/acetone: from 20:1 to 3:2) as white foam (70.5 mg, 83% yield).

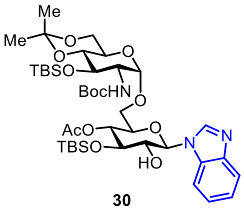

30

1-[(2-*tert*-Butoxycarbonylamino-3-*O*-*tert*-butyldimethylsilyl-2-deoxy-4,6-*O*-isopropylidene-α-D-glucopyranosyl)-(1→6)-(4-*O*-acetyl-3-*O*-*tert*-butyldimethylsilyl-β-D-glucopyranosyl)]benzimidazole (**30**): IR ν_{max} (neat)/cm^{-1}: 3460 (w), 2970 (w), 1739 (s), 1436 (w), 1366 (m), 1229 (m), 1081 (w), 838 (w), 778 (w); ^{1}H NMR (400 MHz, $CDCl_3$) δ 7.53 (s, 1H), 7.33 (d, *J* = 8.2 Hz, 1H), 7.30 – 7.19 (m, 2H), 6.92 – 6.79 (m, 2H), 5.51 (d, *J* = 10.3 Hz, 1H), 5.37 (t, *J* = 9.6 Hz, 1H), 5.17 (d, *J* = 8.5 Hz, 1H), 4.93 (d, *J* = 3.7 Hz, 1H), 4.11 (t, *J* = 8.6 Hz, 1H), 3.92 (t, *J* = 8.9 Hz, 1H), 3.88 – 3.74 (m, 2H), 3.70 (dd, *J* = 10.4, 3.7 Hz, 1H), 3.67 – 3.60 (m, 2H), 3.60 – 3.53 (m, 2H), 3.52 – 3.38 (m, 2H), 2.18 (s, 3H), 1.50 (s, 9H), 1.38 (s, 3H), 1.30 (s, 3H), 0.91 (s, 9H), 0.87 (s, 9H), 0.20 (s, 3H), 0.19 (s, 3H), 0.08 (s, 3H), 0.04 (s, 3H); ^{13}C NMR (100 MHz, $CDCl_3$) δ 169.2, 155.7, 142.5, 141.3, 130.6, 124.4, 123.1, 118.8, 111.9, 99.2, 98.8, 88.7, 79.1, 77.2, 76.2, 74.7, 72.5, 71.0, 70.2, 63.8, 63.7, 62.3, 55.5, 29.0, 28.7 (3C), 25.8 (two peaks overlapped, 6C), 21.4, 18.9, 18.2 (two peaks overlapped, 2C), -3.9, -4.3, -4.85, -4.94; HRMS: m/z (ESI) calcd for $C_{41}H_{70}N_3O_{12}Si_2^+$, [M + H]$^+$, 852.4493, found 852.4518. $^1J^{13}_{C1-H1}$ = 170.8 Hz, 155.0 Hz.

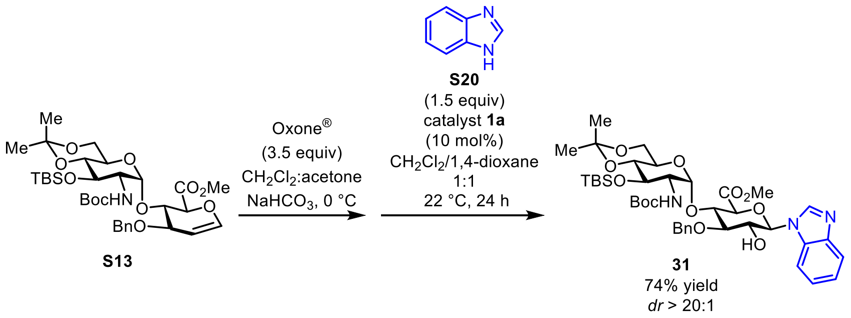

The reaction was carried out on a 0.1 mmol scale by following **Procedure A** for epoxidation and **Procedure D** for *N*-glycosylation. CH_2Cl_2/1,4-dioxane was used as the mixed solvent and the *N*-glycosylation was carried out at 22 °C for 24 h. The *dr* of the corresponding glycal α-epoxide is >20:1. The *dr* of the glycosylation product determined based on crude reaction mixture is >20:1. The desired product **31** was purified through a silica gel flash column (hexanes/acetone: from 20:1 to 1:1) as white foam (60.2 mg, 74% yield).

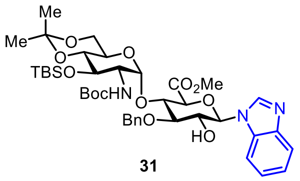

31

1-[(2-*tert*-Butoxycarbonylamino-3-*O*-*tert*-butyldimethylsilyl-2-deoxy-4,6-*O*-isopropylidene-α-D-glucopyranosyl)-(1→4)-(methyl 3-*O*-benzyl-β-D-glucopyranosyluronate)]benzimidazole (**31**): IR ν_{max} (neat)/cm^{-1}: 3456 (w), 2970 (w), 1738 (s), 1456 (w), 1366 (m), 1228 (m), 1077 (m), 836 (w), 738 (w); ^{1}H NMR (400 MHz, $CDCl_3$) δ 7.55 – 7.44 (m, 2H), 7.39 (d, *J* = 6.7 Hz, 2H), 7.34 – 7.22 (m, 3H), 7.18 (t, *J* = 7.7 Hz, 1H), 7.11 (d, *J* = 8.0 Hz, 1H), 6.97 (t, *J* = 7.7 Hz, 1H), 6.90 (brs, 1H), 5.50 (d, *J* = 3.8 Hz, 1H), 5.29 (d, *J* = 8.8 Hz, 1H), 5.13 (d, *J* = 10.3 Hz, 1H), 5.04 (d, *J* = 10.1 Hz, 1H), 4.86 (d, *J* = 10.2 Hz, 1H), 4.35 – 4.21 (m, 2H), 4.18 (d, *J* = 9.6 Hz, 1H), 4.01 – 3.81 (m, 3H), 3.81 (s, 3H), 3.69 (t, *J* = 10.5 Hz, 1H), 3.59 (t, *J* = 9.2 Hz, 1H), 3.52 (t, *J* = 9.1 Hz, 1H), 3.30 (td, *J* = 10.0, 5.2 Hz, 1H), 1.47 (s, 3H), 1.38 (s, 9H), 1.37 (s, 3H), 0.90 (s, 9H), 0.11 (s, 3H), 0.09 (s, 3H); ^{13}C NMR (100 MHz, $CDCl_3$) δ 168.1, 155.1, 142.4, 140.8, 137.5, 131.6, 128.59 (2C), 128.56 (2C), 128.1, 123.8, 123.3, 119.2, 111.6, 99.4, 99.3, 87.0, 84.4, 79.7, 77.0, 75.4, 74.5, 74.0, 72.9, 71.4, 64.4, 61.8, 55.3, 52.9, 29.1, 28.5 (3C), 25.8 (3C), 18.9, 18.3, -4.1, -5.0; HRMS: m/z (ESI) calcd for $C_{41}H_{60}N_3O_{12}Si^+$, $[M + H]^+$, 814.3941, found 814.3957. $^{1}J^{13}{}_{C1-H1}$ = 155.5 Hz, 175.2 Hz.

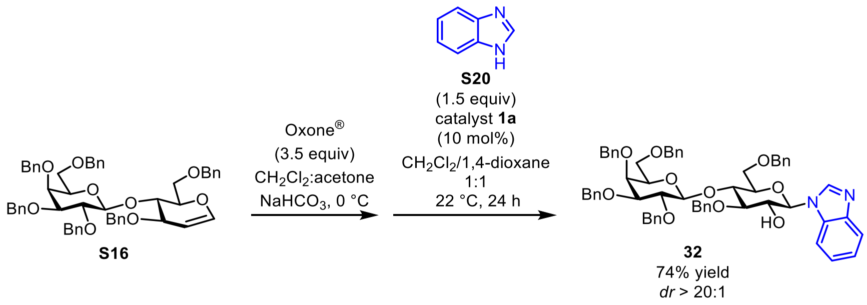

The reaction was carried out on a 0.1 mmol scale by following **Procedure A** for epoxidation and **Procedure D** for *N*-glycosylation. CH_2Cl_2/1,4-dioxane was used as the mixed solvent and the *N*-glycosylation was carried out at 22 °C for 24 h. The *dr* of the corresponding glycal α-epoxide is >20:1. The *dr* of the glycosylation product determined based on crude reaction mixture is >20:1.

The desired product **32** was purified through a silica gel flash column (hexanes/acetone: from 20:1 to 1:1) as white foam (72.7 mg, 74% yield).

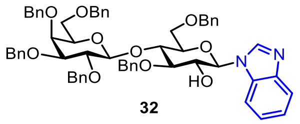

32

1-[(2,3,4,6-Tetra-*O*-benzyl-β-D-galactopyranosyl)-(1→4)-(3,6-di-*O*-benzyl-β-D-glucopyranosyl)]benzimidazole (**32**): IR ν_{max} (neat)/cm^{-1}: 3456 (w), 2970 (w), 1738 (s), 1454 (m), 1365 (m), 1227 (m), 1071 (s), 736 (s), 695 (s); ^{1}H NMR (400 MHz, $CDCl_3$) δ 7.68 (s, 1H), 7.48 – 6.93 (m, 34H), 5.20 (d, *J* = 8.9 Hz, 1H), 5.09 (d, *J* = 11.0 Hz, 1H), 4.92 (d, *J* = 11.5 Hz, 1H), 4.80 (d, *J* = 11.1 Hz, 1H), 4.77 – 4.59 (m, 4H), 4.59 – 4.44 (m, 3H), 4.41 (d, *J* = 12.1 Hz, 1H), 4.36 – 4.18 (m, 3H), 4.13 (t, *J* = 9.4 Hz, 1H), 4.01 (t, *J* = 8.9 Hz, 1H), 3.89 (d, *J* = 2.9 Hz, 1H), 3.83 (dd, *J* = 11.0, 3.4 Hz, 1H), 3.76 (t, *J* = 8.6 Hz, 1H), 3.68 – 3.47 (m, 4H), 3.46 – 3.30 (m, 3H); ^{13}C NMR (100 MHz, $CDCl_3$) δ 143.4, 141.4, 139.0, 138.8, 138.7, 138.4, 138.1, 138.0, 132.3, 128.39 (2C), 128.36 (2C), 128.3 (two peaks overlapped, 4C), 128.2 (two peaks overlapped, 4C), 128.1 (2C), 127.9 (2C), 127.8 (2C), 127.72, 127.68 (2C), 127.6, 127.53, 127.48, 127.43 (2C), 127.41 (two peaks overlapped, 3C), 127.36, 123.1, 122.7, 119.8, 111.7, 102.8, 86.3, 83.3, 82.5, 80.0, 78.2, 75.7, 75.4, 75.2, 74.7, 73.6, 73.5, 73.2, 73.1, 72.6, 71.8, 68.2, 67.8; HRMS: m/z (ESI) calcd for $C_{61}H_{63}N_2O_{10}^+$, $[M + H]^+$, 983.4477, found 983.4495. $^1J^{13}{}_{C1-H1}$ = 154.6 Hz, 161.7 Hz.

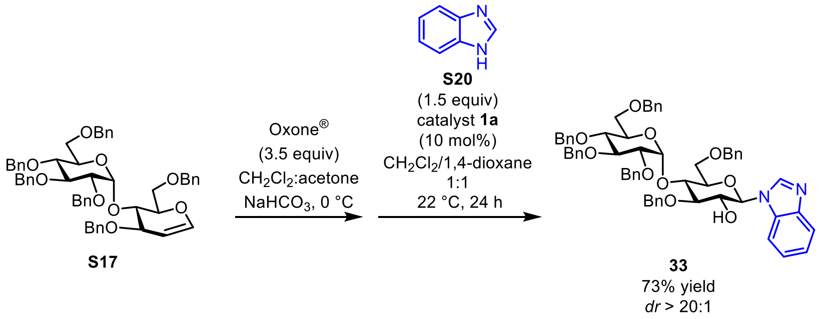

The reaction was carried out on a 0.1 mmol scale by following **Procedure A** for epoxidation and **Procedure D** for *N*-glycosylation. CH_2Cl_2/1,4-dioxane was used as the mixed solvent and the *N*-glycosylation was carried out at 22 °C for 24 h. The *dr* of the corresponding glycal α-epoxide is

>20:1. The *dr* of the glycosylation product determined based on crude reaction mixture is >20:1. The desired product **33** was purified through a silica gel flash column (hexanes/acetone: from 20:1 to 1:1) as white foam (71.8 mg, 73% yield).

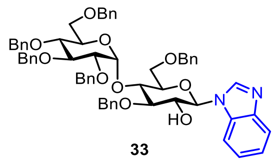

33

1-[(2,3,4,6-Tetra-*O*-benzyl-α-D-glucopyranosyl)-(1→4)-(3,6-di-*O*-benzyl-β-D-glucopyranosyl)]benzimidazole (**33**): IR ν_{max} (neat)/cm^{-1}: 3028 (w), 2920 (w), 1738 (s), 1454 (m), 1363 (m), 1228 (m), 1069 (s), 734 (s), 695 (s); ^{1}H NMR (400 MHz, $CDCl_3$) δ 7.74 (s, 1H), 7.60 (d, *J* = 8.1 Hz, 1H), 7.40 – 7.27 (m, 29H), 7.21 – 7.14 (m, 3H), 7.06 (t, *J* = 7.7 Hz, 1H), 5.79 (d, *J* = 3.6 Hz, 1H), 5.68 (brs, 1H), 5.34 (d, *J* = 8.7 Hz, 1H), 5.17 (d, *J* = 11.6 Hz, 1H), 4.97 (d, *J* = 10.9 Hz, 1H), 4.95 – 4.84 (m, 3H), 4.69 (ABq, $\Delta\nu_{AB}$ = 13.9 Hz, J_{AB} = 12.0 Hz, 2H), 4.61 (d, *J* = 12.1 Hz, 1H), 4.55 – 4.48 (m, 3H), 4.44 – 4.35 (m, 2H), 4.29 (t, *J* = 8.5 Hz, 1H), 4.06 – 3.95 (m, 3H), 3.94 – 3.83 (m, 2H), 3.81 – 3.71 (m, 2H), 3.69 – 3.59 (m, 2H), 3.53 (dd, *J* = 10.7, 2.0 Hz, 1H); ^{13}C NMR (100 MHz, $CDCl_3$) δ 143.1, 141.3, 138.7, 138.5, 138.4, 138.1, 137.9, 137.8, 131.8, 128.5 (2C), 128.38 (2C), 128.36 (2C), 128.35 (2C), 128.31 (2C), 128.26 (2C), 128.0 (2C), 127.94 (2C), 127.87 (2C), 127.80 (2C), 127.77, 127.71, 127.66, 127.6, 127.50, 127.49, 127.3 (2C), 127.2 (2C), 123.2, 122.9, 119.5, 112.1, 96.9, 87.1, 85.4, 82.0, 79.4, 77.6, 77.2, 75.6, 75.1, 74.4, 73.5, 73.43, 73.35, 72.9, 71.6, 71.2, 68.8, 68.2; HRMS: m/z (ESI) calcd for $C_{61}H_{63}N_2O_{10}^+$, $[M + H]^+$, 983.4477, found 983.4497. $^{1}J^{13}{}_{C1-H1}$ = 154.6 Hz, 170.2 Hz.

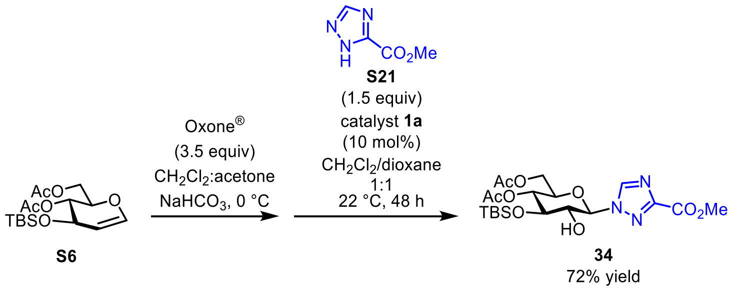

The reaction was carried out on a 0.1 mmol scale by following **Procedure A** for epoxidation and **Procedure D** for *N*-glycosylation. CH_2Cl_2/1,4-dioxane was used as the mixed solvent and the *N*-glycosylation was carried out at 22 °C for 48 h. The *dr* of the corresponding glycal α-epoxide is >20:1. The *dr* of the glycosylation product determined based on crude reaction mixture is >20:1. The desired product **34** was purified through a silica gel flash column (hexanes/acetone: from 20:1 to 3:2) as white foam (35.1 mg, 72% yield).

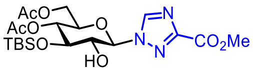

34

Methyl 1-(4,6-Di-*O*-acetyl-3-*O*-*tert*-butyldimethylsilyl-β-D-glucopyranosyl)1,2,4-triazole-3-carboxylate (**34**): IR ν_{max} (neat)/cm^{-1}: 3457 (w), 2970 (w), 1739 (s), 1464 (m), 1366 (m), 1217 (s), 1031 (m), 837 (m), 779 (m); ^{1}H NMR (400 MHz, $CDCl_3$) δ 8.39 (s, 1H), 5.34 (d, *J* = 9.0 Hz, 1H), 5.08 (t, *J* = 9.6 Hz, 1H), 4.24 (td, *J* = 8.9, 4.3 Hz, 1H), 4.17 (dd, *J* = 12.5, 5.3 Hz, 1H), 4.08 (d, *J* = 12.6 Hz, 1H), 3.97 (s, 3H), 3.90 (brs, 1H), 3.85 (t, *J* = 8.9 Hz, 1H), 3.81 – 3.72 (m, 1H), 2.09 (s, 3H), 2.04 (s, 3H), 0.85 (s, 9H), 0.12 (s, 3H), 0.08 (s, 3H); ^{13}C NMR (100 MHz, $CDCl_3$) δ 170.7, 169.4, 159.8, 154.7, 145.2, 87.8, 75.4, 75.1, 72.8, 70.0, 62.2, 52.9, 25.6 (3C), 21.1, 20.7, 18.0, -4.1, -4.9; HRMS: m/z (ESI) calcd for $C_{20}H_{34}N_3O_9Si^+$, [M + H]$^+$, 488.2059, found 488.2081. $^1J^{13}{}_{C1-H1}$ = 159.1 Hz.

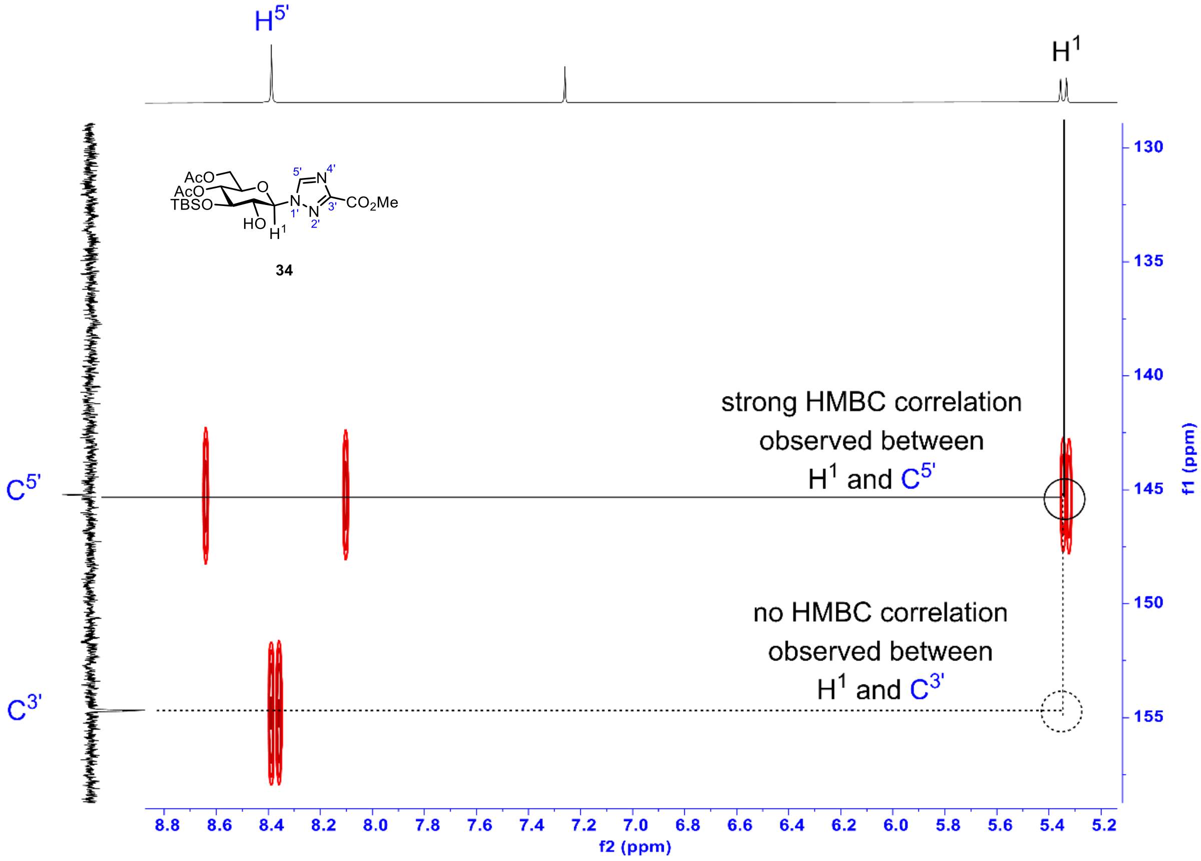

Figure S2. HMBC Analysis to Determine the Structure of **34**.

A strong HMBC correlation between H1 and C5', together with the absence of a correlation between H1 and C3', suggested that triazole **S21** gets glycosylated at the N1 position.

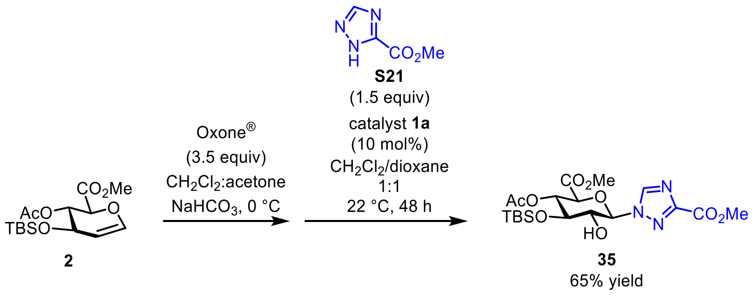

The reaction was carried out on a 0.2 mmol scale by following **Procedure A** for epoxidation and **Procedure D** for *N*-glycosylation. CH_2Cl_2/1,4-dioxane was used as the mixed solvent and the *N*-glycosylation was carried out at 22 °C for 48 h. The *dr* of the corresponding glycal α-epoxide is >20:1. The *dr* of the glycosylation product determined based on crude reaction mixture is >20:1. The desired product **35** was purified through a silica gel flash column (hexanes/acetone: from 20:1 to 3:2) as white foam (61.5 mg, 65% yield).

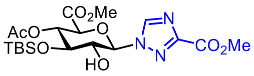

35

Methyl 1-(methyl 4-*O*-acetyl-3-*O*-*tert*-butyldimethylsilyl-β-D-glucopyranosyluronate)1,2,4-triazole-3-carboxylate (**35**): IR ν_{max} (neat)/cm^{-1}: 3456 (w), 2970 (w), 1740 (s), 1463 (m), 1372 (m), 1217 (s), 1033 (m), 835 (m), 780 (m); ^{1}H NMR (400 MHz, $CDCl_3$) δ 8.44 (s, 1H), 5.39 (d, *J* = 9.1 Hz, 1H), 5.12 (dd, *J* = 10.0, 9.0 Hz, 1H), 4.29 (td, *J* = 8.8, 4.5 Hz, 1H), 4.11 (d, *J* = 10.0 Hz, 1H), 3.97 (s, 3H), 3.90 (t, *J* = 8.9 Hz, 1H), 3.73 (brs, 1H), 3.70 (s, 3H), 2.08 (s, 3H), 0.85 (s, 9H), 0.13 (s, 3H), 0.10 (s, 3H); ^{13}C NMR (100 MHz, $CDCl_3$) δ 169.6, 166.9, 159.8, 154.7, 145.4, 87.2, 75.1, 75.0, 72.4, 71.3, 52.94, 52.91, 25.6 (3C), 20.8, 18.0, -4.3, -4.9; HRMS: m/z (ESI) calcd for $C_{19}H_{32}N_3O_9Si^+$, $[M + H]^+$, 474.1902, found 474.1921. $^1J^{13}{}_{C1–H1}$ = 158.4 Hz.

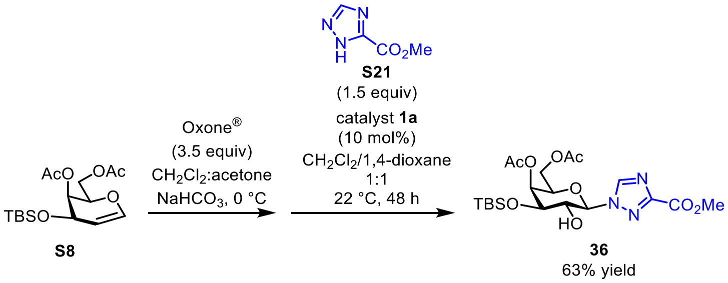

The reaction was carried out on a 0.2 mmol scale by following **Procedure A** for epoxidation and **Procedure D** for *N*-glycosylation. CH_2Cl_2/1,4-dioxane was used as the mixed solvent and the *N*-

glycosylation was carried out at 22 °C for 48 h. The *dr* of the corresponding glycal α-epoxide is >20:1. The *dr* of the glycosylation product determined based on crude reaction mixture is >20:1. The desired product **36** was purified through a silica gel flash column (hexanes/acetone: from 20:1 to 3:2) as white foam (61.2 mg, 63% yield).

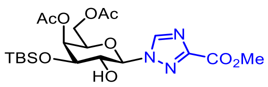

36

Methyl 1-(4,6-Di-*O*-acetyl-3-*O*-*tert*-butyldimethylsilyl-β-D-galactopyranosyl)1,2,4-triazole-3-carboxylate (**36**): IR ν_{max} (neat)/cm^{-1}: 3273 (w), 2970 (w), 1737 (s), 1466 (m), 1366 (m), 1229 (s), 1035 (m), 913 (m), 837 (m); ^{1}H NMR (400 MHz, $CDCl_3$) δ 8.42 (s, 1H), 5.363 (d, *J* = 9.1 Hz, 1H), 5.355 (d, *J* = 3.0 Hz, 1H), 4.38 (td, *J* = 9.1, 3.4 Hz, 1H), 4.17 (dd, *J* = 10.2, 3.9 Hz, 1H), 4.14 – 4.01 (m, 2H), 3.99 (s, 3H), 3.87 (dd, *J* = 9.2, 3.6 Hz, 1H), 3.07 (d, *J* = 4.1 Hz, 1H), 2.14 (s, 3H), 2.04 (s, 3H), 0.86 (s, 9H), 0.12 (s, 6H); ^{13}C NMR (100 MHz, $CDCl_3$) δ 170.5, 170.0, 159.9, 154.9, 145.1, 87.7, 74.3, 73.2, 69.9, 69.3, 62.1, 52.9, 25.5 (3C), 20.71, 20.70, 18.0, -4.8, -5.0; HRMS: m/z (ESI) calcd for $C_{20}H_{34}N_3O_9Si^+$, $[M + H]^+$, 488.2059, found 488.2075. $^1J^{13}{}_{C1\text{–}H1}$ = 158.5 Hz.

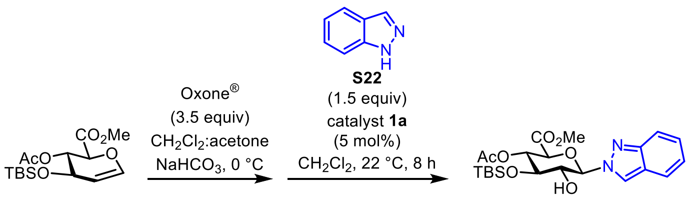

The reaction was carried out on a 0.1 mmol scale by following **Procedure A** for epoxidation and **Procedure D** for *N*-glycosylation. CH_2Cl_2 was used as the solvent and the *N*-glycosylation was carried out at 22 °C for 8 h (5 mol % of catalyst **1a** was used). The *dr* of the corresponding glycal α-epoxide is >20:1. The *dr* of the glycosylation product determined based on crude reaction mixture is >20:1. The desired product **37** was purified through a silica gel flash column (hexanes/EtOAc: from 20:1 to 3:1) as white foam (40.3 mg, 87% yield).

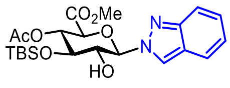

37

2-(Methyl 4-*O*-acetyl-3-*O*-*tert*-butyldimethylsilyl-β-D-glucopyranosyluronate)indazole (37): IR ν_{max} (neat)/cm^{-1}: 3460 (w), 2971 (w), 1743 (s), 1467 (m), 1372 (m), 1221 (s), 1037 (m), 835 (m), 788 (m); ^{1}H NMR (400 MHz, $CDCl_3$) δ 8.22 (s, 1H), 7.71 – 7.63 (m, 2H), 7.35 – 7.26 (m, 1H), 7.13 – 7.06 (m, 1H), 5.48 (d, *J* = 8.8 Hz, 1H), 5.17 (dd, *J* = 10.1, 9.0 Hz, 1H), 4.23 (td, *J* = 8.8, 2.8 Hz, 1H), 4.18 (d, *J* = 10.1 Hz, 1H), 3.98 (t, *J* = 8.9 Hz, 1H), 3.84 (d, *J* = 2.9 Hz, 1H), 3.73 (s, 3H), 2.10 (s, 3H), 0.88 (s, 9H), 0.17 (s, 3H), 0.13 (s, 3H); ^{13}C NMR (100 MHz, $CDCl_3$) δ 169.7, 167.3, 148.7, 127.1, 123.1, 122.4, 121.6, 120.6, 117.7, 89.2, 75.1, 74.9, 73.8, 71.5, 52.9, 25.6 (3C), 20.8, 18.1, -4.2, -4.9; HRMS: m/z (ESI) $C_{22}H_{33}N_2O_7Si^+$, $[M + H]^+$, 465.2052, found 465.2064. $^{1}J^{13}_{C1-H1}$ = 159.3 Hz.

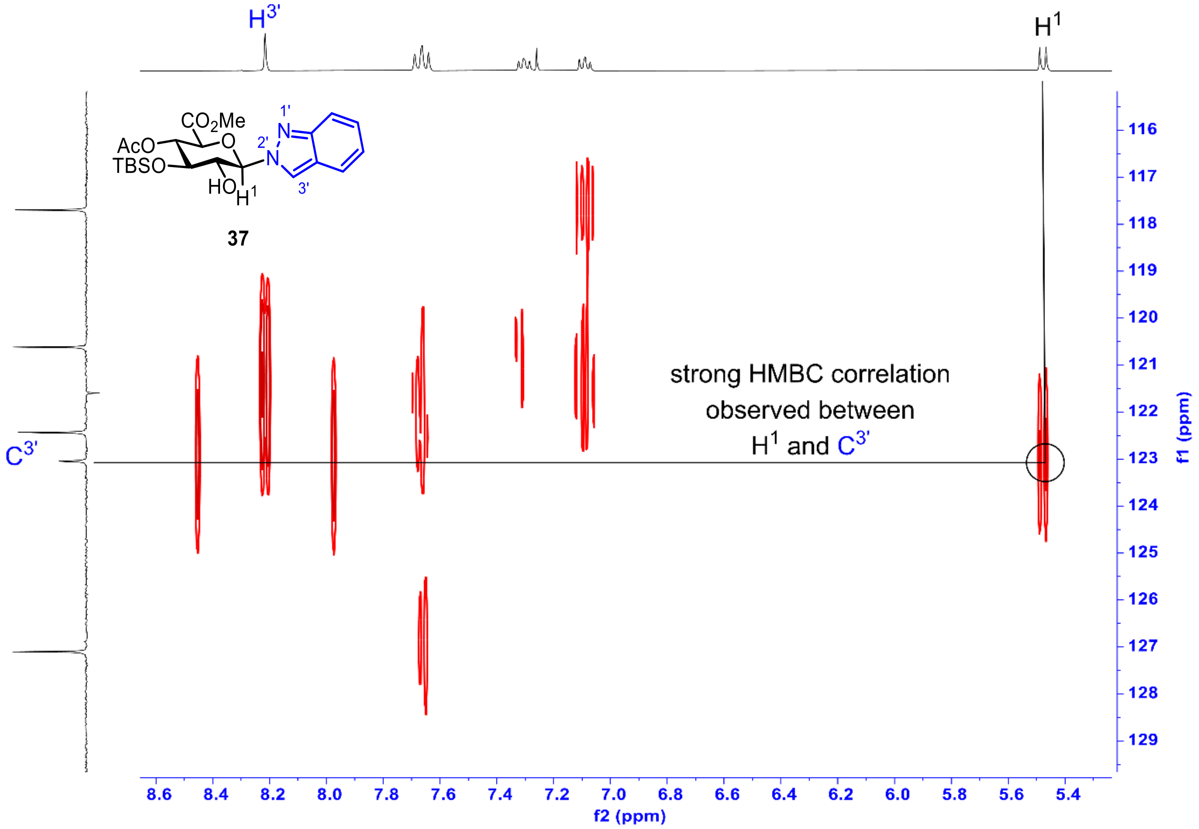

Figure S3. HMBC Analysis to Determine the Structure of **37**.

A strong HMBC correlation between H1 and C3' suggested that indazole **S22** gets glycosylated at the N2 position.

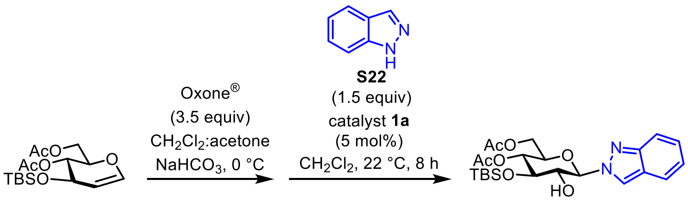

S6 **38**
91% yield

The reaction was carried out on a 0.1 mmol scale by following **Procedure A** for epoxidation and **Procedure D** for *N*-glycosylation. CH_2Cl_2 was used as the solvent and the *N*-glycosylation was carried out at 22 °C for 8 h (5 mol % of catalyst **1a** was used). The *dr* of the corresponding glycal α-epoxide is >20:1. The *dr* of the glycosylation product determined based on crude reaction mixture is >20:1. The desired product **38** was purified through a silica gel flash column (hexanes/Et_2O: from 20:1 to 1:1) as white foam (43.3 mg, 91% yield).

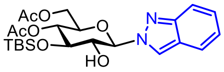

38

2-(4,6-Di-*O*-acetyl-3-*O*-*tert*-butyldimethylsilyl-β-D-glucopyranosyl)indazole (**38**): IR ν_{max} (neat)/cm^{-1}: 3462 (w), 2970 (w), 1740 (s), 1472 (m), 1371 (m), 1228 (s), 1046 (m), 836 (s), 779 (m); ^{1}H NMR (400 MHz, $CDCl_3$) δ 8.18 (d, *J* = 1.0 Hz, 1H), 7.72 – 7.64 (m, 2H), 7.35 – 7.28 (m, 1H), 7.14 – 7.07 (m, 1H), 5.46 (d, *J* = 8.8 Hz, 1H), 5.12 (dd, *J* = 10.2, 9.0 Hz, 1H), 4.25 (dd, *J* = 12.4, 5.5 Hz, 1H), 4.21 – 4.12 (m, 2H), 3.93 (t, *J* = 9.0 Hz, 1H), 3.86 (ddd, *J* = 10.0, 5.3, 2.4 Hz, 1H), 3.81 – 3.75 (m, 1H), 2.12 (s, 3H), 2.07 (s, 3H), 0.88 (s, 9H), 0.16 (s, 3H), 0.12 (s, 3H); ^{13}C NMR (100 MHz, $CDCl_3$) δ 170.8, 169.6, 148.7, 127.0, 122.9, 122.4, 121.5, 120.6, 117.7, 89.6,

75.3, 75.1, 74.2, 70.4, 62.5, 25.7 (3C), 21.1, 20.8, 18.1, -4.1, -4.9; HRMS: m/z (ESI) calcd for $C_{23}H_{35}N_2O_7Si^+$, [M + H]$^+$, 479.2208, found 479.2236. $^1J^{13}{}_{C1-H1}$ = 157.6 Hz.

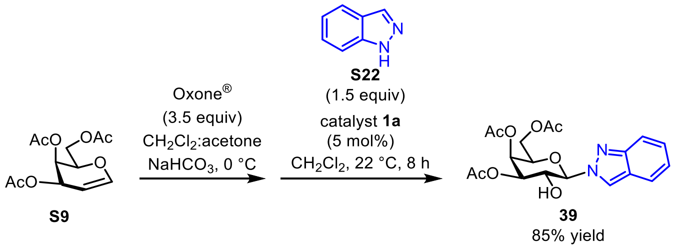

The reaction was carried out on a 0.1 mmol scale by following **Procedure A** for epoxidation and **Procedure D** for *N*-glycosylation. CH_2Cl_2 was used as the solvent and the *N*-glycosylation was carried out at 22 °C for 8 h (5 mol % of catalyst **1a** was used). The *dr* of the corresponding glycal α-epoxide is >20:1. The *dr* of the glycosylation product determined based on crude reaction mixture is >20:1. The desired product **39** was purified through a silica gel flash column (hexanes/acetone: from 20:1 to 2:1) as white foam (33.3 mg, 85% yield).

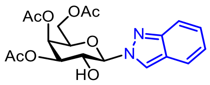

39

2-(3,4,6-Tri-*O*-acetyl-β-D-galactopyranosyl)indazole (**39**): IR ν_{max} (neat)/cm^{-1}: 3447 (w), 2923 (w), 1740 (s), 1432 (m), 1368 (m), 1216 (s), 1048 (m), 922 (m), 729 (m); ^{1}H NMR (400 MHz, $CDCl_3$) δ 8.22 (s, 1H), 7.68 (dt, *J* = 8.5, 1.3 Hz, 2H), 7.35 – 7.27 (m, 1H), 7.15 – 7.06 (m, 1H), 5.56 – 5.49 (m, 2H), 5.19 (dd, *J* = 10.2, 3.4 Hz, 1H), 4.67 – 4.508 (m, 1H), 4.25 – 4.15 (m, 3H), 4.10 (d, *J* = 3.3 Hz, 1H), 2.14 (s, 3H), 2.06 (s, 3H), 2.05 (s, 3H); ^{13}C NMR (100 MHz, $CDCl_3$) δ 170.4, 170.3, 170.1, 148.7, 127.1, 123.3, 122.5, 121.4, 120.6, 117.7, 89.8, 73.6, 72.5, 68.2, 67.0, 61.5, 20.7, 20.64, 20.58; HRMS: m/z (ESI) calcd for $C_{19}H_{23}N_2O_8^+$, [M + H]$^+$, 407.1449, found 407.1460. $^1J^{13}{}_{C1-H1}$ = 157.9 Hz.

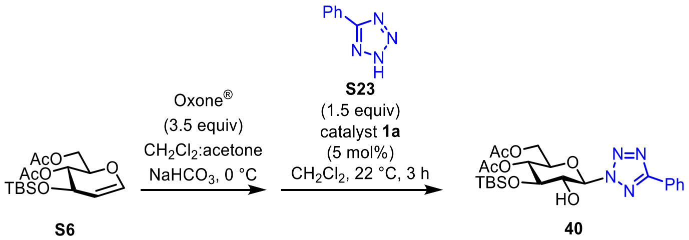

The reaction was carried out on a 0.1 mmol scale by following **Procedure A** for epoxidation and **Procedure D** for *N*-glycosylation. CH_2Cl_2 was used as the solvent and the *N*-glycosylation was carried out at 22 °C for 3 h (5 mol % of catalyst **1a** was used). The *dr* of the corresponding glycal α-epoxide is >20:1. The *dr* of the glycosylation product determined based on crude reaction mixture is >20:1. The desired product **40** was purified through a silica gel flash column (hexanes/EtOAc: from 20:1 to 4:1) as white foam (39.9 mg, 79% yield).

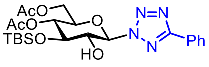

40

2-(4,6-Di-*O*-acetyl-3-*O*-*tert*-butyldimethylsilyl-β-D-glucopyranosyl)5-phenyltetrazole (40): IR ν_{max} (neat)/cm^{-1}: 3455 (w), 2929 (w), 1740 (s), 1451 (m), 1366 (m), 1228 (s), 1035 (m), 835 (m), 779 (m); ^{1}H NMR (400 MHz, $CDCl_3$) δ 8.19 – 8.07 (m, 2H), 7.52 – 7.44 (m, 3H), 5.82 (dd, *J* = 9.3, 1.0 Hz, 1H), 5.16 (dd, *J* = 10.2, 9.0 Hz, 1H), 4.55 (td, *J* = 9.1, 4.8 Hz, 1H), 4.20 (dd, *J* = 12.7, 5.4 Hz, 1H), 4.12 (d, *J* = 12.2 Hz, 1H), 3.93 (t, *J* = 9.0 Hz, 1H), 3.90 – 3.83 (m, 1H), 2.90 – 2.66 (m, 1H), 2.12 (s, 3H), 2.05 (s, 3H), 0.89 (s, 9H), 0.15 (s, 3H), 0.12 (s, 3H); ^{13}C NMR (100 MHz, $CDCl_3$) δ 170.8, 169.5, 165.5, 130.7, 128.9 (2C), 127.1 (2C), 126.7, 89.1, 75.5, 75.2, 72.5, 70.0, 62.1, 25.6 (3C), 21.1, 20.8, 18.0, -4.1, -4.8; HRMS: m/z (ESI) calcd for $C_{23}H_{35}N_4O_7Si^+$, [M + H]$^+$, 507.2270, found 507.2291. $^1J^{13}_{C1-H1}$ = 161.3 Hz.

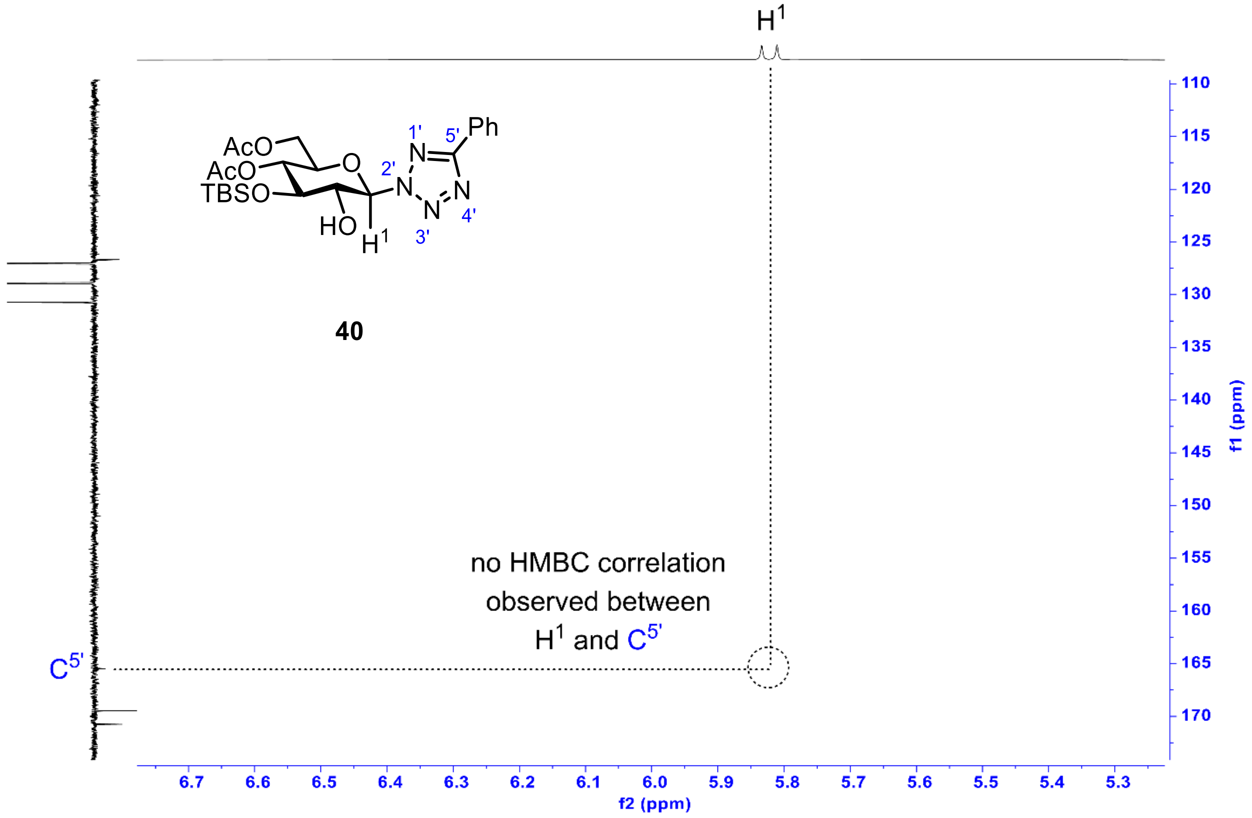

Figure S4. HMBC Analysis to Determine the Structure of **40**.

The absence of an HMBC correlation between H1 and C5' suggested that tetrazole **S23** gets glycosylated at the N2 position.

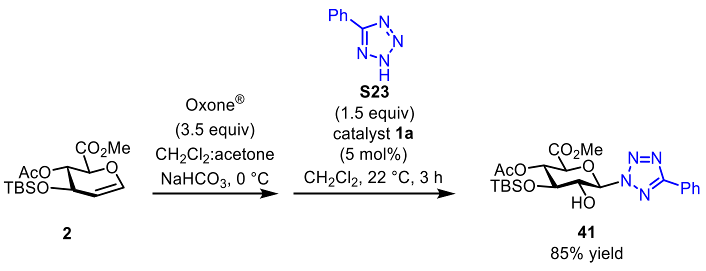

The reaction was carried out on a 0.2 mmol scale by following **Procedure A** for epoxidation and **Procedure D** for *N*-glycosylation. CH_2Cl_2 was used as the solvent and the *N*-glycosylation was

carried out at 22 °C for 3 h (5 mol % of catalyst **1a** was used). The *dr* of the corresponding glycal α-epoxide is >20:1. The *dr* of the glycosylation product determined based on crude reaction mixture is >20:1. The desired product **41** was purified through a silica gel flash column (hexanes/EtOAc: from 20:1 to 3:1) as white foam (83.6 mg, 85% yield).

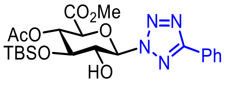

41

2-(Methyl 4-*O*-acetyl-3-*O*-*tert*-butyldimethylsilyl-β-D-glucopyranosyluronate)5-phenyltetrazole (41): IR ν_{max} (neat)/cm^{-1}: 3308 (w), 2948 (w), 1763 (m), 1736 (s), 1471 (m), 1375 (m), 1255 (m), 1136 (m), 834 (m); ^{1}H NMR (400 MHz, $CDCl_3$) δ 8.13 (dd, *J* = 6.7, 3.0 Hz, 2H), 7.55 – 7.44 (m, 3H), 5.83 (d, *J* = 9.3 Hz, 1H), 5.19 (dd, *J* = 10.1, 9.1 Hz, 1H), 4.61 (td, *J* = 9.1, 4.8 Hz, 1H), 4.19 (d, *J* = 10.1 Hz, 1H), 3.98 (t, *J* = 9.0 Hz, 1H), 3.71 (s, 3H), 2.85 (d, *J* = 4.8 Hz, 1H), 2.11 (s, 3H), 0.89 (s, 9H), 0.16 (s, 3H), 0.13 (s, 3H); ^{13}C NMR (100 MHz, $CDCl_3$) δ 169.6, 166.8, 165.5, 130.8, 128.9 (2C), 127.1 (2C), 126.6, 88.6, 75.09, 75.07, 72.1, 71.3, 53.0, 25.6 (3C), 20.8, 18.0, -4.2, -4.8; HRMS: m/z (ESI) calcd for $C_{22}H_{33}N_4O_7Si^+$, $[M + H]^+$, 493.2113, found 493.2119. $^1J^{13}{}_{C1–H1}$ = 159.9 Hz.

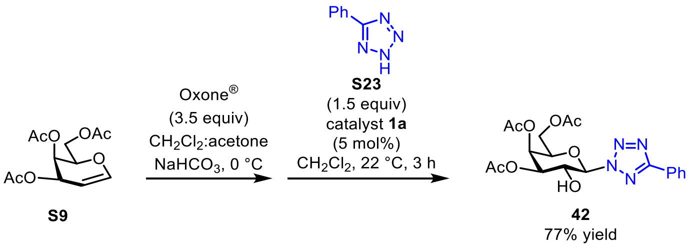

The reaction was carried out on a 0.2 mmol scale by following **Procedure A** for epoxidation and **Procedure D** for *N*-glycosylation. CH_2Cl_2 was used as the solvent and the *N*-glycosylation was carried out at 22 °C for 3 h (5 mol % of catalyst **1a** was used). The *dr* of the corresponding glycal α-epoxide is >20:1. The *dr* of the glycosylation product determined based on crude reaction mixture is >20:1. The desired product **42** was purified through a silica gel flash column (hexanes/EtOAc: from 20:1 to 1:1) as white foam (66.8 mg, 77% yield).

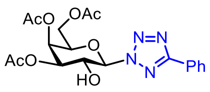

42

2-(3,4,6-Tri-*O*-acetyl-β-D-galactopyranosyl)5-phenyltetrazole (**42**): IR ν_{max} (neat)/cm^{-1}: 3446 (w), 2970 (w), 1741 (s), 1450 (m), 1367 (m), 1227 (s), 1086 (m), 923 (m), 693 (m); ^{1}H NMR (400 MHz, $CDCl_3$) δ 8.20 – 8.07 (m, 2H), 7.46 (dd, J = 4.8, 2.0 Hz, 3H), 5.92 (d, J = 9.1 Hz, 1H), 5.53 (dd, J = 3.5, 1.2 Hz, 1H), 5.19 (dd, J = 10.2, 3.4 Hz, 1H), 4.99 – 4.85 (m, 1H), 4.26 (t, J = 6.4 Hz, 1H), 4.21 – 4.04 (m, 2H), 3.46 (d, J = 5.2 Hz, 1H), 2.20 (s, 3H), 2.06 (s, 3H), 2.02 (s, 3H); ^{13}C NMR (100 MHz, $CDCl_3$) δ 170.43, 170.41, 170.1, 165.3, 130.8, 128.9 (2C), 127.0 (2C), 126.5, 89.6, 73.8, 72.9, 67.0 (two peaks overlapped, 2C), 61.3, 20.64, 20.62, 20.60; HRMS: m/z (ESI) calcd for $C_{19}H_{23}N_4O_8^+$, $[M + H]^+$, 435.1510, found 435.1523. $^1J^{13}_{C1-H1}$ = 159.6 Hz.

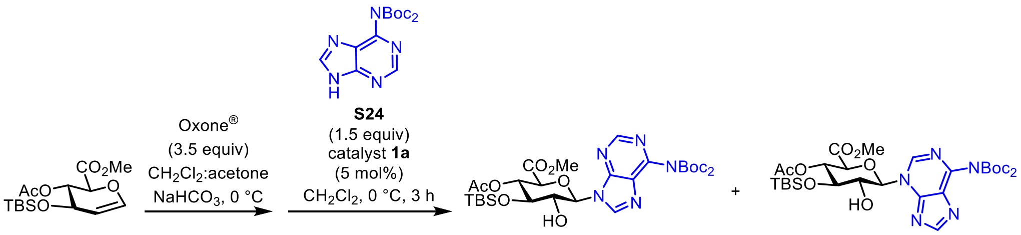

2 **43a** 38% yield **43b** 48% yield

S24 was synthesized according to a literature procedure.[10]

The reaction was carried out on a 0.1 mmol scale by following **Procedure A** for epoxidation and **Procedure D** for *N*-glycosylation. CH_2Cl_2 was used as the solvent and the *N*-glycosylation was carried out at 0 °C for 3 h (5 mol % of catalyst **1a** was used). The *dr* of the corresponding glycal α-epoxide is >20:1. The *dr* of the glycosylation product determined based on crude reaction mixture is >20:1. The desired product **43a** (25.9 mg, 38% yield, white foam) and **43b** (32.7 mg, 48% yield, white foam) were purified through a silica gel flash column (hexanes/EtOAc: from 20:1 to 3:2).

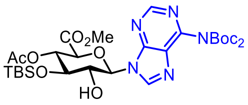

43a

***N*,*N*-Di-*tert*-butoxycarbonyl-9-(methyl 4-*O*-acetyl-3-*O*-*tert*-butyldimethylsilyl-β-D-glucopyranosyluronate)adenine** (**43a**): IR ν_{max} (neat)/cm^{-1}: 3461 (w), 2970 (w), 1740 (s), 1446 (m), 1370 (m), 1228 (s), 1105 (m), 839 (m), 777 (m); ^{1}H NMR (400 MHz, $CDCl_3$) ^{1}H NMR (400 MHz, $CDCl_3$) δ 8.84 (s, 1H), 8.26 (s, 1H), 5.64 (d, *J* = 9.4 Hz, 1H), 5.22 – 5.09 (m, 1H), 4.27 (td, *J* = 8.9, 4.5 Hz, 1H), 4.17 (d, *J* = 10.0 Hz, 1H), 3.94 (td, *J* = 8.8, 2.1 Hz, 1H), 3.70 (s, 3H), 3.43 – 3.33 (m, 1H), 2.10 (s, 3H), 1.45 (s, 18H), 0.87 (s, 9H), 0.13 (s, 3H), 0.12 (s, 3H); ^{13}C NMR (100 MHz, $CDCl_3$) δ 169.7, 167.1, 153.0, 152.3, 150.7, 150.4 (2C), 143.3, 128.8, 84.0 (2C), 83.3, 75.5, 75.0, 72.8, 71.4, 52.9, 27.8 (6C), 25.6 (3C), 20.8, 18.0, -4.2, -4.8; HRMS: m/z (ESI) calcd for $C_{30}H_{48}N_5O_{11}Si^+$, $[M + H]^+$, 682.3114, found 682.3128. $^{1}J^{13}{}_{C1-H1}$ = 155.7 Hz.

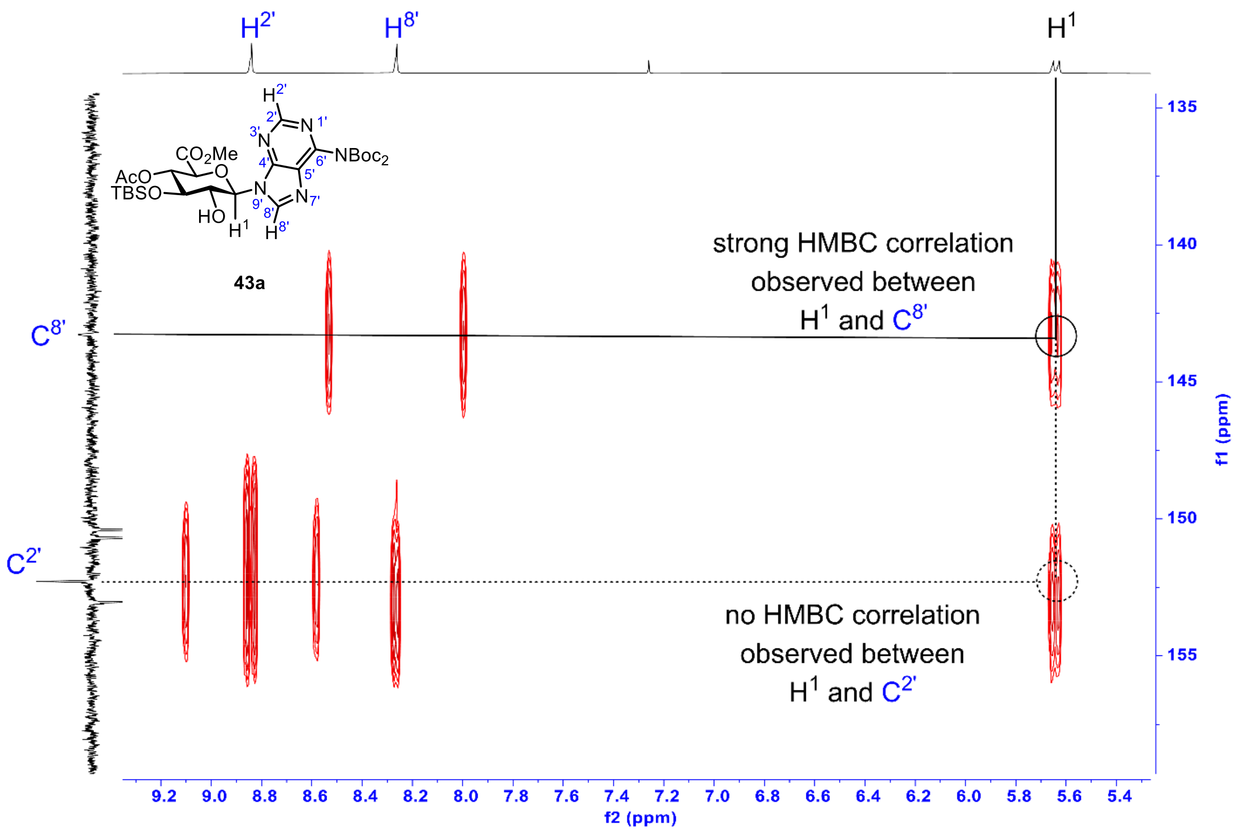

Figure S5. HMBC Analysis to Determine the Structure of **43a**.

A strong HMBC correlation between H1 and C8', together with the lack of a correlation between H1 and C2', suggested that a glycosidic linkage is formed at the N9 position in **43a**.

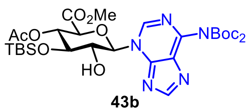

43b

***N*,*N*-Di-*tert*-butoxycarbonyl-3-(methyl 4-*O*-acetyl-3-*O*-*tert*-butyldimethylsilyl-β-D-glucopyranosyluronate)adenine** (**43b**): IR ν_{max} (neat)/cm^{-1}: 3460 (w), 2975 (w), 1744 (s), 1441 (m), 1374 (m), 1226 (s), 1109 (m), 780 (m); ^{1}H NMR (400 MHz, $CDCl_3$) δ 8.75 (s, 1H), 8.37 (s, 1H), 6.17 (d, *J* = 8.9 Hz, 1H), 5.19 (dd, *J* = 9.6, 8.6 Hz, 1H), 4.25 (d, *J* = 9.6 Hz, 1H), 4.14 – 4.07 (m, 1H), 4.07 – 3.98 (brs, 1H), 4.02 (t, *J* = 8.4 Hz, 1H), 3.73 (s, 3H), 2.11 (s, 3H), 1.49 (s, 18H),

0.84 (s, 9H), 0.114 (s, 3H), 0.106 (s, 3H); ^{13}C NMR (100 MHz, $CDCl_3$) δ 169.7, 166.8, 161.1, 156.4, 150.1 (2C), 147.3, 137.6, 133.1, 87.4, 84.5 (2C), 75.3 (two peaks overlapped, 2C), 74.7, 71.2, 53.0, 27.7 (6C), 25.6 (3C), 20.8, 18.0, -4.3, -4.9; HRMS: m/z (ESI) calcd for $C_{30}H_{48}N_5O_{11}Si^+$, $[M + H]^+$, 682.3114, found 682.3132. $^1J^{13}{}_{C1–H1}$ = 160.2 Hz.

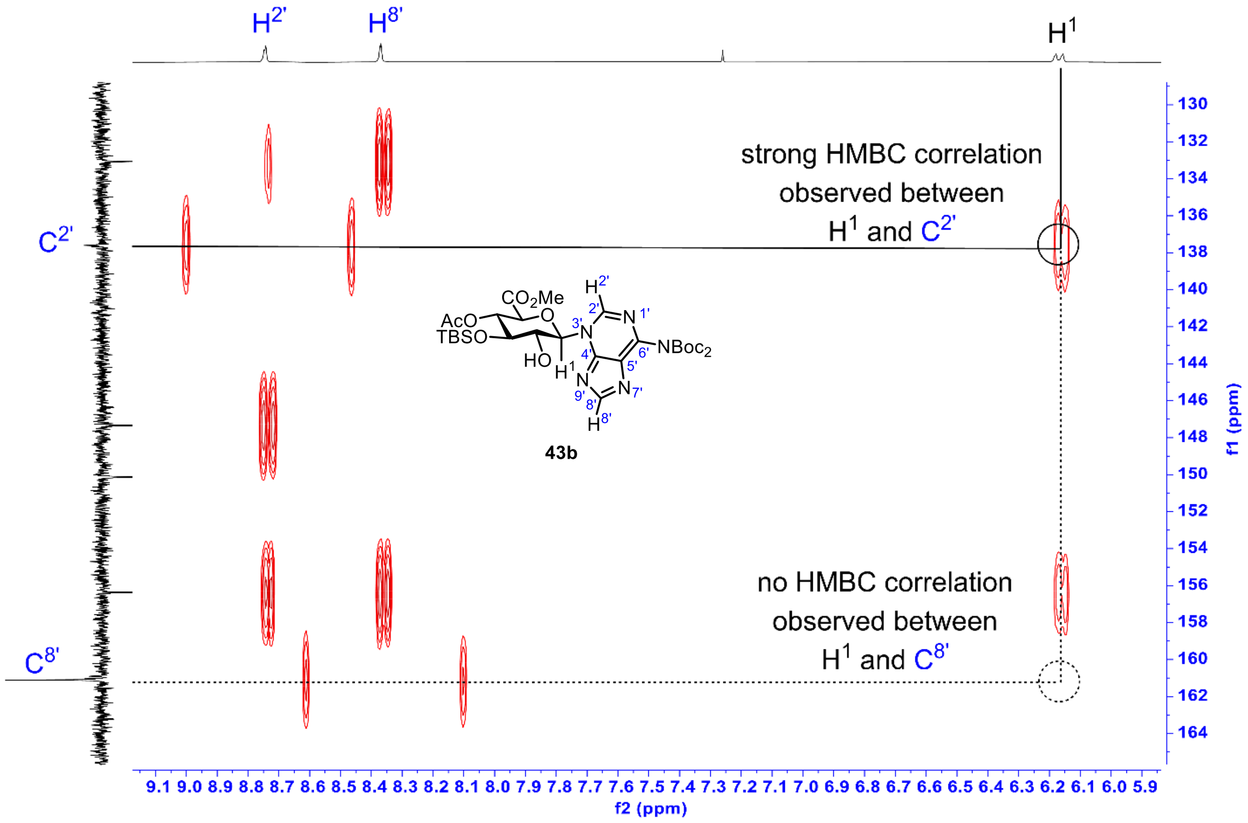

Figure S6. HMBC Analysis to Determine the Structure of **43b**.

A strong HMBC correlation between H1 and C2', together with lack of a correlation between H1 and C8 ', suggested that a glycosidic linkage is formed at the N3 position in **43b**.

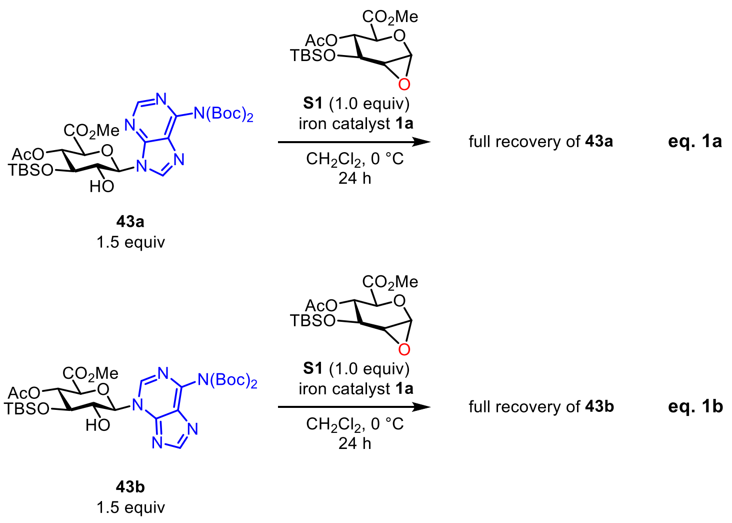

Figure S7. Control Experiments for Product Interconversion between **43a** and **43b**.

The *N*-glycosylation products **43a** and **43b** were separated through flash column chromatography and independently resubjected to the reaction conditions (Figure S7). In both cases, the starting material was fully recovered and no interconversion between **43a** and **43b** was observed.

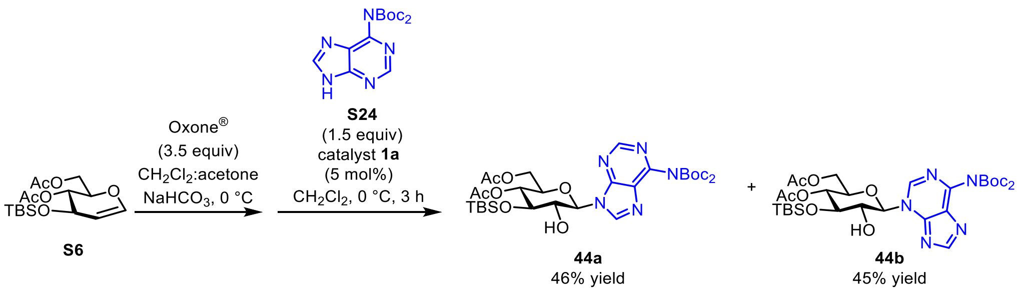

The reaction was carried out on a 0.1 mmol scale by following **Procedure A** for epoxidation and **Procedure D** for *N*-glycosylation. CH_2Cl_2 was used as the solvent and the *N*-glycosylation was carried out at 0 °C for 3 h (5 mol % of catalyst **1a** was used). The *dr* of the corresponding glycal α-epoxide is >20:1. The *dr* of the glycosylation product determined based on crude reaction mixture is >20:1. The desired product **44a** (32.0 mg, 46% yield, white foam) and **44b** (31.2 mg,

45% yield, white foam) were purified through a silica gel flash column (hexanes/EtOAc: from 20:1 to 3:2).

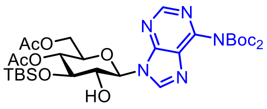

44a

***N,N*-Di-*tert*-butoxycarbonyl-9-(4,6-Di-*O*-acetyl-3-*O*-*tert*-butyldimethylsilyl-β-D-glucopyranosyl)adenine** (**44a**): IR ν_{max} (neat)/cm^{-1}: 2975 (w), 1743 (s), 1452 (w), 1371 (m), 1234 (s), 1130 (m), 1056 (m), 845 (m); ^{1}H NMR (400 MHz, $CDCl_3$) δ 8.86 (s, 1H), 8.23 (s, 1H), 5.63 (d, *J* = 9.2 Hz, 1H), 5.12 (dd, *J* = 10.4, 9.2 Hz, 1H), 4.27 – 4.16 (m, 2H), 4.14 – 4.06 (m, 1H), 3.91 (t, *J* = 8.9 Hz, 1H), 3.85 (ddd, *J* = 10.1, 5.4, 2.4 Hz, 1H), 3.41 (brs, 1H), 2.12 (s, 3H), 2.05 (s, 3H), 1.47 (s, 18H), 0.88 (s, 9H), 0.14 (s, 3H), 0.11 (s, 3H); ^{13}C NMR (100 MHz, $CDCl_3$) δ 170.7, 169.6, 153.0, 152.2, 150.7, 150.5 (2C), 143.2, 128.8, 84.0 (2C), 83.9, 75.8, 75.1, 73.4, 70.1, 62.2, 27.8 (6C), 25.6 (3C), 21.1, 20.7, 18.0, -4.1, -4.8; HRMS: m/z (ESI) calcd for $C_{31}H_{50}N_5O_{11}Si^+$, [M + H]$^+$, 696.3271, found 696.3289. $^{1}J^{13}{}_{C1-H1}$ = 156.5 Hz.

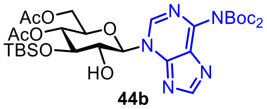

44b

***N,N*-Di-*tert*-butoxycarbonyl-3-(4,6-Di-*O*-acetyl-3-*O*-*tert*-butyldimethylsilyl-β-D-glucopyranosyl)adenine** (**44b**): IR ν_{max} (neat)/cm^{-1}: 2970 (w), 1740 (s), 1456 (w), 1369 (m), 1230 (s), 1134 (m), 1051 (m), 838 (m), 780 (m); ^{1}H NMR (400 MHz, $CDCl_3$) δ 8.64 (s, 1H), 8.39 (s, 1H), 6.13 (d, *J* = 9.1 Hz, 1H), 5.14 (dd, *J* = 10.2, 8.9 Hz, 1H), 4.23 (dd, *J* = 12.7, 5.6 Hz, 1H), 4.13 (dd, *J* = 12.7, 2.2 Hz, 1H), 4.06 (t, *J* = 8.8 Hz, 1H), 3.97 (t, *J* = 8.8 Hz, 1H), 3.91 (ddd, *J* = 10.0, 5.6, 2.1 Hz, 1H), 3.86 (brs, 1H), 2.12 (s, 3H), 2.06 (s, 3H), 1.50 (s, 18H), 0.85 (s, 9H), 0.11 (s, 3H), 0.10 (s, 3H); ^{13}C NMR (100 MHz, $CDCl_3$) δ 170.6, 169.5, 161.3, 156.5, 150.2 (2C), 147.3, 137.5, 133.3, 87.9, 84.5 (2C), 75.9 (two peaks overlapped, 2C), 75.1, 69.9, 62.1, 27.8 (6C), 25.6 (3C), 21.1, 20.7, 18.0, -4.1, -4.9; HRMS: m/z (ESI) calcd for $C_{31}H_{50}N_5O_{11}Si^+$, [M + H]$^+$, 696.3271, found 696.3285. $^{1}J^{13}{}_{C1-H1}$ = 162.8 Hz.

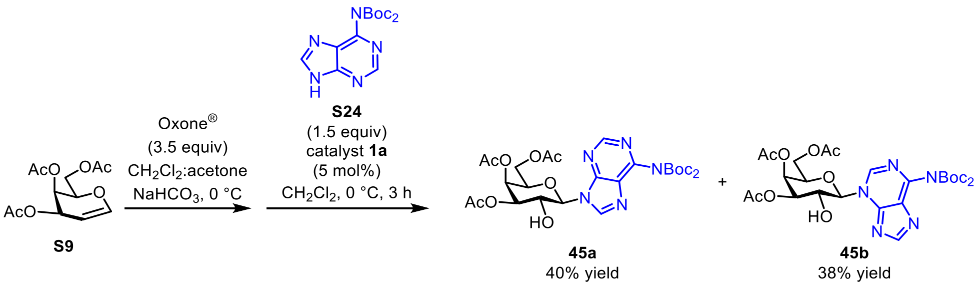

The reaction was carried out on a 0.2 mmol scale by following **Procedure A** for epoxidation and **Procedure D** for *N*-glycosylation. CH_2Cl_2 was used as the solvent and the *N*-glycosylation was carried out at 0 °C for 3 h (5 mol % of catalyst **1a** was used). The *dr* of the corresponding glycal α-epoxide is >20:1. The *dr* of the glycosylation product determined based on crude reaction mixture is >20:1. The desired product **45a** (50.2 mg, 40% yield, white foam) and **45b** (47.8 mg, 38% yield, white foam) were purified through a silica gel flash column (hexanes/EtOAc: from 20:1 to 3:2).

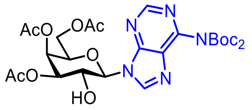

45a

***N*,*N*-Di-*tert*-butoxycarbonyl-9-(3,4,6-Tri-*O*-acetyl-β-D-galactopyranosyl)adenine (45a)**: IR ν_{max} (neat)/cm^{-1}: 3461 (w), 2970 (w), 1742 (s), 1455 (m), 1368 (m), 1217 (m), 1089 (m), 917 (m), 849 (m); ^{1}H NMR (400 MHz, $CDCl_3$) δ 8.83 (s, 1H), 8.27 (s, 1H), 5.67 (d, *J* = 9.1 Hz, 1H), 5.53 (dd, *J* = 3.3, 1.2 Hz, 1H), 5.14 (dd, *J* = 10.0, 3.3 Hz, 1H), 4.64 (t, *J* = 9.6 Hz, 1H), 4.27 – 4.21 (m, 1H), 4.21 – 4.12 (m, 3H), 2.18 (s, 3H), 2.07 (s, 3H), 2.04 (s, 3H), 1.46 (s, 18H); ^{13}C NMR (100 MHz, $CDCl_3$) δ 170.37, 170.36, 170.0, 152.8, 152.1, 150.8, 150.5 (2C), 143.4, 128.8, 84.9, 84.1 (2C), 73.6, 73.2, 67.4, 67.1, 61.3, 27.8 (6C), 20.7, 20.63, 20.61; HRMS: m/z (ESI) calcd for for $C_{27}H_{38}N_5O_{12}^+$, [M + H]$^+$, 624.2511, found 624.2527. $^1J^{13}{}_{C1\text{–}H1}$ = 157.1 Hz.

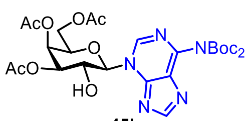

45b

***N*,*N*-Di-*tert*-butoxycarbonyl-3-(3,4,6-Tri-*O*-acetyl-β-D-galactopyranosyl)adenine (45b)**: IR ν_{max} (neat)/cm^{-1}: 3461 (w), 2970 (w), 1740 (s), 1456 (m), 1369 (m), 1227 (s), 1103 (m), 838 (m), 777 (m); ^{1}H NMR (400 MHz, $CDCl_3$) δ 8.70 (s, 1H), 8.24 (d, J = 4.8 Hz, 1H), 6.20 (dd, J = 9.0, 1.9 Hz, 1H), 5.57 (d, J = 3.2 Hz, 1H), 5.31 – 5.12 (m, 2H), 4.49 – 4.30 (m, 2H), 4.25 – 4.11 (m, 2H), 2.20 (s, 3H), 2.06 (s, 3H), 2.03 (s, 3H), 1.49 (s, 18H); ^{13}C NMR (100 MHz, $CDCl_3$) δ 170.3 (two peaks overlapped, 2C), 169.8, 160.9, 156.3, 150.2 (2C), 147.2, 137.8, 133.1, 88.1, 84.6 (2C), 74.5, 73.0, 69.4, 67.3, 61.4, 27.7 (6C), 20.7, 20.62, 20.61; HRMS: m/z (ESI) calcd for $C_{27}H_{38}N_5O_{12}^+$, $[M + H]^+$, 624.2511, found 624.2522. $^1J^{13}_{C1–H1}$ = 162.9 Hz .

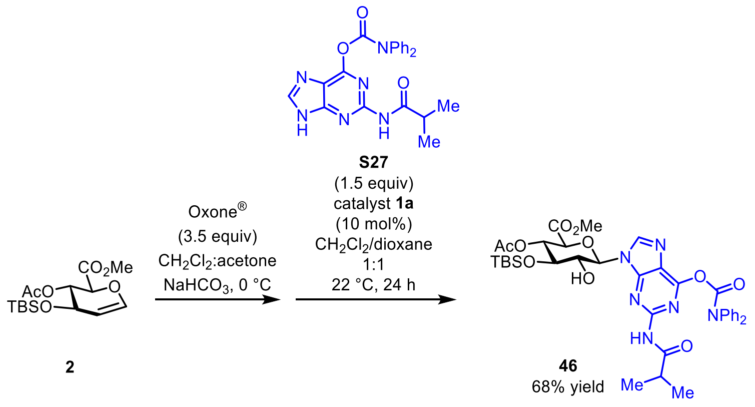

S27 was synthesized according to a literature procedure.[11]

The reaction was carried out on a 0.1 mmol scale by following **Procedure A** for epoxidation and **Procedure D** for *N*-glycosylation. CH_2Cl_2/1,4-dioxane was used as the mixed solvent and the *N*-glycosylation was carried out at 22 °C for 24 h. The *dr* of the corresponding glycal α-epoxide is >20:1. The *dr* of the glycosylation product determined based on crude reaction mixture is >20:1. The desired product **46** (51.5 mg, 68% yield, white foam) was purified through a silica gel flash column (hexanes/acetone: from 20:1 to 2:1).

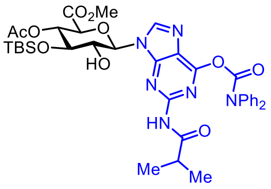

46

6-*O*-Diphenylcarbamoyl-2-*N*-isobutylryl-9-(methyl 4-*O*-acetyl-3-*O*-*tert*-butyldimethylsilyl-β-D-glucopyranosyluronate)guanine (**46**): IR ν_{max} (neat)/cm^{-1}: 3460 (w), 2974 (w), 1746 (s), 1596 (s), 1445 (m), 1372 (m), 1225 (s), 784 (m); ^{1}H NMR (400 MHz, $CDCl_3$-D_2O) δ 8.14 (s, 1H), 7.43 (d, *J* = 7.8 Hz, 4H), 7.36 (t, *J* = 7.8 Hz, 4H), 7.29 – 7.22 (m, 2H), 5.77 (d, *J* = 8.9 Hz, 1H), 5.13 (dd, *J* = 10.1, 9.0 Hz, 1H), 4.23 – 4.15 (m, 2H), 3.99 (t, *J* = 8.8 Hz, 1H), 3.69 (s, 3H), 2.65 – 2.52 (m, 1H), 2.10 (s, 3H), 1.23 (d, *J* = 6.9 Hz, 3H), 1.20 (d, *J* = 6.9 Hz, 3H), 0.89 (s, 9H), 0.22 (s, 3H), 0.15 (s, 3H); ^{13}C NMR (100 MHz, $CDCl_3$) δ 175.0, 169.8, 167.4, 156.1, 154.2, 151.4, 150.2, 143.3, 141.7 (2C), 129.2 (4C), 126.7 (two peaks overlapped, 6C), 120.6, 84.5, 75.6, 74.5, 72.8, 71.8, 52.8, 36.4, 25.7 (3C), 20.9, 19.3, 18.9, 18.2, -4.1, -4.7; HRMS: m/z (ESI) calcd for $C_{37}H_{47}N_6O_{10}Si^+$, $[M + H]^+$, 763.3117, found 763.3130. $^{1}J^{13}_{C1-H1}$ = 162.0 Hz.

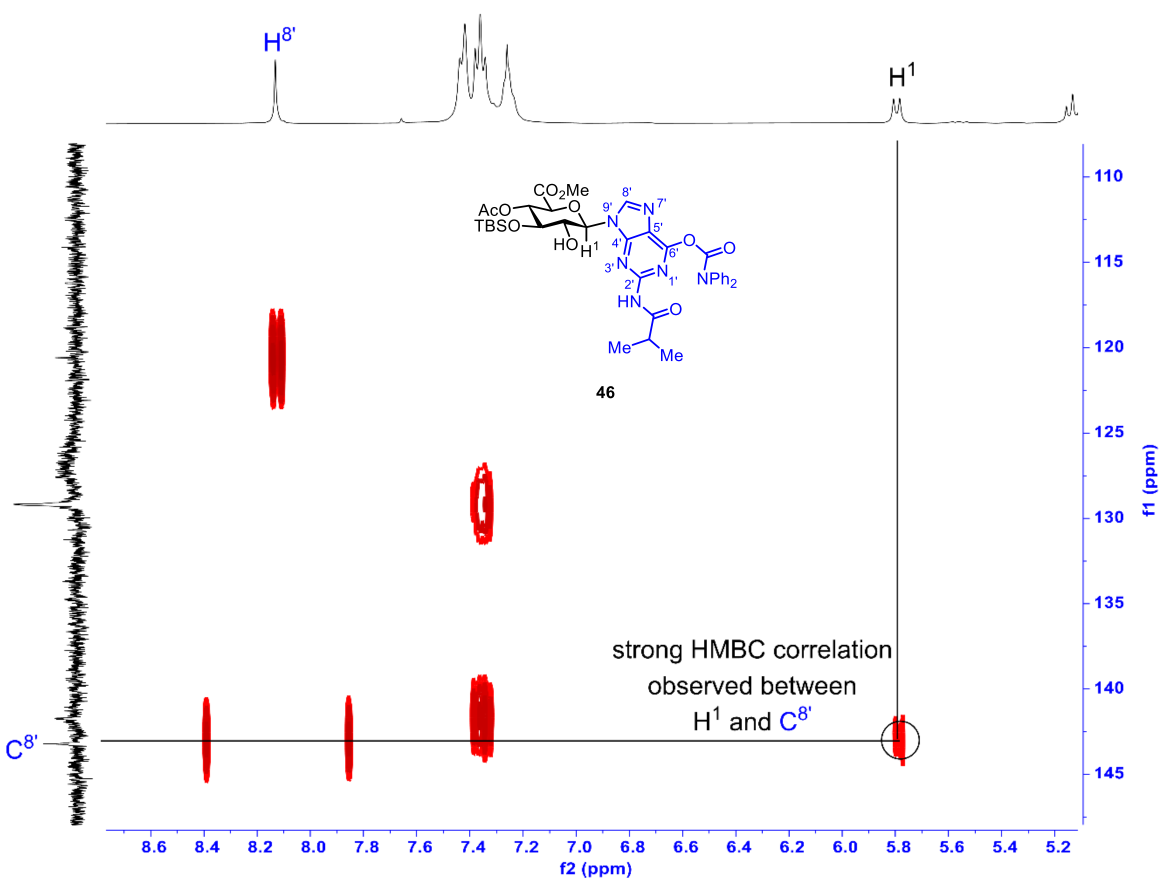

Figure S8. HMBC Analysis to Determine the Structure of **46**.

A strong HMBC correlation between H1 and C8' suggested that guanine **S27** gets glycosylated at the N9 position.

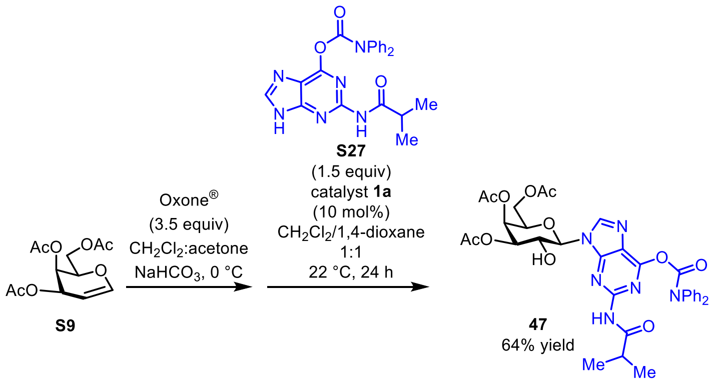

The reaction was carried out on a 0.1 mmol scale by following **Procedure A** for epoxidation and **Procedure D** for *N*-glycosylation. CH_2Cl_2/1,4-dioxane was used as the mixed solvent and the *N*-glycosylation was carried out at 22 °C for 24 h. The *dr* of the corresponding glycal α-epoxide is >20:1. The *dr* of the glycosylation product determined based on crude reaction mixture is >20:1. The desired product **47** (45.1 mg, 64% yield, white foam) was purified through a silica gel flash column (hexanes/acetone: from 20:1 to 1:1).

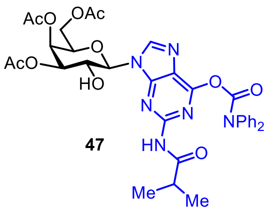

47

6-*O*-Diphenylcarbamoyl-2-*N*-isobutylryl-9-(3,4,6-Tri-*O*-acetyl-β-D-galactopyranosyl)guanine (**47**): IR ν_{max} (neat)/cm^{-1}: 3411 (w), 2977 (w), 1746 (s), 1590 (s), 1450 (m), 1370 (m), 1225 (s), 838 (m); ^1H NMR (400 MHz, $CDCl_3$) δ 8.13 (s, 1H), 7.91 (s, 1H), 7.57 – 7.34 (m, 8H), 7.29 (d, *J* = 7.4 Hz, 2H), 6.37 (d, *J* = 8.7 Hz, 1H), 6.16 (brs, 1H), 6.04 – 5.90 (m, 1H), 5.56 (d, *J* = 3.5 Hz, 1H), 4.40 – 4.32 (m, 1H), 4.30 (t, *J* = 6.4 Hz, 1H), 4.14 – 4.00 (m, 2H), 2.41 – 2.30 (m, 1H), 2.25 (s, 3H), 2.11 (s, 3H), 2.02 (s, 3H), 1.14 (d, *J* = 6.7 Hz, 3H), 1.02 (d, *J* = 7.1 Hz, 3H); ^{13}C NMR (100 MHz, $CDCl_3$) δ 175.2, 170.5, 170.1, 170.0, 155.7, 154.8, 150.9, 150.4, 142.8, 141.8 (2C), 129.2 (4C), 127.3 (two peaks overlapped, 6C), 120.6, 83.3, 73.0,

71.8, 70.4, 68.5, 61.9, 36.4, 21.0, 20.9, 20.7, 19.7, 18.4; HRMS: m/z (ESI) calcd for $C_{34}H_{37}N_6O_{11}^+$, [M + H]$^+$, 705.2515, found 705.2531. $^{1}J^{13}_{C1-H1}$ = 163.4 Hz.

E. References

1. Zhang, X.-W.; Yin, L.; Zhang, D.; Jiang, Z.; Wang, P.; Xu, H. Iron-Catalyzed Highly Stereospecific Glycosylation with Glycal Epoxides. *Angew. Chem. Int. Ed.* **2025**, *64*, e202517634.
2. Eby, R.; Srivastava, V. K. Conformational Analysis of 1,2-Anhydro-3,4,6-tri-*O*-Benzyl-α-D-Glucopyranose and -β-D-Mannopyranose. *Carbohydr. Res.* **1982**, *102*, 1.
3. Taber, D. F.; DeMatteo, P. W.; Hassan, R. A. Simplified Preparation of Dimethyldioxirane (DMDO). In *Organic Syntheses*, pp 350-357.
4. Schell, P.; Orgueira, H. A.; Roehrig, S.; Seeberger, P. H. Synthesis and Transformations of D-Glucuronic and L-Iduronic Acid Glycals. *Tetrahedron Lett.* **2001**, *42*, 3811.
5. Li, H.; Zhang, D.; Li, C.; Yin, L.; Jiang, Z.; Luo, Y.; Xu, H. Stereoselective Glycosylation for 1,2-*cis*-Aminoglycoside Assembly by Cooperative Atom Transfer Catalysis. *J. Am. Chem. Soc.* **2024**, *146*, 33316.
6. Yin, L.; Zhang, D.; Jiang, Z.; Xu, H. Stereoselective Multigram-Scale Tn Antigen Synthesis via the Iron-Catalyzed Glycal 1, 2-cis-Aminoglycosylation. *Org. Lett.* **2025**, *27*, 5515.
7. Brik, A.; Yang, Y.-Y.; Ficht, S.; Wong, C.-H. Sugar-Assisted Glycopeptide Ligation. *J. Am. Chem. Soc.* **2006**, *128*, 5626.
8. Ruhela, D.; Chatterjee, P.; Vishwakarma, R. A. 1-Oxabicyclic β-Lactams as New Inhibitors of Elongating MPT–a Key Enzyme Responsible for Assembly of Cell-Surface Phosphoglycans of Leishmania Parasite. *Org. Biomol. Chem.* **2005**, *3*, 1043.
9. Liu, L.; Ji, X.; Li, Y.; Ji, W.; Mo, T.; Ding, W.; Zhang, Q. A Mechanistic Study of the Non-Oxidative Decarboxylation Catalyzed by the Radical *S*-Adenosyl-L-Methionine Enzyme BlsE Involved in Blasticidin S Biosynthesis. *Chem. Commun.* **2017**, *53*, 8952.
10. Dey, S.; Garner, P. Synthesis of *tert*-Butoxycarbonyl (Boc)-Protected Purines. *J. Org. Chem.* **2000**, *65*, 7697.
11. Robins, M. J.; Zou, R.; Guo, Z.; Wnuk, S. F. Nucleic Acid Related Compounds. 93. A Solution for the Historic Problem of Regioselective Sugar−Base Coupling To Produce 9-Glycosylguanines or 7-Glycosylguanines1. *J. Org. Chem.* **1996**, *61*, 9207.

F. NMR Spectra

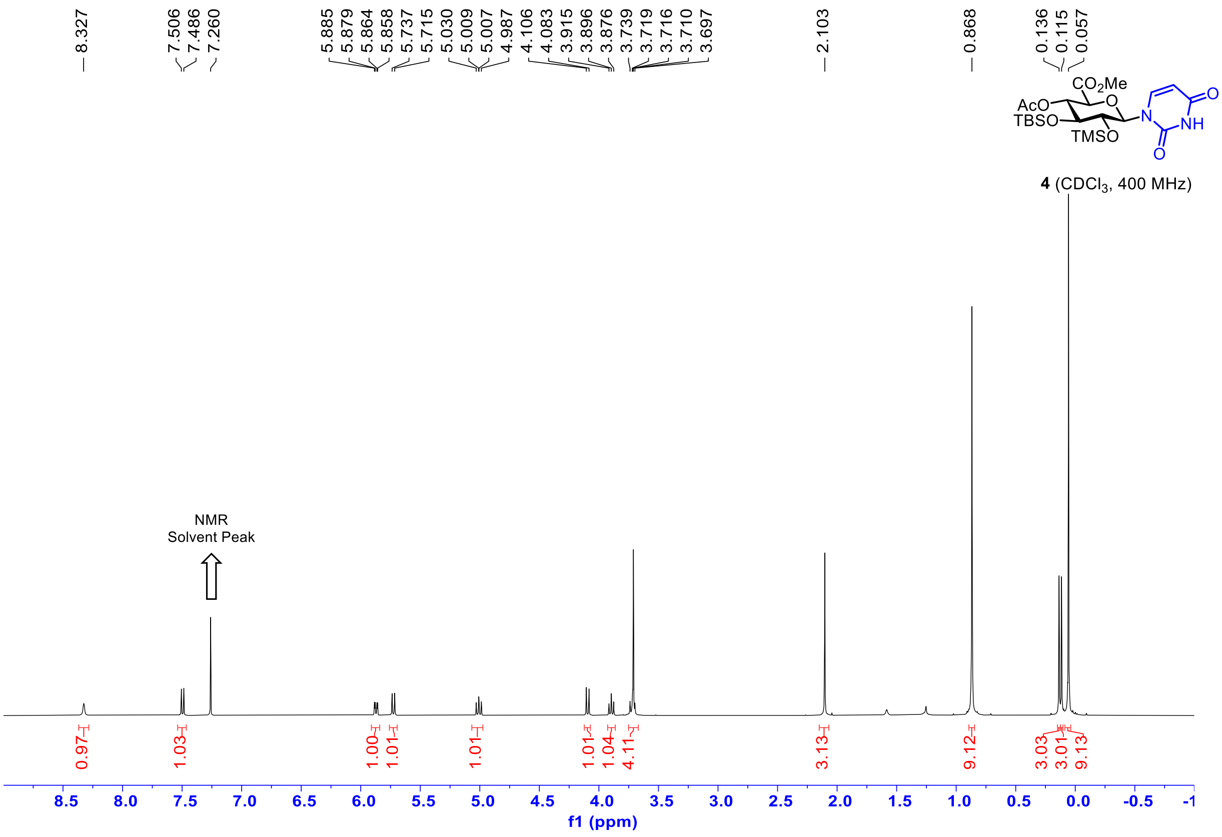

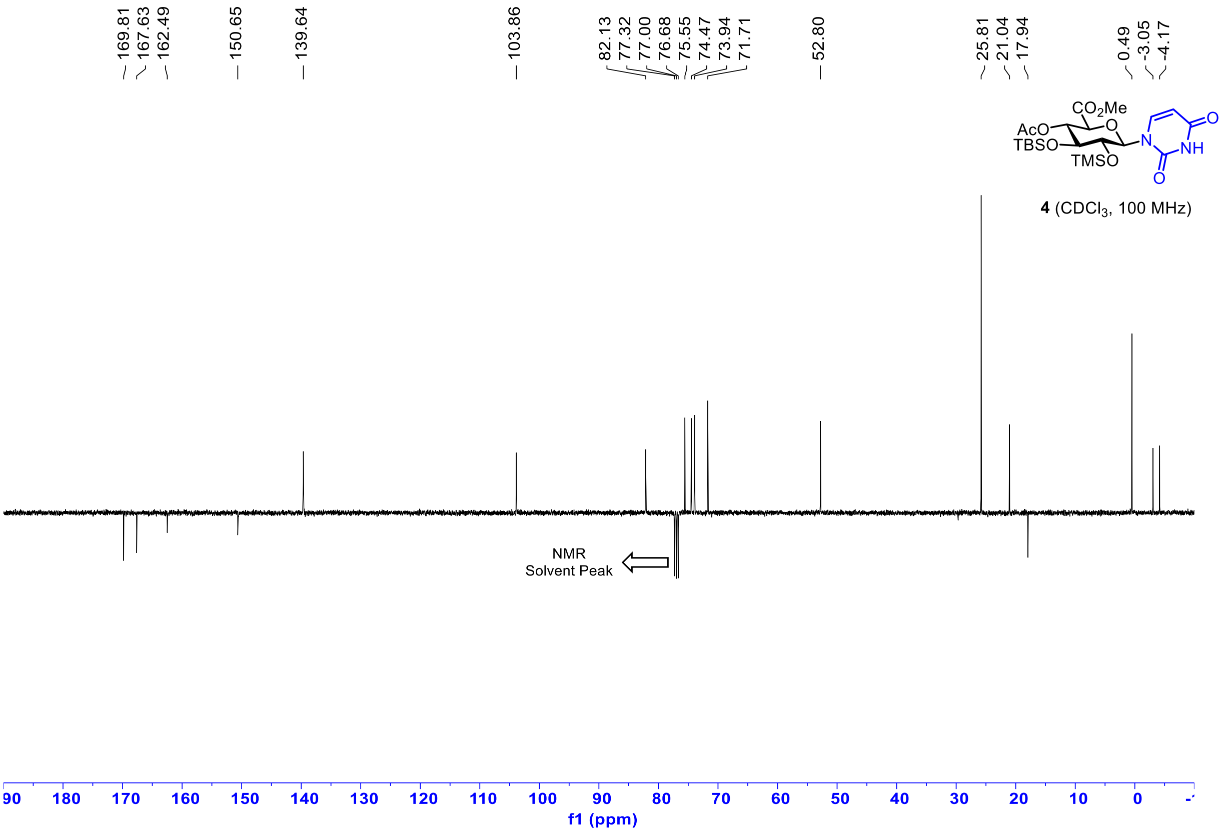

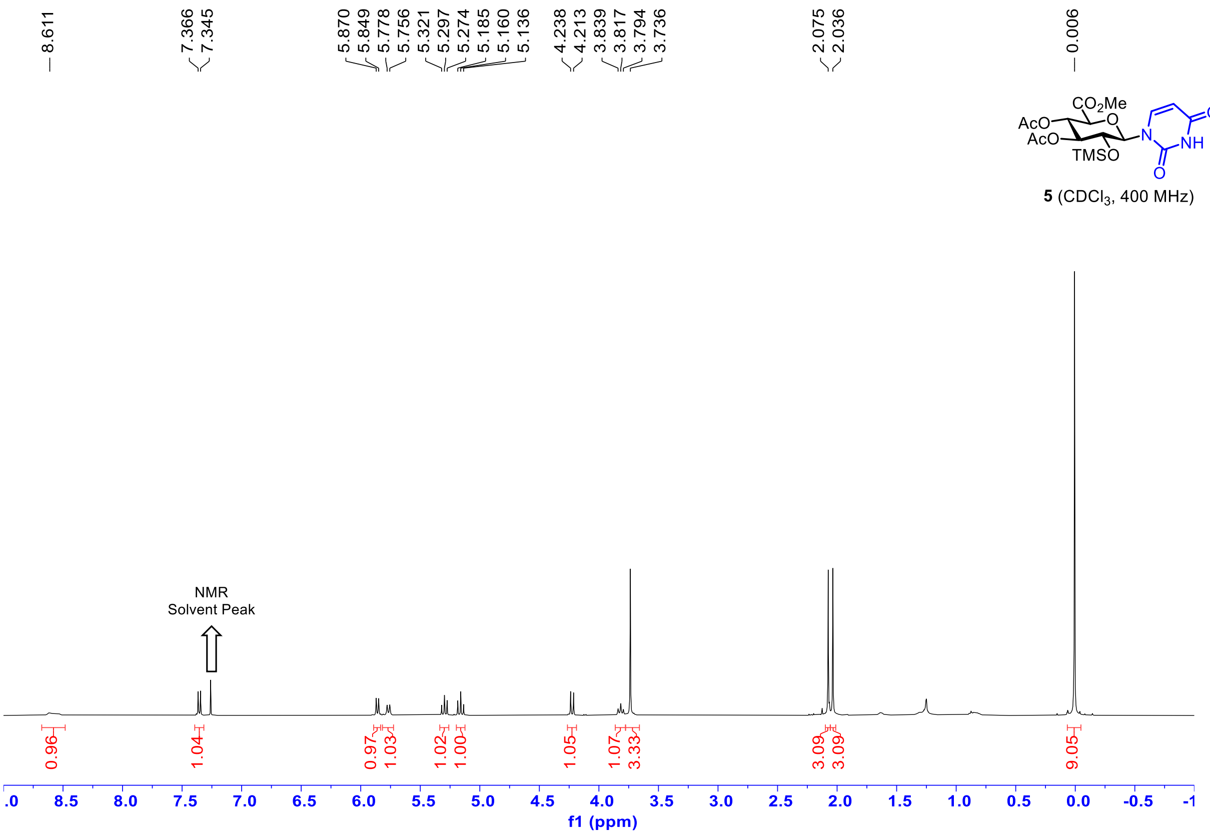
8.611
7.366
7.345
5.870
5.849
5.778
5.756
5.321
5.297
5.274
5.185
5.160
5.136
4.238
4.213
3.839
3.817
3.794
3.736
2.075
2.036
0.006
AcO
AcO
CO2Me
O
TMSO
N
NH
O
O
5 (CDCl3, 400 MHz)
NMR
Solvent Peak
0.96
1.04
0.97
1.03
1.02
1.00
1.05
1.07
3.33
3.09
3.09
9.05
f1 (ppm)

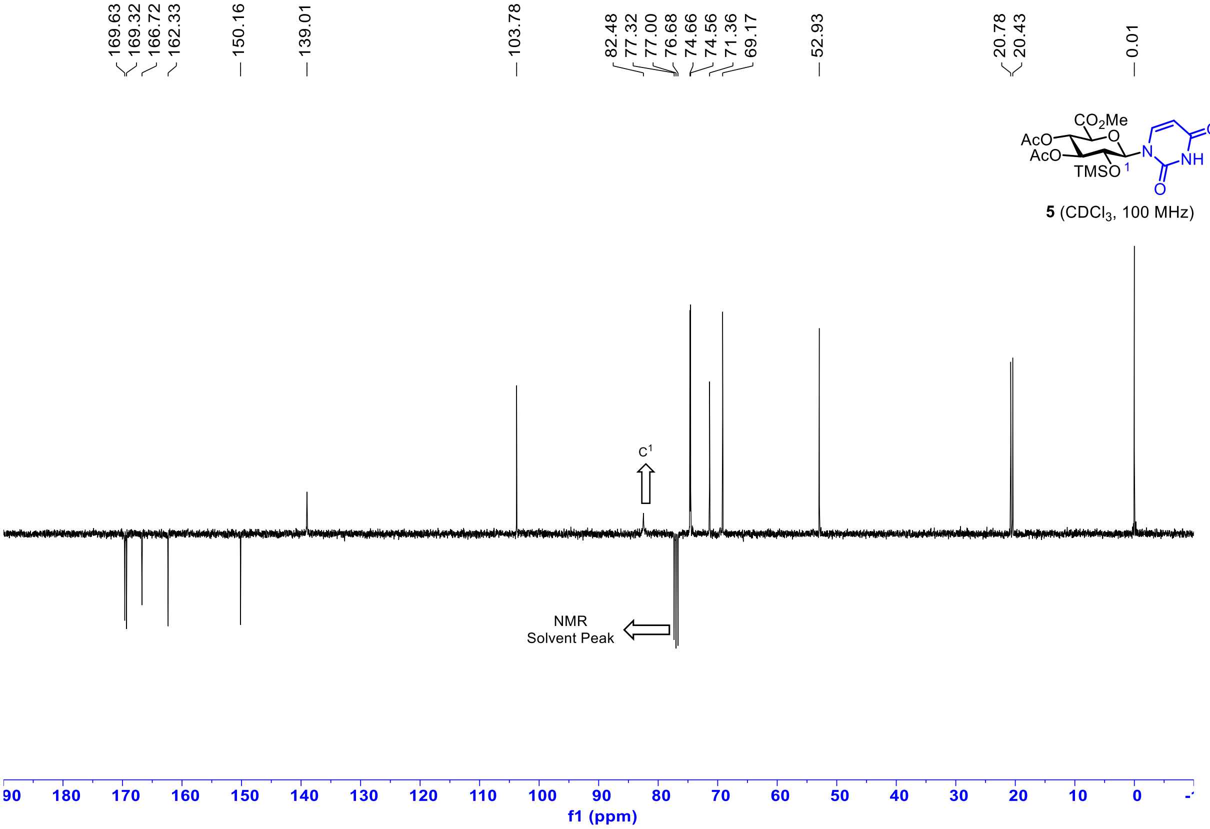
169.63
169.32
166.72
162.33
150.16
139.01
103.78
82.48
77.32
77.00
76.68
74.66
74.56
71.36
69.17
52.93
20.78
20.43
0.01
AcO
AcO
CO2Me
O
TMSO
1
N
NH
O
O
5 (CDCl3, 100 MHz)
C1
NMR
Solvent Peak
f1 (ppm)

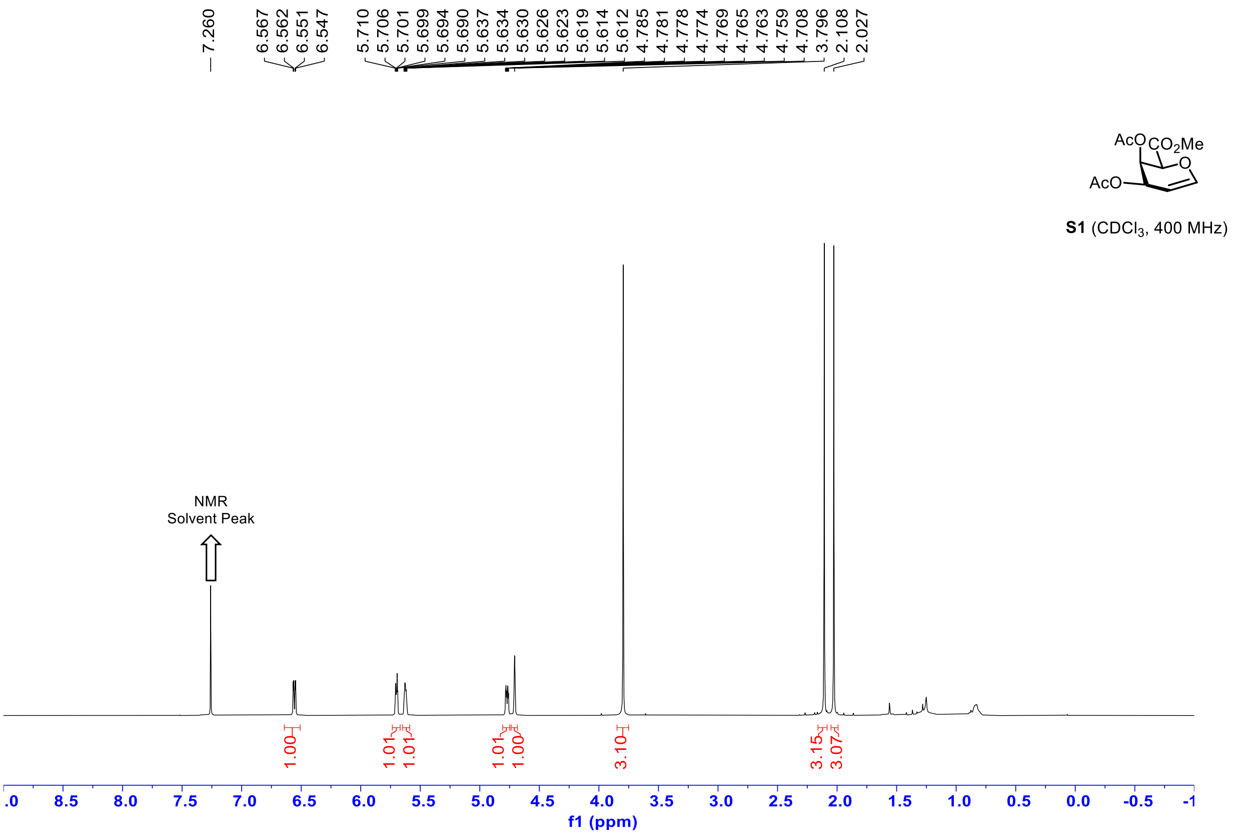

7.260
6.567
6.562
6.551
6.547
5.710
5.706
5.701
5.699
5.694
5.690
5.637
5.634
5.630
5.626
5.623
5.619
5.614
5.612
4.785
4.781
4.778
4.774
4.769
4.765
4.763
4.759
4.708
3.796
2.108
2.027
AcO
CO_2Me
O
AcO
S1 ($CDCl_3$, 400 MHz)
NMR
Solvent Peak
1.00
1.01
1.01
1.01
1.00
3.10
3.15
3.07
f1 (ppm)

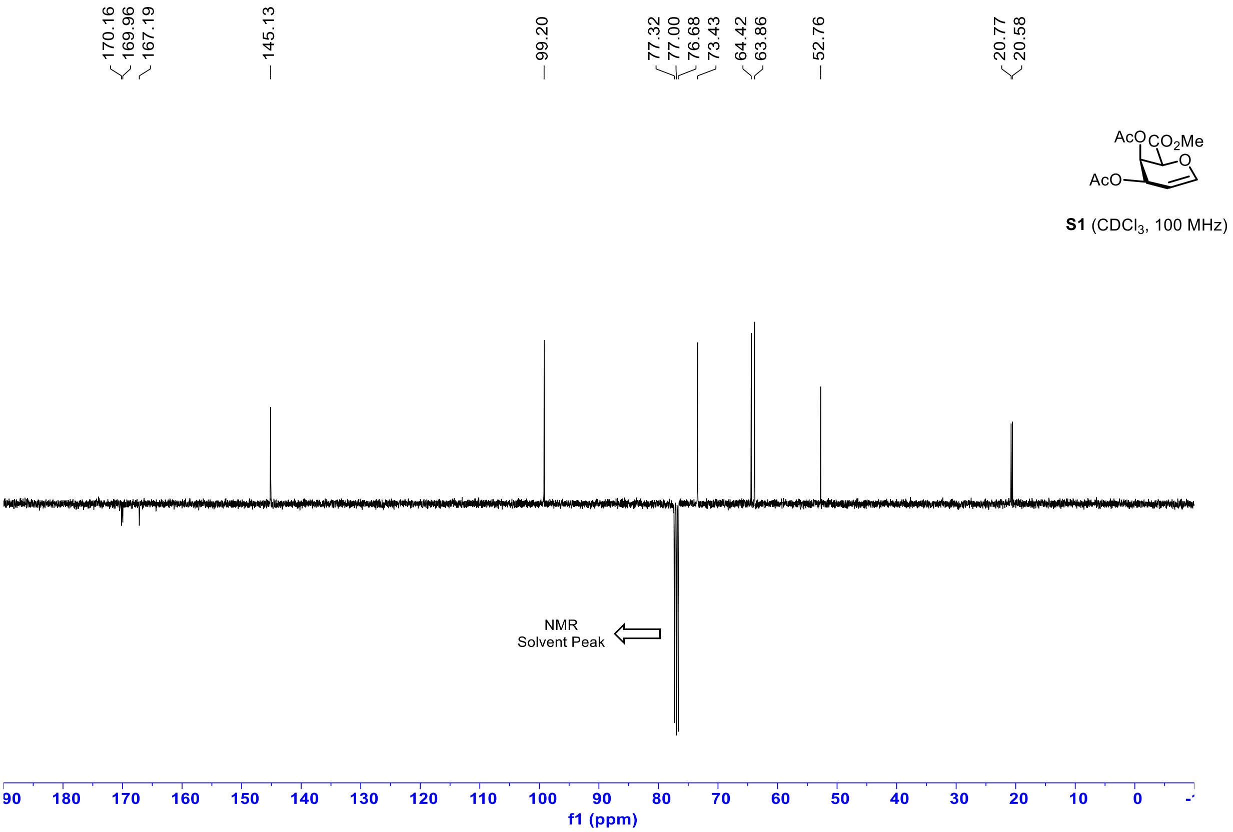

170.16
169.96
167.19
145.13
99.20
77.32
77.00
76.68
73.43
64.42
63.86
52.76
20.77
20.58
AcO
CO_2Me
O
AcO
S1 ($CDCl_3$, 100 MHz)
NMR
Solvent Peak
f1 (ppm)

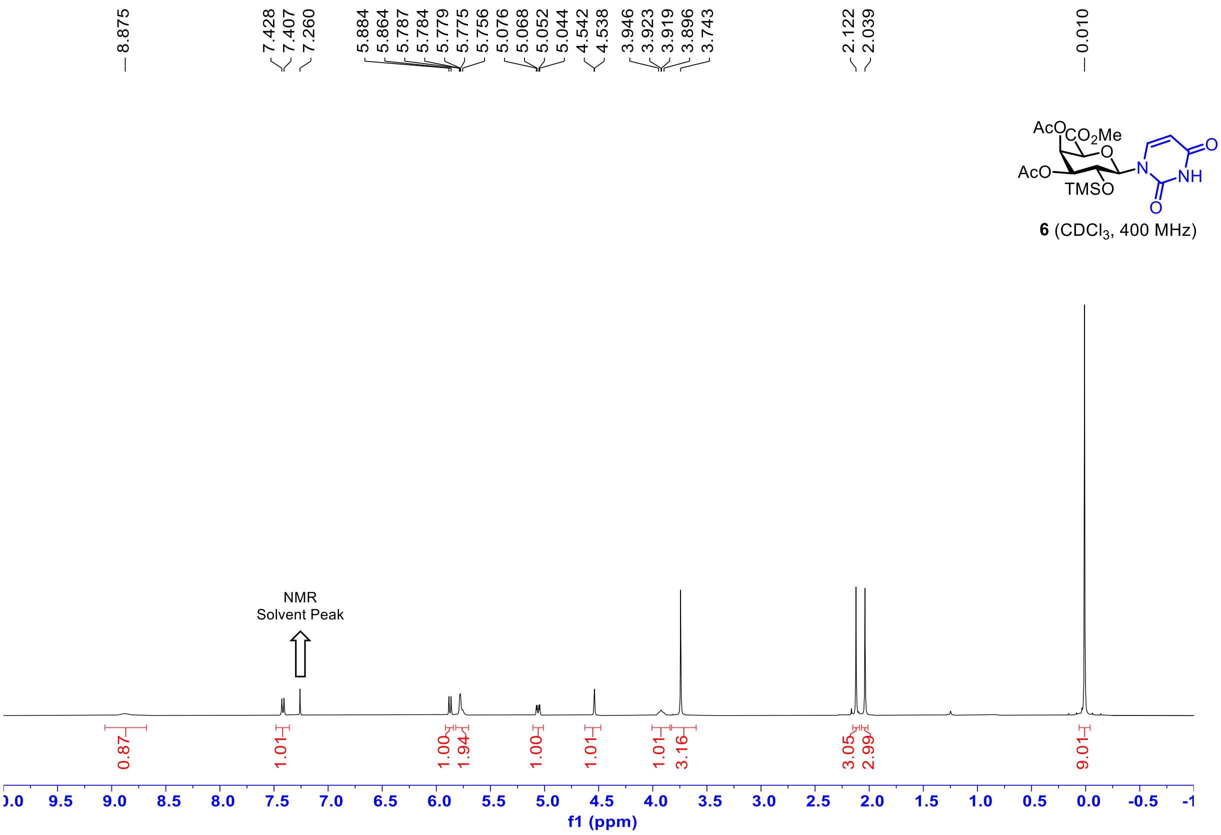
8.875
7.428
7.407
7.260
5.884
5.864
5.787
5.784
5.779
5.775
5.756
5.076
5.068
5.052
5.044
4.542
4.538
3.946
3.923
3.919
3.896
3.743
2.122
2.039
0.010
AcO
CO2Me
O
N
O
NH
AcO
TMSO
O
6 (CDCl3, 400 MHz)
NMR
Solvent Peak
0.87
1.01
1.00
1.94
1.00
1.01
1.01
3.16
3.05
2.99
9.01
f1 (ppm)

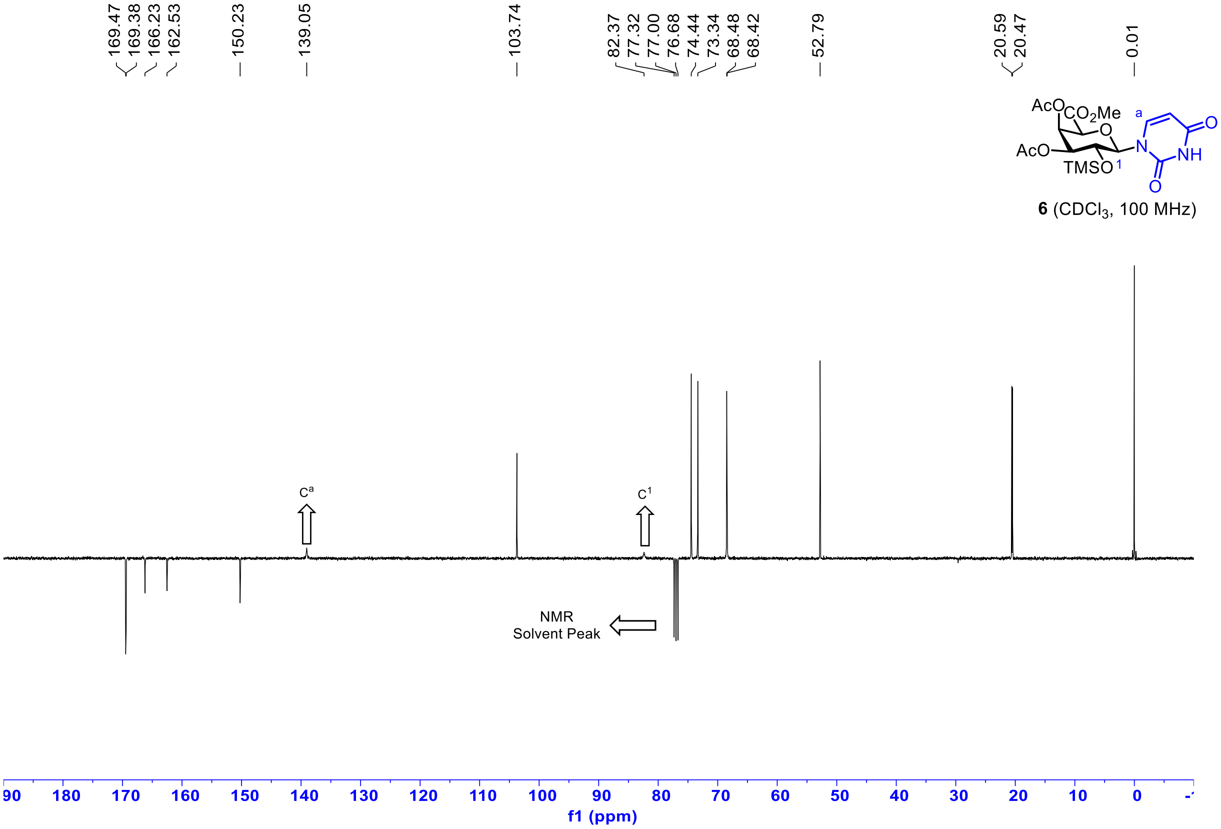
169.47
169.38
166.23
162.53
150.23
139.05
103.74
82.37
77.32
77.00
76.68
74.44
73.34
68.48
68.42
52.79
20.59
20.47
0.01
AcO
CO2Me
a
O
N
O
NH
AcO
TMSO
1
O
6 (CDCl3, 100 MHz)
Ca
C1
NMR
Solvent Peak
f1 (ppm)

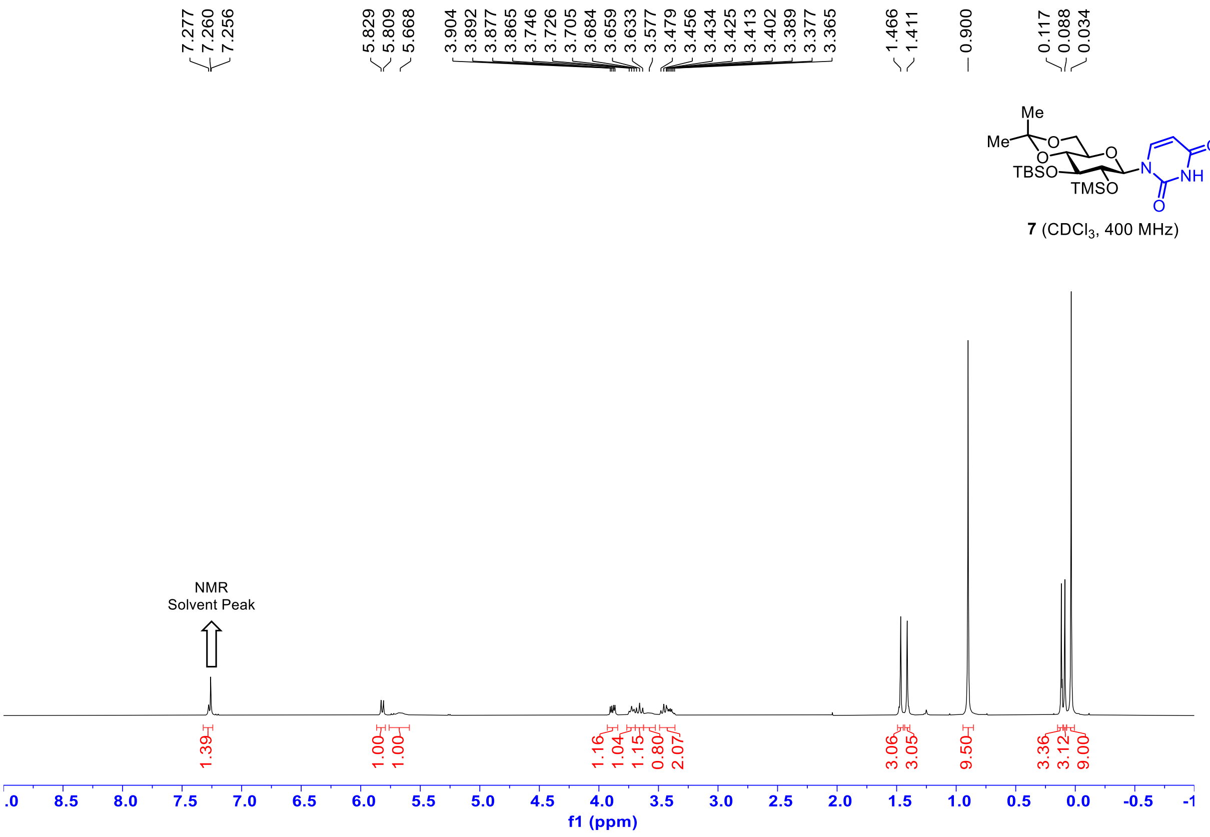

7 (CDCl$_3$, 400 MHz)

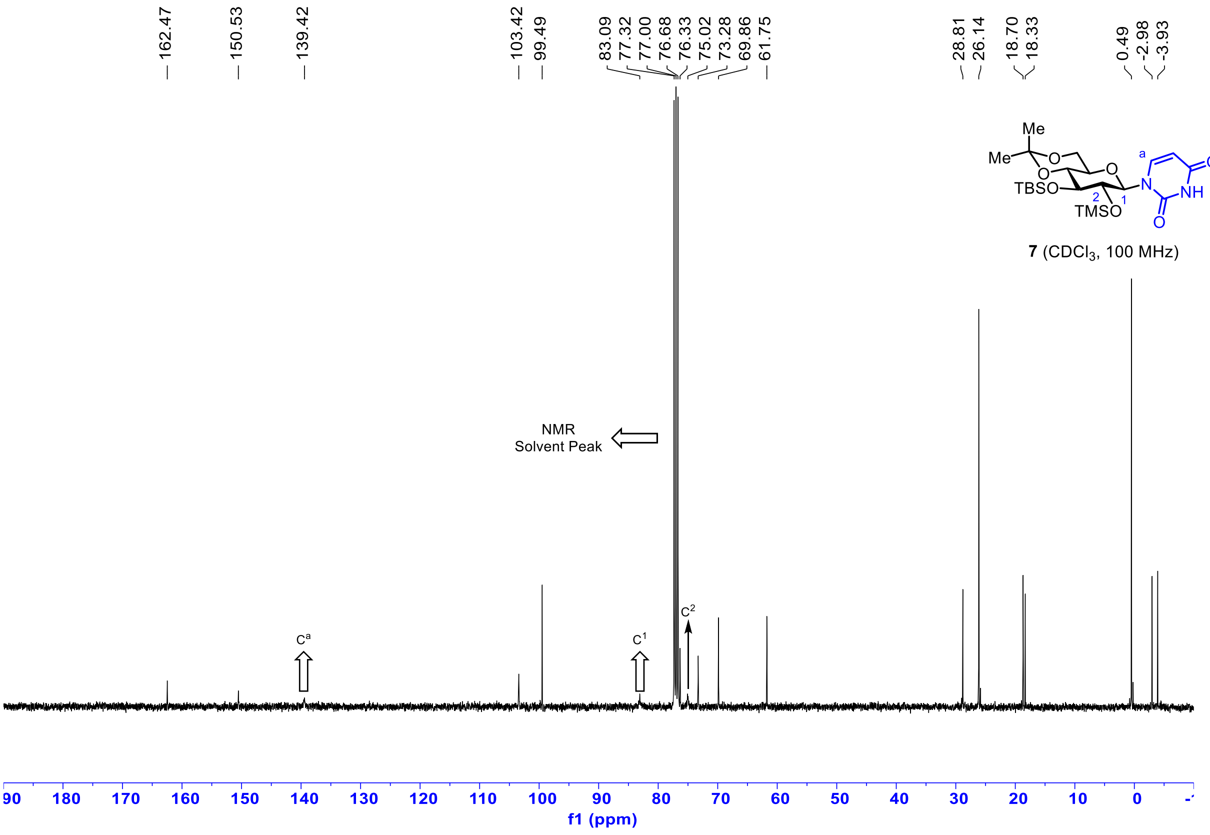

7 (CDCl$_3$, 100 MHz)

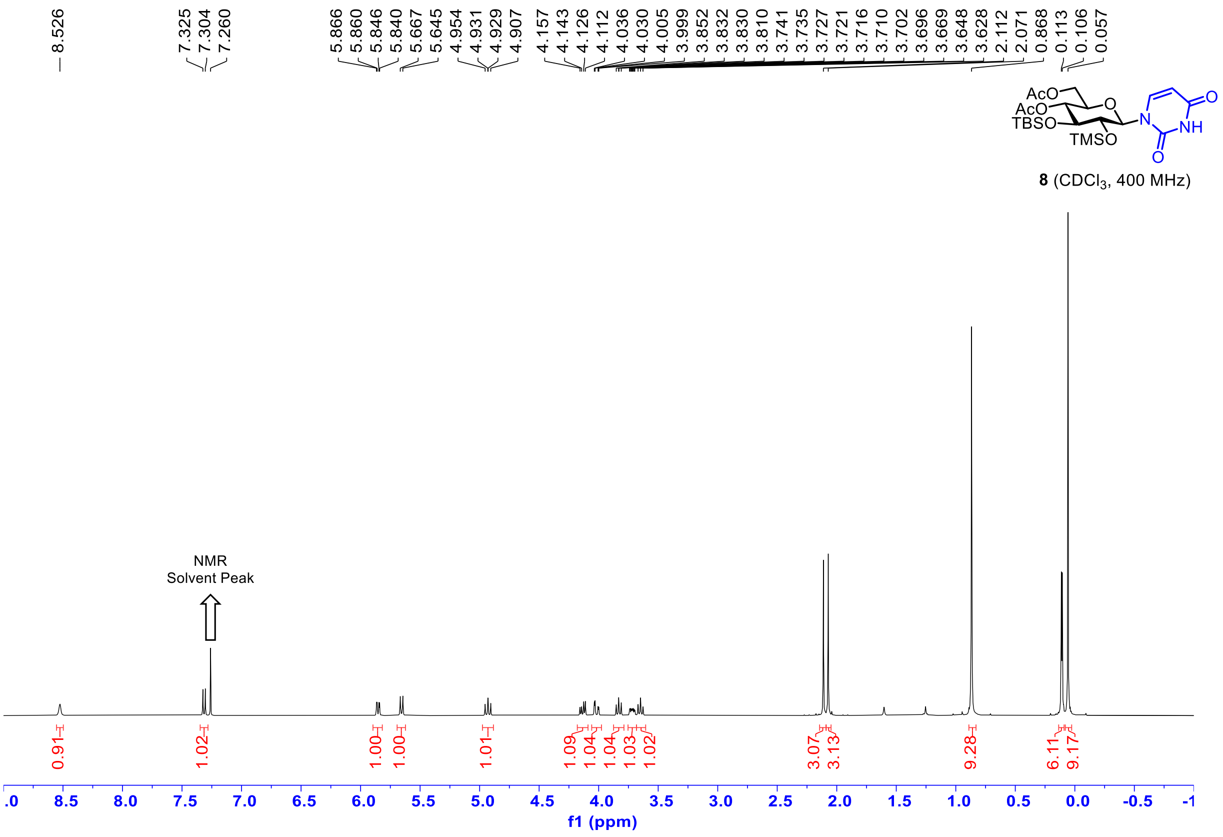
8.526
7.325
7.304
7.260
5.866
5.860
5.846
5.840
5.667
5.645
4.954
4.931
4.929
4.907
4.157
4.143
4.126
4.112
4.036
4.030
4.005
3.999
3.852
3.832
3.830
3.810
3.741
3.735
3.727
3.721
3.716
3.710
3.702
3.696
3.669
3.648
3.628
2.112
2.071
0.868
0.113
0.106
0.057
AcO
AcO
TBSO
TMSO
O
N
NH
O
O
8 (CDCl3, 400 MHz)
NMR
Solvent Peak
0.91
1.02
1.00
1.00
1.01
1.09
1.04
1.04
1.03
1.02
3.07
3.13
9.28
6.11
9.17
f1 (ppm)

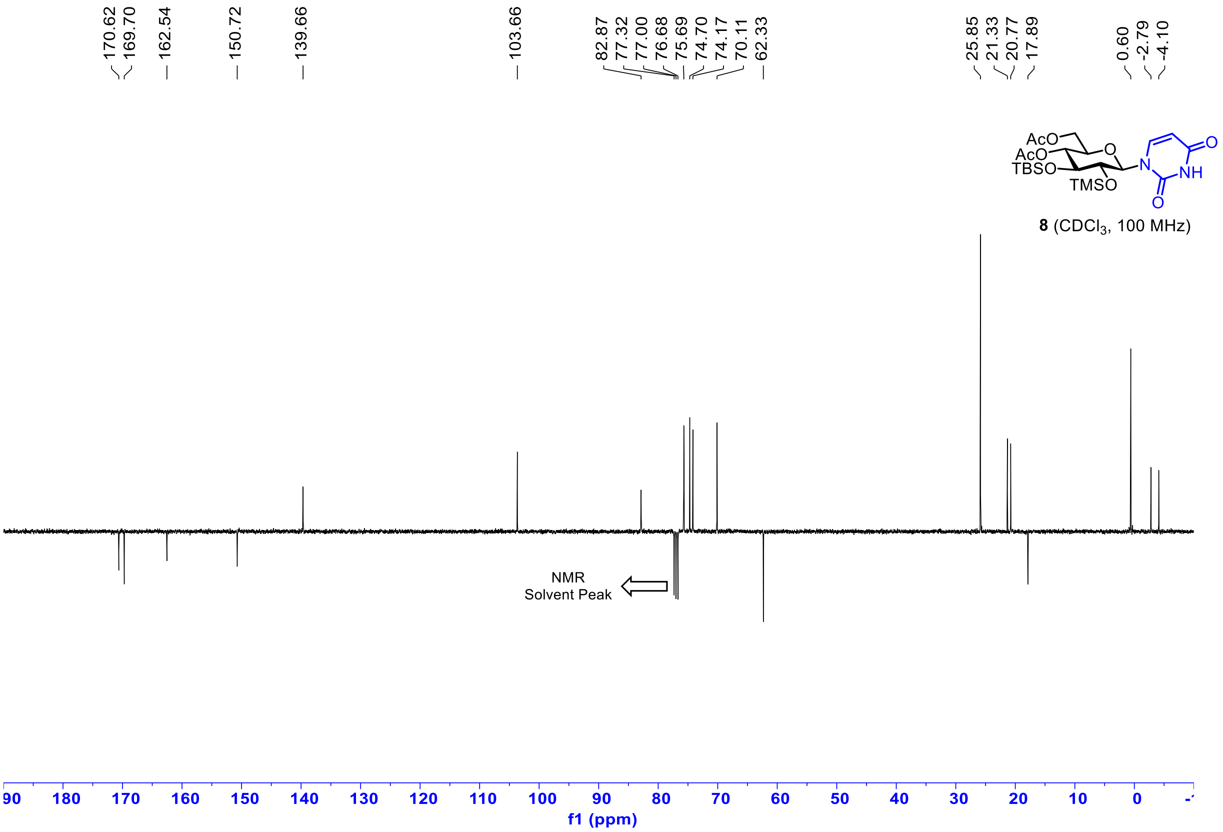
170.62
169.70
162.54
150.72
139.66
103.66
82.87
77.32
77.00
76.68
75.69
74.70
74.17
70.11
62.33
25.85
21.33
20.77
17.89
0.60
-2.79
-4.10
AcO
AcO
TBSO
TMSO
O
N
NH
O
O
8 (CDCl3, 100 MHz)
NMR
Solvent Peak
f1 (ppm)

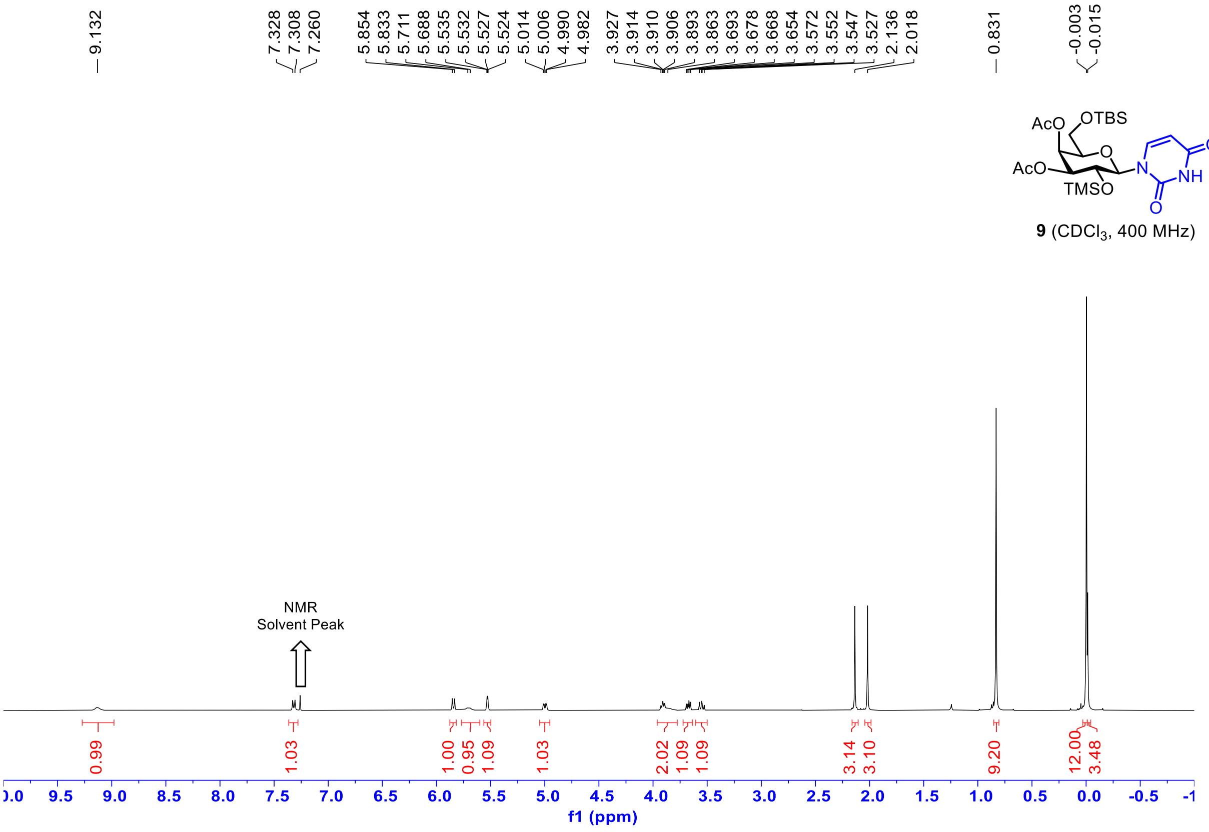
9.132
7.328
7.308
7.260
5.854
5.833
5.711
5.688
5.535
5.532
5.527
5.524
5.014
5.006
4.990
4.982
3.927
3.914
3.910
3.906
3.893
3.863
3.693
3.678
3.668
3.654
3.572
3.552
3.547
3.527
2.136
2.018
0.831
-0.003
-0.015
AcO
OTBS
O
N
O
NH
O
AcO
TMSO
9 (CDCl3, 400 MHz)
NMR
Solvent Peak
0.99
1.03
1.00
0.95
1.09
1.03
2.02
1.09
1.09
3.14
3.10
9.20
12.00
3.48
f1 (ppm)

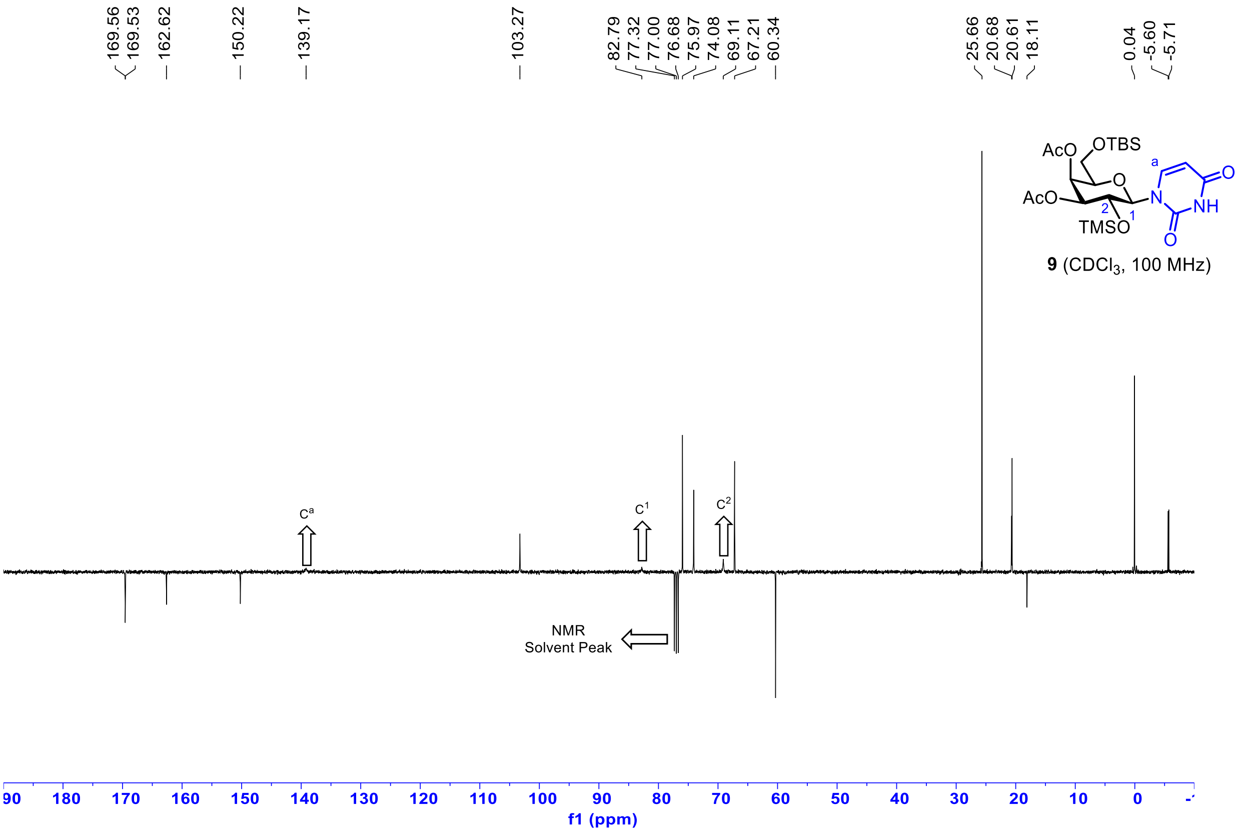
169.56
169.53
162.62
150.22
139.17
103.27
82.79
77.32
77.00
76.68
75.97
74.08
69.11
67.21
60.34
25.66
20.68
20.61
18.11
0.04
-5.60
-5.71
AcO
OTBS
a
O
N
O
NH
O
AcO
2
1
TMSO
9 (CDCl3, 100 MHz)
Ca
C1
C2
NMR
Solvent Peak
f1 (ppm)

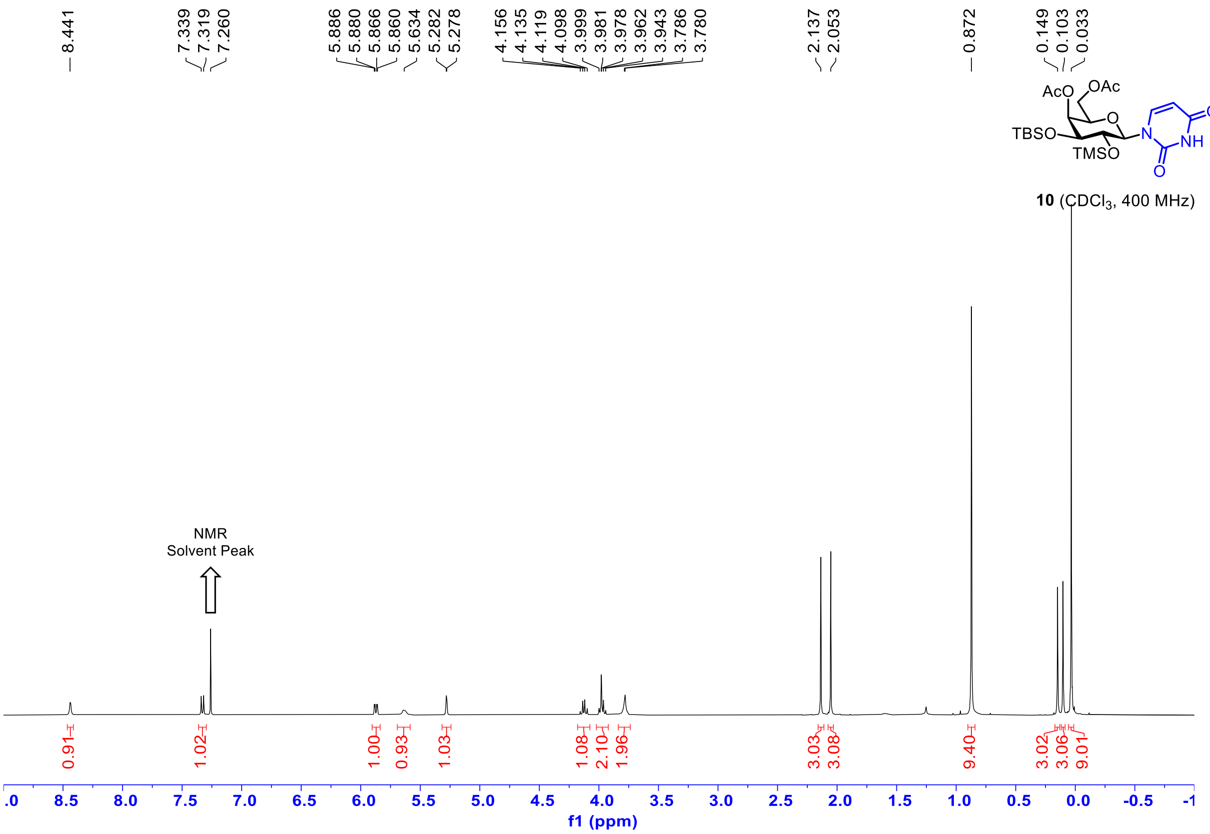
8.441
7.339
7.319
7.260
5.886
5.880
5.866
5.860
5.634
5.282
5.278
4.156
4.135
4.119
4.098
3.999
3.981
3.978
3.962
3.943
3.786
3.780
2.137
2.053
0.872
0.149
0.103
0.033
AcO
OAc
TBSO
TMSO
O
N
NH
10 (CDCl3, 400 MHz)
NMR
Solvent Peak
0.91
1.02
1.00
0.93
1.03
1.08
2.10
1.96
3.03
3.08
9.40
3.02
3.06
9.01
f1 (ppm)

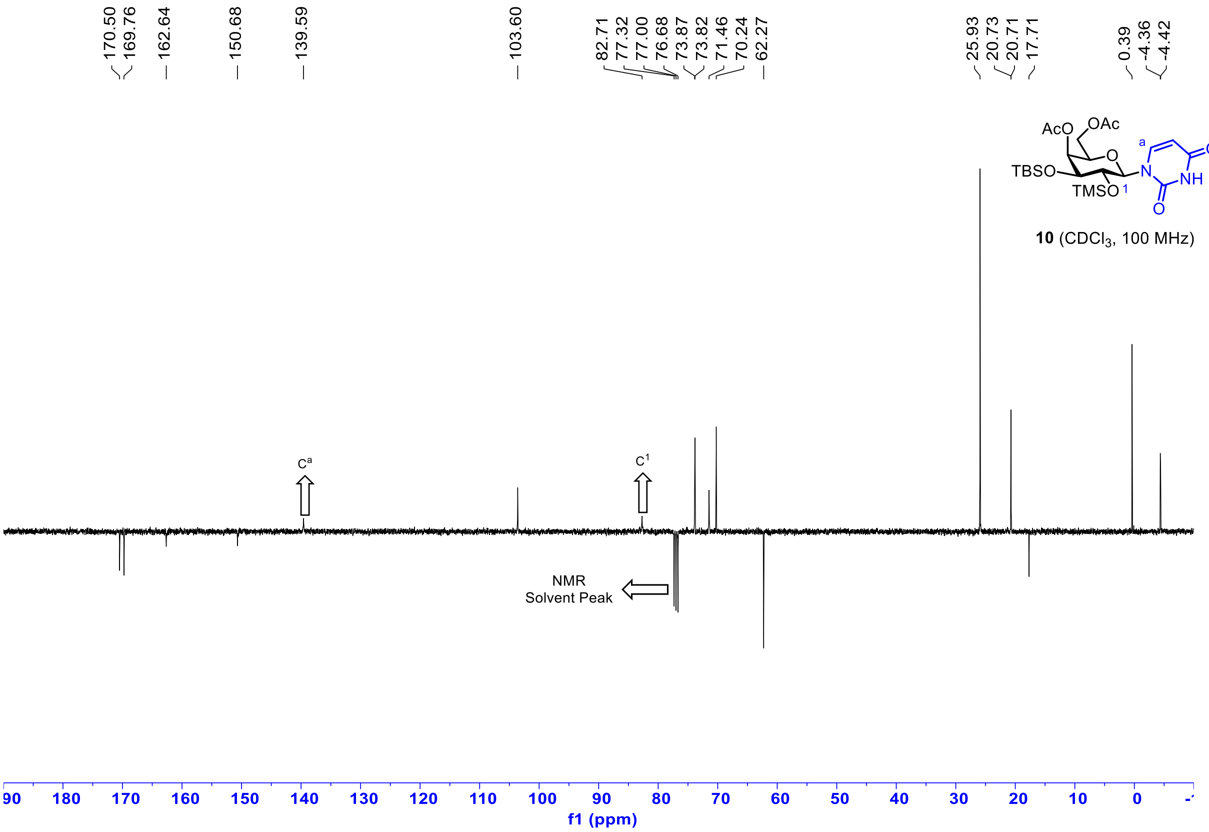
170.50
169.76
162.64
150.68
139.59
103.60
82.71
77.32
77.00
76.68
73.87
73.82
71.46
70.24
62.27
25.93
20.73
20.71
17.71
0.39
-4.36
-4.42
AcO
OAc
TBSO
TMSO
a
1
10 (CDCl3, 100 MHz)
Ca
C1
NMR
Solvent Peak
f1 (ppm)

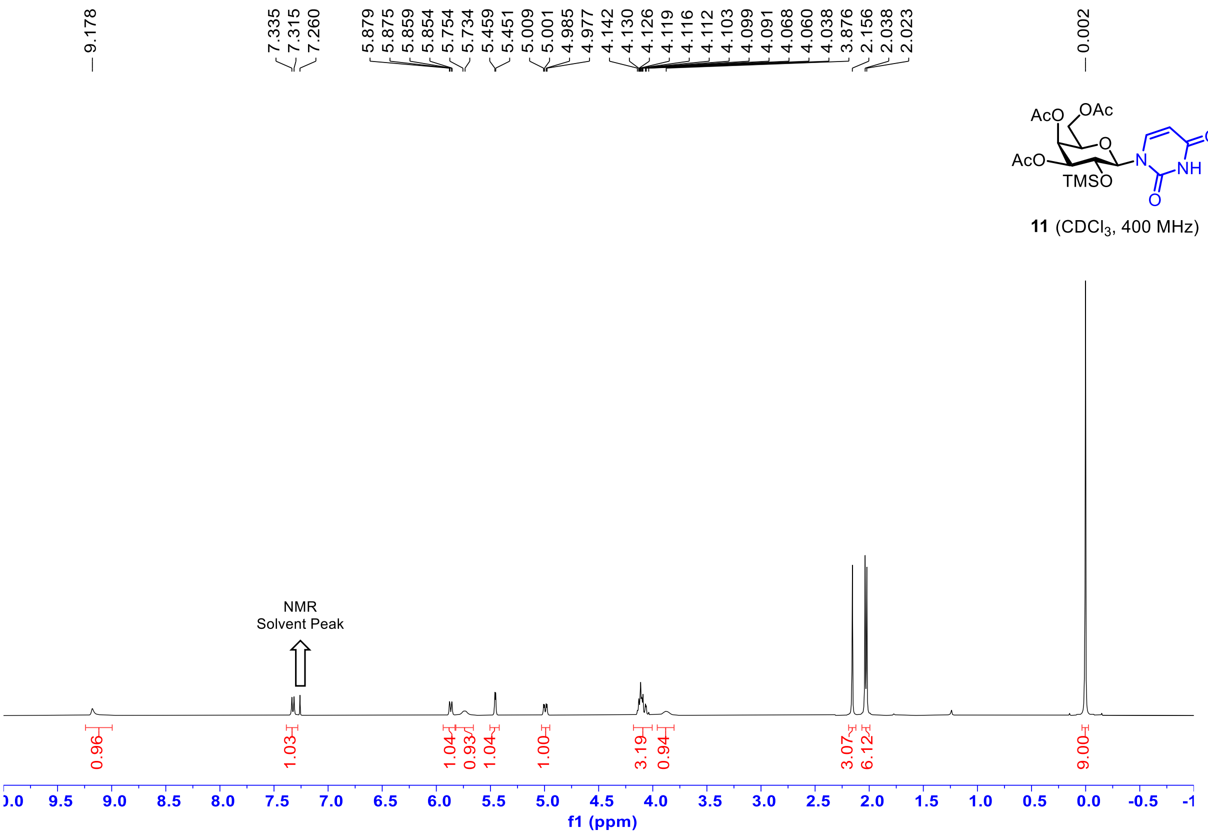
9.178
7.335
7.315
7.260
5.879
5.875
5.859
5.854
5.754
5.734
5.459
5.451
5.009
5.001
4.985
4.977
4.142
4.130
4.126
4.119
4.116
4.112
4.103
4.099
4.091
4.068
4.060
4.038
3.876
2.156
2.038
2.023
0.002
AcO
OAc
AcO
TMSO
O
N
NH
O
O
11 (CDCl3, 400 MHz)
NMR
Solvent Peak
0.96
1.03
1.04
0.93
1.04
1.00
3.19
0.94
3.07
6.12
9.00
f1 (ppm)

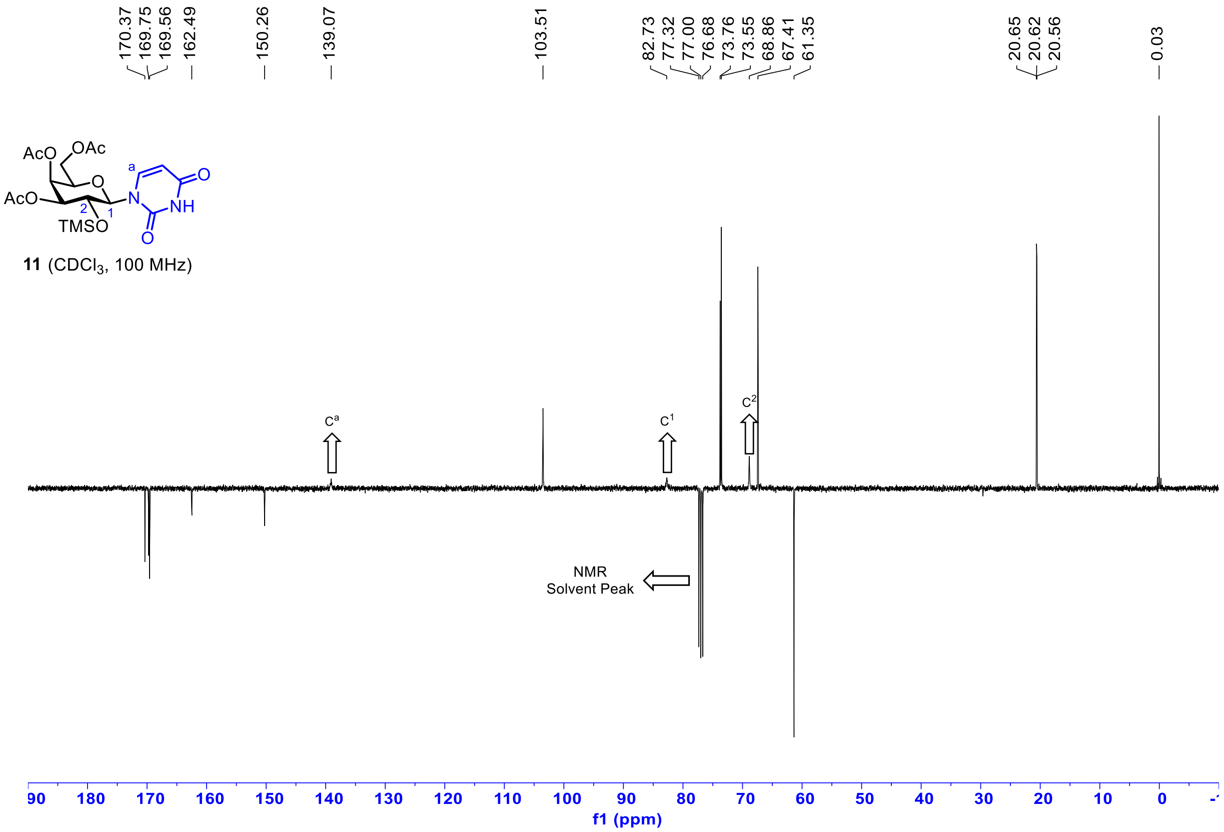
170.37
169.75
169.56
162.49
150.26
139.07
103.51
82.73
77.32
77.00
76.68
73.76
73.55
68.86
67.41
61.35
20.65
20.62
20.56
0.03
AcO
OAc
AcO
TMSO
a
2
1
N
NH
O
O
11 (CDCl3, 100 MHz)
Ca
C1
C2
NMR
Solvent Peak
f1 (ppm)

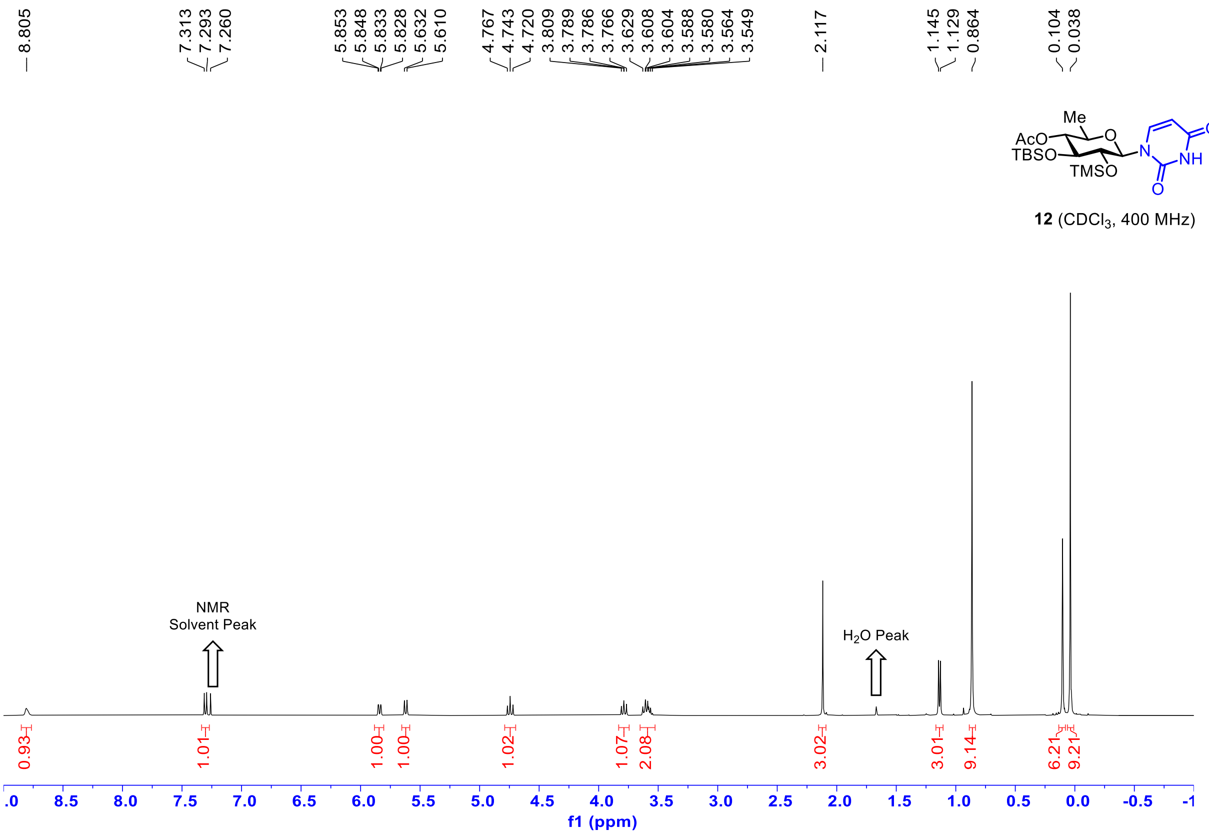
8.805
7.313
7.293
7.260
5.853
5.848
5.833
5.828
5.632
5.610
4.767
4.743
4.720
3.809
3.789
3.786
3.766
3.629
3.608
3.604
3.588
3.580
3.564
3.549
2.117
1.145
1.129
0.864
0.104
0.038
Me
AcO
TBSO
TMSO
O
N
O
NH
O
12 (CDCl3, 400 MHz)
NMR
Solvent Peak
H2O Peak
0.93
1.01
1.00
1.00
1.02
1.07
2.08
3.02
3.01
9.14
6.21
9.21
f1 (ppm)

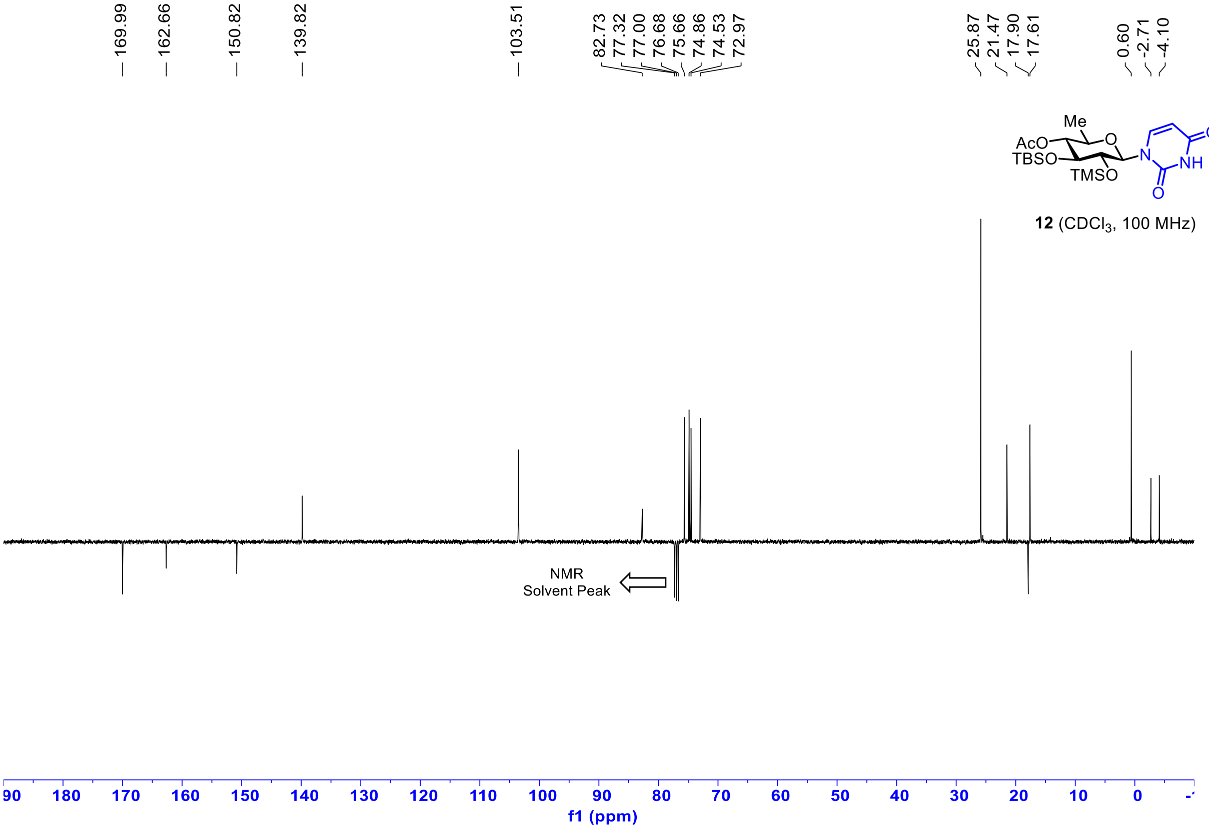
169.99
162.66
150.82
139.82
103.51
82.73
77.32
77.00
76.68
75.66
74.86
74.53
72.97
25.87
21.47
17.90
17.61
0.60
-2.71
-4.10
Me
AcO
TBSO
TMSO
O
N
O
NH
O
12 (CDCl3, 100 MHz)
NMR
Solvent Peak
f1 (ppm)

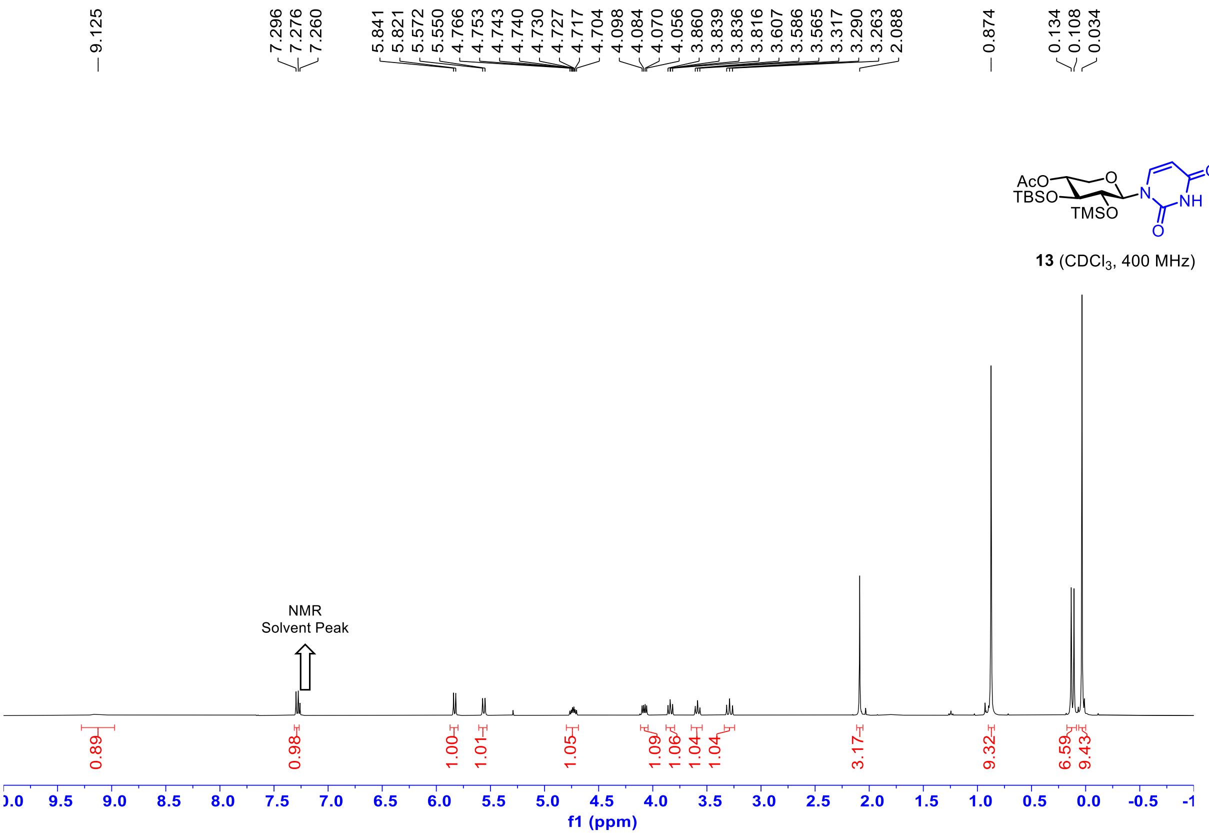
9.125
7.296
7.276
7.260
5.841
5.821
5.572
5.550
4.766
4.753
4.743
4.740
4.730
4.727
4.717
4.704
4.098
4.084
4.070
4.056
3.860
3.839
3.836
3.816
3.607
3.586
3.565
3.317
3.290
3.263
2.088
0.874
0.134
0.108
0.034
AcO
TBSO
TMSO
O
N
NH
O
O
13 (CDCl3, 400 MHz)
NMR
Solvent Peak
0.89
0.98
1.00
1.01
1.05
1.09
1.06
1.04
1.04
3.17
9.32
6.59
9.43
f1 (ppm)

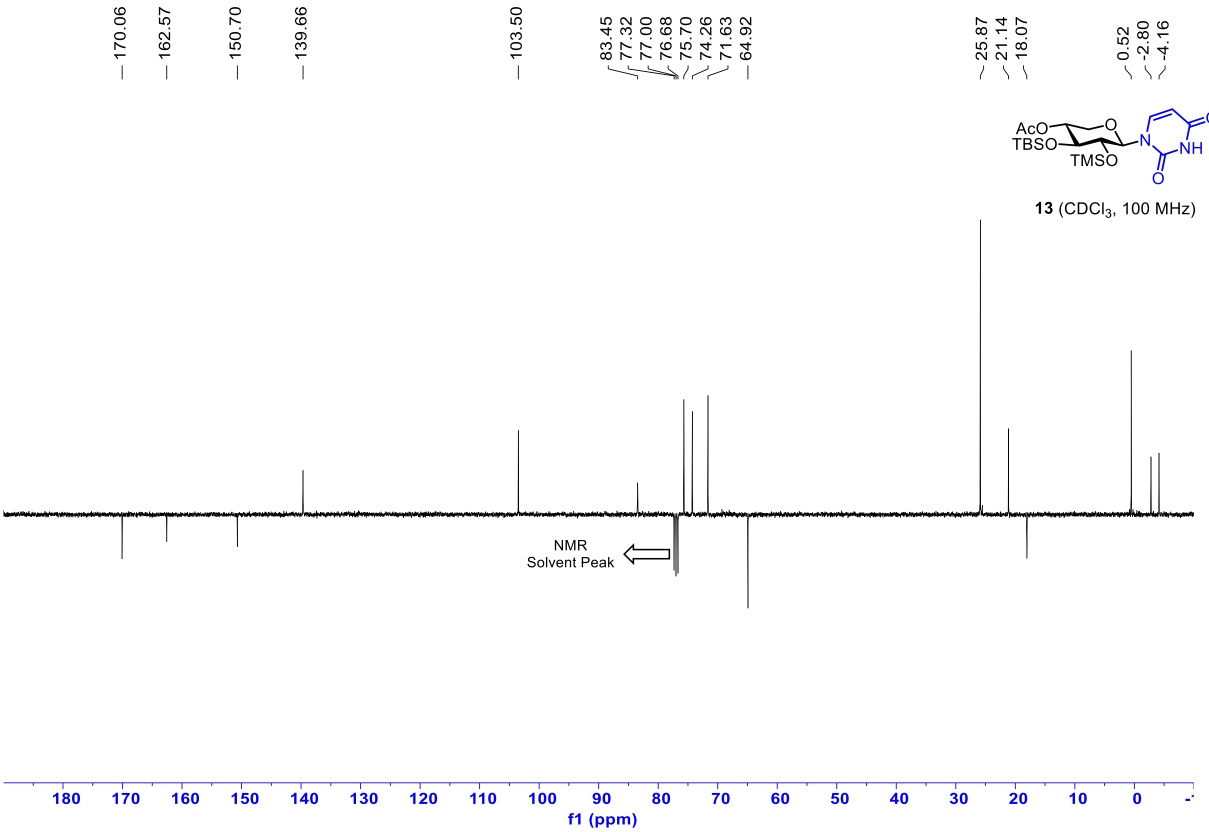
170.06
162.57
150.70
139.66
103.50
83.45
77.32
77.00
76.68
75.70
74.26
71.63
64.92
25.87
21.14
18.07
0.52
-2.80
-4.16
AcO
TBSO
TMSO
O
N
NH
O
O
13 (CDCl3, 100 MHz)
NMR
Solvent Peak
f1 (ppm)

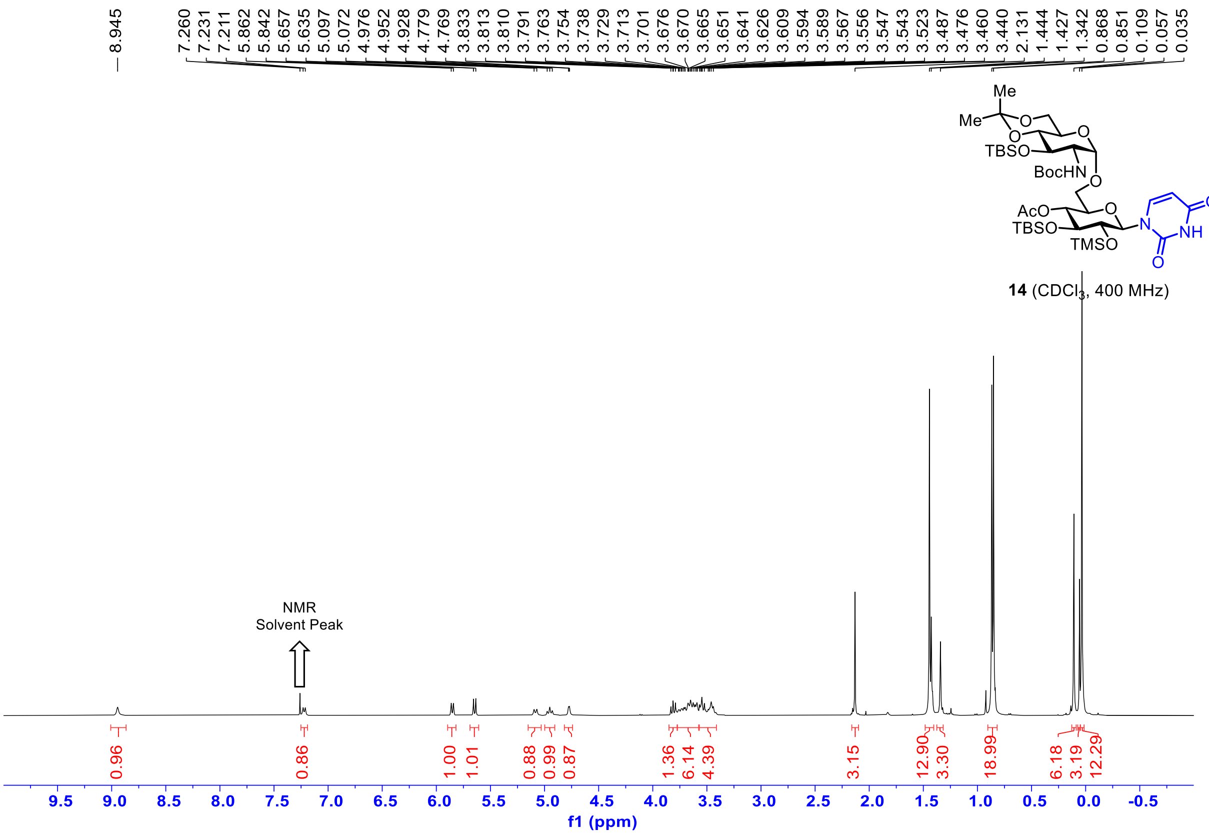
14 (CDCl3, 400 MHz)
NMR Solvent Peak
f1 (ppm)

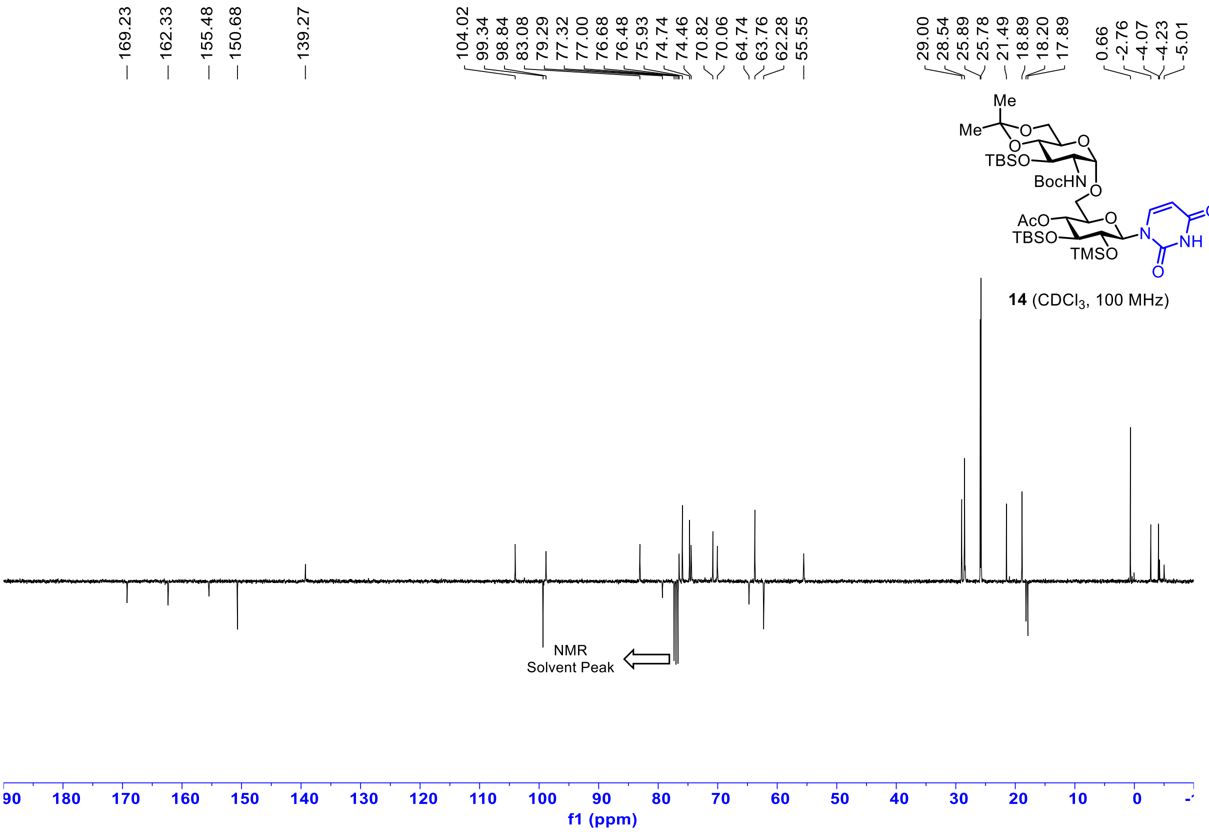
14 (CDCl3, 100 MHz)
NMR Solvent Peak
f1 (ppm)

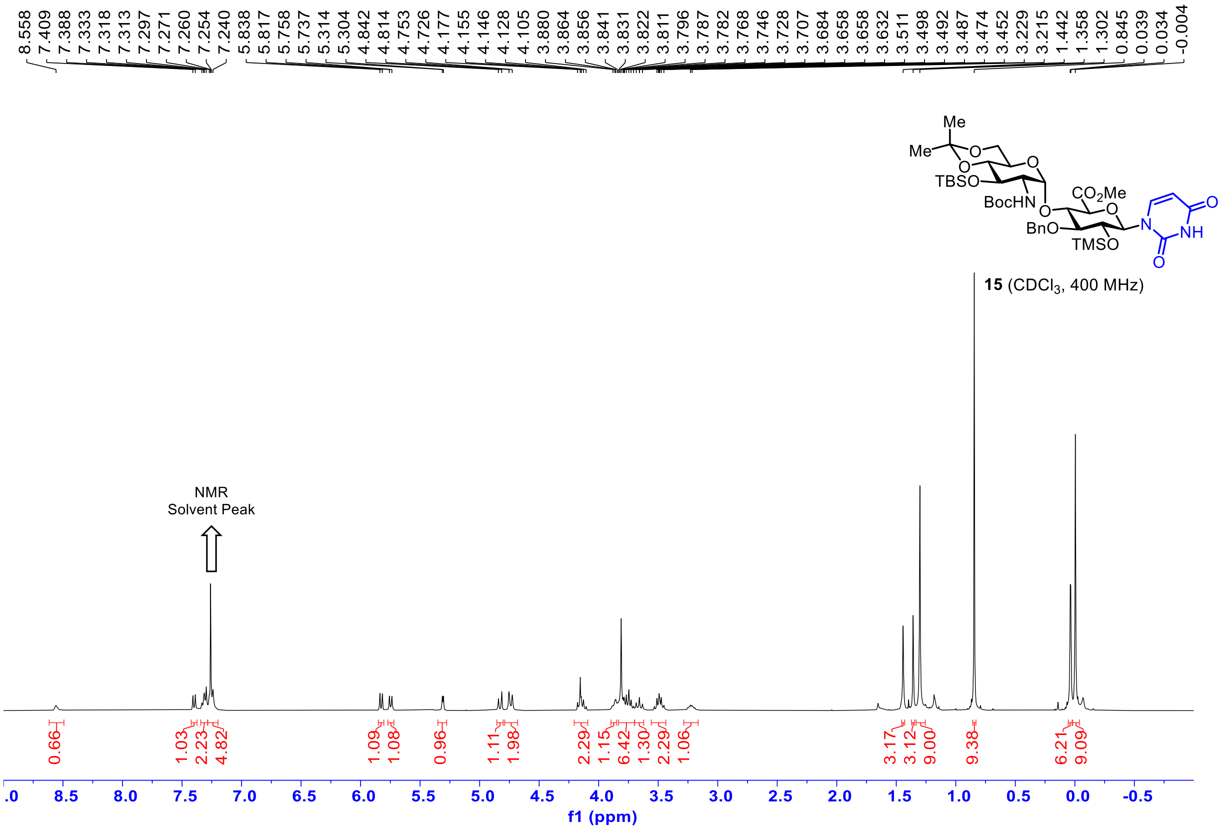
8.558 7.409 7.388 7.333 7.318 7.313 7.297 7.271 7.260 7.254 7.240 5.838 5.817 5.758 5.737 5.304 4.842 4.814 4.753 4.726 4.177 4.155 4.146 4.128 4.105 3.880 3.864 3.856 3.841 3.831 3.822 3.811 3.796 3.787 3.782 3.768 3.746 3.728 3.707 3.684 3.658 3.658 3.632 3.511 3.498 3.492 3.487 3.474 3.452 3.229 3.215 1.442 1.358 1.302 0.845 0.039 0.034 -0.004
Me
Me
O
O
O
TBSO
BocHN
O
CO2Me
O
BnO
TMSO
N
O
NH
O
15 (CDCl3, 400 MHz)
NMR
Solvent Peak
0.66 1.03 2.23 4.82 1.09 1.08 0.96 1.11 1.98 2.29 1.15 6.42 1.30 2.29 1.06 3.17 3.12 9.00 9.38 6.21 9.09
f1 (ppm)

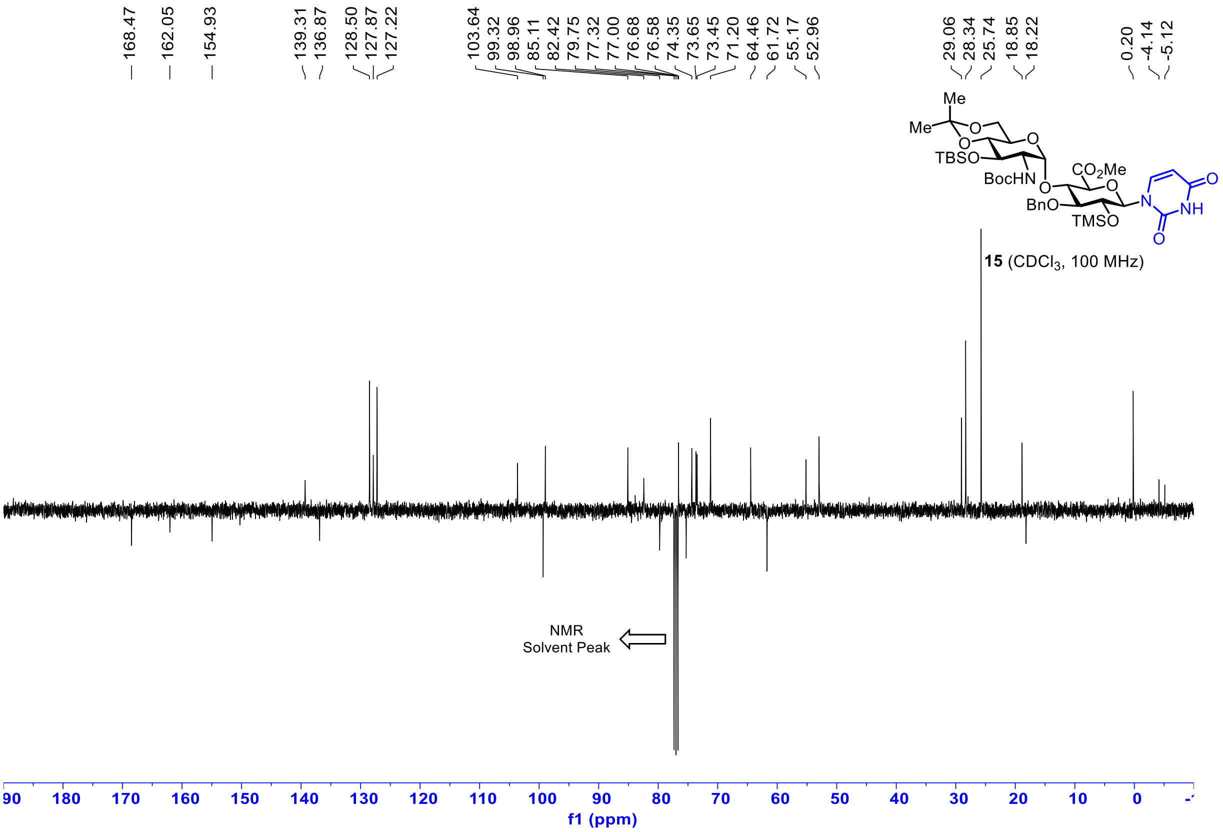
168.47 162.05 154.93 139.31 136.87 128.50 127.87 127.22 103.64 99.32 98.96 85.11 82.42 79.75 77.32 77.00 76.68 76.58 74.35 73.65 73.45 71.20 64.46 61.72 55.17 52.96 29.06 28.34 25.74 18.85 18.22 0.20 -4.14 -5.12
Me
Me
O
O
O
TBSO
BocHN
O
CO2Me
O
BnO
TMSO
N
O
NH
O
15 (CDCl3, 100 MHz)
NMR
Solvent Peak
f1 (ppm)

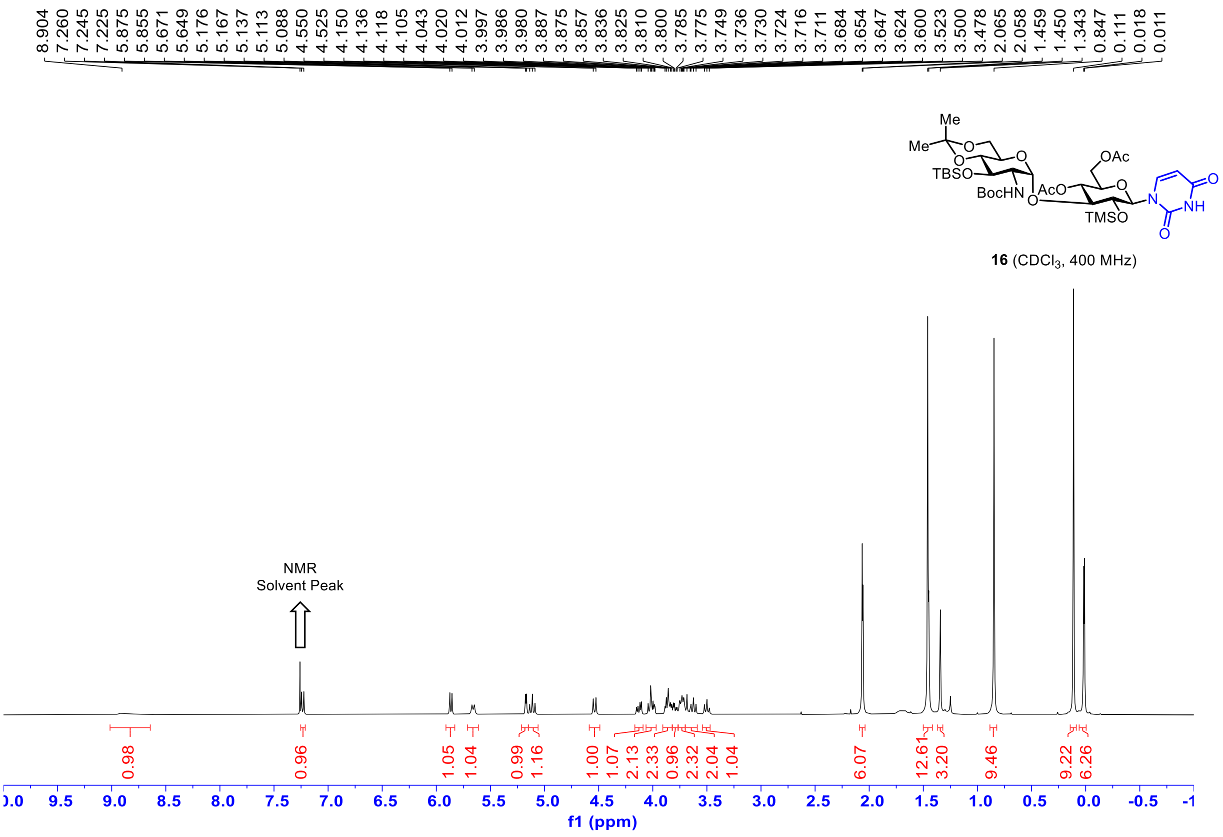

16 (CDCl3, 400 MHz)
NMR
Solvent Peak
f1 (ppm)

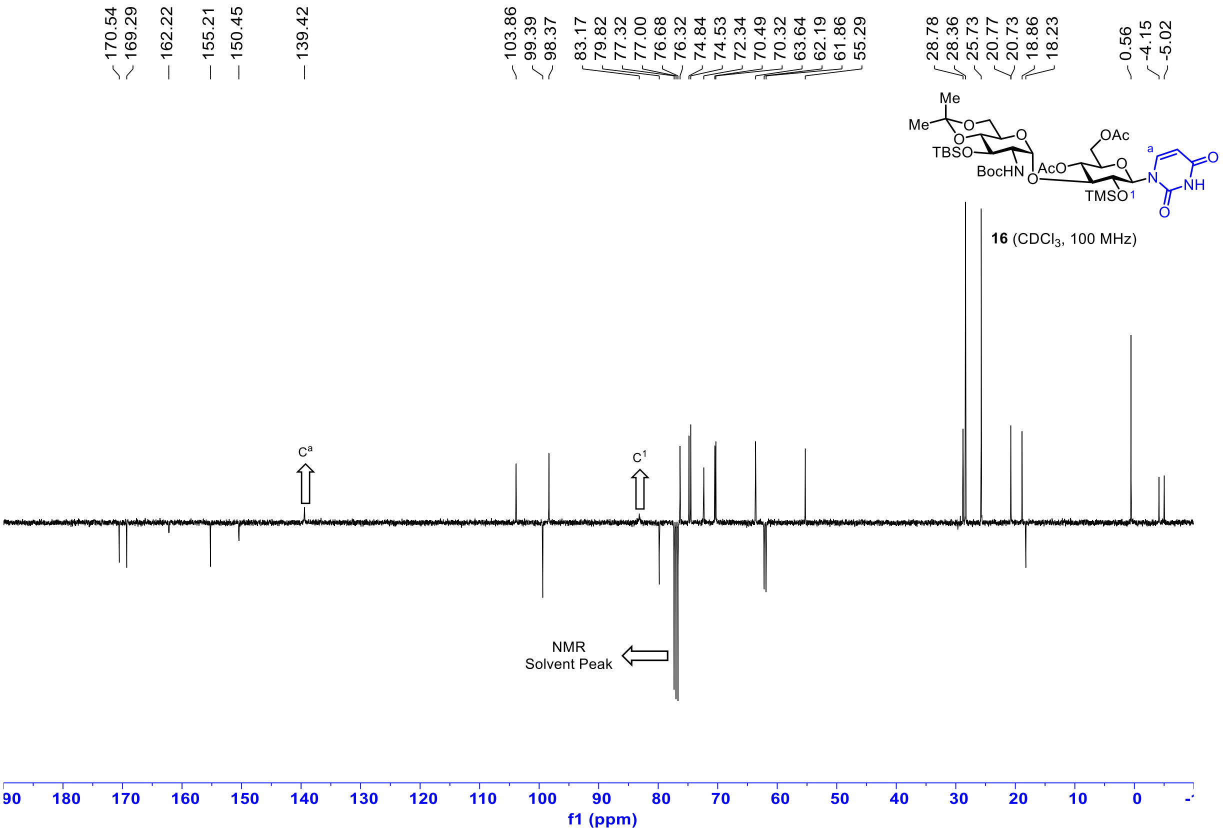

16 (CDCl3, 100 MHz)
NMR
Solvent Peak
f1 (ppm)

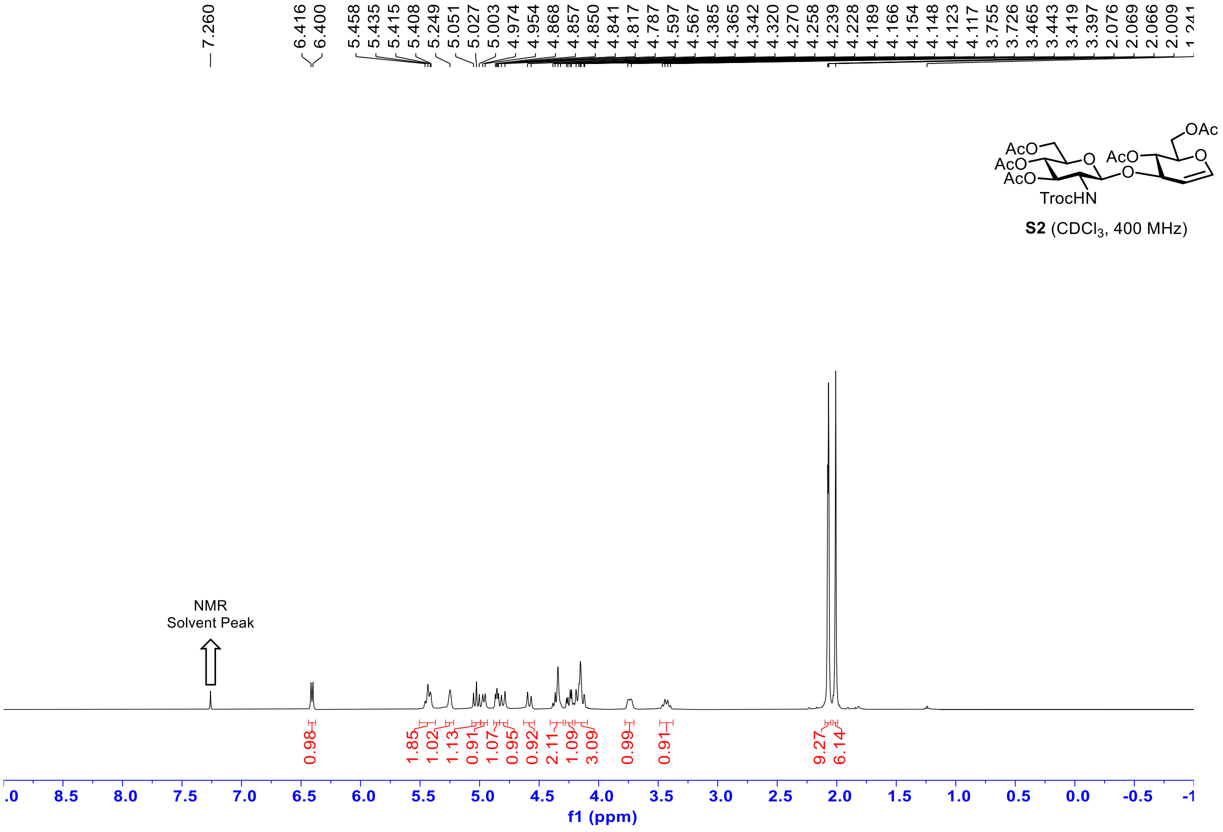
7.260
6.416
6.400
5.458
5.435
5.415
5.408
5.249
5.051
5.027
5.003
4.974
4.954
4.868
4.857
4.850
4.841
4.817
4.787
4.597
4.567
4.385
4.365
4.342
4.320
4.270
4.258
4.239
4.228
4.189
4.166
4.154
4.148
4.123
4.117
3.755
3.726
3.465
3.443
3.419
3.397
2.076
2.069
2.066
2.009
AcO
AcO
AcO
OAc
AcO
O
O
O
TrocHN
S2 (CDCl3, 400 MHz)
NMR
Solvent Peak
0.98
1.85
1.02
1.13
0.91
1.07
0.95
0.92
2.11
1.09
3.09
0.99
0.91
9.27
6.14
f1 (ppm)

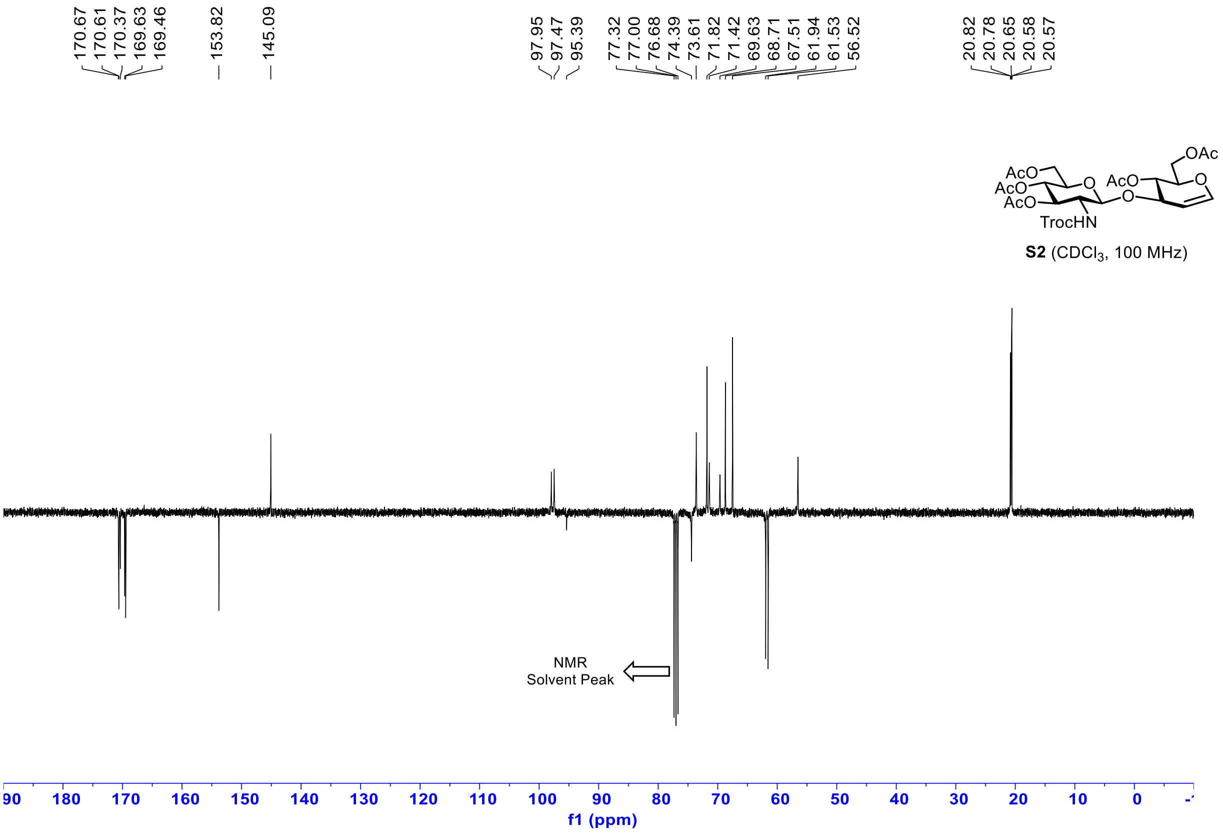
170.67
170.61
170.37
169.63
169.46
153.82
145.09
97.95
97.47
95.39
77.32
77.00
76.68
74.39
73.61
71.82
71.42
69.63
68.71
67.51
61.94
61.53
56.52
20.82
20.78
20.65
20.58
20.57
AcO
AcO
AcO
OAc
AcO
O
O
O
TrocHN
S2 (CDCl3, 100 MHz)
NMR
Solvent Peak
f1 (ppm)

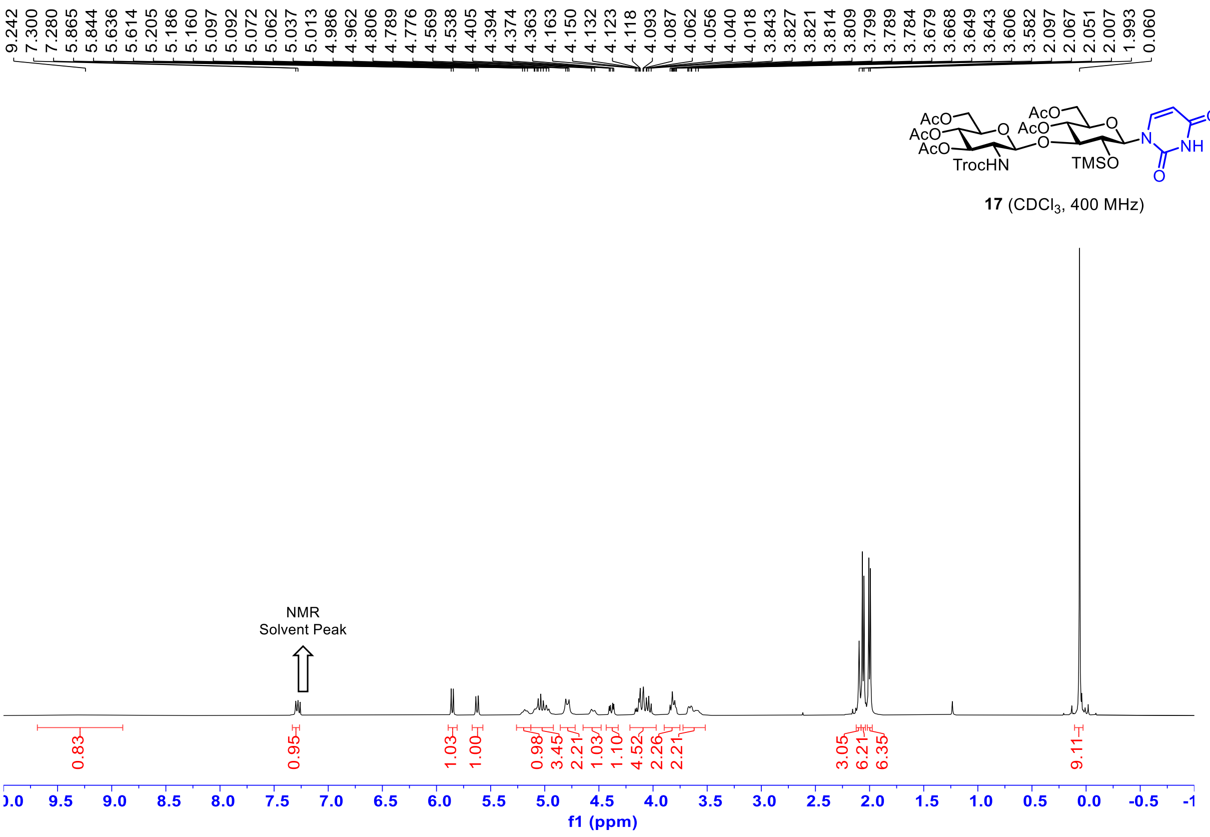
9.242 7.300 7.280 5.865 5.844 5.636 5.614 5.205 5.186 5.160 5.097 5.092 5.072 5.062 5.037 5.013 4.986 4.962 4.806 4.789 4.776 4.569 4.538 4.405 4.394 4.374 4.363 4.163 4.150 4.132 4.123 4.118 4.093 4.087 4.062 4.056 4.040 4.018 3.843 3.827 3.821 3.814 3.809 3.799 3.789 3.784 3.679 3.668 3.649 3.643 3.606 3.582 2.097 2.067 2.051 2.007 1.993 0.060
AcO
AcO
AcO
O
TrocHN
AcO
AcO
O
O
TMSO
N
NH
O
O
17 (CDCl3, 400 MHz)
NMR
Solvent Peak
0.83
0.95
1.03
1.00
0.98
3.45
2.21
1.03
1.10
4.52
2.26
2.21
3.05
6.21
6.35
9.11
f1 (ppm)

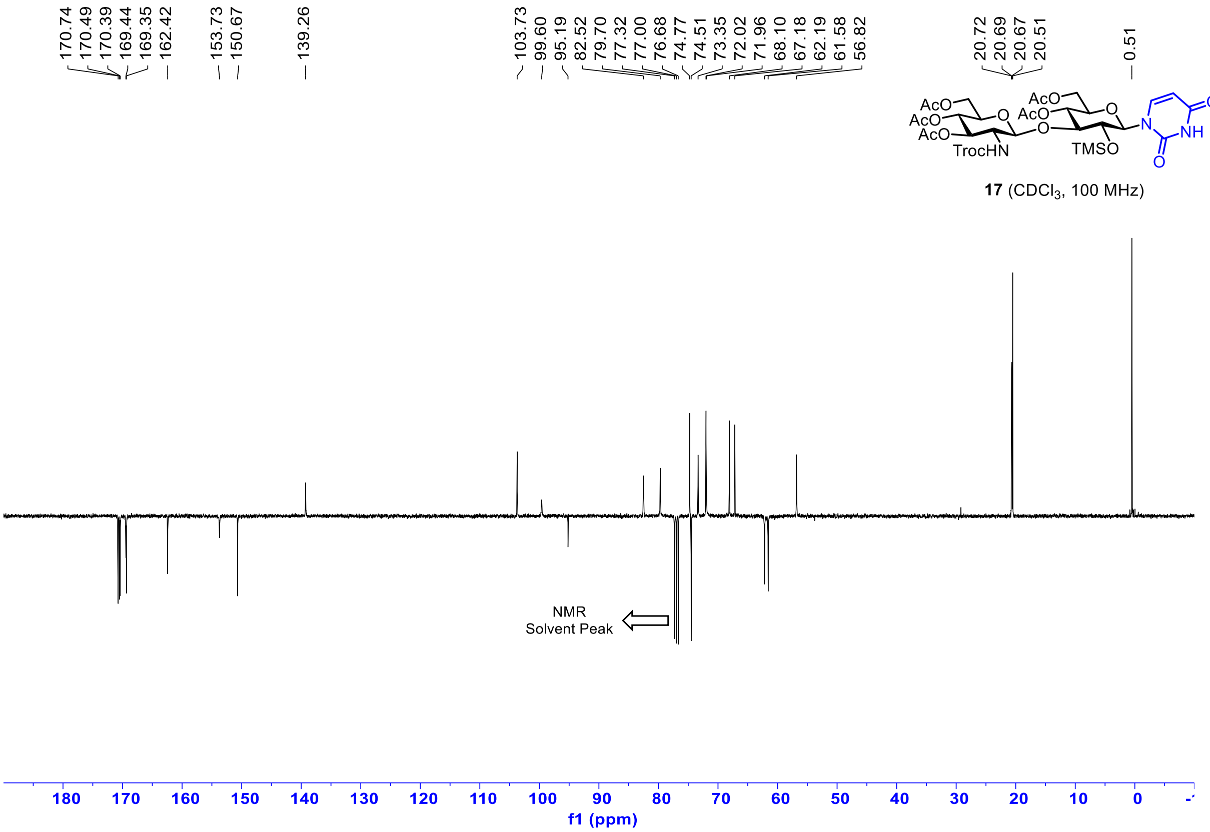
170.74 170.49 170.39 169.44 169.35 162.42 153.73 150.67 139.26 103.73 99.60 95.19 82.52 79.70 77.32 77.00 76.68 74.77 74.51 73.35 72.02 71.96 68.10 67.18 62.19 61.58 56.82 20.72 20.69 20.67 20.51 0.51
AcO
AcO
AcO
O
TrocHN
AcO
AcO
O
O
TMSO
N
NH
O
O
17 (CDCl3, 100 MHz)
NMR
Solvent Peak
f1 (ppm)

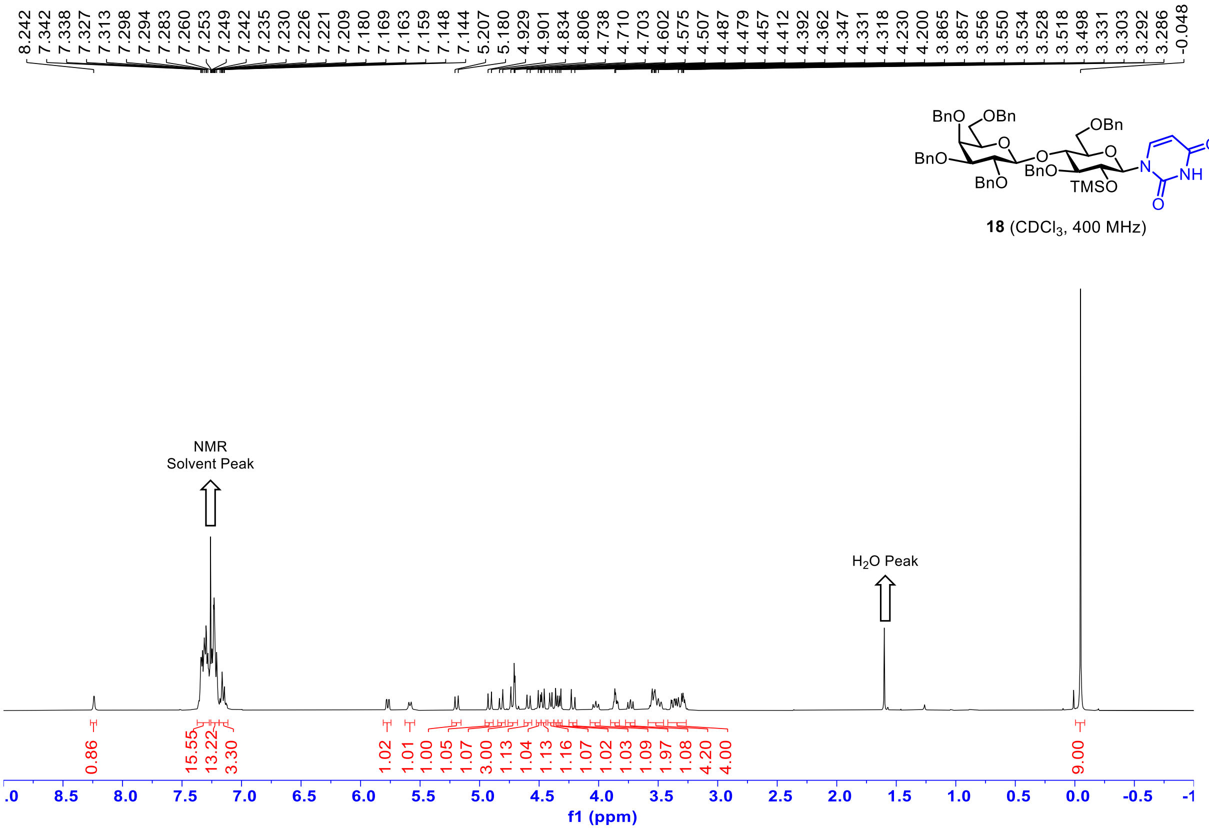
8.242
7.342
7.338
7.327
7.313
7.298
7.294
7.283
7.260
7.253
7.249
7.242
7.235
7.230
7.226
7.221
7.209
7.180
7.169
7.163
7.159
7.148
7.144
5.207
5.180
4.929
4.901
4.834
4.806
4.738
4.710
4.703
4.602
4.575
4.507
4.487
4.479
4.457
4.412
4.392
4.362
4.347
4.331
4.318
4.230
4.200
3.865
3.857
3.556
3.550
3.534
3.528
3.518
3.498
3.331
3.303
3.292
3.286
-0.048
18 (CDCl3, 400 MHz)
NMR Solvent Peak
H2O Peak
0.86
15.55
13.22
3.30
1.02
1.01
1.00
1.05
1.07
3.00
1.13
1.04
1.13
1.16
1.07
1.02
1.03
1.09
1.97
1.08
4.20
4.00
9.00
f1 (ppm)

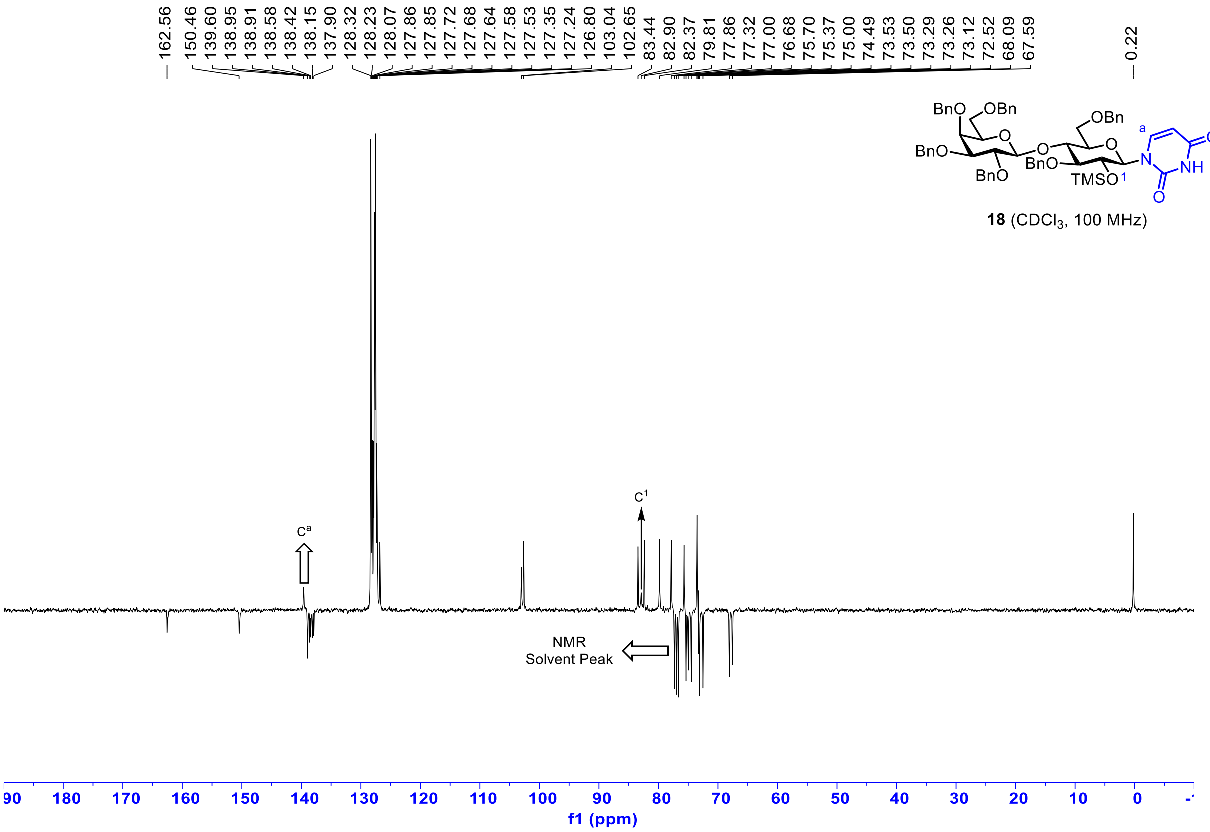
162.56
150.46
139.60
138.95
138.91
138.58
138.42
138.15
137.90
128.32
128.23
128.07
127.86
127.85
127.72
127.68
127.64
127.58
127.53
127.35
127.24
126.80
103.04
102.65
83.44
82.90
82.37
79.81
77.86
77.32
77.00
76.68
75.70
75.37
75.00
74.49
73.53
73.50
73.29
73.26
73.12
72.52
68.09
67.59
0.22
18 (CDCl3, 100 MHz)
C^a
C^1
NMR Solvent Peak
f1 (ppm)

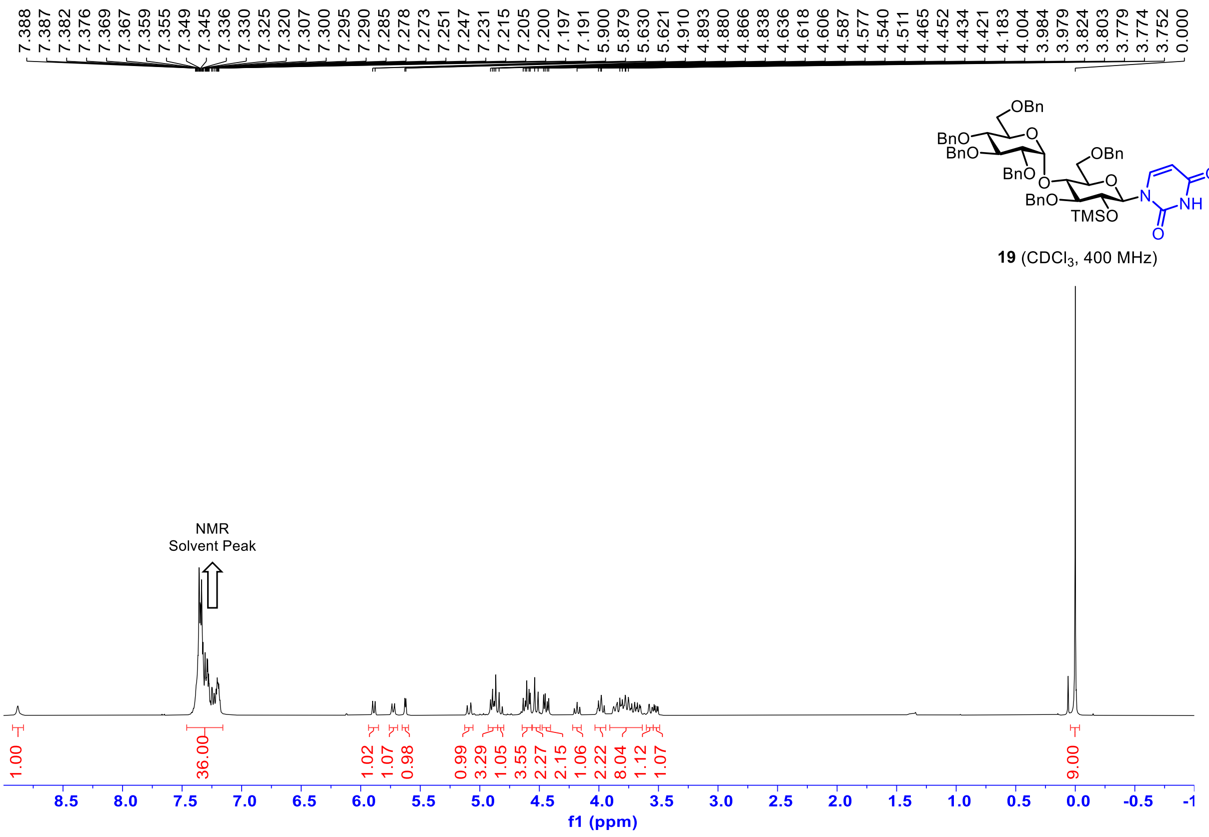
7.388 7.387 7.382 7.376 7.369 7.367 7.359 7.355 7.349 7.345 7.336 7.330 7.325 7.320 7.307 7.300 7.295 7.290 7.285 7.278 7.273 7.251 7.247 7.231 7.215 7.205 7.200 7.197 7.191 5.900 5.879 5.630 5.621 4.910 4.893 4.880 4.866 4.838 4.636 4.618 4.606 4.587 4.577 4.540 4.511 4.465 4.452 4.434 4.421 4.183 4.004 3.984 3.979 3.824 3.803 3.779 3.774 3.752 0.000
19 (CDCl3, 400 MHz)
NMR
Solvent Peak
1.00 36.00 1.02 1.07 0.98 0.99 3.29 1.05 3.55 2.27 2.15 1.06 2.22 8.04 1.12 1.07 9.00
f1 (ppm)

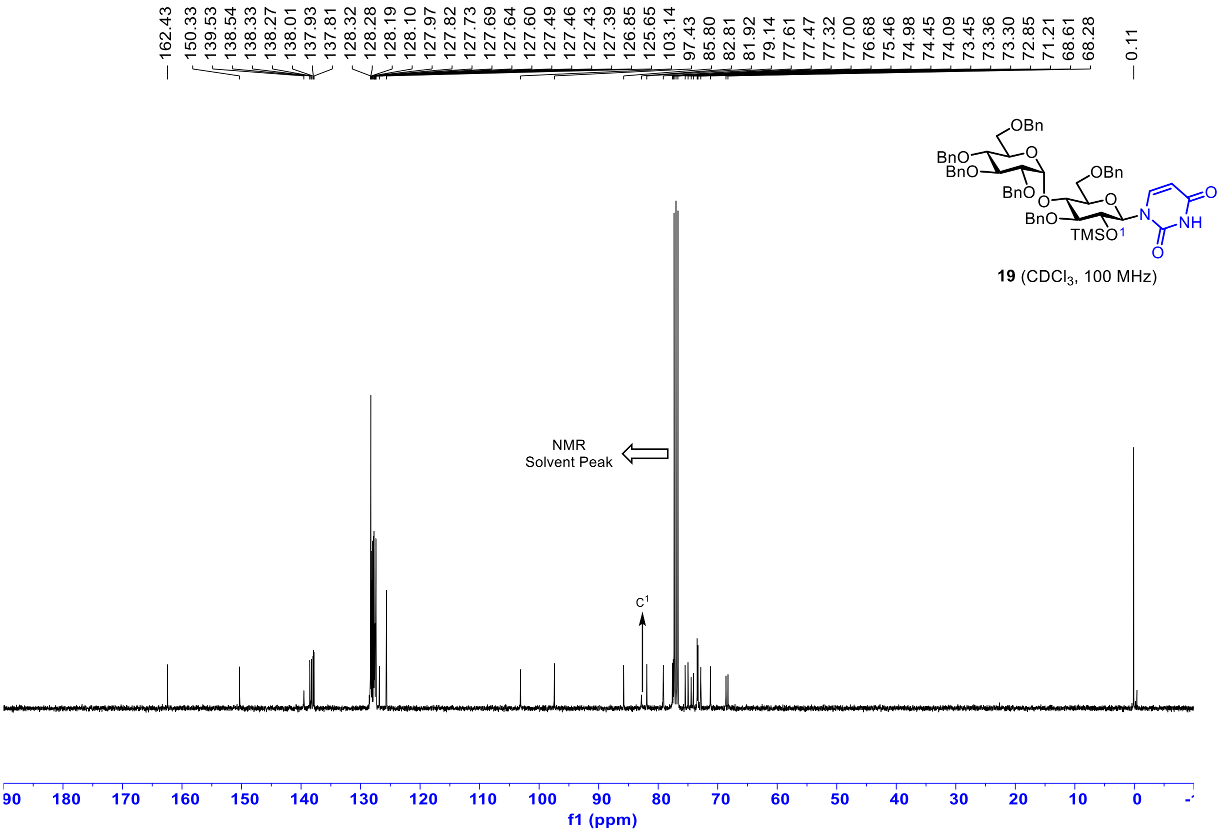
162.43 150.33 139.53 138.54 138.33 138.27 138.01 137.93 137.81 128.32 128.28 128.19 128.10 127.97 127.82 127.73 127.69 127.64 127.60 127.49 127.46 127.43 127.39 126.85 125.65 103.14 97.43 85.80 82.81 81.92 79.14 77.61 77.47 77.32 77.00 76.68 75.46 74.98 74.45 74.09 73.45 73.36 73.30 72.85 71.21 68.61 68.28 0.11
19 (CDCl3, 100 MHz)
NMR
Solvent Peak
C1
f1 (ppm)

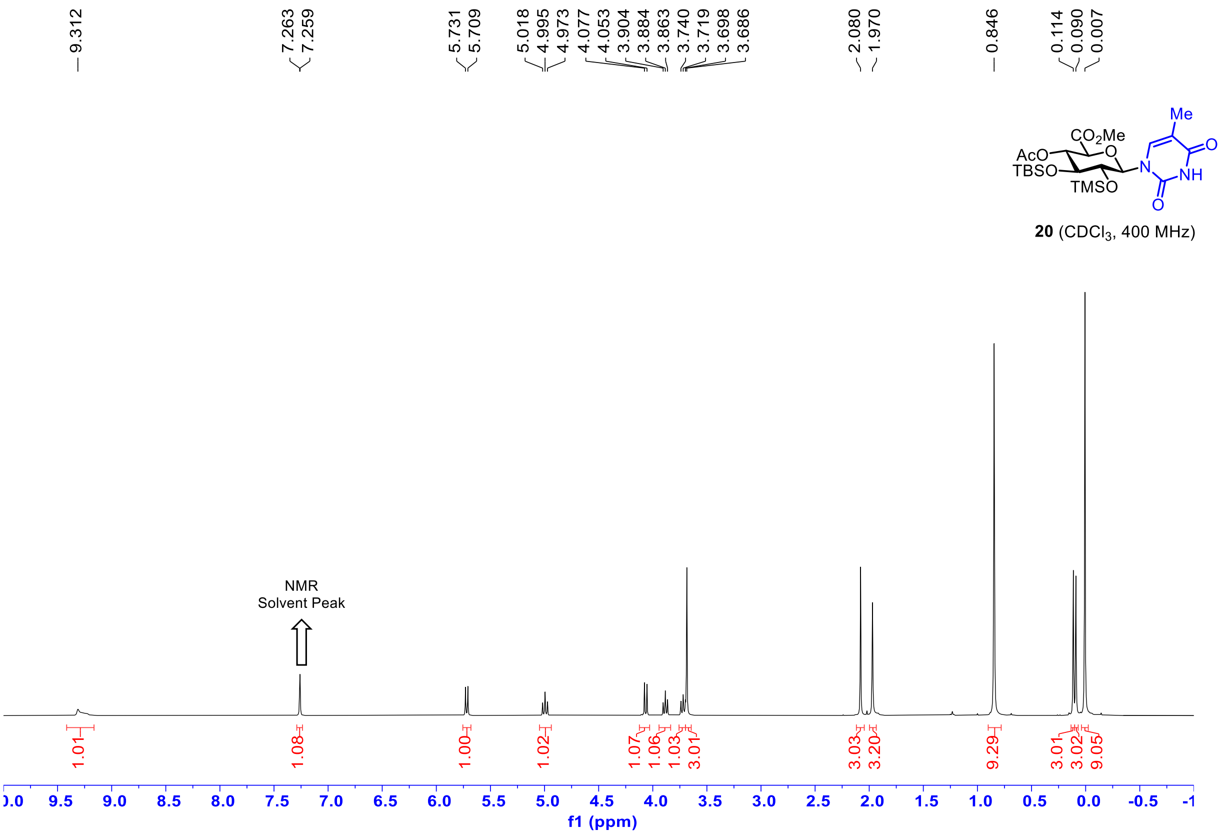
9.312
7.263
7.259
5.731
5.709
5.018
4.995
4.973
4.077
4.053
3.904
3.884
3.863
3.740
3.719
3.698
3.686
2.080
1.970
0.846
0.114
0.090
0.007
Me
CO2Me
AcO
TBSO
TMSO
O
N
NH
O
O
20 (CDCl3, 400 MHz)
NMR
Solvent Peak
1.01
1.08
1.00
1.02
1.07
1.06
1.03
3.01
3.03
3.20
9.29
3.01
3.02
9.05
f1 (ppm)

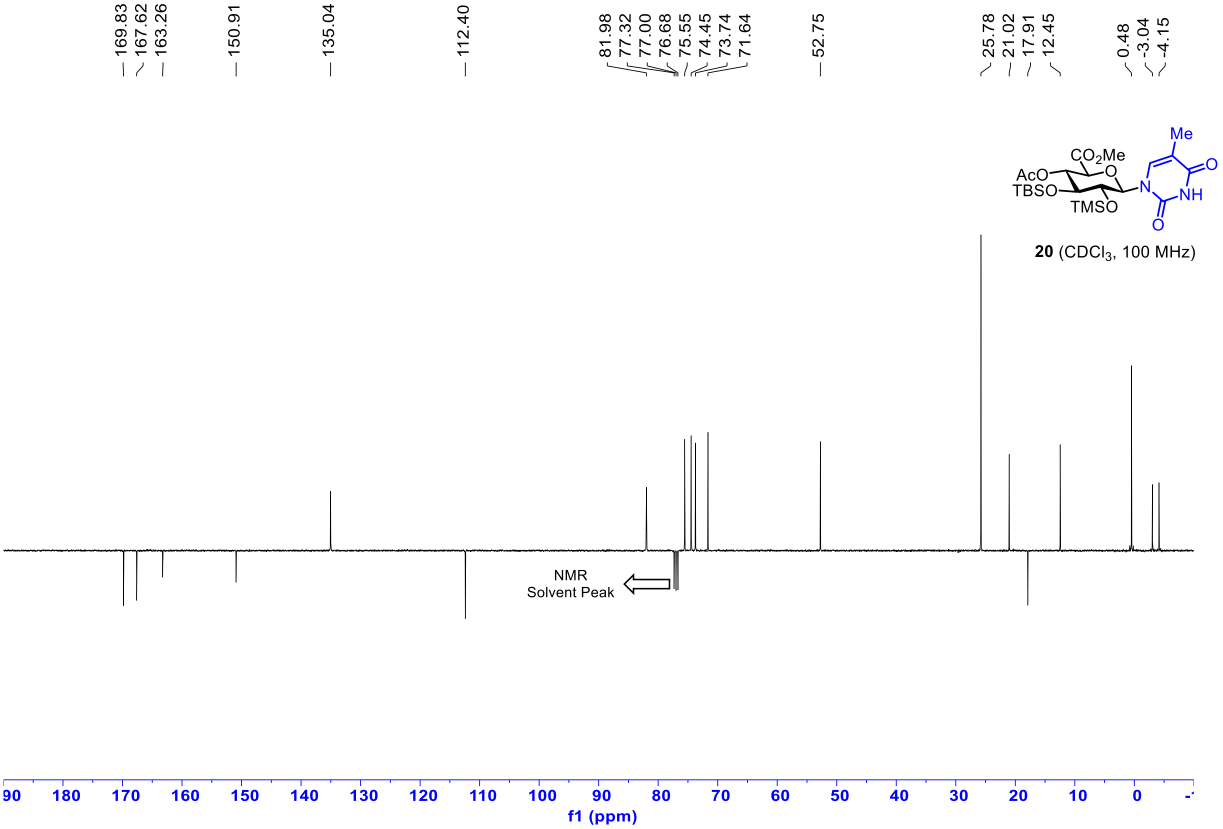
169.83
167.62
163.26
150.91
135.04
112.40
81.98
77.32
77.00
76.68
75.55
74.45
73.74
71.64
52.75
25.78
21.02
17.91
12.45
0.48
-3.04
-4.15
Me
CO2Me
AcO
TBSO
TMSO
O
N
NH
O
O
20 (CDCl3, 100 MHz)
NMR
Solvent Peak
f1 (ppm)

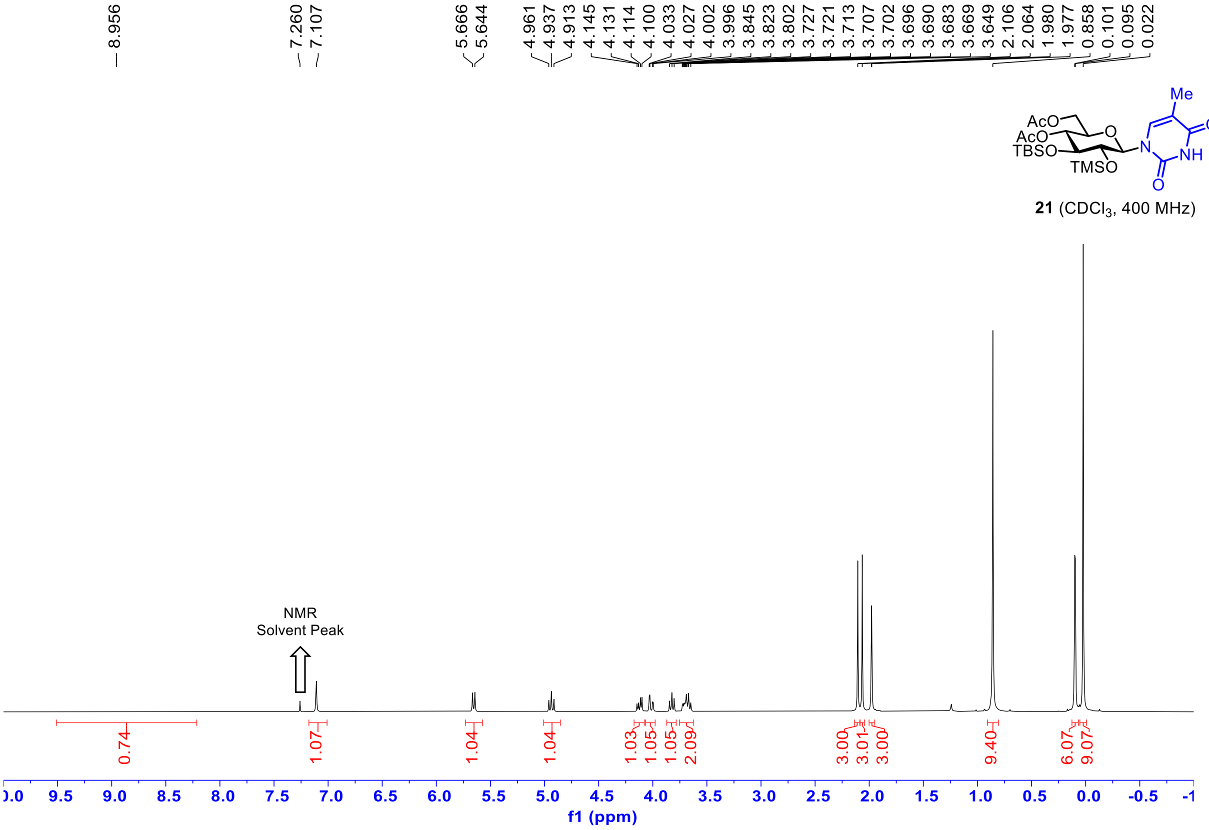
8.956
7.260
7.107
5.666
5.644
4.961
4.937
4.913
4.145
4.131
4.114
4.100
4.033
4.027
4.002
3.996
3.845
3.823
3.802
3.727
3.721
3.713
3.707
3.702
3.696
3.690
3.683
3.669
3.649
2.106
2.064
1.980
1.977
0.858
0.101
0.095
0.022
Me
AcO
AcO
TBSO
TMSO
O
N
O
NH
O
21 (CDCl3, 400 MHz)
NMR Solvent Peak
0.74
1.07
1.04
1.04
1.03
1.05
1.05
2.09
3.00
3.01
3.00
9.40
6.07
9.07
f1 (ppm)

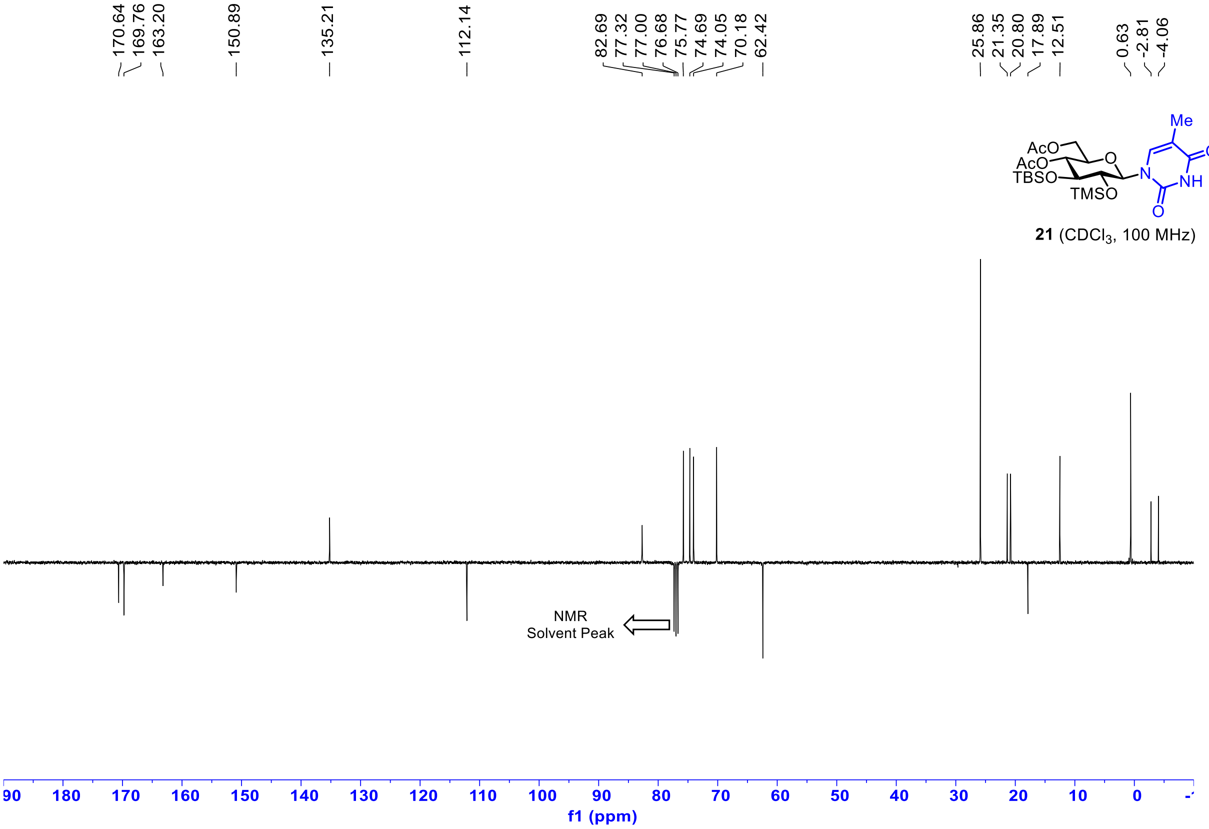
170.64
169.76
163.20
150.89
135.21
112.14
82.69
77.32
77.00
76.68
75.77
74.69
74.05
70.18
62.42
25.86
21.35
20.80
17.89
12.51
0.63
-2.81
-4.06
Me
AcO
AcO
TBSO
TMSO
O
N
O
NH
O
21 (CDCl3, 100 MHz)
NMR Solvent Peak
f1 (ppm)

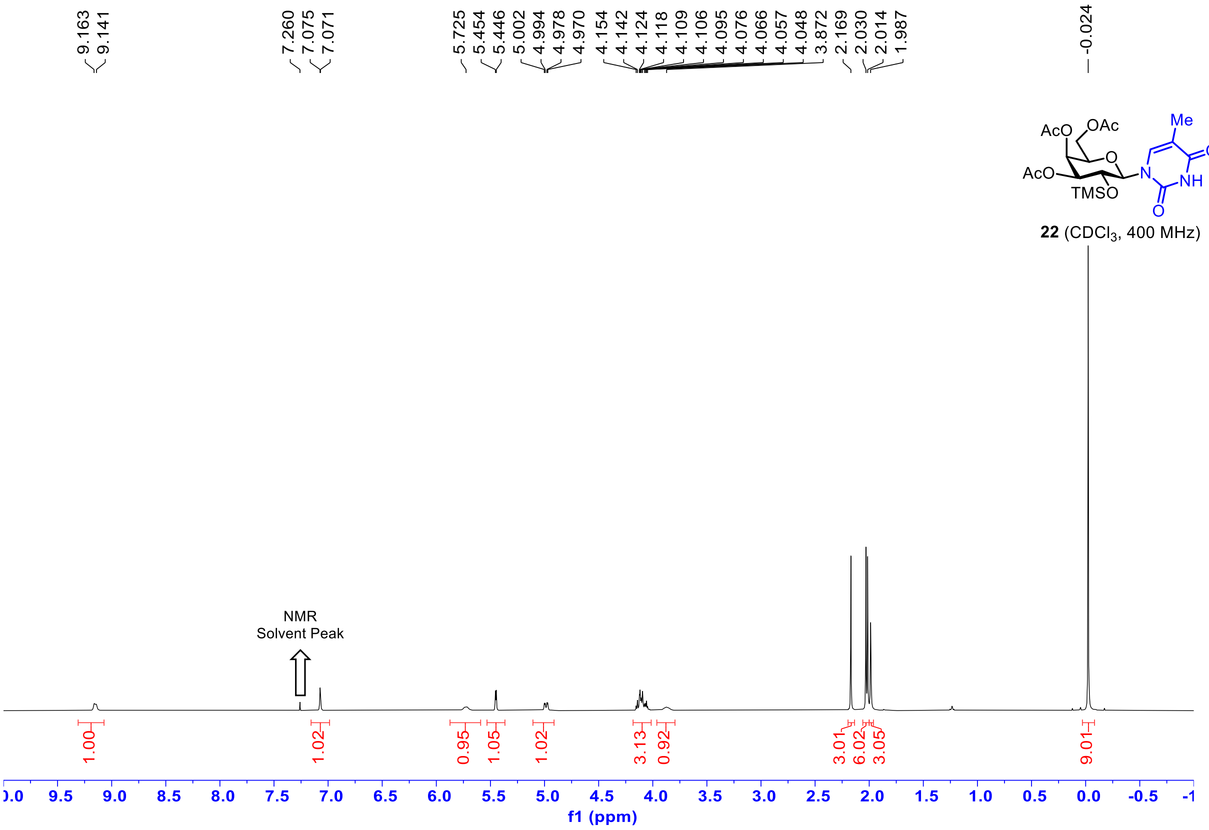
9.163
9.141
7.260
7.075
7.071
5.725
5.454
5.446
5.002
4.994
4.978
4.970
4.154
4.142
4.124
4.118
4.109
4.106
4.095
4.076
4.066
4.057
4.048
3.872
2.169
2.030
2.014
1.987
-0.024
AcO
OAc
Me
O
N
O
AcO
NH
TMSO
O
22 (CDCl3, 400 MHz)
NMR
Solvent Peak
1.00
1.02
0.95
1.05
1.02
3.13
0.92
3.01
6.02
3.05
9.01
f1 (ppm)

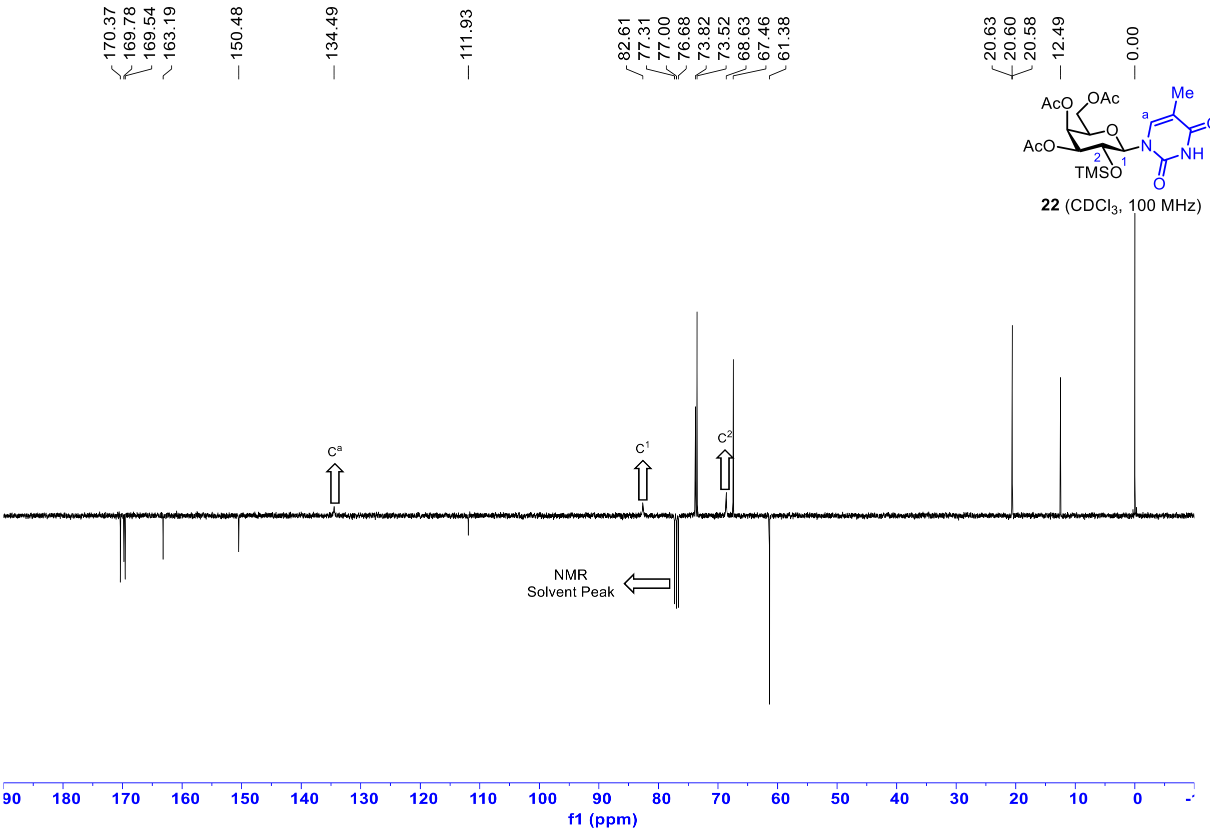
170.37
169.78
169.54
163.19
150.48
134.49
111.93
82.61
77.31
77.00
76.68
73.82
73.52
68.63
67.46
61.38
20.63
20.60
20.58
12.49
0.00
AcO
OAc
Me
a
O
N
O
AcO
2
1
NH
TMSO
O
22 (CDCl3, 100 MHz)
C^a
C^1
C^2
NMR
Solvent Peak
f1 (ppm)

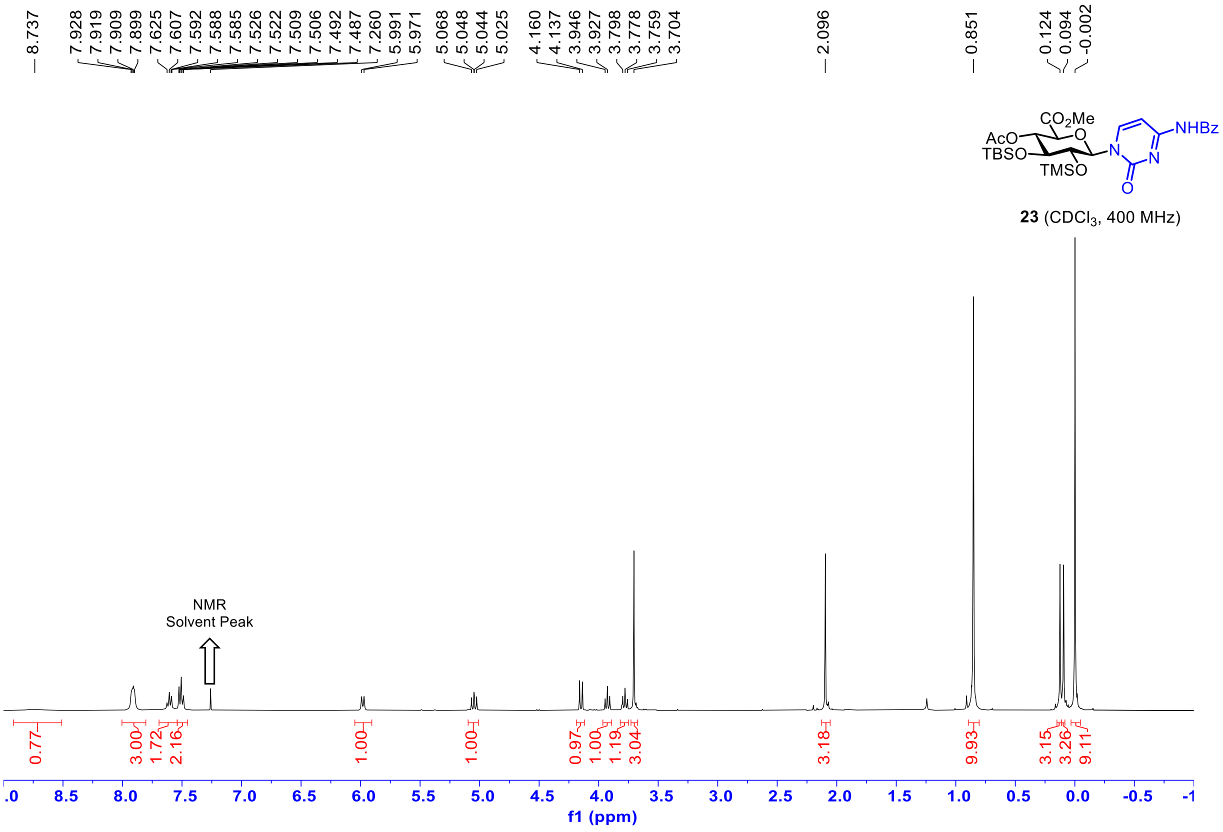

8.737
7.928
7.919
7.909
7.899
7.625
7.607
7.592
7.588
7.585
7.526
7.522
7.509
7.506
7.492
7.487
7.260
5.991
5.971
5.068
5.048
5.044
5.025
4.160
4.137
3.946
3.927
3.798
3.778
3.759
3.704
2.096
0.851
0.124
0.094
-0.002
CO2Me
AcO
TBSO
TMSO
NHBz
N
N
O
23 (CDCl3, 400 MHz)
NMR Solvent Peak
0.77
3.00
1.72
2.16
1.00
1.00
0.97
1.00
1.19
3.04
3.18
9.93
3.15
3.26
9.11
f1 (ppm)

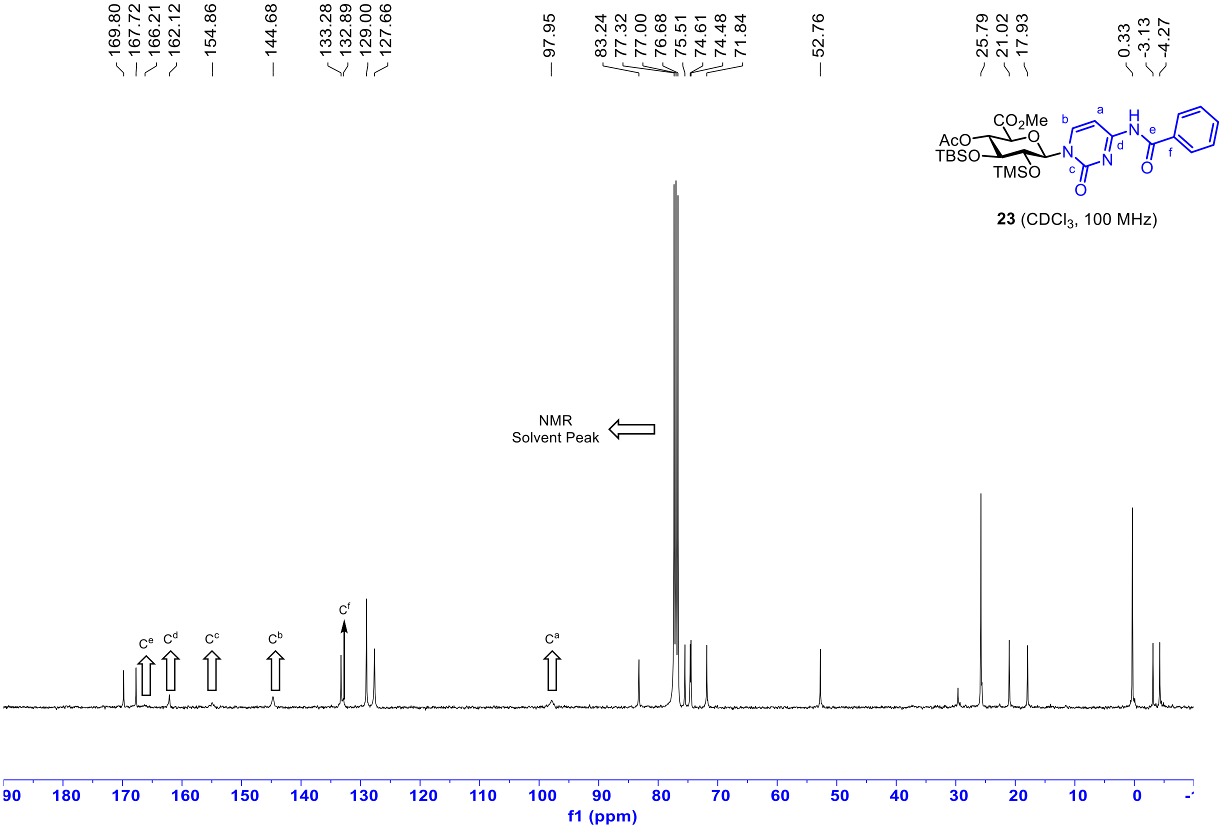

169.80
167.72
166.21
162.12
154.86
144.68
133.28
132.89
129.00
127.66
97.95
83.24
77.32
77.00
76.68
75.51
74.61
74.48
71.84
52.76
25.79
21.02
17.93
0.33
-3.13
-4.27
CO2Me
AcO
TBSO
TMSO
23 (CDCl3, 100 MHz)
NMR Solvent Peak
Ce
Cd
Cc
Cb
Cf
Ca
f1 (ppm)

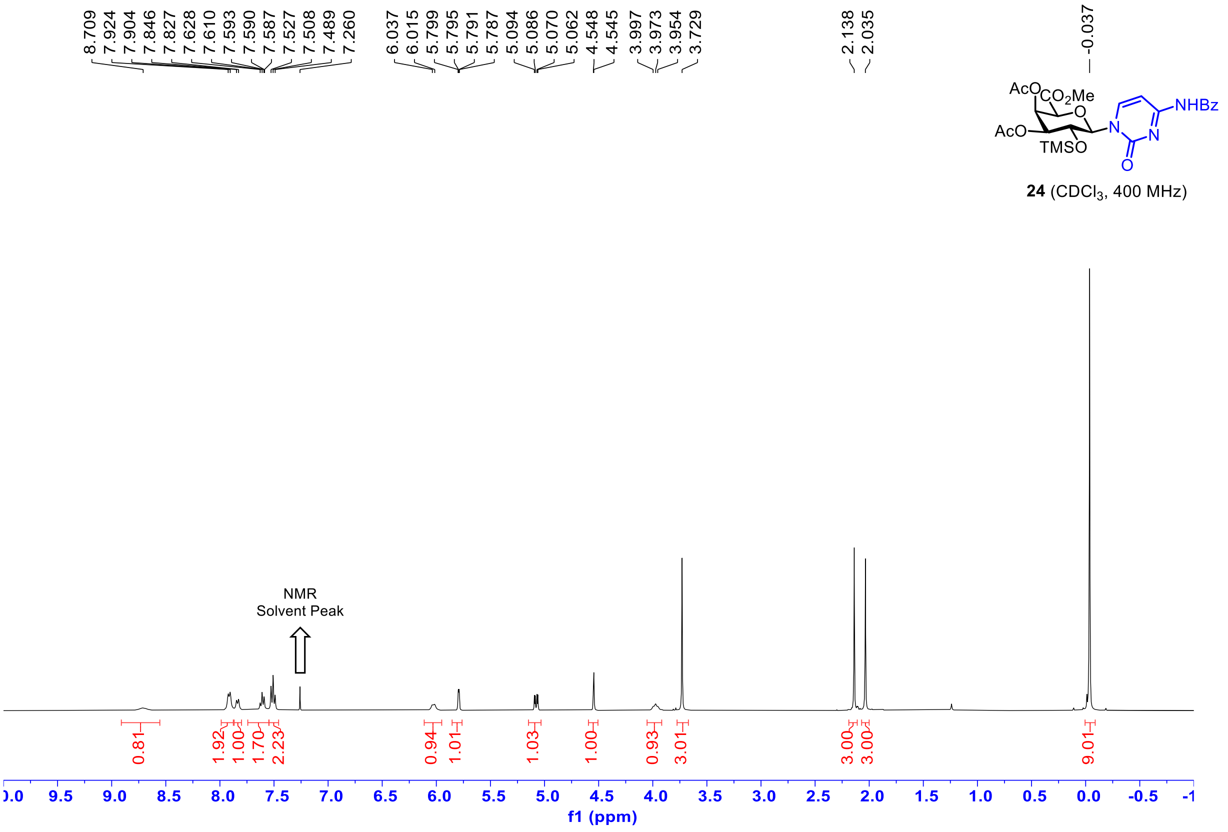
NMR
Solvent Peak
24 (CDCl$_3$, 400 MHz)
f1 (ppm)

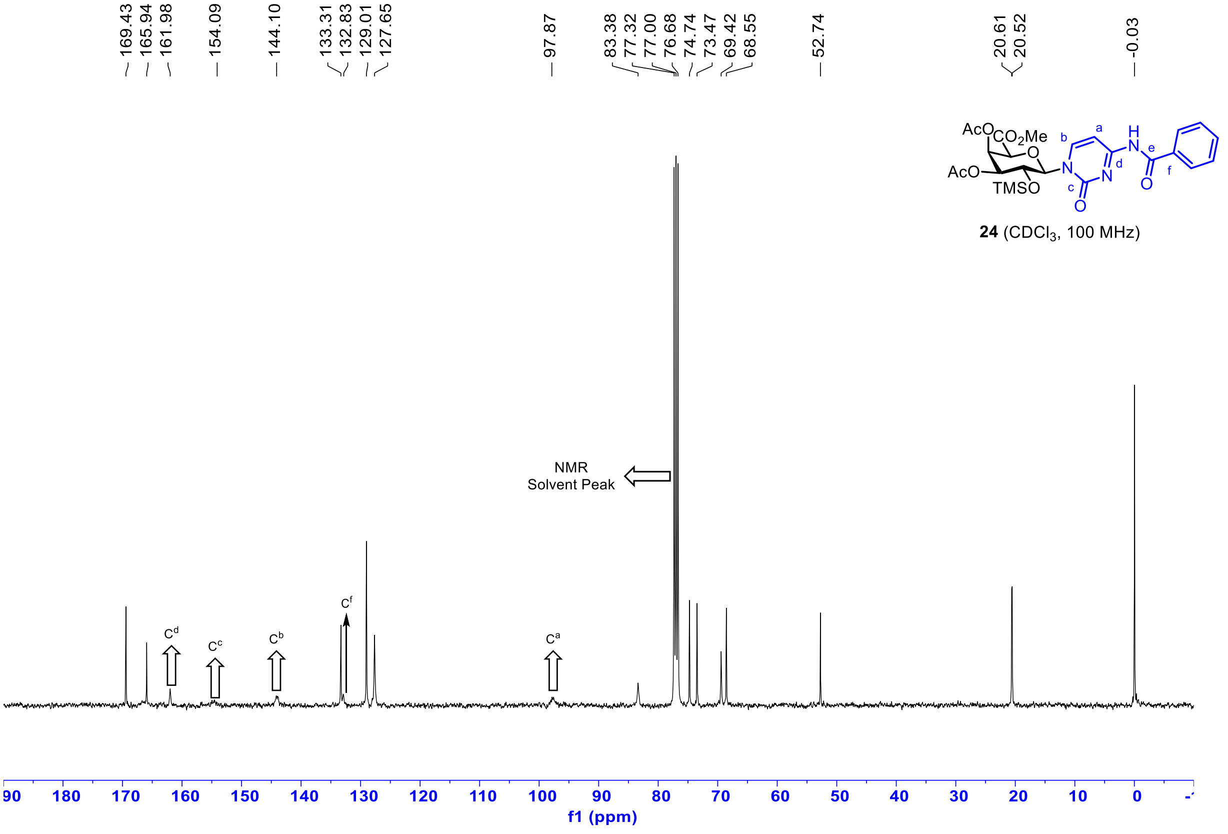
NMR
Solvent Peak
24 (CDCl$_3$, 100 MHz)
f1 (ppm)

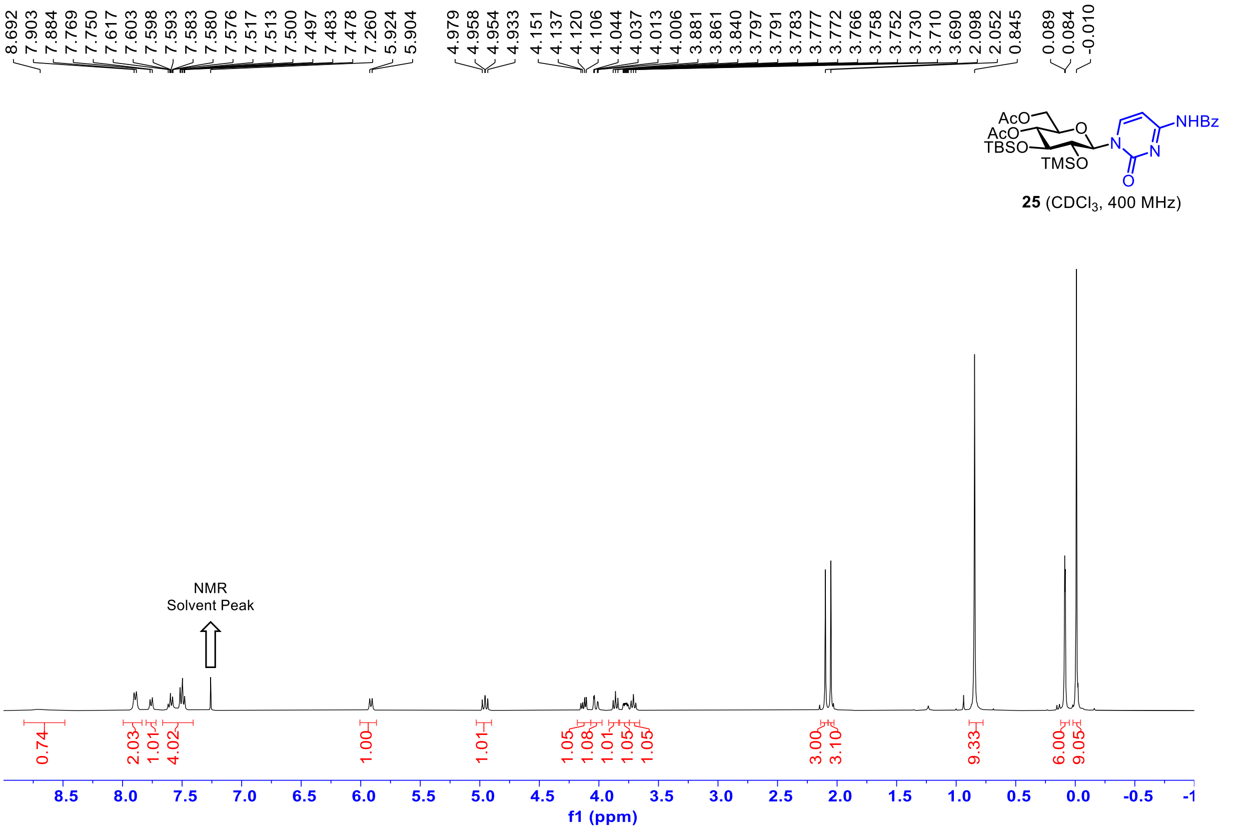
8.692 7.903 7.884 7.769 7.750 7.617 7.603 7.598 7.593 7.583 7.580 7.576 7.517 7.513 7.500 7.497 7.483 7.478 7.260 5.924 5.904 4.979 4.958 4.954 4.933 4.151 4.137 4.120 4.106 4.044 4.037 4.013 4.006 3.881 3.861 3.840 3.797 3.791 3.783 3.777 3.772 3.766 3.758 3.752 3.730 3.710 3.690 2.098 2.052 0.845 0.089 0.084 -0.010
AcO
AcO
TBSO
TMSO
NHBz
25 (CDCl3, 400 MHz)
NMR Solvent Peak
0.74 2.03 1.01 4.02 1.00 1.01 1.05 1.08 1.01 1.05 1.05 3.00 3.10 9.33 6.00 9.05
f1 (ppm)

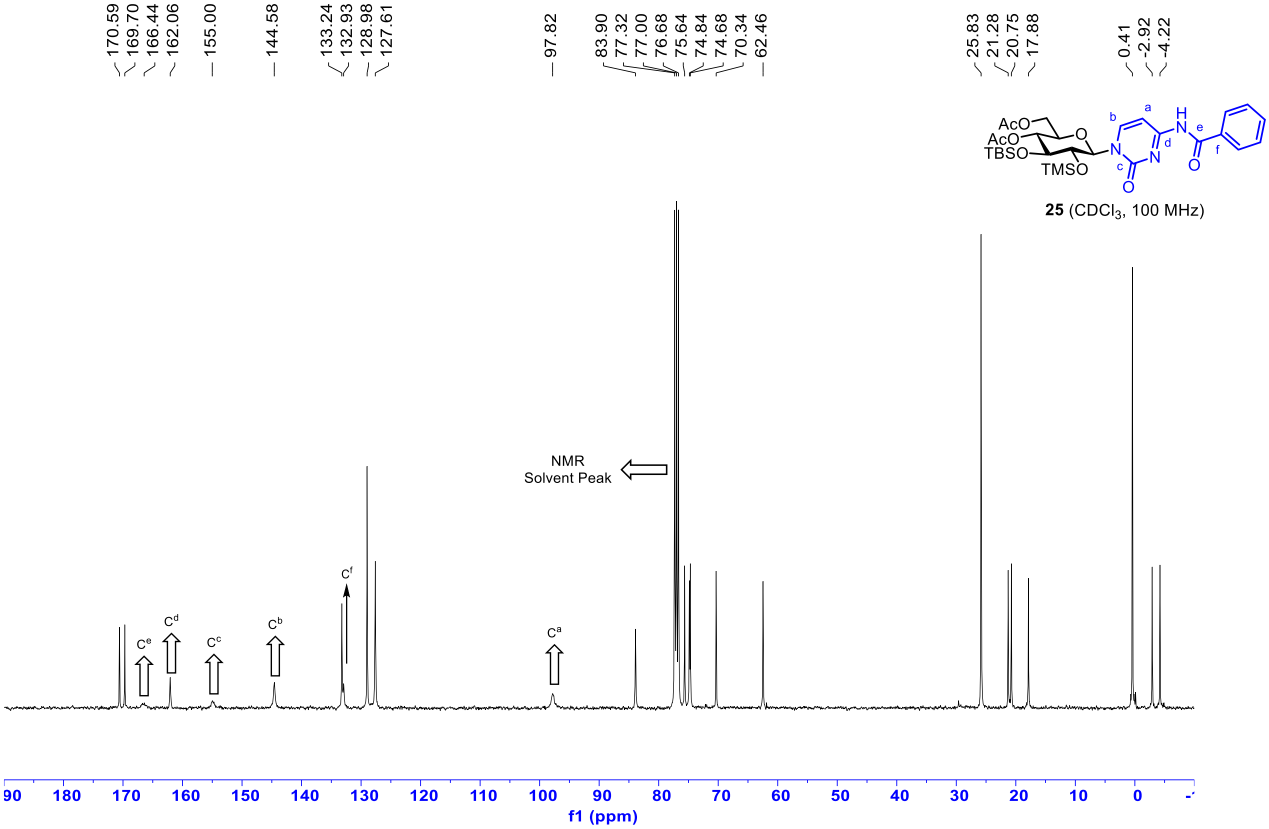
170.59 169.70 166.44 162.06 155.00 144.58 133.24 132.93 128.98 127.61 97.82 83.90 77.32 77.00 76.68 75.64 74.84 74.68 70.34 62.46 25.83 21.28 20.75 17.88 0.41 -2.92 -4.22
AcO
AcO
TBSO
TMSO
25 (CDCl3, 100 MHz)
NMR Solvent Peak
f1 (ppm)

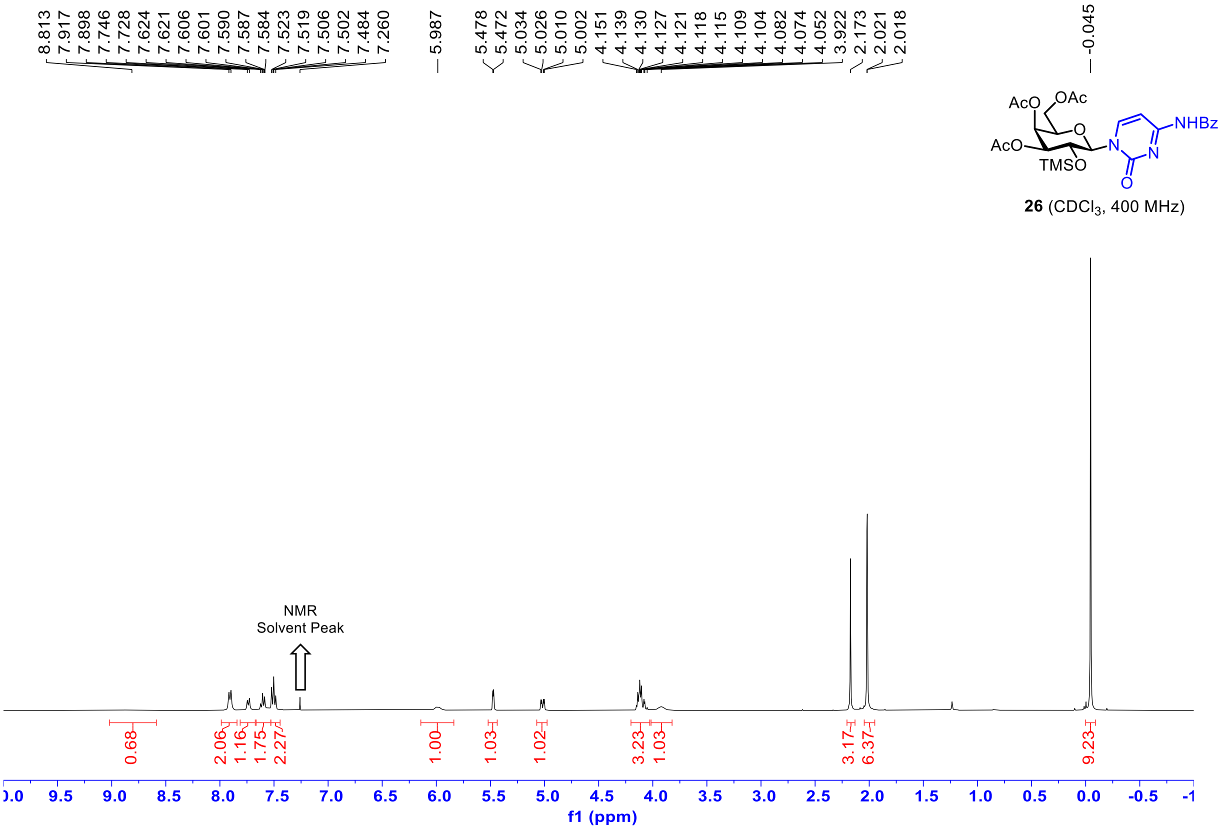
8.813 7.917 7.898 7.746 7.728 7.624 7.621 7.606 7.601 7.590 7.587 7.584 7.523 7.519 7.506 7.502 7.484 7.260
5.987
5.478 5.472 5.034 5.026 5.010 5.002 4.151 4.139 4.130 4.127 4.121 4.118 4.115 4.109 4.104 4.082 4.074 4.052 3.922 2.173 2.021 2.018
-0.045
AcO
OAc
O
N
NHBz
N
AcO
TMSO
O
26 (CDCl3, 400 MHz)
NMR
Solvent Peak
0.68
2.06
1.16
1.75
2.27
1.00
1.03
1.02
3.23
1.03
3.17
6.37
9.23
f1 (ppm)

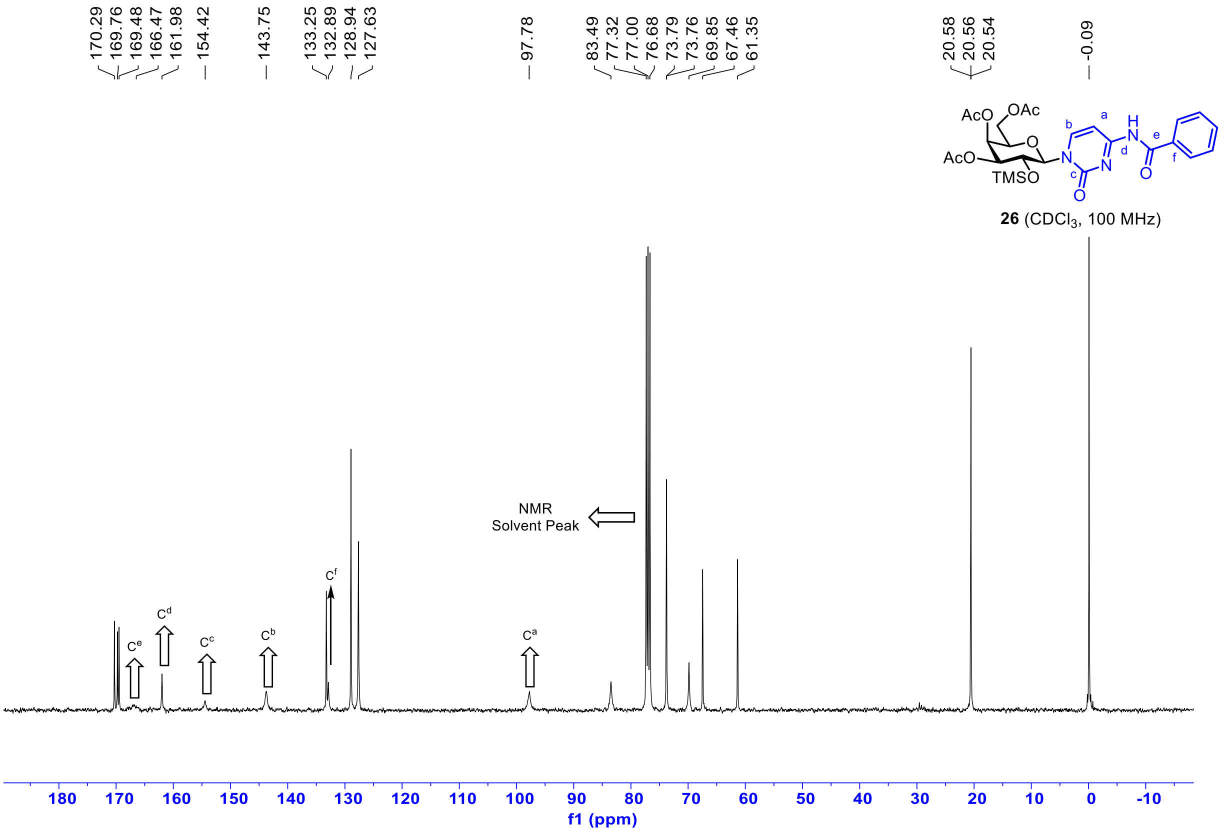
170.29 169.76 169.48 166.47 161.98
154.42
143.75
133.25 132.89 128.94 127.63
97.78
83.49 77.32 77.00 76.68 73.79 73.76 69.85 67.46 61.35
20.58 20.56 20.54
-0.09
AcO
OAc
O
b
a
H
N
e
d
f
N
c
N
AcO
TMSO
O
O
26 (CDCl3, 100 MHz)
Ce
Cd
Cc
Cb
Cf
Ca
NMR
Solvent Peak
f1 (ppm)

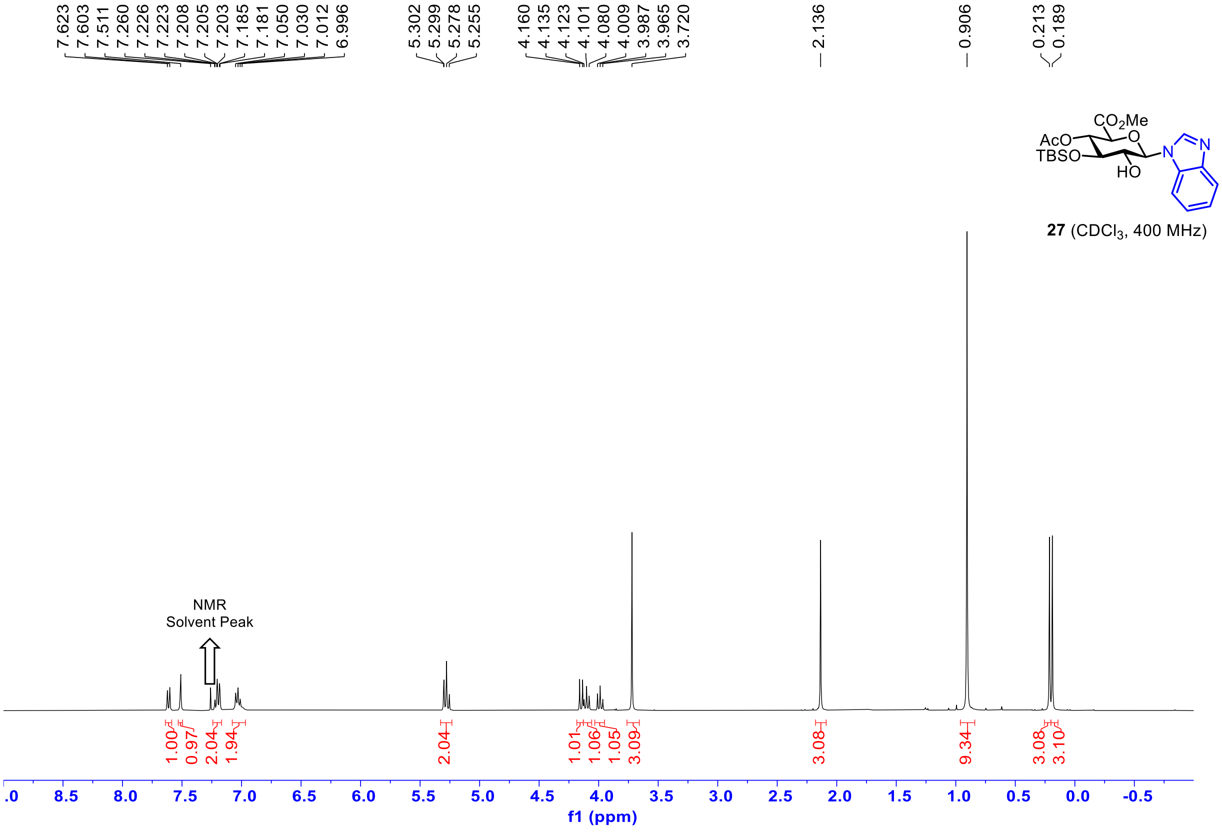

27 ($CDCl_3$, 400 MHz)

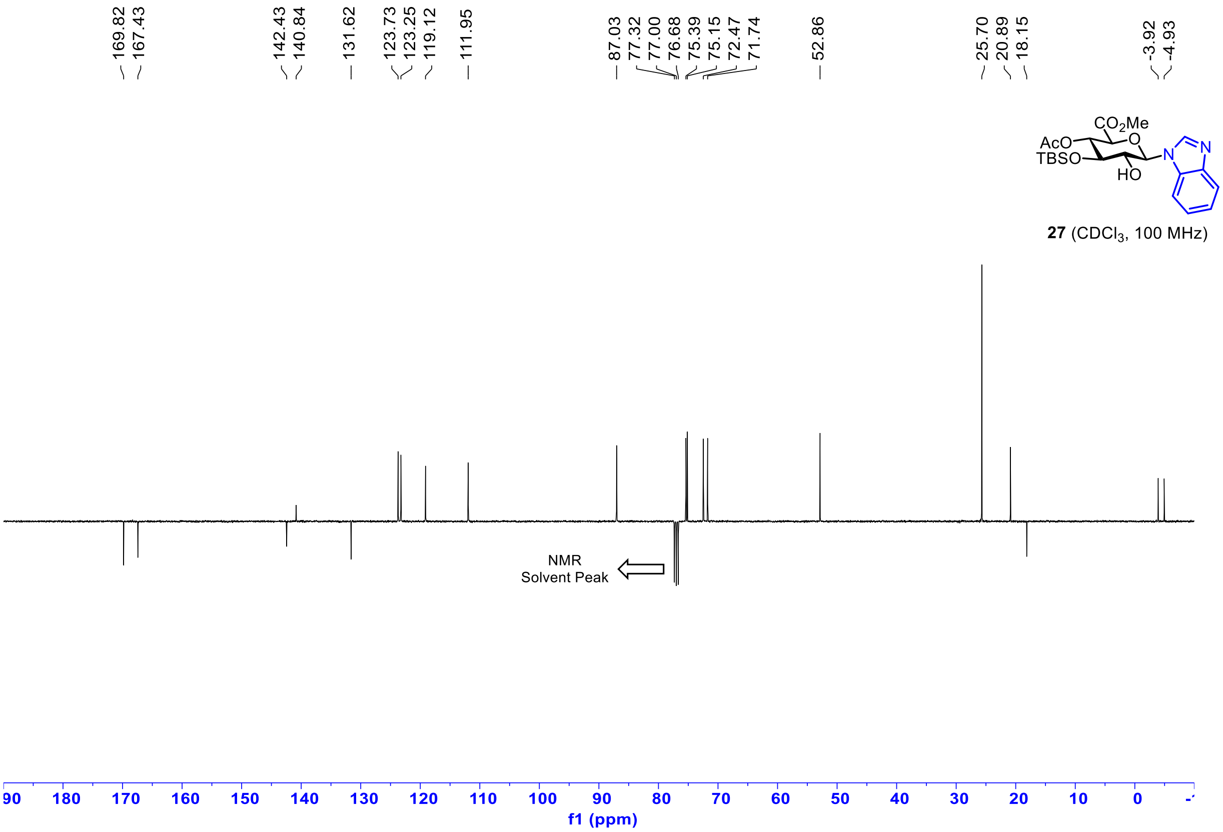

27 ($CDCl_3$, 100 MHz)

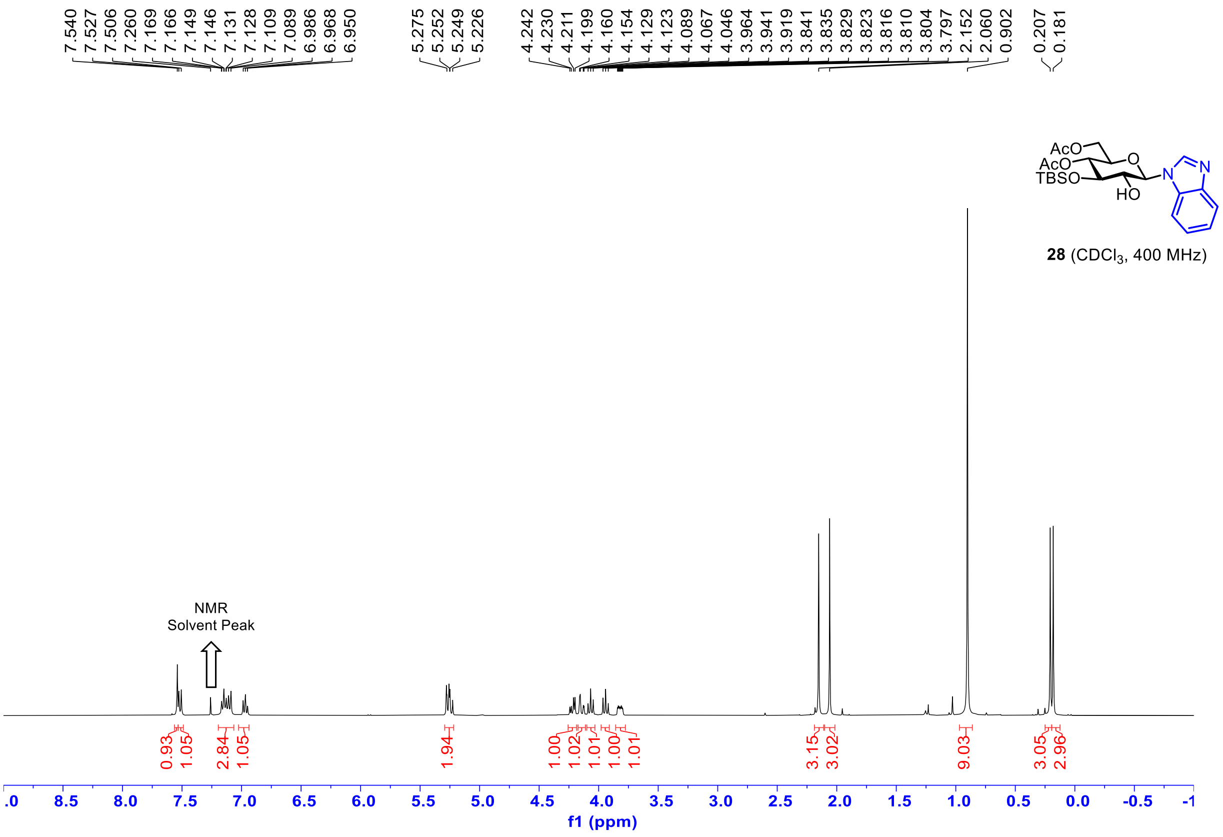
7.540
7.527
7.506
7.260
7.169
7.166
7.149
7.146
7.131
7.128
7.109
7.089
6.986
6.968
6.950
5.275
5.252
5.249
5.226
4.242
4.230
4.211
4.199
4.160
4.154
4.129
4.123
4.089
4.067
4.046
3.964
3.941
3.919
3.841
3.835
3.829
3.823
3.816
3.810
3.804
3.797
2.152
2.060
0.902
0.207
0.181
AcO
AcO
TBSO
O
N
N
HO
28 (CDCl3, 400 MHz)
NMR
Solvent Peak
0.93
1.05
2.84
1.05
1.94
1.00
1.02
1.01
1.00
1.01
3.15
3.02
9.03
3.05
2.96
f1 (ppm)

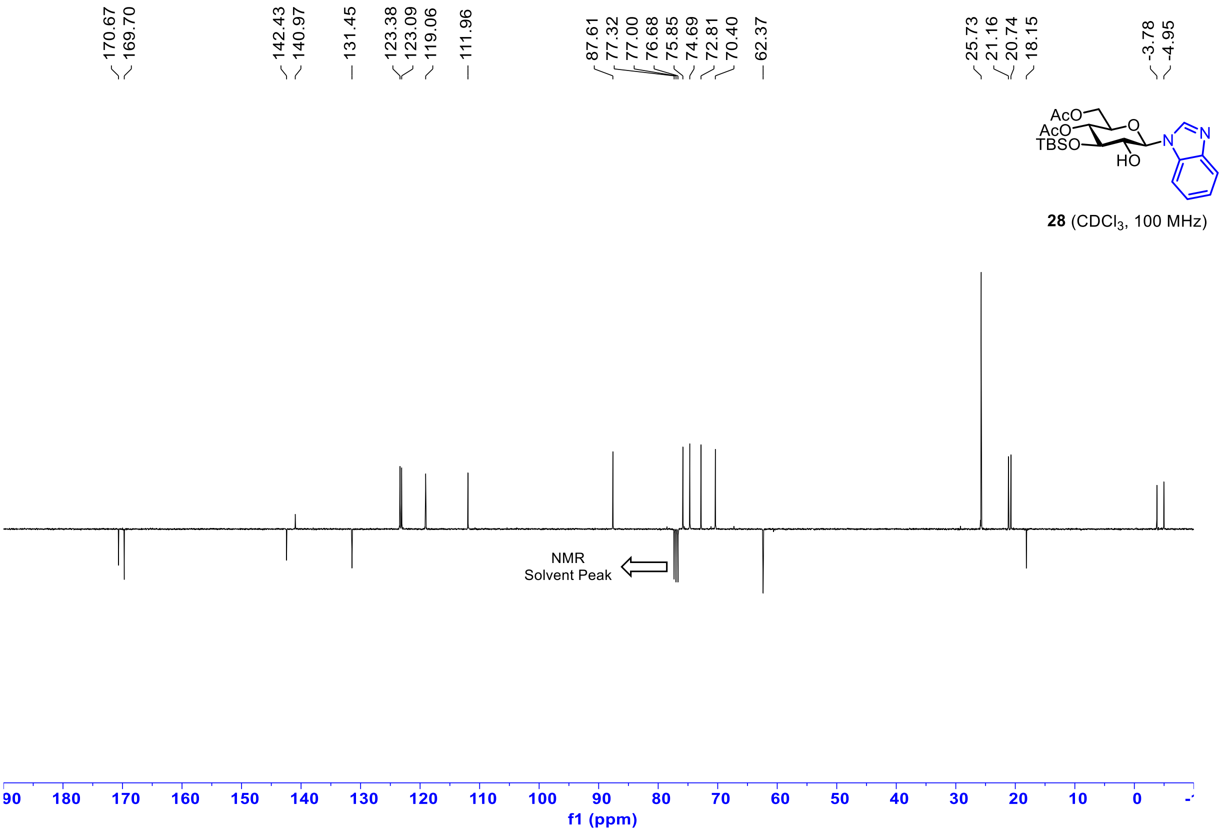
170.67
169.70
142.43
140.97
131.45
123.38
123.09
119.06
111.96
87.61
77.32
77.00
76.68
75.85
74.69
72.81
70.40
62.37
25.73
21.16
20.74
18.15
-3.78
-4.95
AcO
AcO
TBSO
O
N
N
HO
28 (CDCl3, 100 MHz)
NMR
Solvent Peak
f1 (ppm)

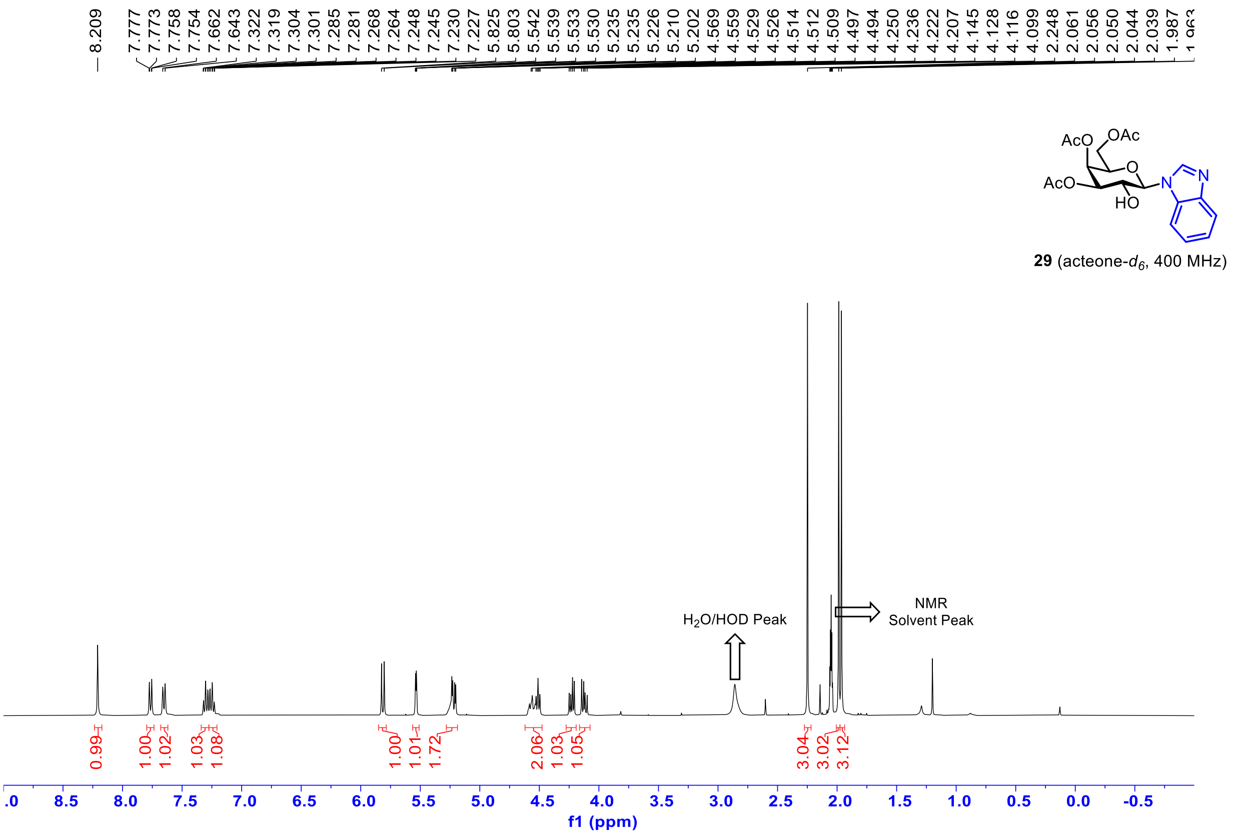

29 (acteone-d_6, 400 MHz)

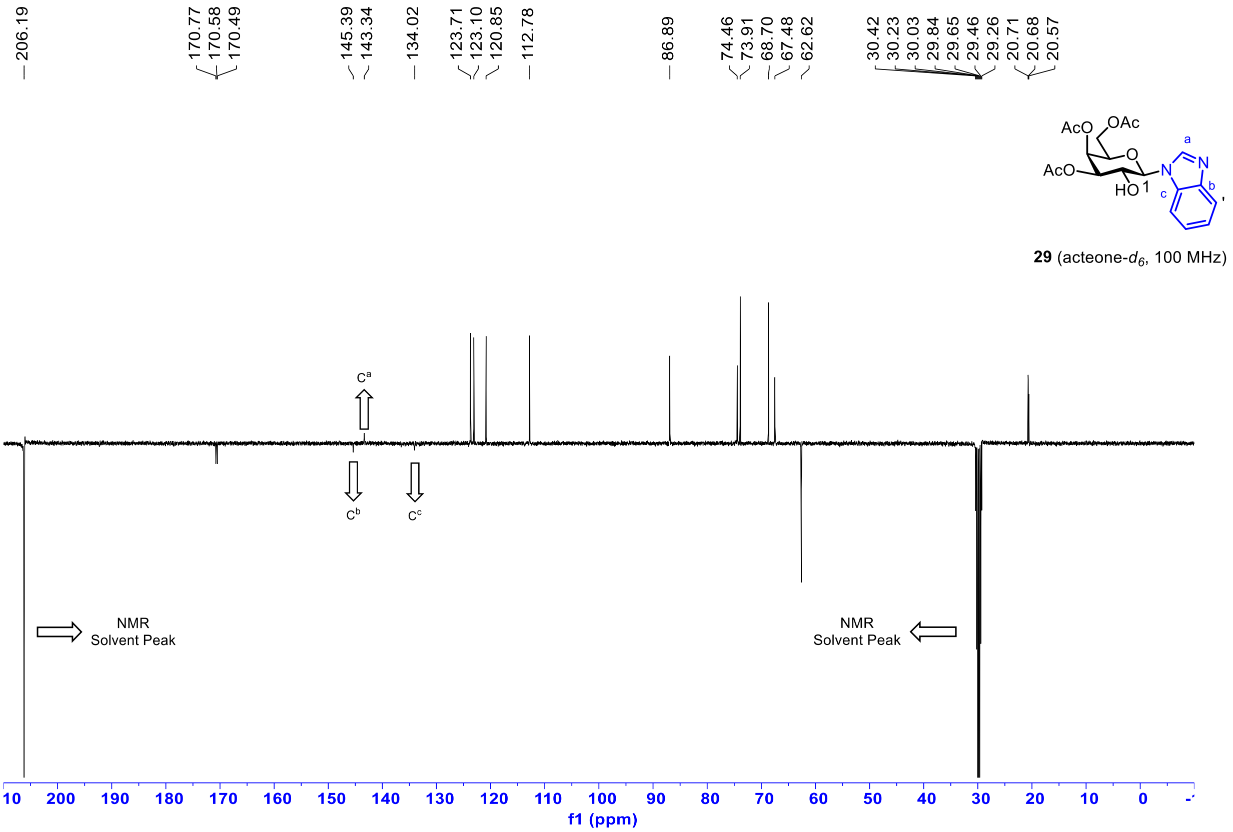

29 (acteone-d_6, 100 MHz)

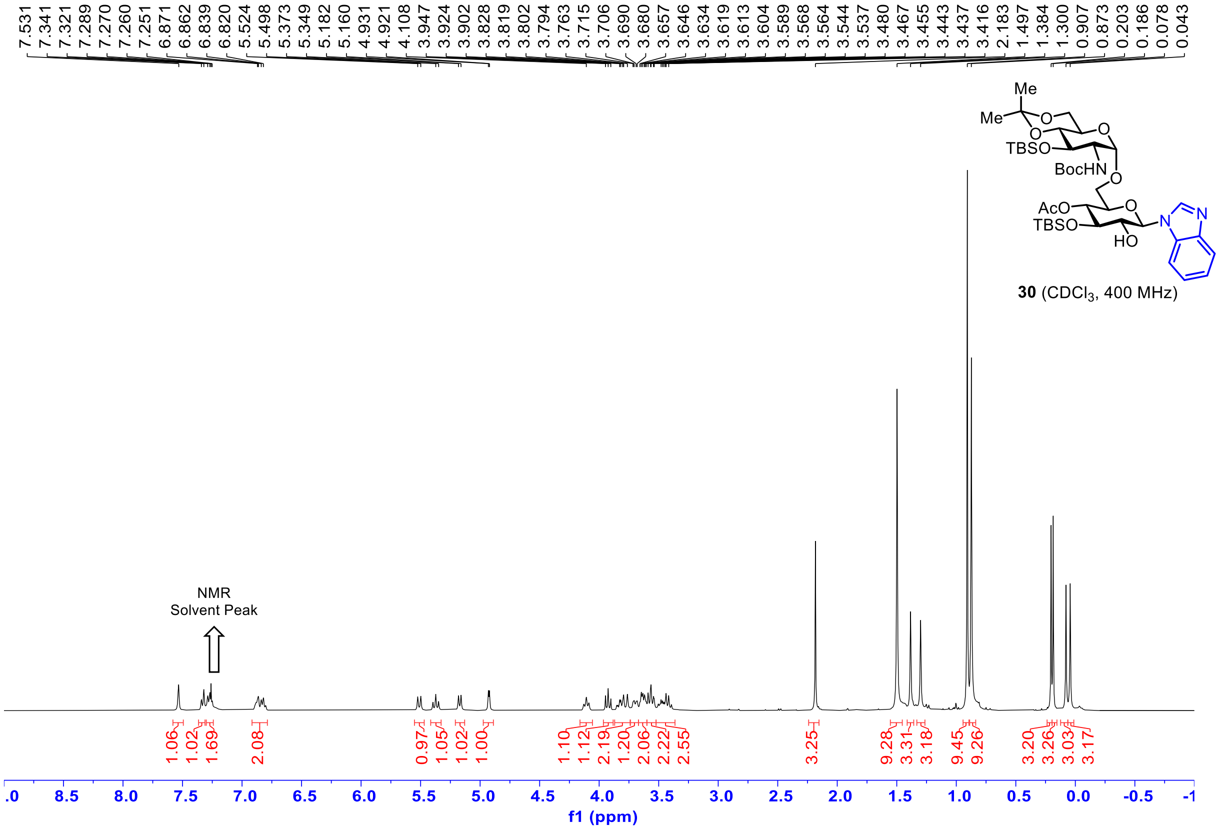
7.531 7.341 7.321 7.289 7.270 7.260 7.251 6.871 6.862 6.839 6.820 5.524 5.498 5.373 5.349 5.182 5.160 4.931 4.921 4.108 3.947 3.924 3.902 3.828 3.819 3.802 3.794 3.763 3.715 3.706 3.690 3.680 3.657 3.646 3.634 3.619 3.613 3.604 3.589 3.568 3.564 3.544 3.537 3.480 3.467 3.455 3.443 3.437 3.416 2.183 1.497 1.384 1.300 0.907 0.873 0.203 0.186 0.078 0.043
Me
Me
O
O
O
TBSO
BocHN
O
AcO
O
N
N
TBSO
HO
30 (CDCl3, 400 MHz)
NMR
Solvent Peak
1.06 1.02 1.69 2.08 0.97 1.05 1.02 1.00 1.10 1.12 2.19 1.20 2.06 2.22 2.55 3.25 9.28 3.31 3.18 9.45 9.26 3.20 3.26 3.03 3.17
.0 8.5 8.0 7.5 7.0 6.5 6.0 5.5 5.0 4.5 4.0 3.5 3.0 2.5 2.0 1.5 1.0 0.5 0.0 -0.5 -1
f1 (ppm)

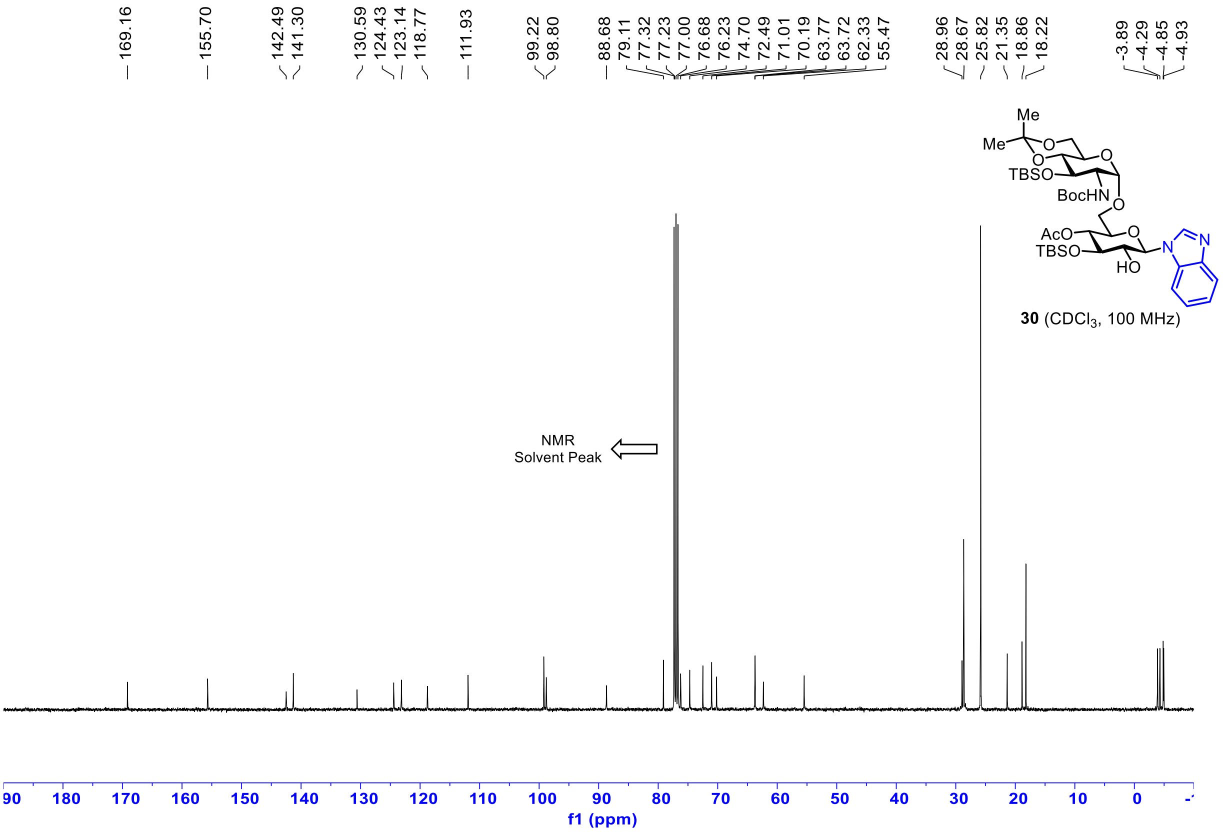
169.16 155.70 142.49 141.30 130.59 124.43 123.14 118.77 111.93 99.22 98.80 88.68 79.11 77.32 77.23 77.00 76.68 76.23 74.70 72.49 71.01 70.19 63.77 63.72 62.33 55.47 28.96 28.67 25.82 21.35 18.86 18.22 -3.89 -4.29 -4.85 -4.93
Me
Me
O
O
O
TBSO
BocHN
O
AcO
O
N
N
TBSO
HO
30 (CDCl3, 100 MHz)
NMR
Solvent Peak
90 180 170 160 150 140 130 120 110 100 90 80 70 60 50 40 30 20 10 0
f1 (ppm)

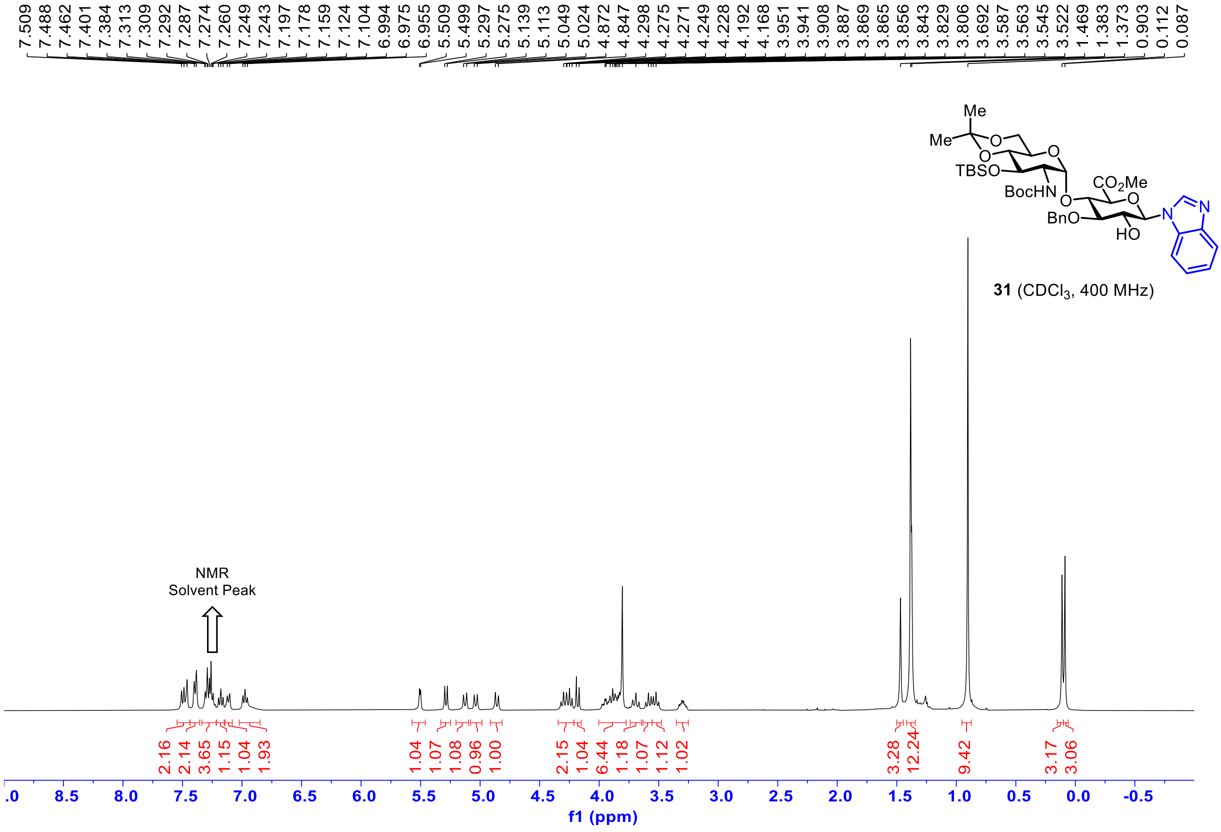
7.509 7.488 7.462 7.401 7.384 7.313 7.309 7.292 7.287 7.274 7.260 7.249 7.243 7.197 7.178 7.159 7.124 7.104 6.994 6.975 6.955 5.509 5.499 5.297 5.275 5.139 5.113 5.049 5.024 4.872 4.847 4.298 4.275 4.271 4.249 4.228 4.192 4.168 3.951 3.941 3.908 3.887 3.869 3.865 3.856 3.843 3.829 3.806 3.692 3.587 3.563 3.545 3.522 1.469 1.383 1.373 0.903 0.112 0.087
Me
Me
O
O
O
TBSO
BocHN
O
CO_2Me
O
N
N
BnO
HO
31 ($CDCl_3$, 400 MHz)
NMR
Solvent Peak
2.16 2.14 3.65 1.15 1.04 1.93 1.04 1.07 1.08 0.96 1.00 2.15 1.04 6.44 1.18 1.07 1.12 1.02 3.28 12.24 9.42 3.17 3.06
f1 (ppm)

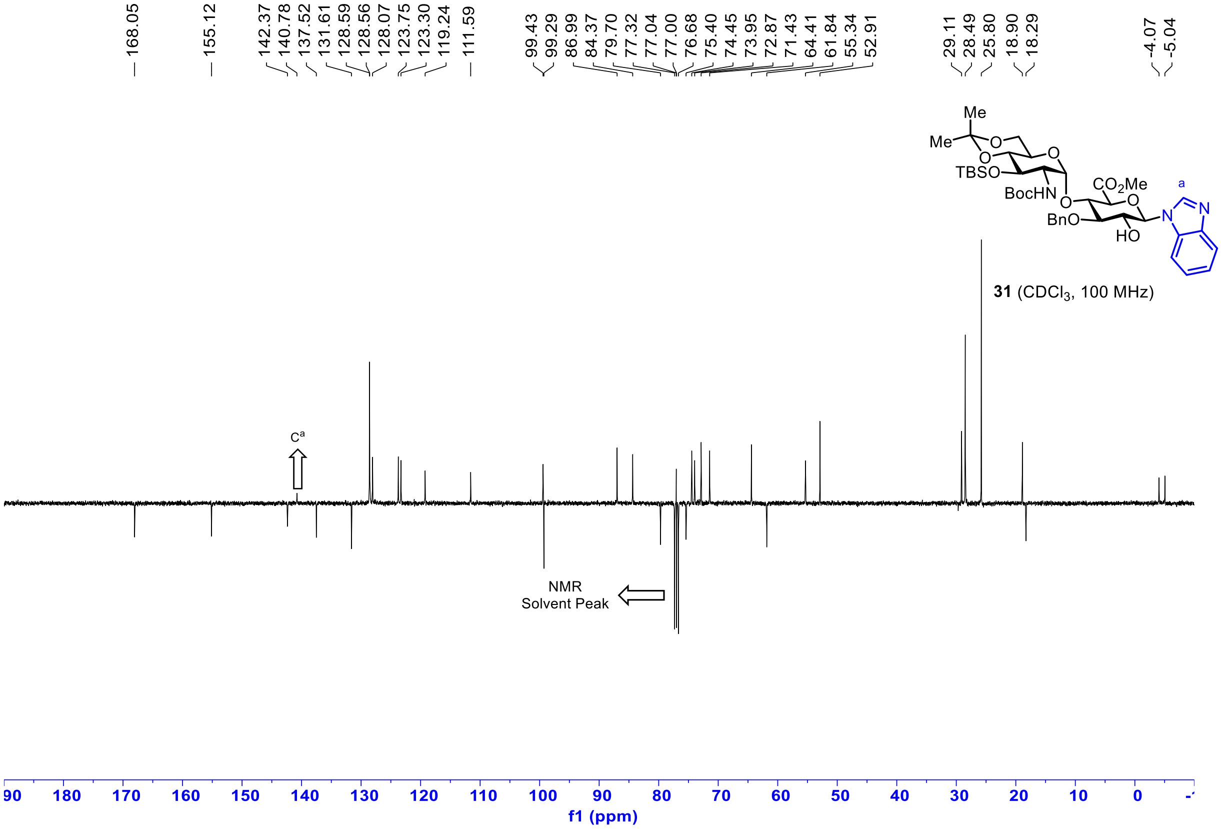
168.05 155.12 142.37 140.78 137.52 131.61 128.59 128.56 128.07 123.75 123.30 119.24 111.59 99.43 99.29 86.99 84.37 79.70 77.32 77.04 77.00 76.68 75.40 74.45 73.95 72.87 71.43 64.41 61.84 55.34 52.91 29.11 28.49 25.80 18.90 18.29 -4.07 -5.04
Me
Me
O
O
O
TBSO
BocHN
O
CO_2Me
O
a
N
N
BnO
HO
31 ($CDCl_3$, 100 MHz)
C[a]
NMR
Solvent Peak
f1 (ppm)

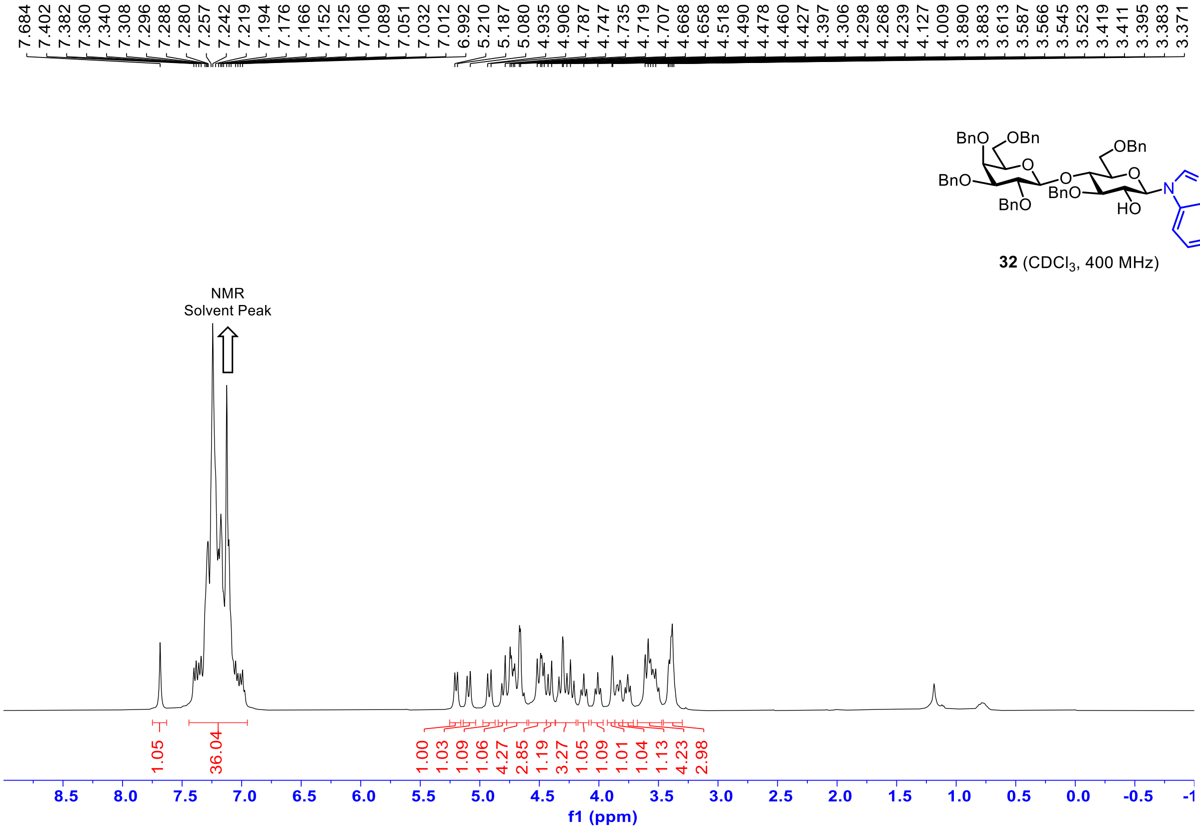

32 ($CDCl_3$, 400 MHz)

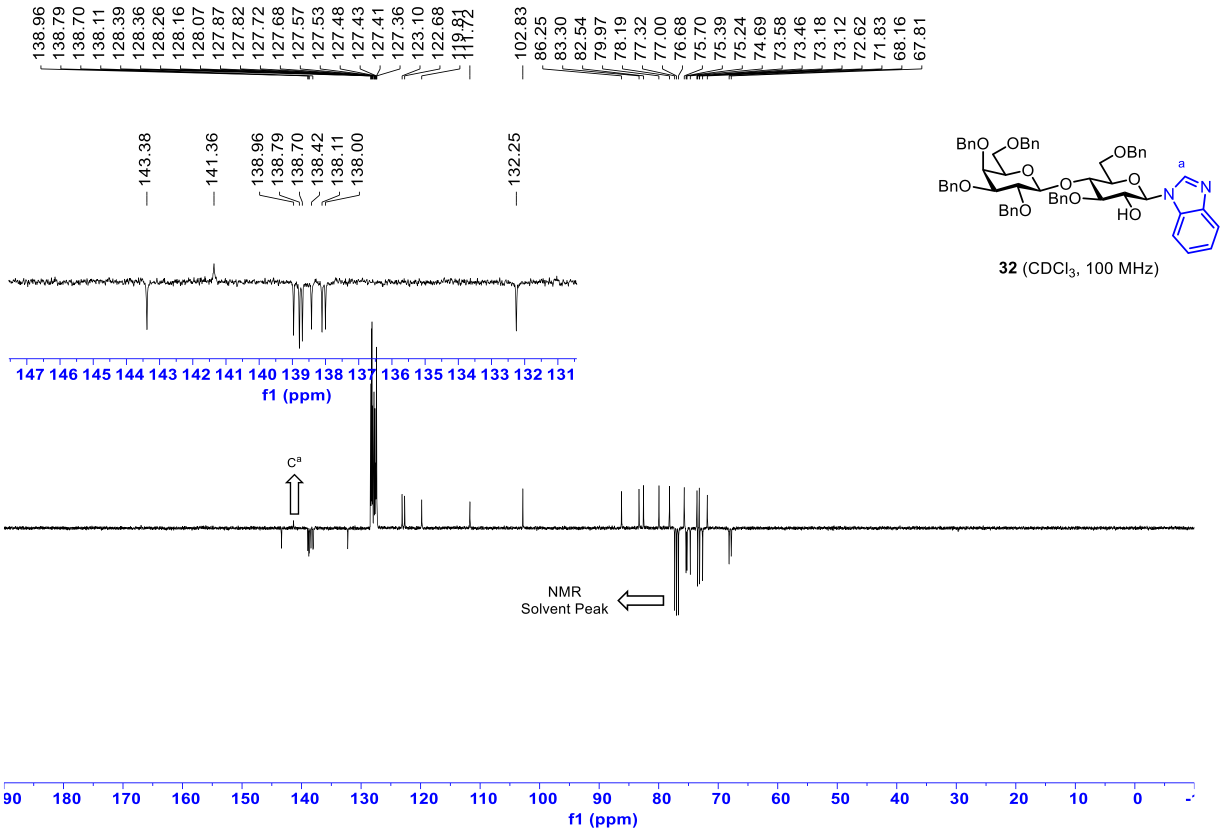

32 ($CDCl_3$, 100 MHz)

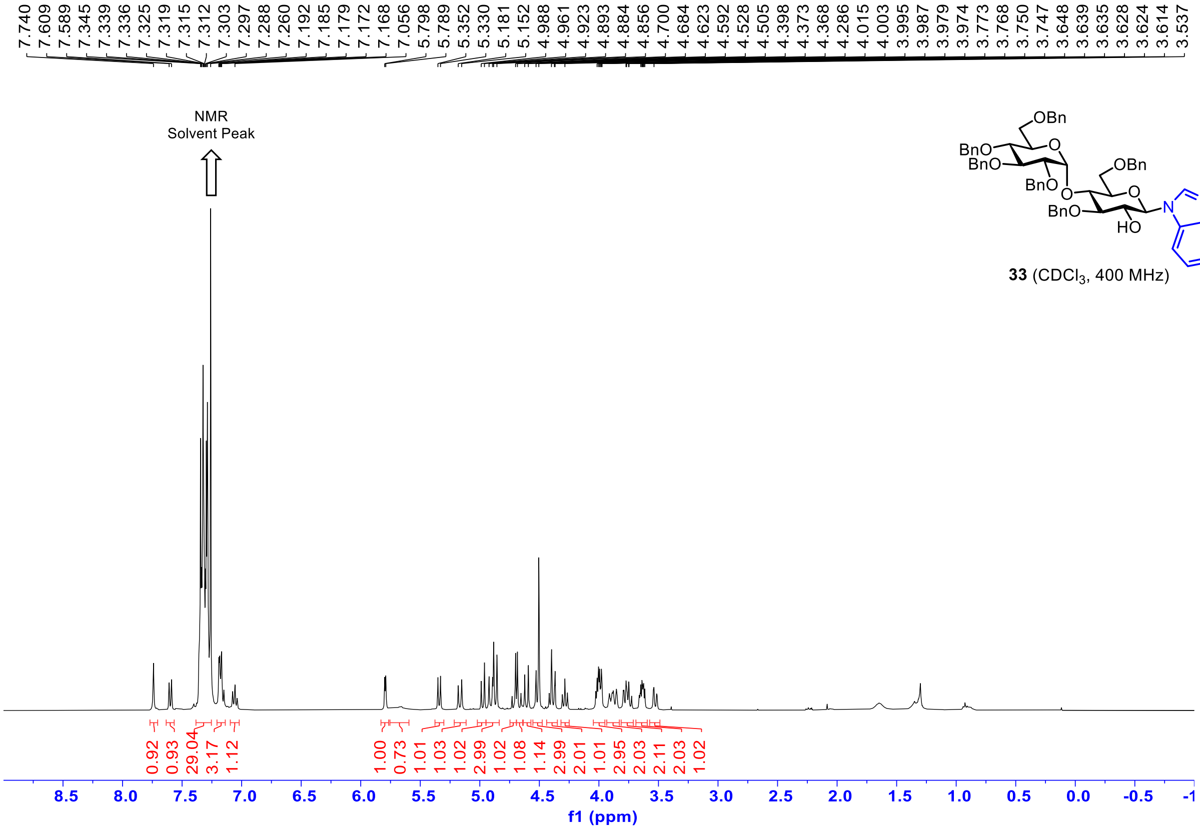
7.740 7.609 7.589 7.345 7.339 7.336 7.325 7.319 7.315 7.312 7.303 7.297 7.288 7.260 7.192 7.185 7.179 7.172 7.168 7.056 5.798 5.789 5.352 5.330 5.181 5.152 4.988 4.961 4.923 4.893 4.884 4.856 4.700 4.684 4.623 4.592 4.528 4.505 4.398 4.373 4.368 4.286 4.015 4.003 3.995 3.987 3.979 3.974 3.773 3.768 3.750 3.747 3.648 3.639 3.635 3.628 3.624 3.614 3.537
NMR
Solvent Peak
OBn
BnO
BnO
BnO
O
OBn
O
N
N
BnO
HO
33 (CDCl3, 400 MHz)
0.92 0.93 29.04 3.17 1.12 1.00 0.73 1.01 1.03 1.02 2.99 1.02 1.08 1.14 2.99 2.01 1.01 2.95 2.03 2.11 2.03 1.02
8.5 8.0 7.5 7.0 6.5 6.0 5.5 5.0 4.5 4.0 3.5 3.0 2.5 2.0 1.5 1.0 0.5 0.0 -0.5 -1
f1 (ppm)

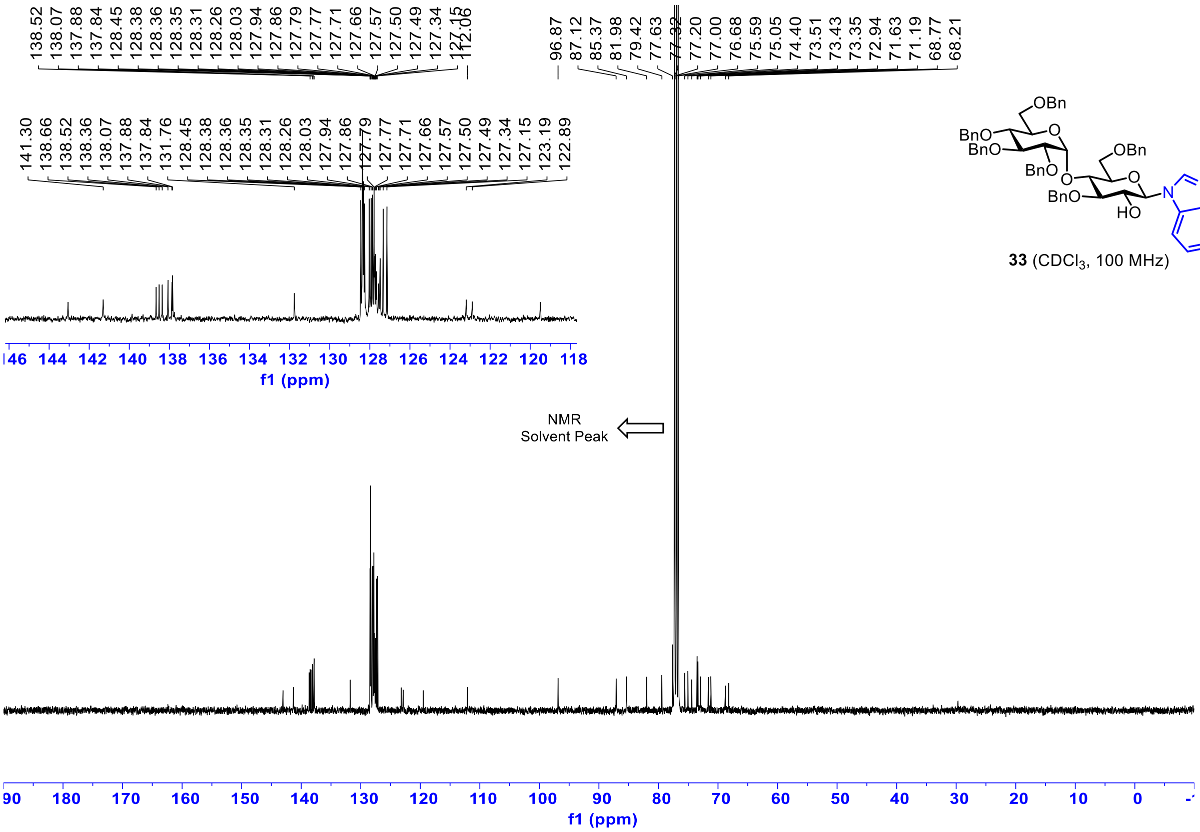
138.52 138.07 137.88 137.84 128.45 128.38 128.36 128.35 128.31 128.26 128.03 127.94 127.86 127.79 127.77 127.71 127.66 127.57 127.50 127.49 127.34 127.15 112.06
96.87 87.12 85.37 81.98 79.42 77.63 77.32 77.20 77.00 76.68 75.59 75.05 74.40 73.51 73.43 73.35 72.94 71.63 71.19 68.77 68.21
141.30 138.66 138.52 138.36 138.07 137.88 137.84 131.76 128.45 128.38 128.36 128.35 128.31 128.26 128.03 127.94 127.86 127.79 127.77 127.71 127.66 127.57 127.50 127.49 127.34 127.15 123.19 122.89
146 144 142 140 138 136 134 132 130 128 126 124 122 120 118
f1 (ppm)
OBn
BnO
BnO
BnO
O
OBn
O
N
N
BnO
HO
33 (CDCl3, 100 MHz)
NMR
Solvent Peak
90 180 170 160 150 140 130 120 110 100 90 80 70 60 50 40 30 20 10 0
f1 (ppm)

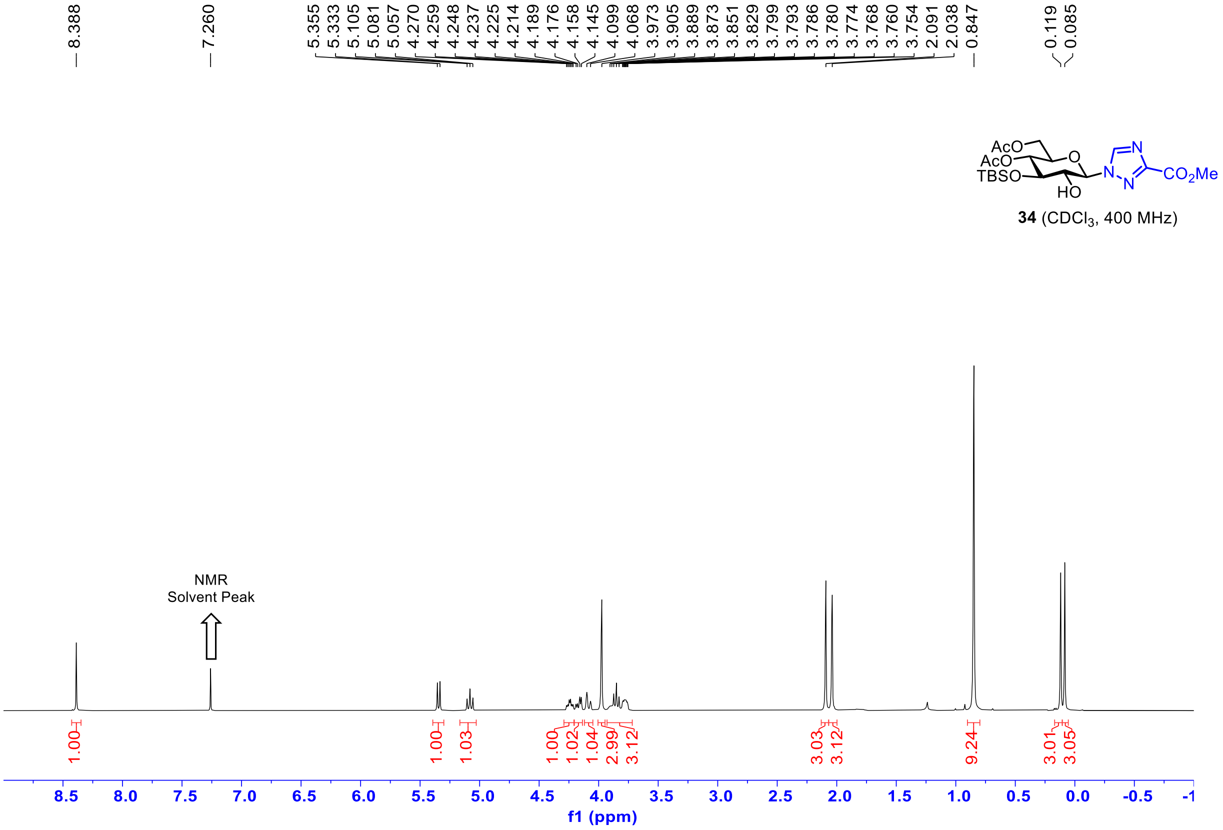

34 ($CDCl_3$, 400 MHz)

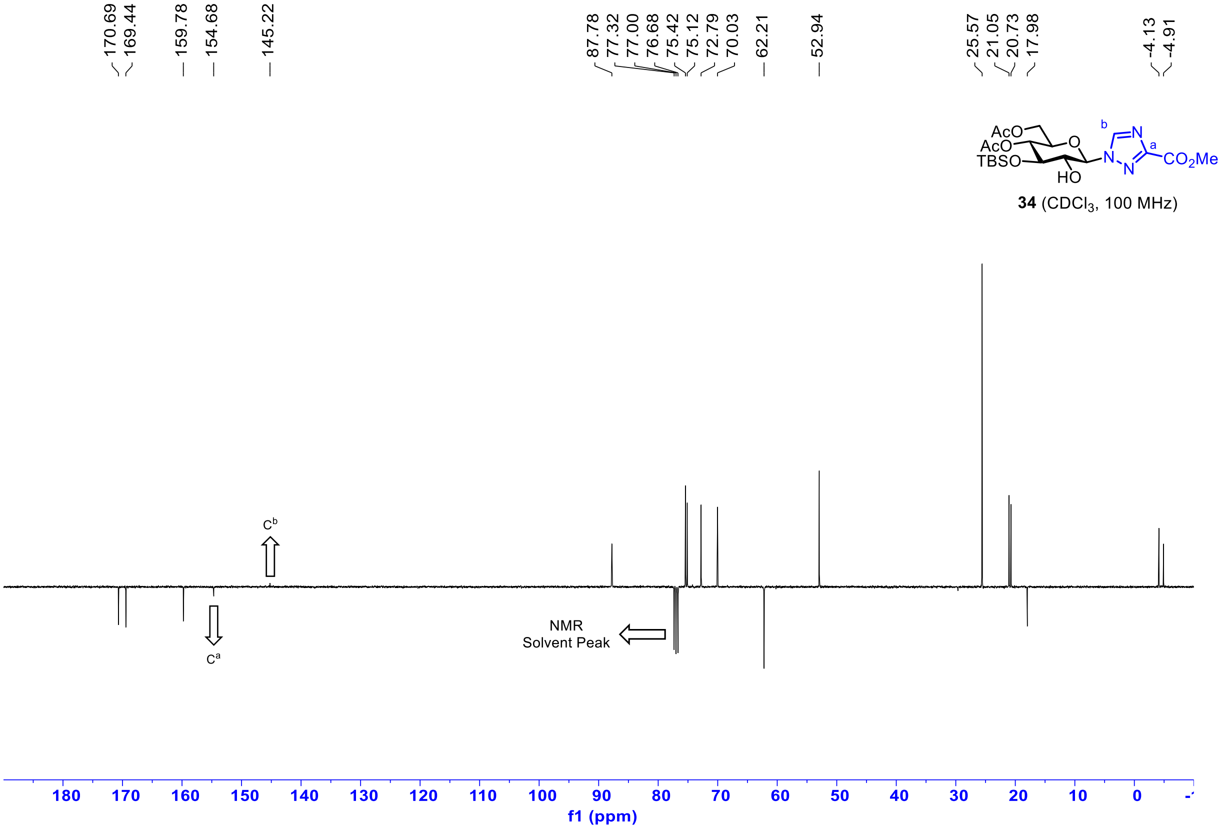

34 ($CDCl_3$, 100 MHz)

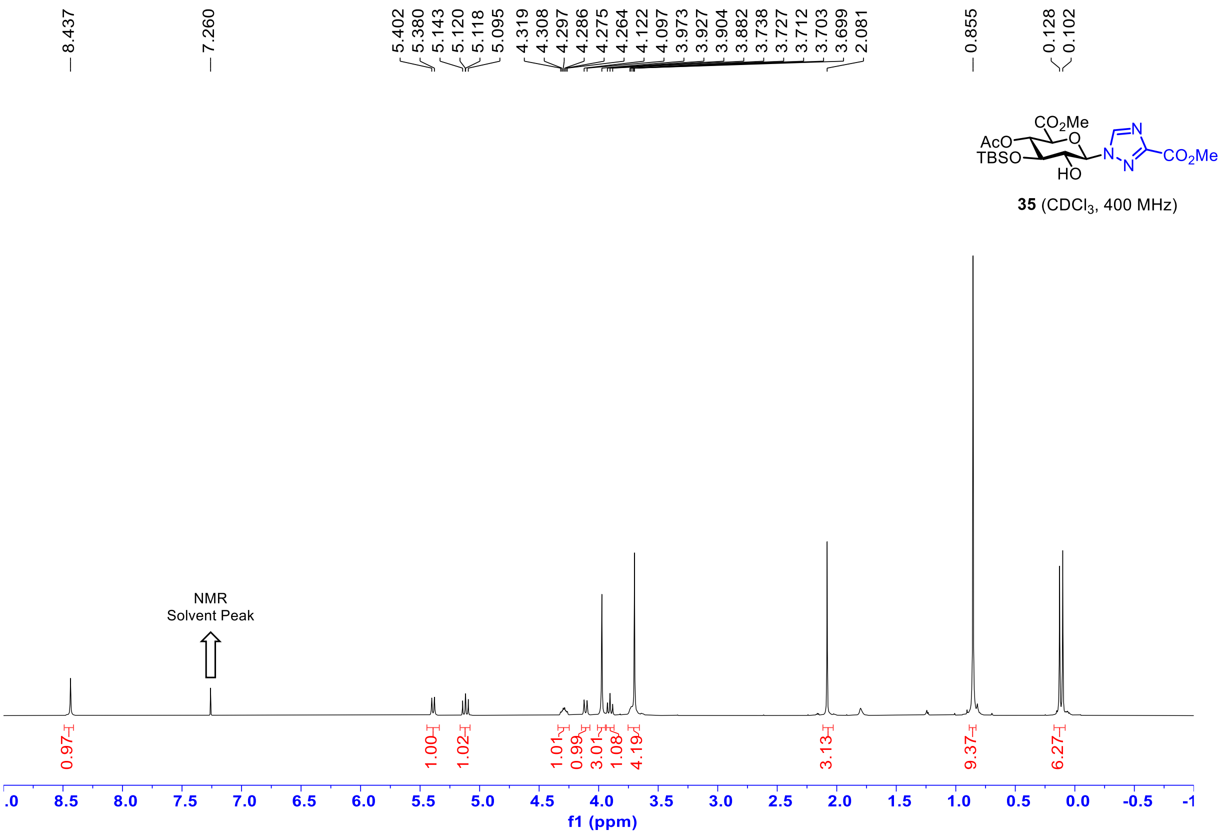

8.437
7.260
5.402
5.380
5.143
5.120
5.118
5.095
4.319
4.308
4.297
4.286
4.275
4.264
4.122
4.097
3.973
3.927
3.904
3.882
3.738
3.727
3.712
3.703
3.699
2.081
0.855
0.128
0.102
CO_2Me
AcO
TBSO
HO
N
CO_2Me
35 ($CDCl_3$, 400 MHz)
NMR
Solvent Peak
0.97
1.00
1.02
1.01
0.99
3.01
1.08
4.19
3.13
9.37
6.27
f1 (ppm)

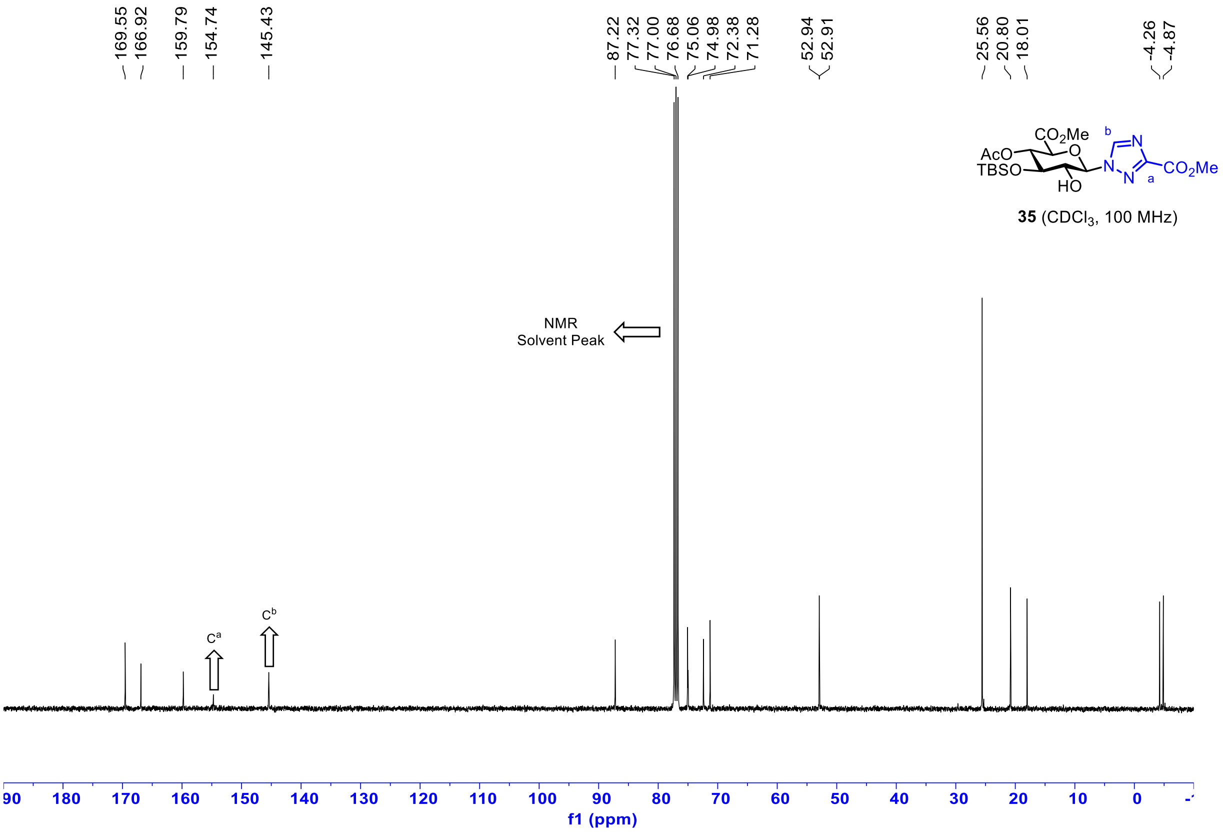

169.55
166.92
159.79
154.74
145.43
87.22
77.32
77.00
76.68
75.06
74.98
72.38
71.28
52.94
52.91
25.56
20.80
18.01
-4.26
-4.87
CO_2Me
AcO
TBSO
HO
CO_2Me
35 ($CDCl_3$, 100 MHz)
NMR
Solvent Peak
C^a
C^b
f1 (ppm)

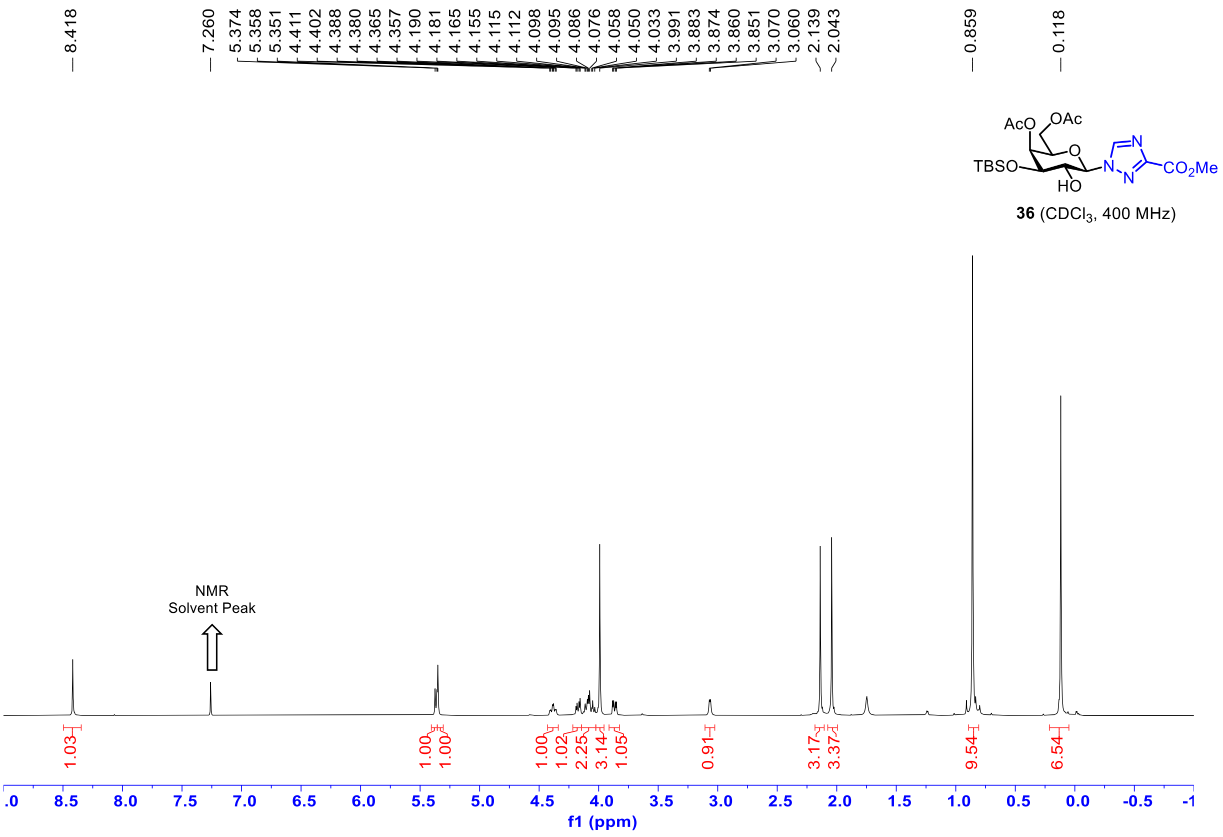
8.418
7.260
5.374
5.358
5.351
4.411
4.402
4.388
4.380
4.365
4.357
4.190
4.181
4.165
4.155
4.115
4.112
4.098
4.095
4.086
4.076
4.058
4.050
4.033
3.991
3.883
3.874
3.860
3.851
3.070
3.060
2.139
2.043
0.859
0.118
AcO
OAc
TBSO
HO
O
N
N
N
CO2Me
36 (CDCl3, 400 MHz)
NMR Solvent Peak
1.03
1.00
1.00
1.00
1.02
2.25
3.14
1.05
0.91
3.17
3.37
9.54
6.54
f1 (ppm)

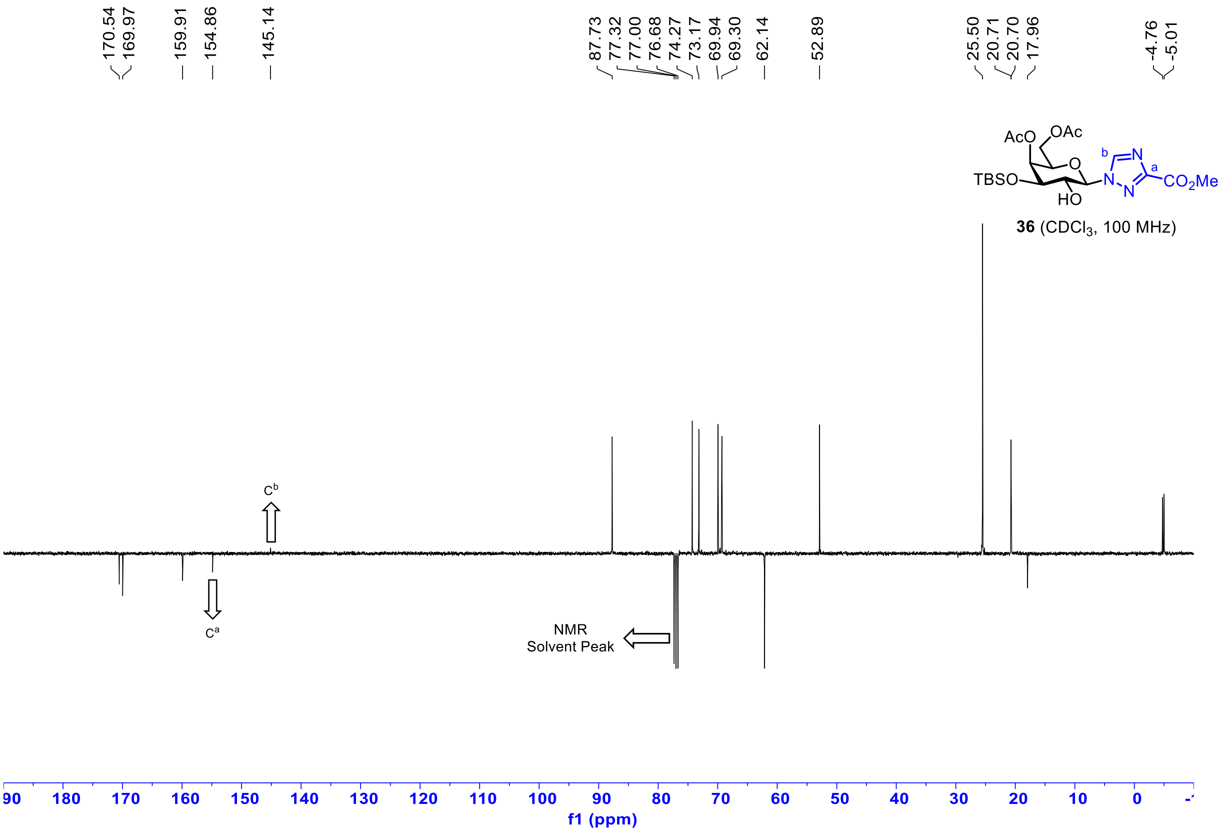
170.54
169.97
159.91
154.86
145.14
87.73
77.32
77.00
76.68
74.27
73.17
69.94
69.30
62.14
52.89
25.50
20.71
20.70
17.96
-4.76
-5.01
AcO
OAc
TBSO
HO
b
a
CO2Me
36 (CDCl3, 100 MHz)
Cb
Ca
NMR Solvent Peak
f1 (ppm)

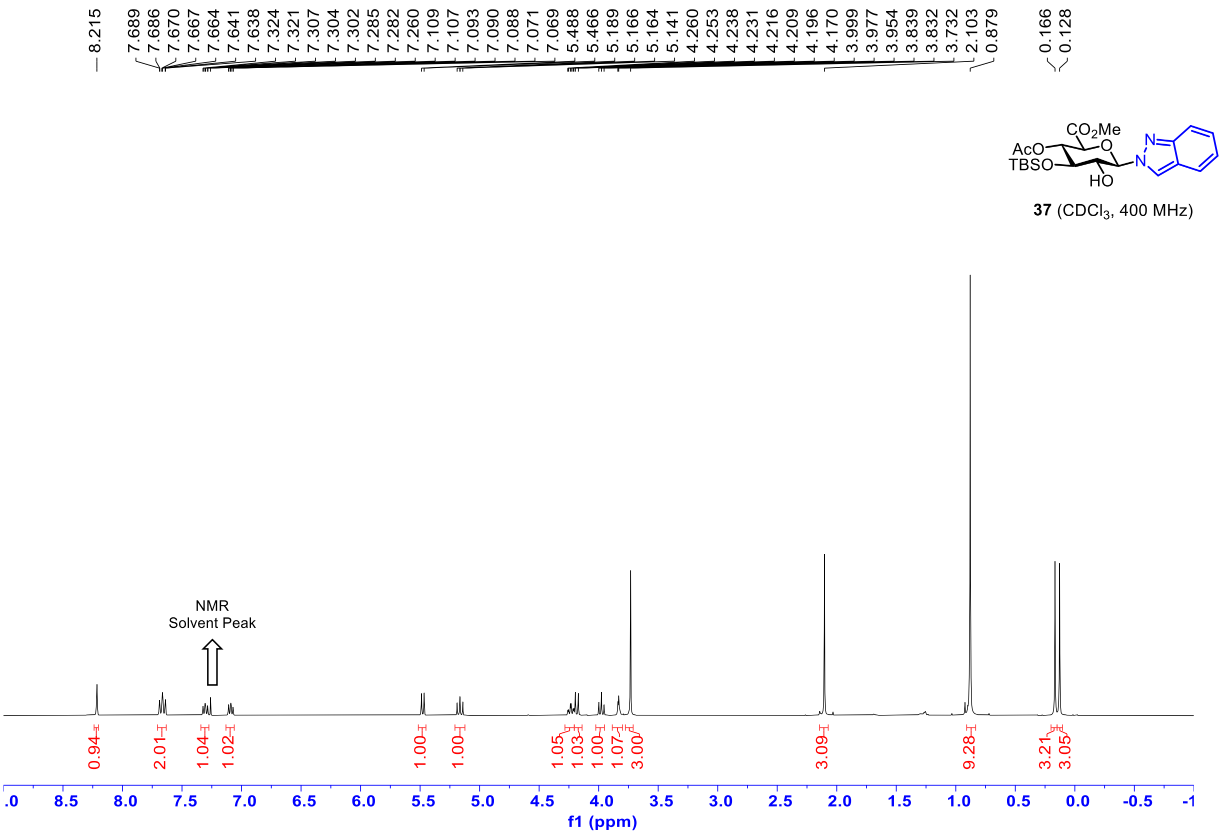
8.215
7.689
7.686
7.670
7.667
7.664
7.641
7.638
7.324
7.321
7.307
7.304
7.302
7.285
7.282
7.260
7.109
7.107
7.093
7.090
7.088
7.071
7.069
5.488
5.466
5.189
5.166
5.164
5.141
4.260
4.253
4.238
4.231
4.216
4.209
4.196
4.170
3.999
3.977
3.954
3.839
3.832
3.732
2.103
0.879
0.166
0.128
CO_2Me
AcO
TBSO
O
HO
N
N
37 ($CDCl_3$, 400 MHz)
NMR
Solvent Peak
0.94
2.01
1.04
1.02
1.00
1.00
1.05
1.03
1.00
1.07
3.00
3.09
9.28
3.21
3.05
f1 (ppm)

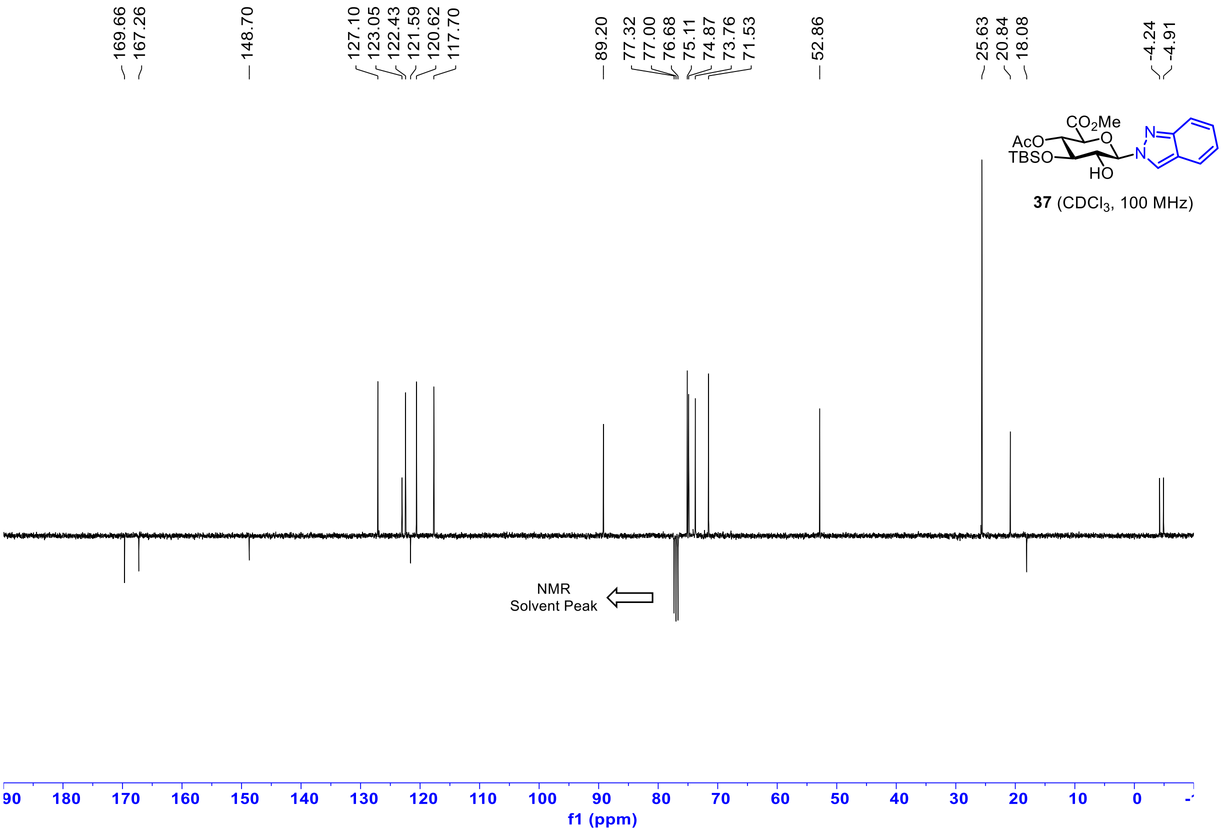
169.66
167.26
148.70
127.10
123.05
122.43
121.59
120.62
117.70
89.20
77.32
77.00
76.68
75.11
74.87
73.76
71.53
52.86
25.63
20.84
18.08
-4.24
-4.91
CO_2Me
AcO
TBSO
O
HO
N
N
37 ($CDCl_3$, 100 MHz)
NMR
Solvent Peak
f1 (ppm)

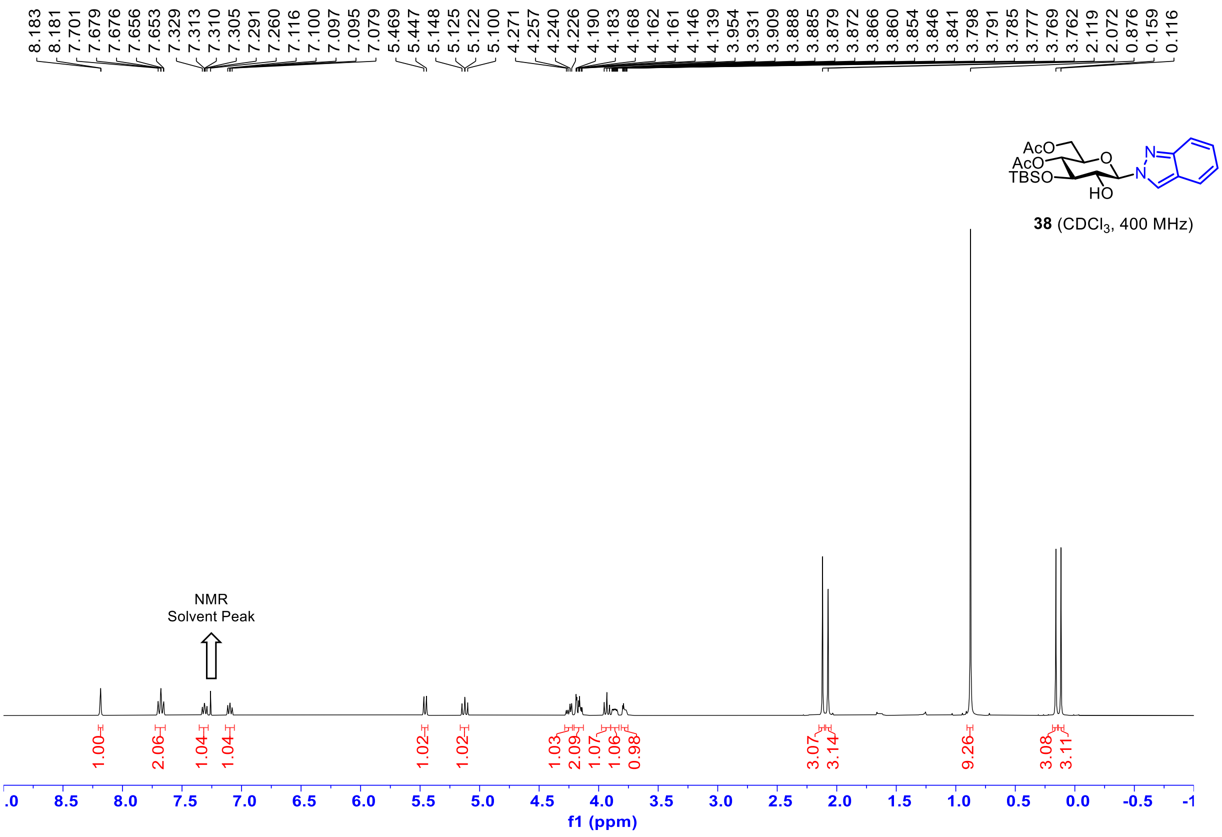

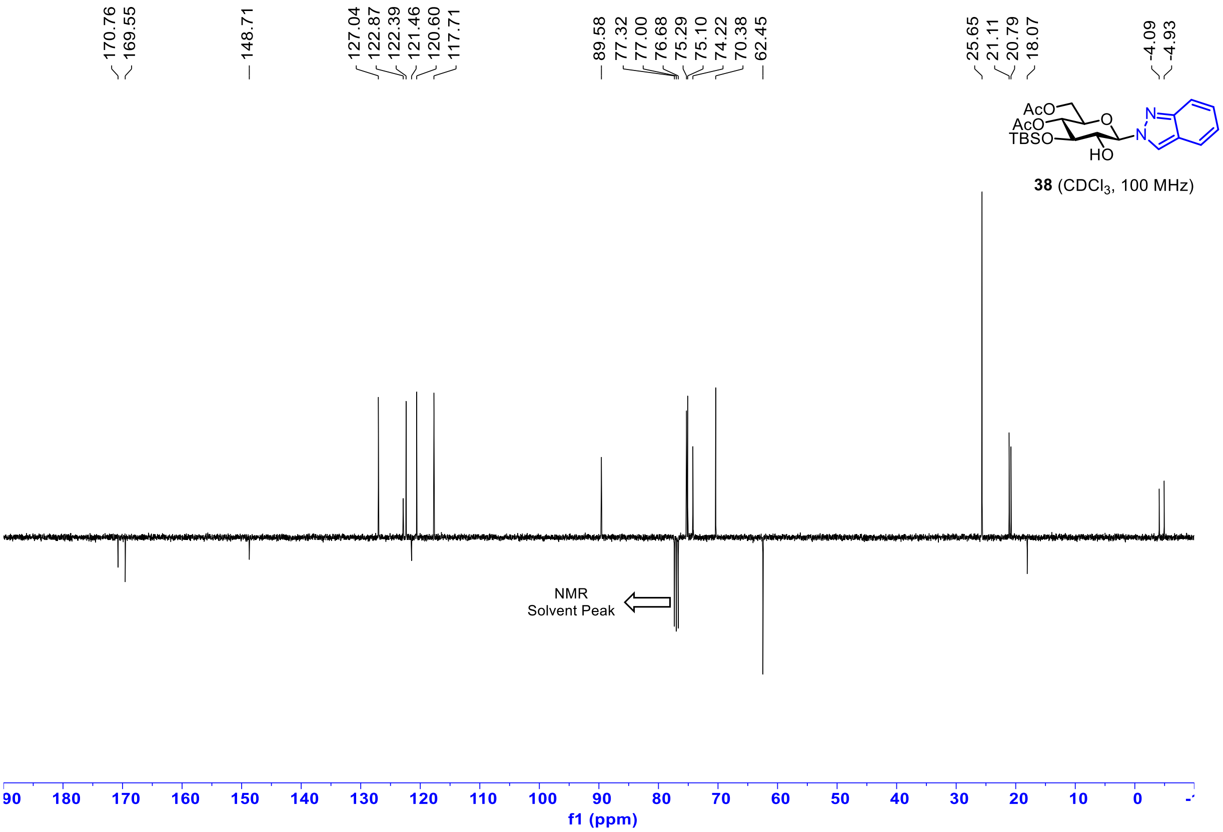

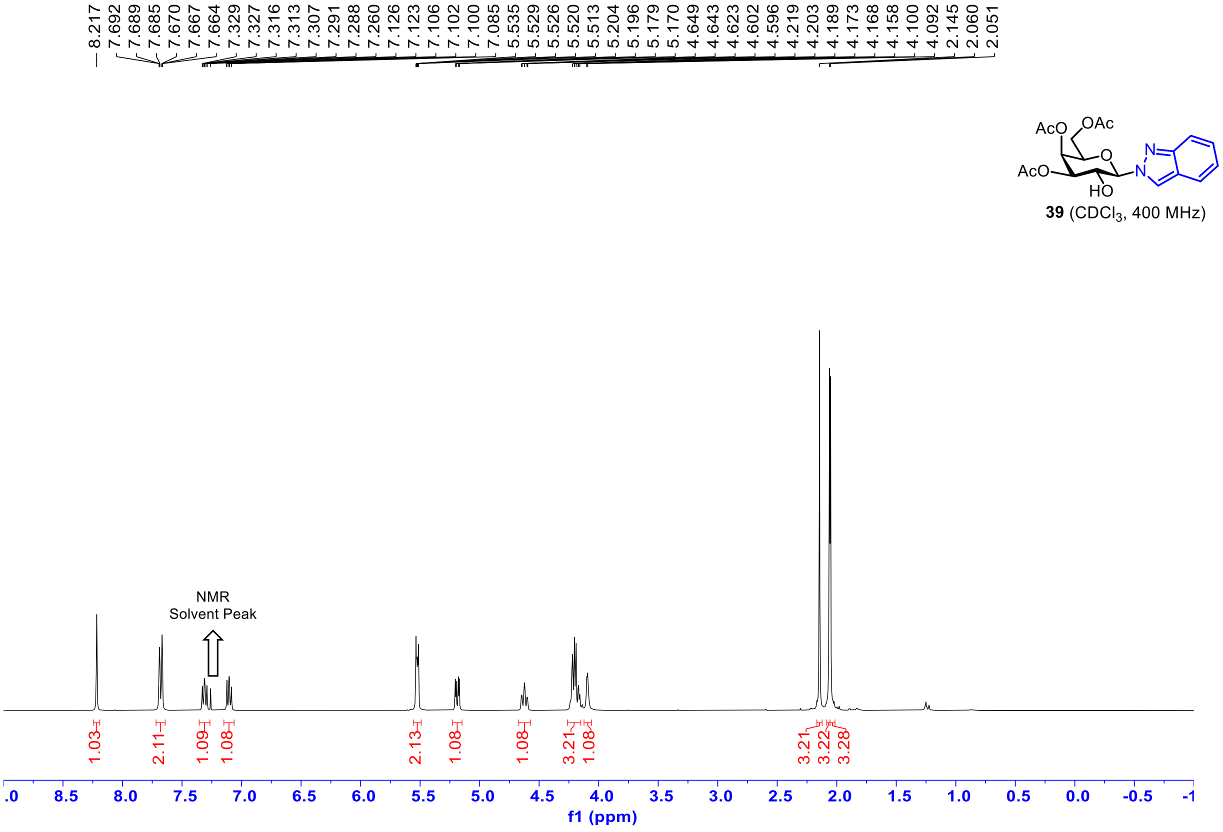

39 ($CDCl_3$, 400 MHz)

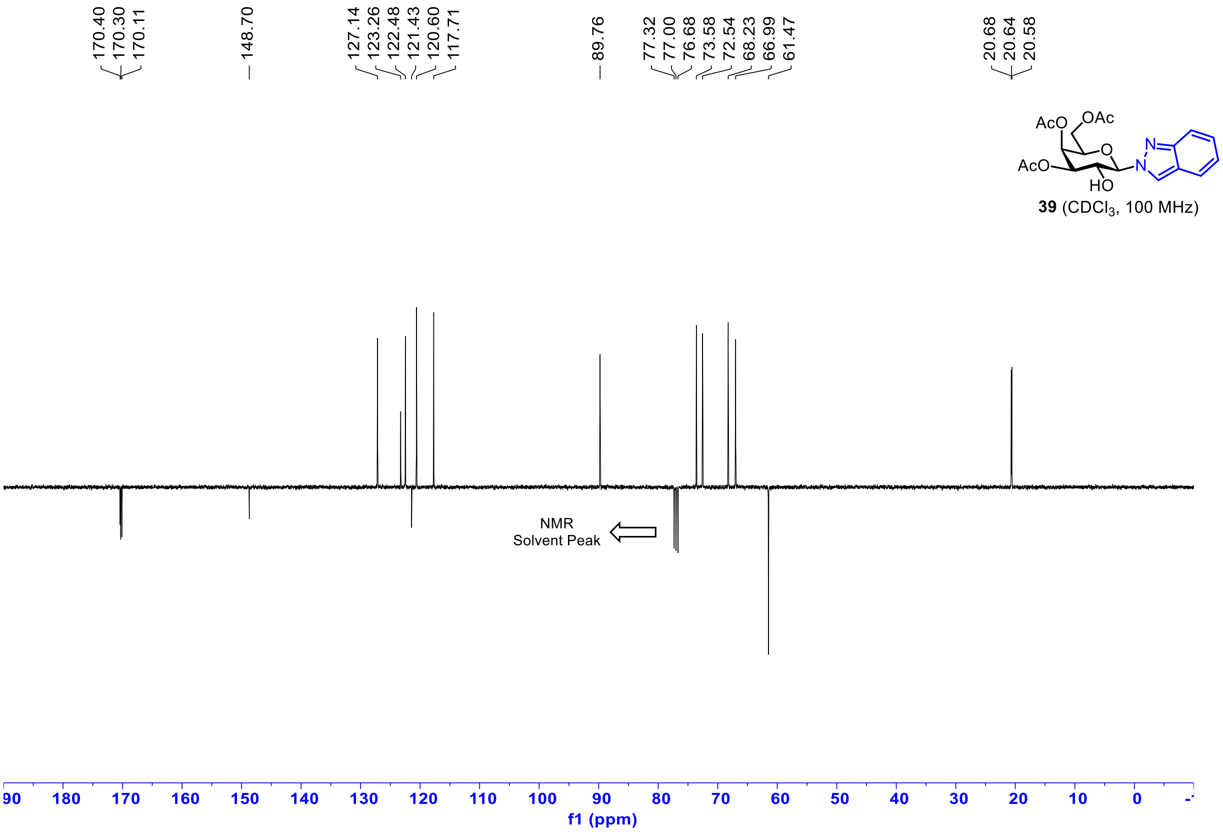

39 ($CDCl_3$, 100 MHz)

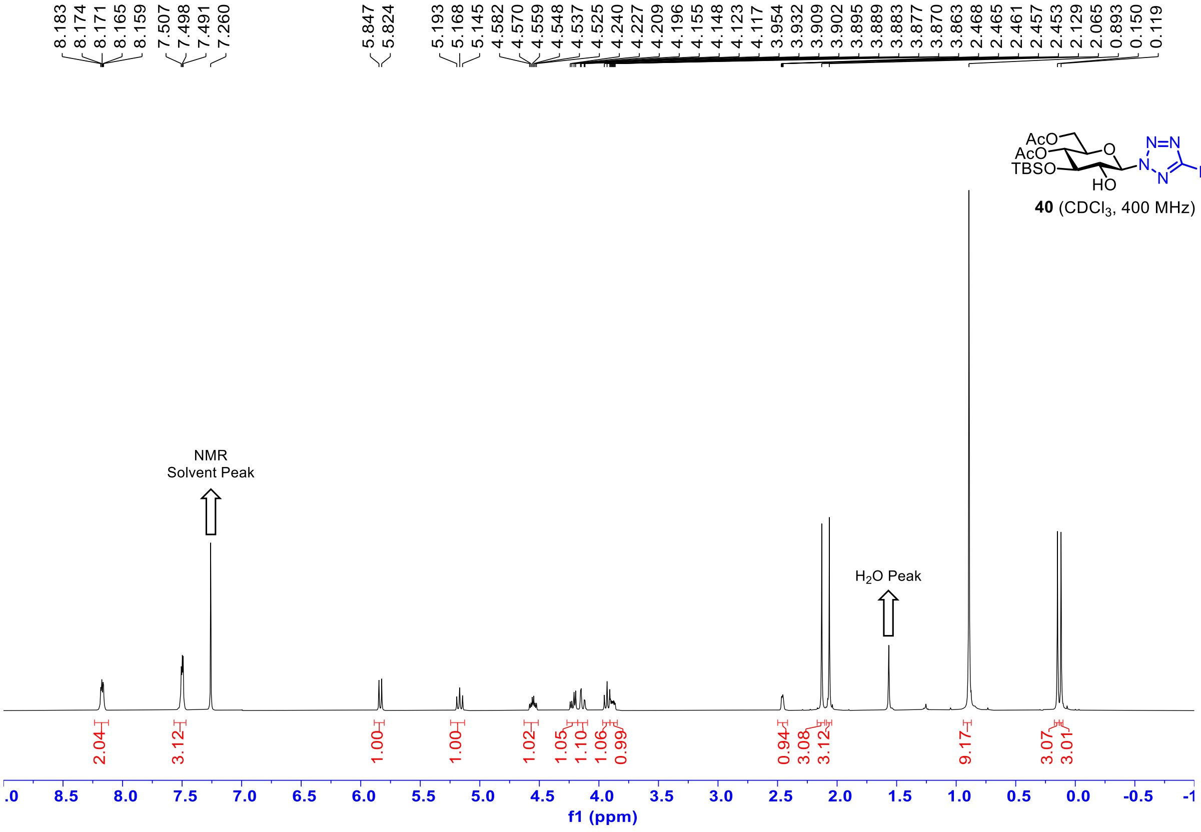
8.183
8.174
8.171
8.165
8.159
7.507
7.498
7.491
7.260
5.847
5.824
5.193
5.168
5.145
4.582
4.570
4.559
4.548
4.537
4.525
4.240
4.227
4.209
4.196
4.155
4.148
4.123
4.117
3.954
3.932
3.909
3.902
3.895
3.889
3.883
3.877
3.870
3.863
2.468
2.465
2.461
2.457
2.453
2.129
2.065
0.893
0.150
0.119
AcO
AcO
TBSO
HO
O
N=N
N
N
Ph
40 (CDCl3, 400 MHz)
NMR
Solvent Peak
H2O Peak
2.04
3.12
1.00
1.00
1.02
1.05
1.10
1.06
0.99
0.94
3.08
3.12
9.17
3.07
3.01
f1 (ppm)
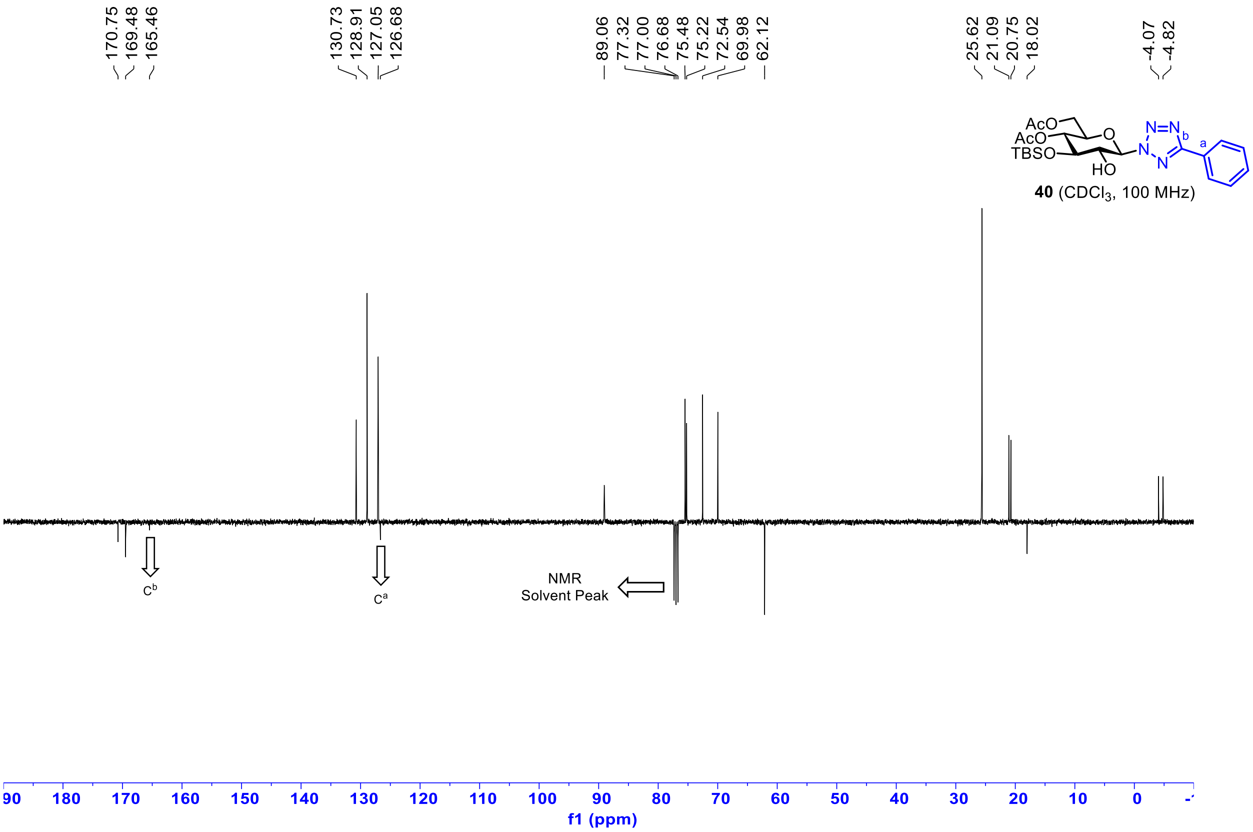
170.75
169.48
165.46
130.73
128.91
127.05
126.68
89.06
77.32
77.00
76.68
75.48
75.22
72.54
69.98
62.12
25.62
21.09
20.75
18.02
-4.07
-4.82
AcO
AcO
TBSO
HO
O
N=N
N
N
b
a
40 (CDCl3, 100 MHz)
Cb
Ca
NMR
Solvent Peak
f1 (ppm)

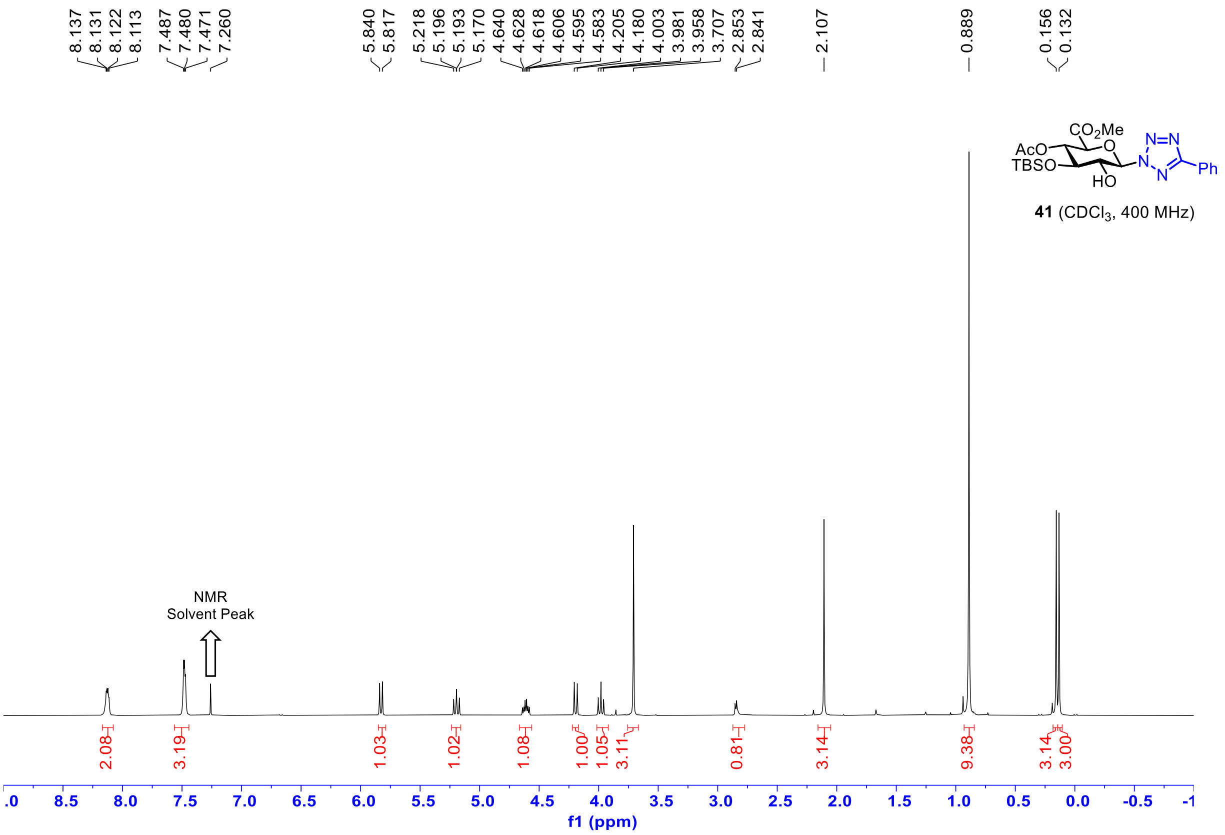
8.137
8.131
8.122
8.113
7.487
7.480
7.471
7.260
5.840
5.817
5.218
5.196
5.193
5.170
4.640
4.628
4.618
4.606
4.595
4.583
4.205
4.180
4.003
3.981
3.958
3.707
2.853
2.841
2.107
0.889
0.156
0.132
CO2Me
AcO
TBSO
O
HO
N=N
N
N
Ph
41 (CDCl3, 400 MHz)
NMR
Solvent Peak
2.08
3.19
1.03
1.02
1.08
1.00
1.05
3.11
0.81
3.14
9.38
3.14
3.00
f1 (ppm)

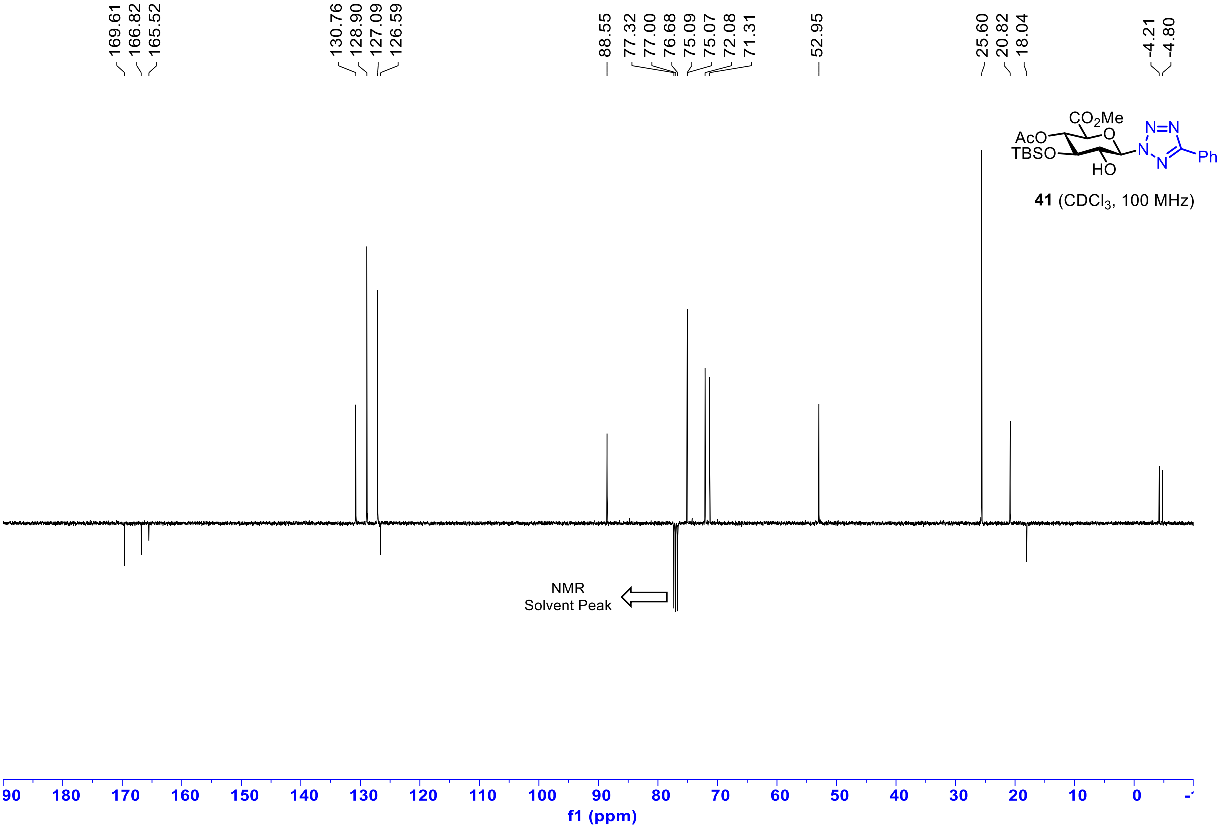
169.61
166.82
165.52
130.76
128.90
127.09
126.59
88.55
77.32
77.00
76.68
75.09
75.07
72.08
71.31
52.95
25.60
20.82
18.04
-4.21
-4.80
CO2Me
AcO
TBSO
O
HO
N=N
N
N
Ph
41 (CDCl3, 100 MHz)
NMR
Solvent Peak
f1 (ppm)

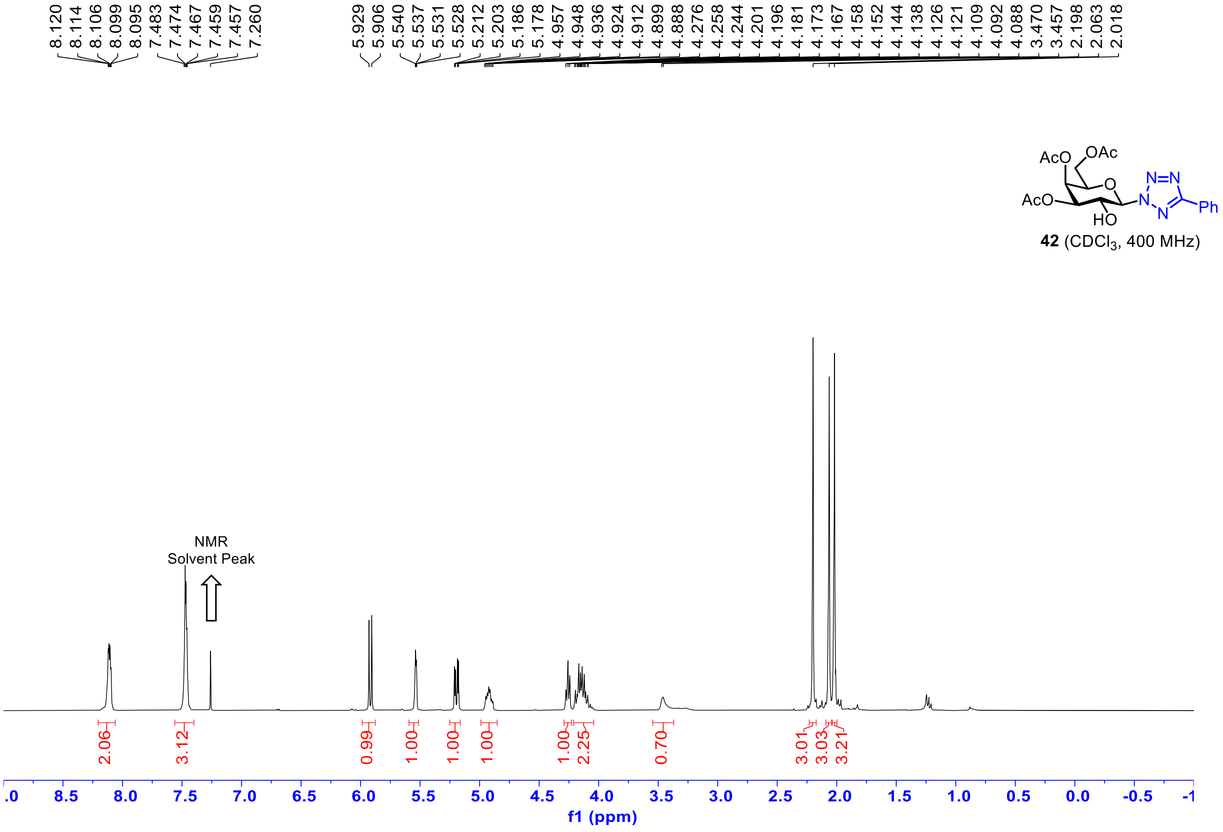

42 ($CDCl_3$, 400 MHz)

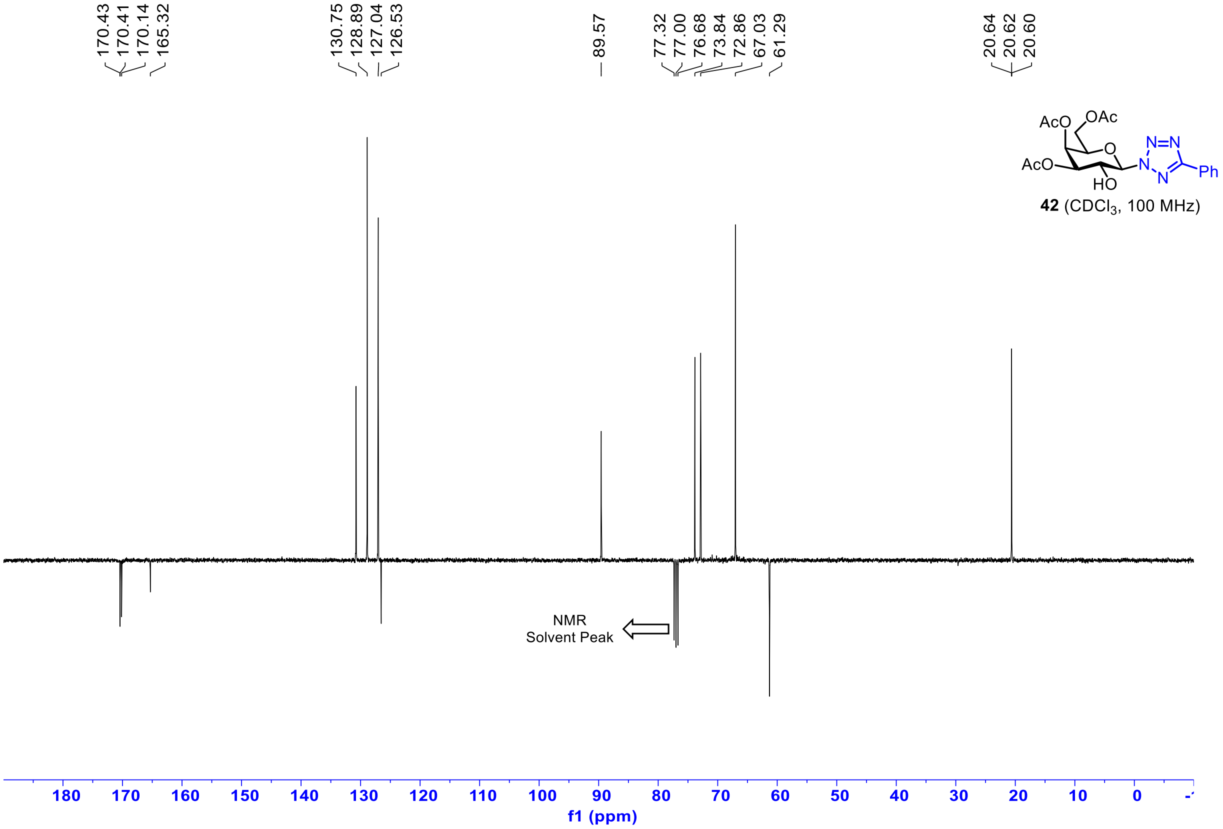

42 ($CDCl_3$, 100 MHz)

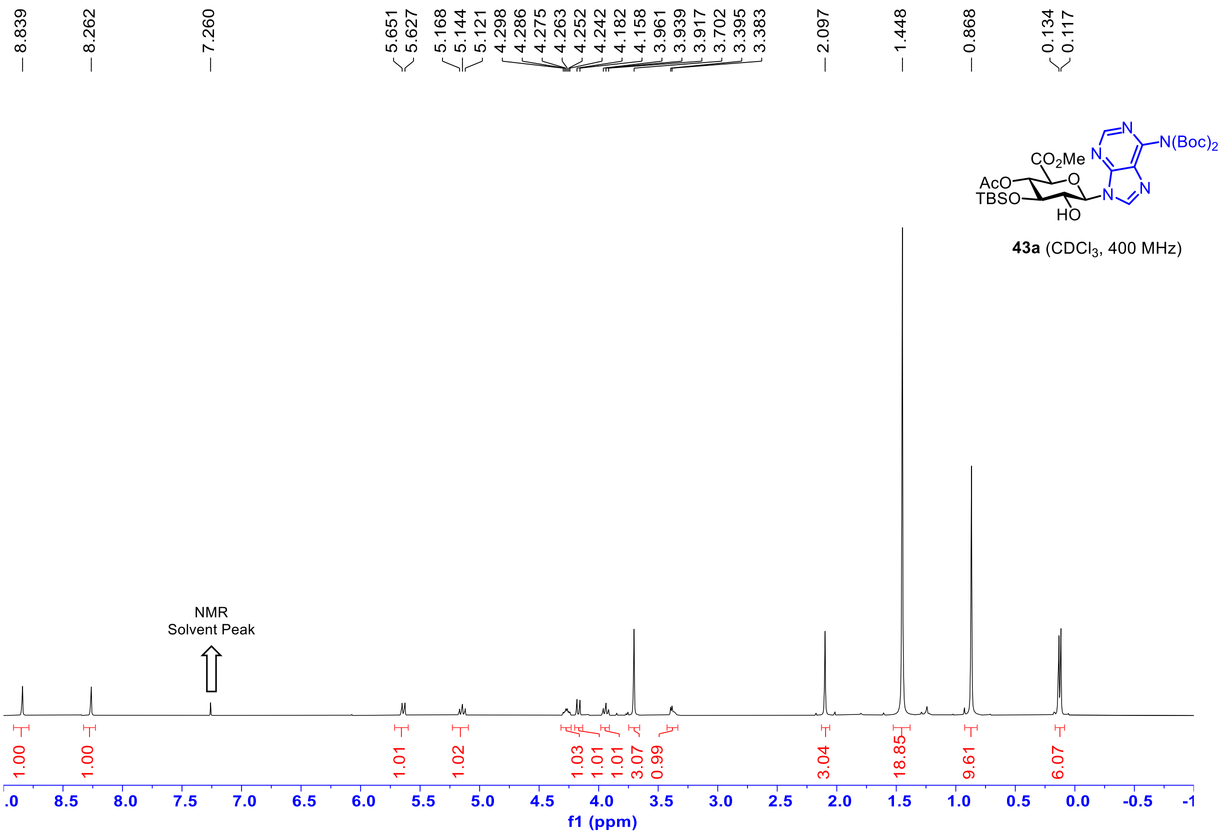
8.839
8.262
7.260
5.651
5.627
5.168
5.144
5.121
4.298
4.286
4.275
4.263
4.252
4.242
4.182
4.158
3.961
3.939
3.917
3.702
3.395
3.383
2.097
1.448
0.868
0.134
0.117
CO2Me
N(Boc)2
AcO
TBSO
HO
43a (CDCl3, 400 MHz)
NMR Solvent Peak
1.00
1.00
1.01
1.02
1.03
1.01
1.01
3.07
0.99
3.04
18.85
9.61
6.07
f1 (ppm)

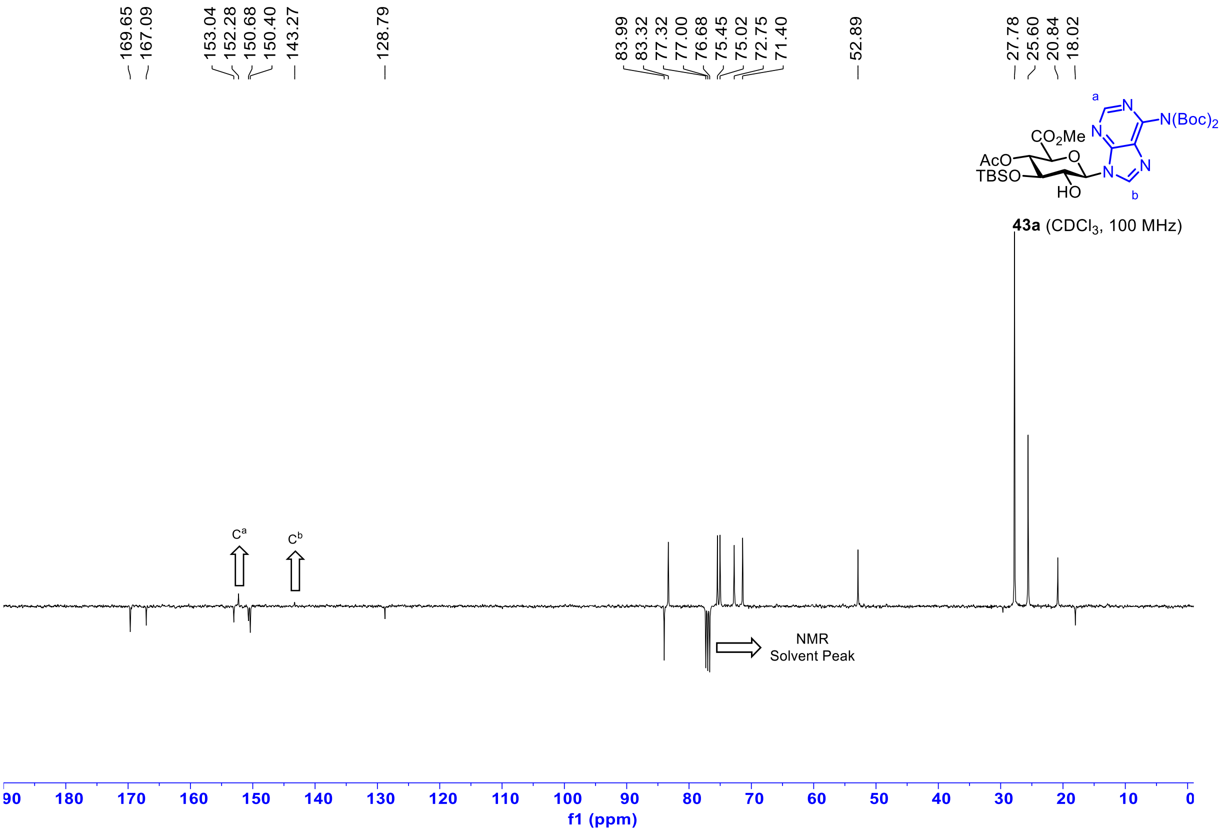
169.65
167.09
153.04
152.28
150.68
150.40
143.27
128.79
83.99
83.32
77.32
77.00
76.68
75.45
75.02
72.75
71.40
52.89
27.78
25.60
20.84
18.02
CO2Me
N(Boc)2
AcO
TBSO
HO
a
b
43a (CDCl3, 100 MHz)
Ca
Cb
NMR Solvent Peak
f1 (ppm)

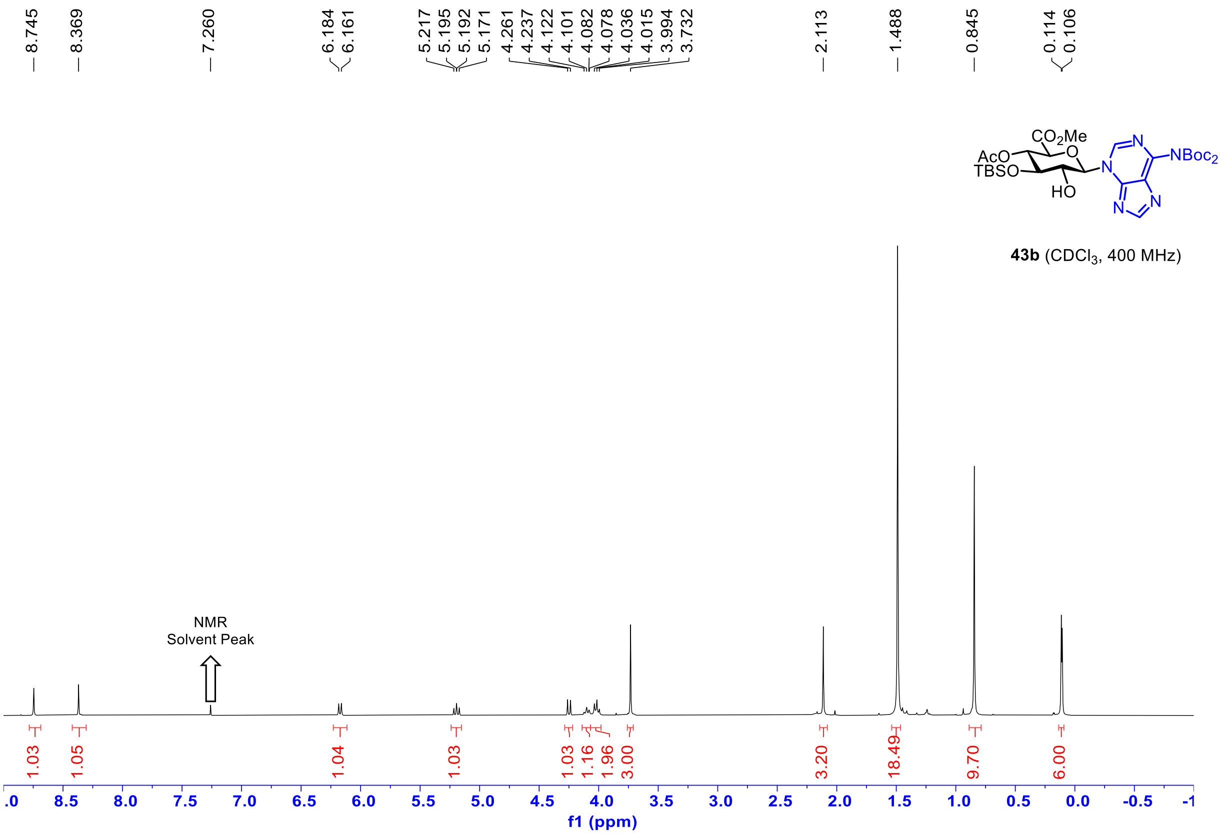
8.745
8.369
7.260
6.184
6.161
5.217
5.195
5.192
5.171
4.261
4.237
4.122
4.101
4.082
4.078
4.036
4.015
3.994
3.732
2.113
1.488
0.845
0.114
0.106
CO2Me
AcO
TBSO
HO
NBoc2
43b (CDCl3, 400 MHz)
NMR
Solvent Peak
1.03
1.05
1.04
1.03
1.03
1.16
1.96
3.00
3.20
18.49
9.70
6.00
f1 (ppm)

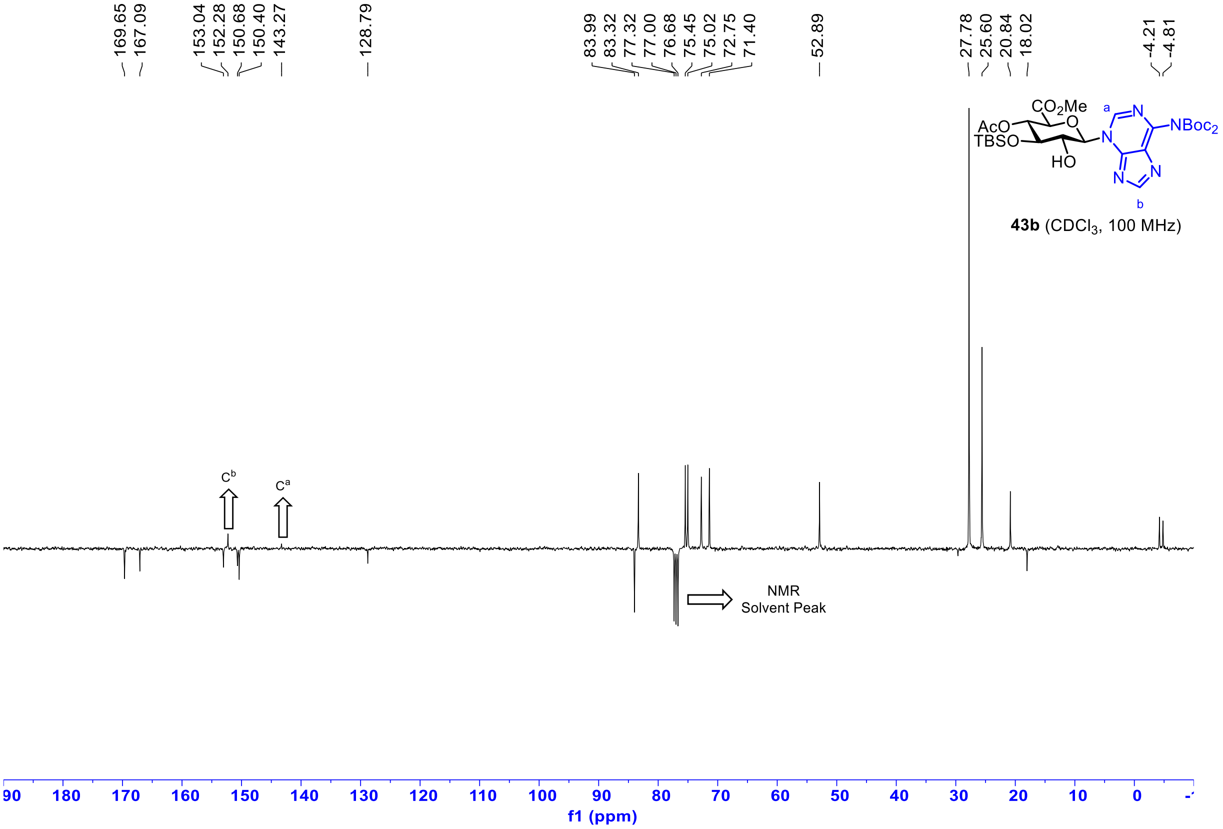
169.65
167.09
153.04
152.28
150.68
150.40
143.27
128.79
83.99
83.32
77.32
77.00
76.68
75.45
75.02
72.75
71.40
52.89
27.78
25.60
20.84
18.02
-4.21
-4.81
CO2Me
AcO
TBSO
HO
NBoc2
43b (CDCl3, 100 MHz)
NMR
Solvent Peak
f1 (ppm)

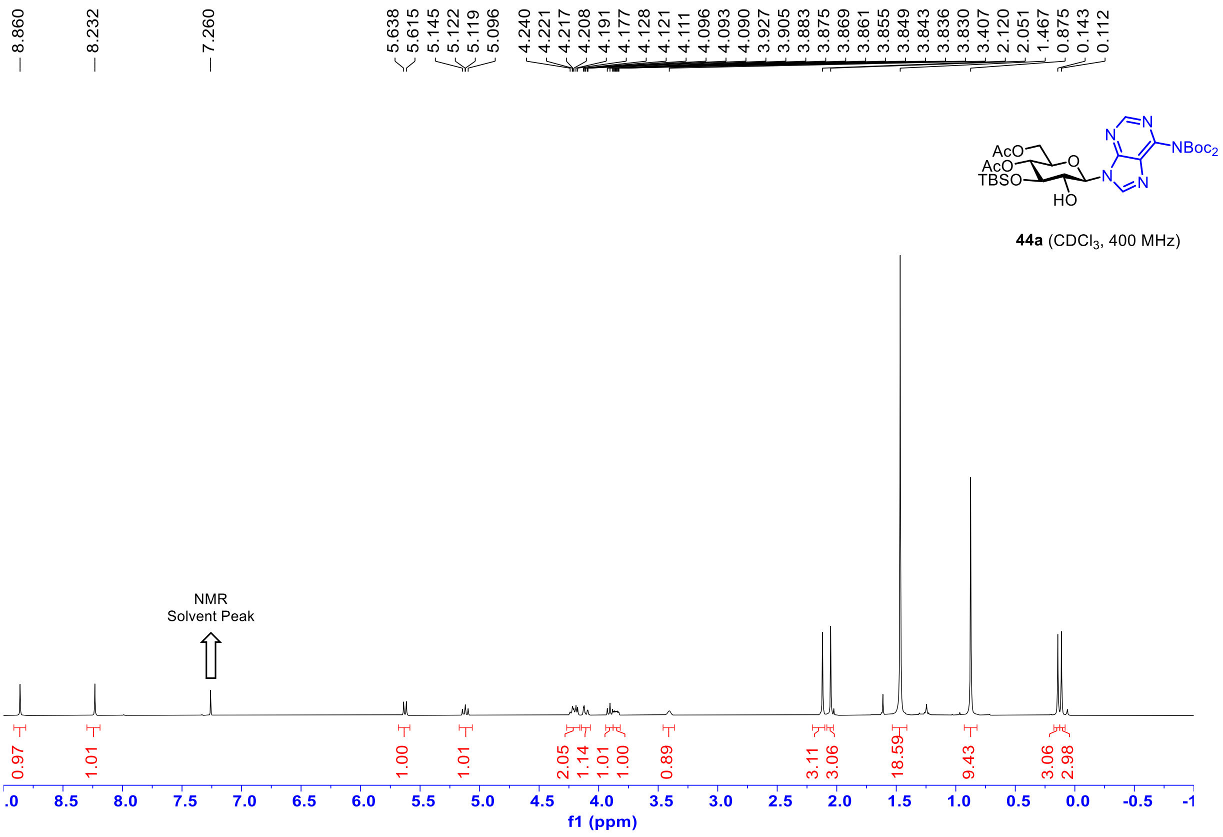

8.860
8.232
7.260
5.638
5.615
5.145
5.122
5.119
5.096
4.240
4.221
4.217
4.208
4.191
4.177
4.128
4.121
4.111
4.096
4.093
4.090
3.927
3.905
3.883
3.875
3.869
3.861
3.855
3.849
3.843
3.836
3.830
3.407
2.120
2.051
1.467
0.875
0.143
0.112
AcO
AcO
TBSO
O
HO
N
N
N
N
NBoc2
44a (CDCl3, 400 MHz)
NMR
Solvent Peak
0.97
1.01
1.00
1.01
2.05
1.14
1.01
1.00
0.89
3.11
3.06
18.59
9.43
3.06
2.98
f1 (ppm)

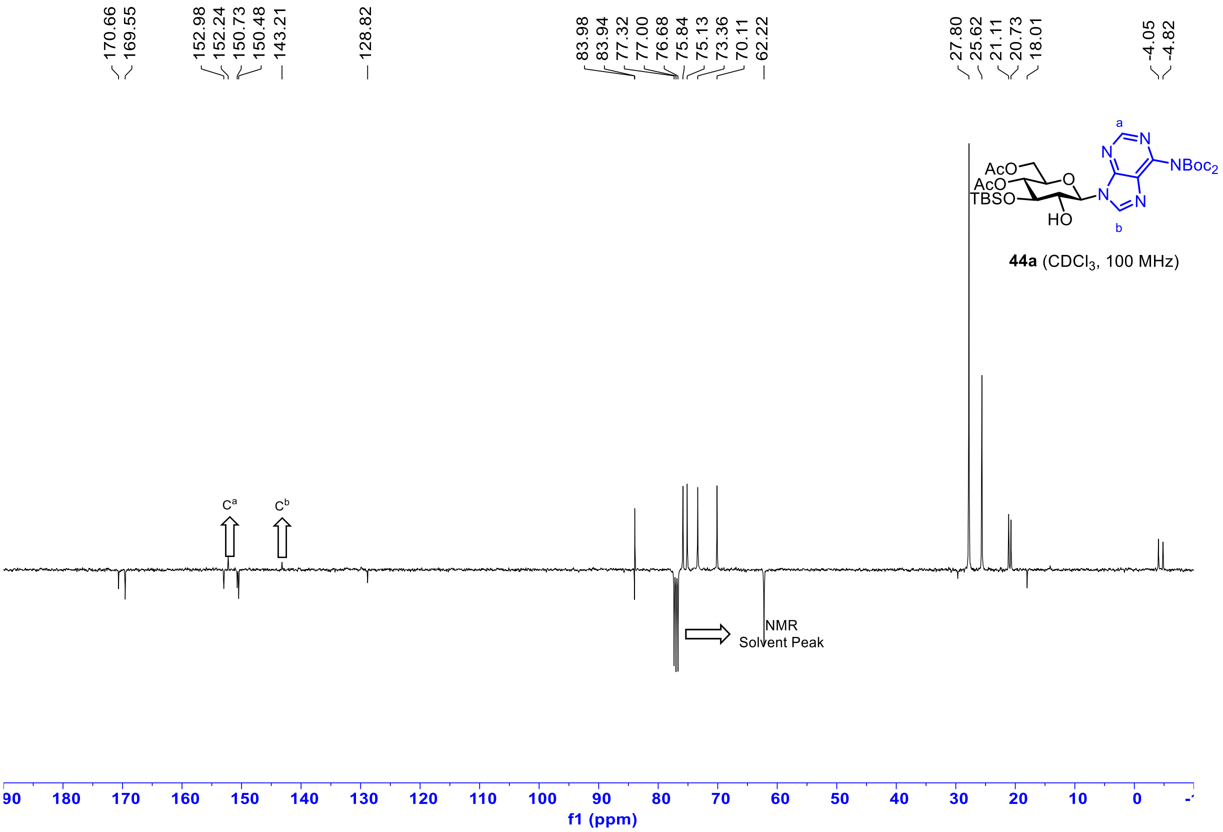

170.66
169.55
152.98
152.24
150.73
150.48
143.21
128.82
83.98
83.94
77.32
77.00
76.68
75.84
75.13
73.36
70.11
62.22
27.80
25.62
21.11
20.73
18.01
-4.05
-4.82
AcO
AcO
TBSO
O
HO
N
N
N
N
NBoc2
a
b
44a (CDCl3, 100 MHz)
Ca
Cb
NMR
Solvent Peak
f1 (ppm)

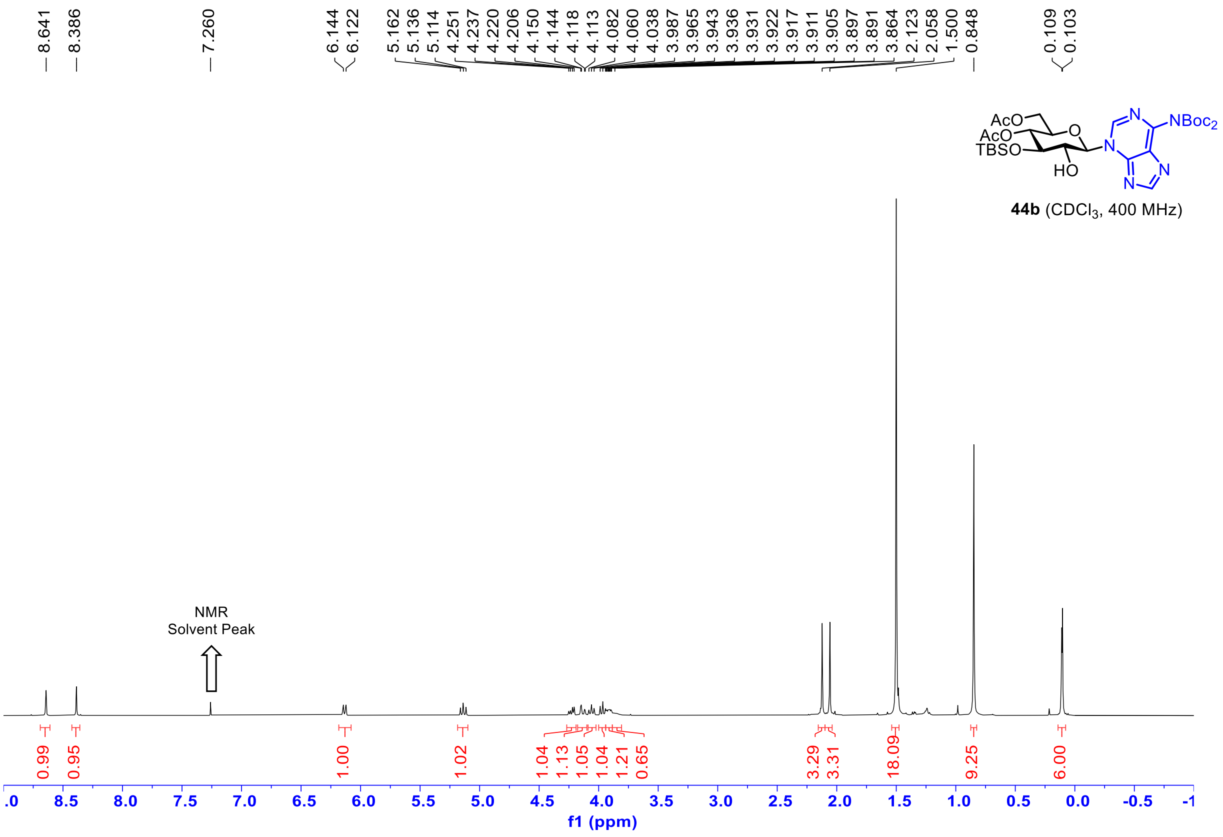
8.641
8.386
7.260
6.144
6.122
5.162
5.136
5.114
4.251
4.237
4.220
4.206
4.150
4.144
4.118
4.113
4.082
4.060
4.038
3.987
3.965
3.943
3.936
3.931
3.922
3.917
3.911
3.905
3.897
3.891
3.864
2.123
2.058
1.500
0.848
0.109
0.103
AcO
AcO
TBSO
HO
NBoc2
44b (CDCl3, 400 MHz)
NMR
Solvent Peak
0.99
0.95
1.00
1.02
1.04
1.13
1.05
1.04
1.21
0.65
3.29
3.31
18.09
9.25
6.00
f1 (ppm)

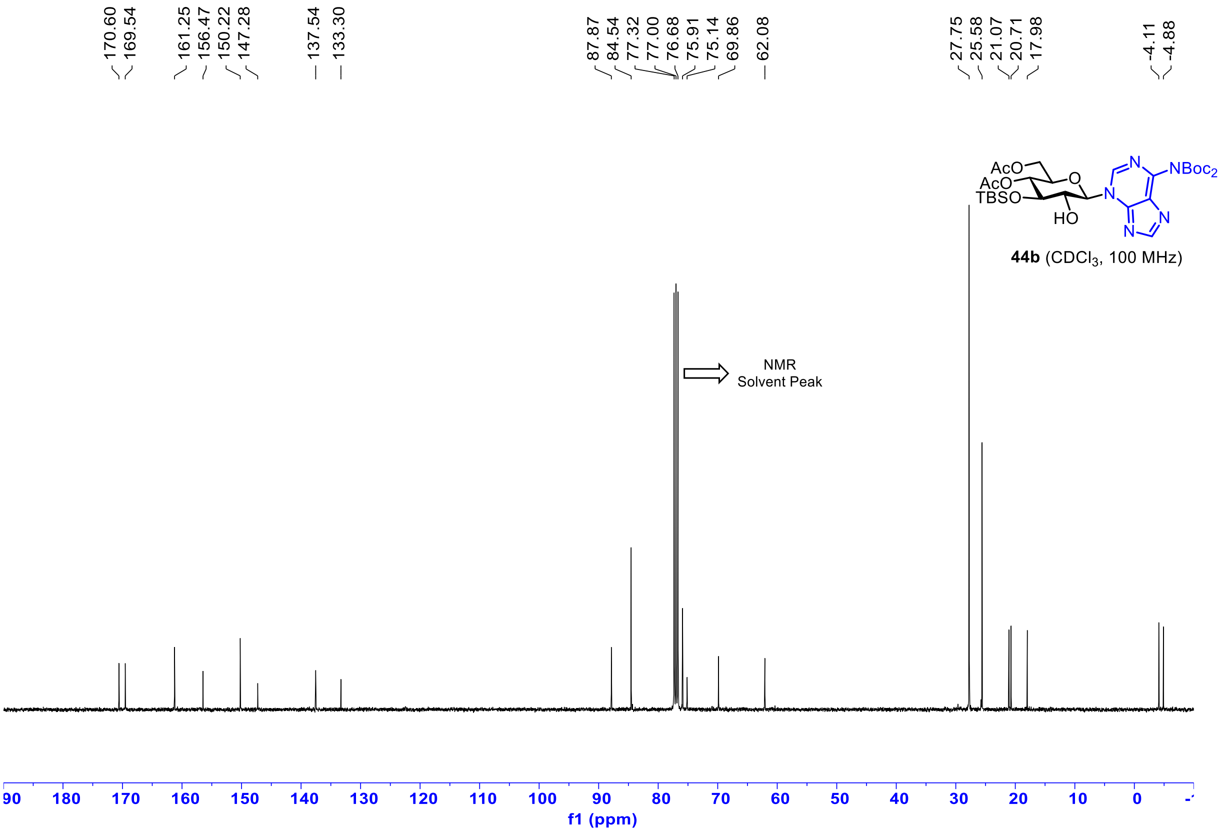
170.60
169.54
161.25
156.47
150.22
147.28
137.54
133.30
87.87
84.54
77.32
77.00
76.68
75.91
75.14
69.86
62.08
27.75
25.58
21.07
20.71
17.98
-4.11
-4.88
AcO
AcO
TBSO
HO
NBoc2
44b (CDCl3, 100 MHz)
NMR
Solvent Peak
f1 (ppm)

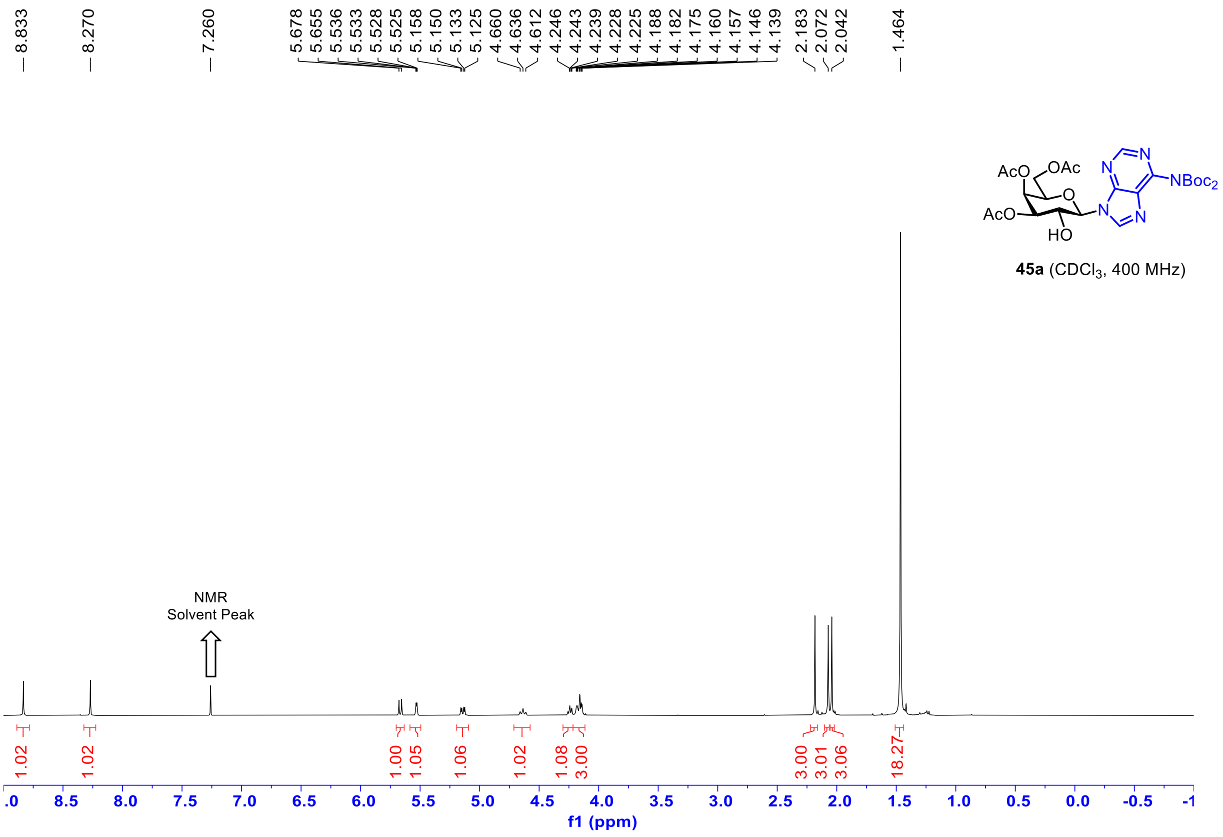

45a ($CDCl_3$, 400 MHz)

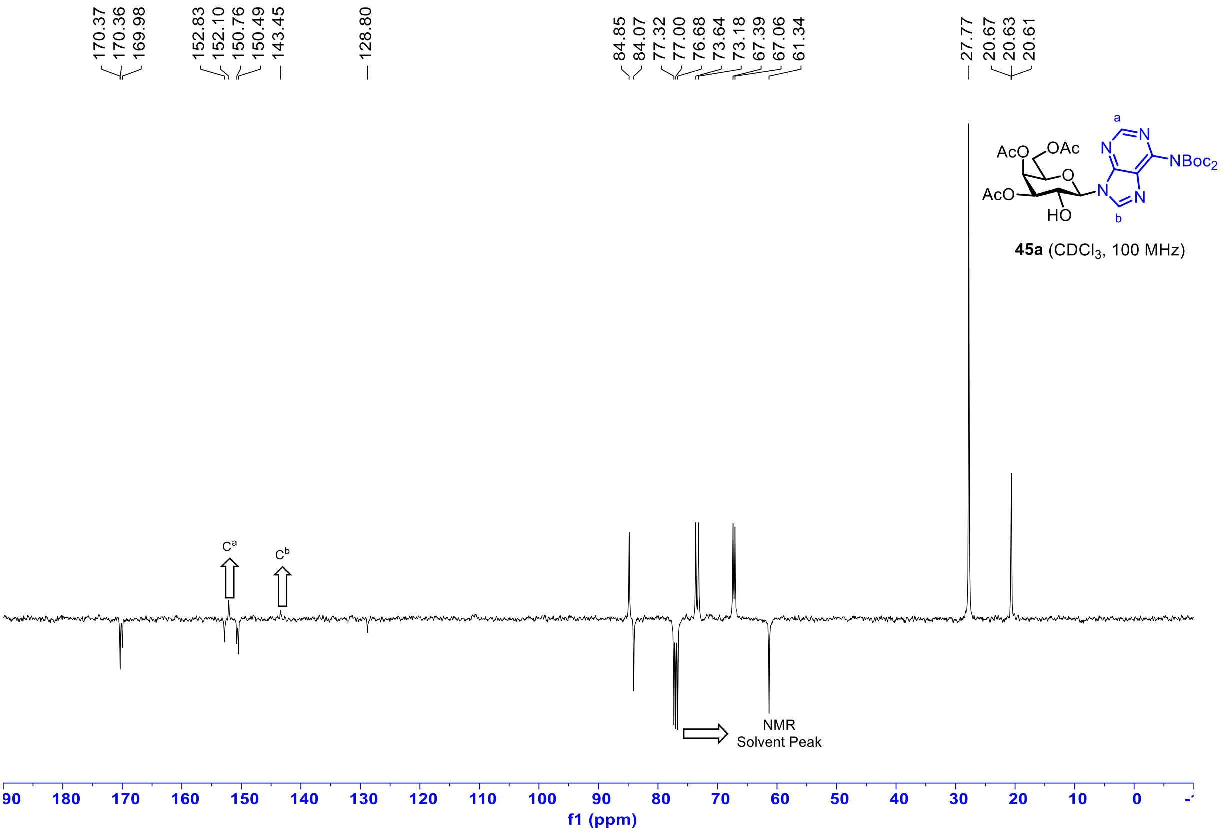

45a ($CDCl_3$, 100 MHz)

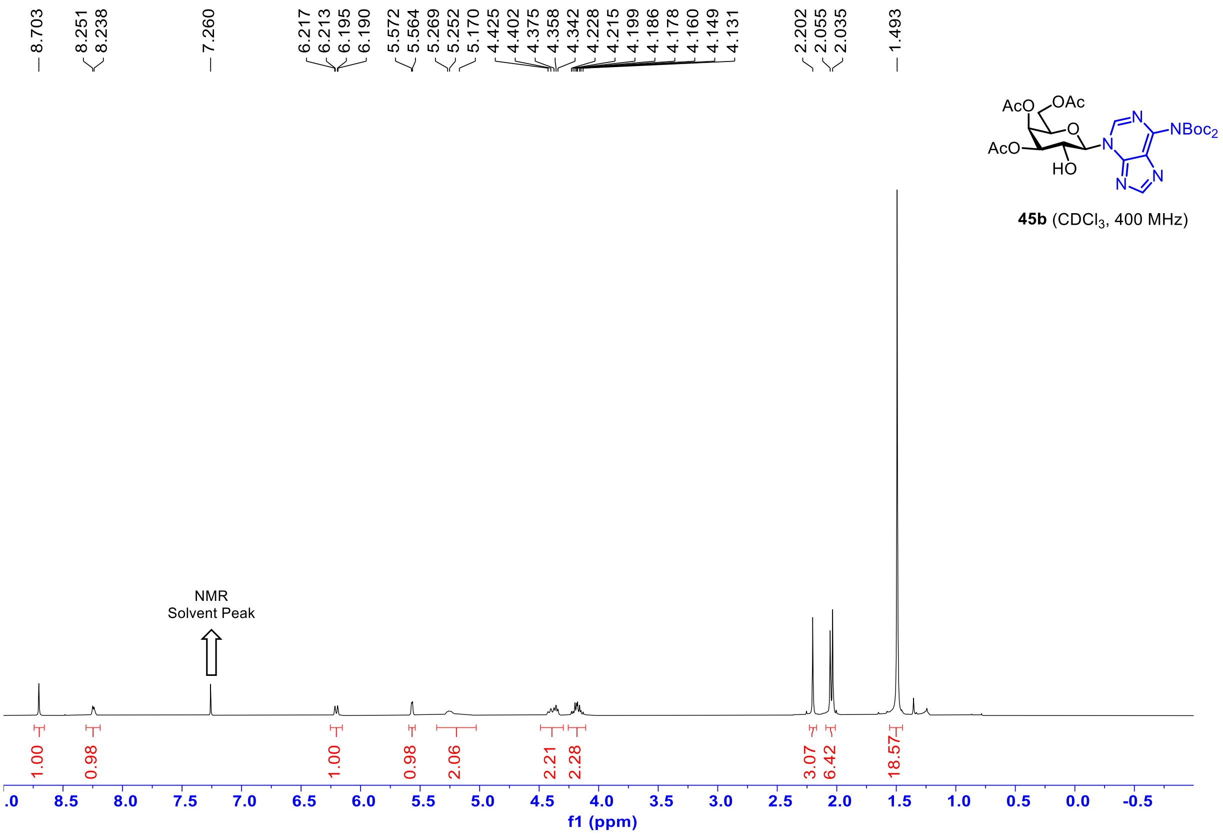

45b ($CDCl_3$, 400 MHz)

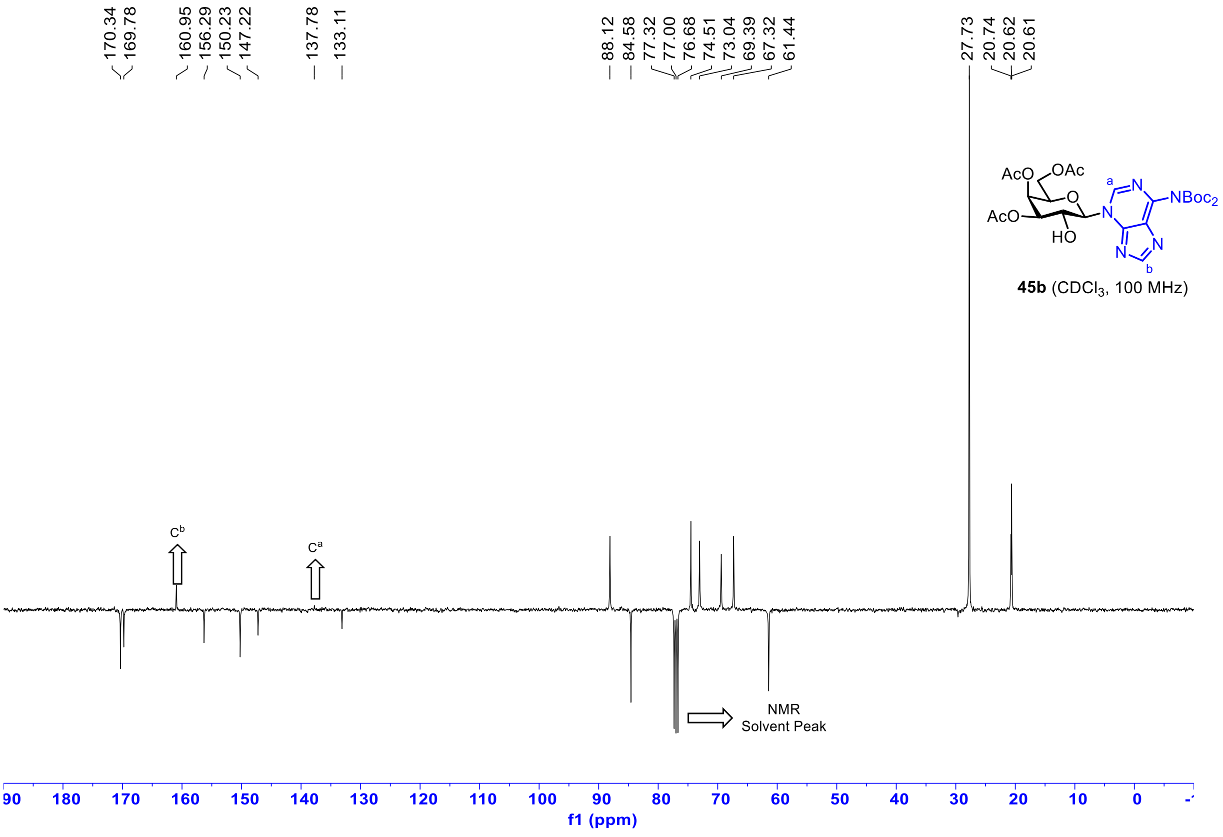

45b ($CDCl_3$, 100 MHz)

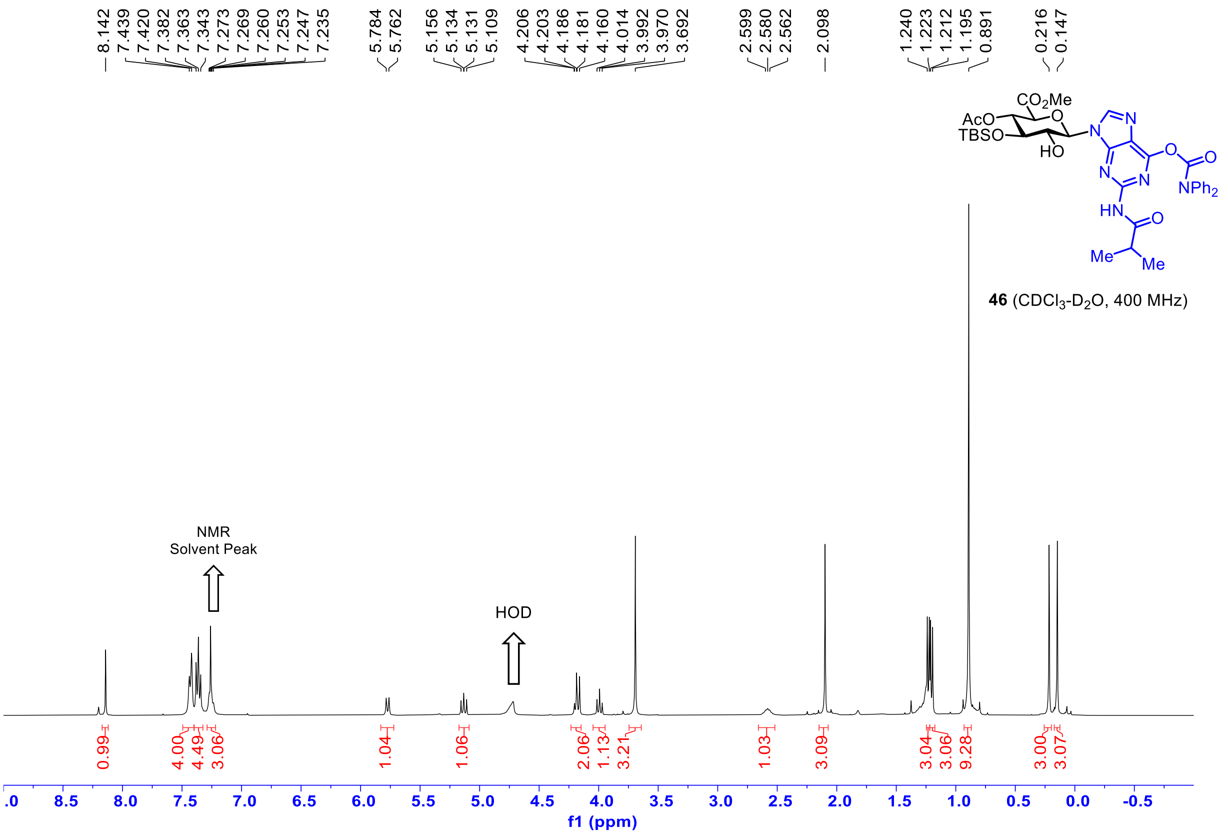

8.142
7.439
7.420
7.382
7.363
7.343
7.273
7.269
7.260
7.253
7.247
7.235
5.784
5.762
5.156
5.134
5.131
5.109
4.206
4.203
4.186
4.181
4.160
4.014
3.992
3.970
3.692
2.599
2.580
2.562
2.098
1.240
1.223
1.212
1.195
0.891
0.216
0.147
AcO
TBSO
CO2Me
O
HO
N
N
O
O
NPh2
N
N
HN
O
Me
Me
46 (CDCl3-D2O, 400 MHz)
NMR Solvent Peak
HOD
0.99
4.00
4.49
3.06
1.04
1.06
2.06
1.13
3.21
1.03
3.09
3.04
3.06
9.28
3.00
3.07
f1 (ppm)

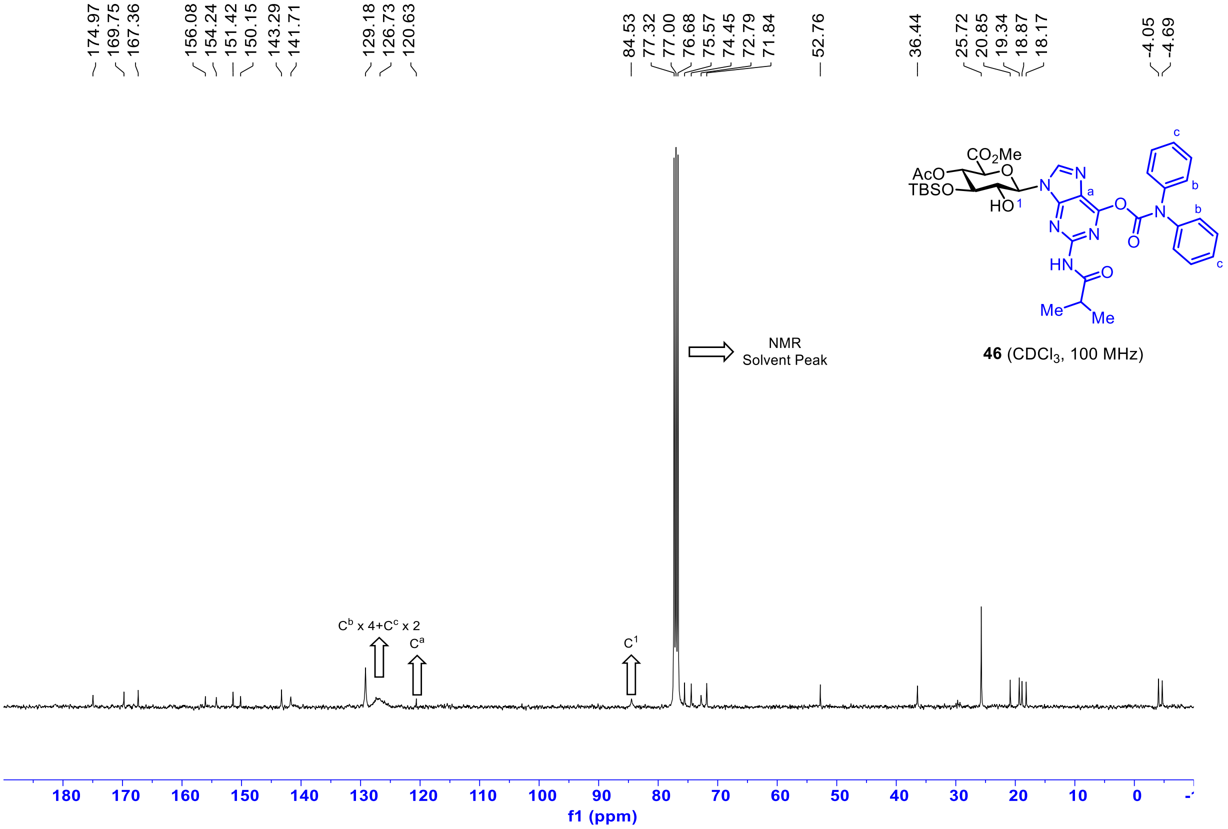

174.97
169.75
167.36
156.08
154.24
151.42
150.15
143.29
141.71
129.18
126.73
120.63
84.53
77.32
77.00
76.68
75.57
74.45
72.79
71.84
52.76
36.44
25.72
20.85
19.34
18.87
18.17
-4.05
-4.69
AcO
TBSO
CO2Me
O
HO 1
N
N
a
O
N
O
b
b
c
c
N
N
HN
O
Me
Me
46 (CDCl3, 100 MHz)
NMR Solvent Peak
Cb x 4+Cc x 2
Ca
C1
f1 (ppm)

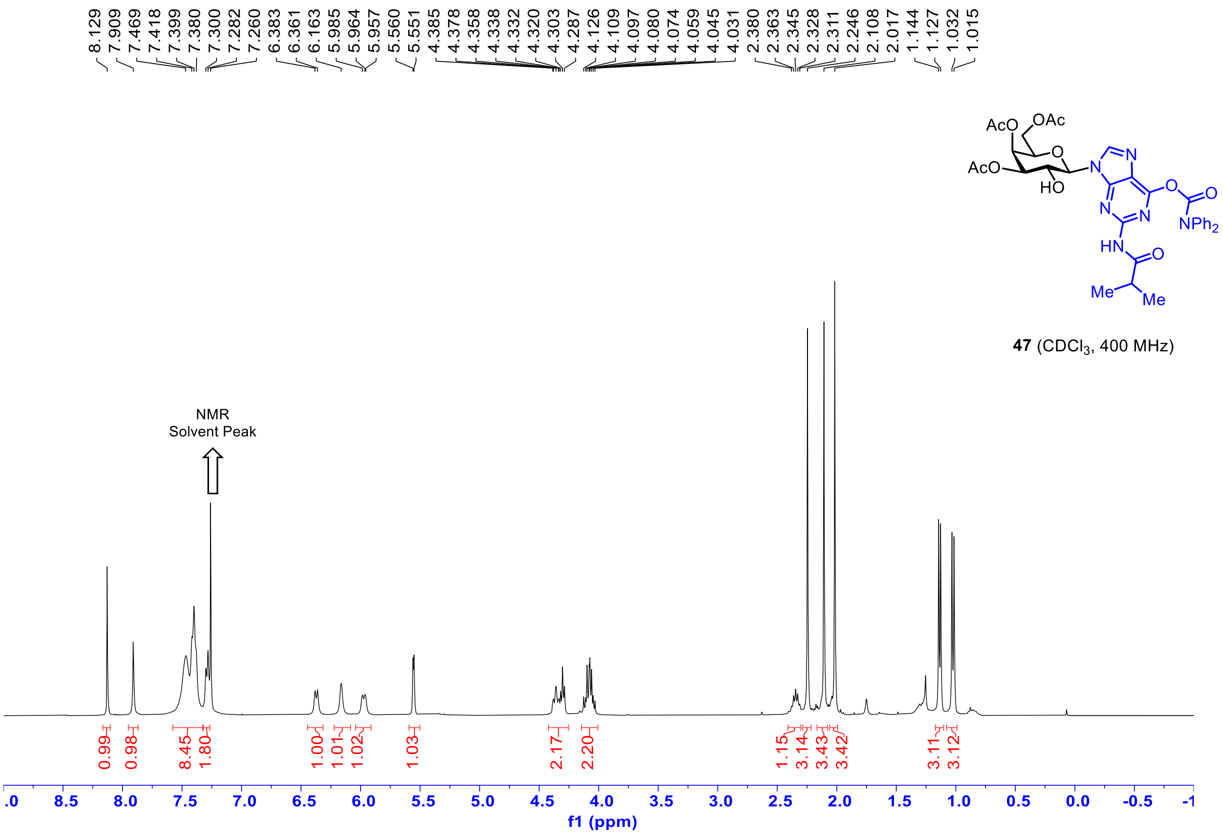

47 ($CDCl_3$, 400 MHz)

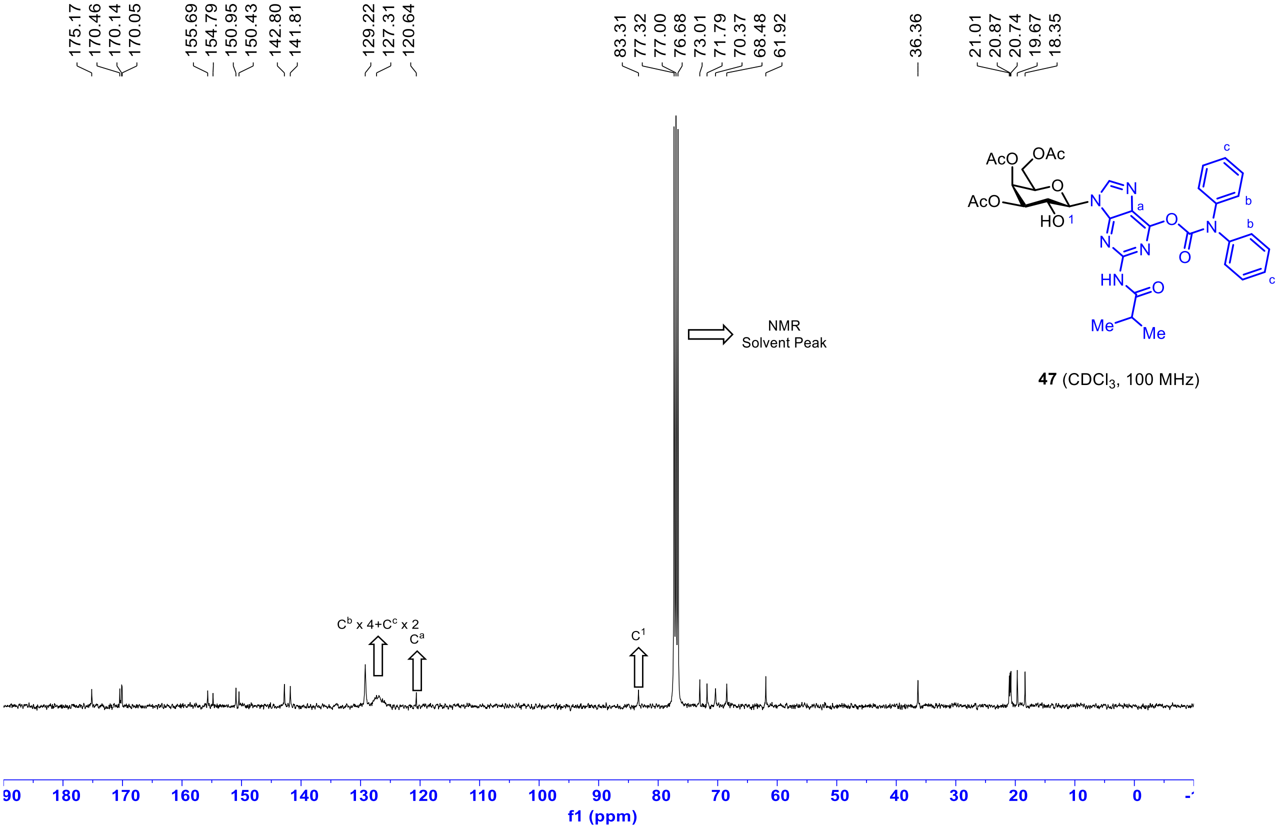

47 ($CDCl_3$, 100 MHz)
